# Combination of epigenetic regulation with gene therapy-mediated immune checkpoint blockade induces anti-tumour effects and immune response in vivo

**DOI:** 10.1038/s41467-021-27078-x

**Published:** 2021-11-18

**Authors:** Huapan Fang, Zhaopei Guo, Jie Chen, Lin Lin, Yingying Hu, Yanhui Li, Huayu Tian, Xuesi Chen

**Affiliations:** 1 Key Laboratory of Polymer Ecomaterials, Changchun Institute of Applied Chemistry, Chinese Academy of Sciences, Changchun, 130022 China; 2 University of Science and Technology of China, Hefei, 230026 China; 3 Jilin Biomedical Polymers Engineering Laboratory, Changchun, 130022 China; 4 Jiangsu Key Laboratory for Carbon Based Functional Materials and Devices, Institute of Functional Nano and Soft Materials (FUNSOM), Soochow University, Suzhou, Jiangsu 215123 China; 5 School of Materials Science and Engineering, Changchun University of Science and Technology, Changchun, 130022 China

**Keywords:** DNA and RNA, Drug delivery, Cancer immunotherapy, Tumour immunology

## Abstract

Immunotherapy has become a powerful cancer treatment, but only a small fraction of patients have achieved durable benefits due to the immune escape mechanism. In this study, epigenetic regulation is combined with gene therapy-mediated immune checkpoint blockade to relieve this immune escape mechanism. **PPD** (i.e., m**P**EG-b-PLG/**P**EI-RT3/**D**NA) is developed to mediate plasmid-encoding shPD-L1 delivery by introducing multiple interactions (i.e., electrostatic, hydrogen bonding, and hydrophobic interactions) and polyproline II (PPII)-helix conformation, which downregulates PD-L1 expression on tumour cells to relieve the immunosuppression of T cells. Zebularine (abbreviated as Zeb), a DNA methyltransferase inhibitor (DNMTi), is used for the epigenetic regulation of the tumour immune microenvironment, thus inducing DC maturation and MHC I molecule expression to enhance antigen presentation. PPD plus Zeb combination therapy initiates a systemic anti-tumour immune response and effectively prevents tumour relapse and metastasis by generating durable immune memory. This strategy provides a scheme for tumour treatment and the inhibition of relapse and metastasis.

## Abstract (web summary)

While immunotherapy is a promising cancer treatment option, durable benefits are often rare due to immune escape. Here, the authors combine epigenetic regulation with gene therapy-mediated immune checkpoint blockade and show efficient anti-tumour effects and immune response in vivo.

## Introduction

Immunotherapy has become a powerful treatment strategy for cancer in the past few years and checkpoint inhibitors have revolutionized the field of oncology by activating the anti-tumour immune response^1,2^. However, clinical trials have shown that only a small fraction of patients acquired durable benefits^3,4^ mainly due to the immune escape mechanism in cancer^5–7^. One of the reasons for this is the low levels of tumour antigens^8,9^ and insufficient ability of antigen-presenting cells (APCs) to present antigens to T cells^10,11^, all of which cause poor cytotoxic T-lymphocyte responses. Equally important is that T cells have immune checkpoint-dependent immunosuppression, e.g., programmed cell death 1 (PD-1) receptor expressed on T cells binds to its two ligands, PD-1 ligand 1 (PD-L1) and ligand 2 (PD-L2), which are upregulated on tumour cells. This results in T cell failing to exert its effector functions in tumour immune surveillance^12–14^.

Generally speaking, tumour cell fragments could be used as tumour antigens, which were achieved by photothermal therapy^15,16^, photodynamic therapy^17,18^, and chemotherapy-mediated immunogenic cell death^19,20^. However, photothermal and photodynamic therapies are susceptible to photothermal agents, photosensitizers, and light. In addition, chemotherapeutic agents usually have severe toxic side effects on normal tissues^21,22^. Therefore, an efficient and safe strategy is urgently needed to generate an ideal condition to boost antigen-specific immune response. Recently, it has been reported that epigenetic therapy such as DNA methyltransferase inhibitors (DNMTi) could not only upregulate the expression of tumour-associated antigens but also induce the enhancement of major histocompatibility complex (MHC) class I molecules on tumour cells via cancer testis antigen (CTA), thereby ultimately mounting the visibility of tumour cells to the adaptive immune system^23–26^.

Moreover, immune checkpoints are crucial regulatory pathways that invalidate T-cell-mediated tumour killing^27^. The PD-1 receptor is a crucial immune checkpoint molecule and is mainly expressed on mature cytotoxic T lymphocytes (CTLs) in peripheral tissues and the tumour microenvironment^12,28^. PD-1 signaling is mediated via the engagements of PD-L1 and PD-L2, resulting in T-cell exhaustion. A number of monoclonal antibodies targeting PD-1 or PD-L1 have demonstrated beneficial treatments against several tumour types, including melanoma^29,30^, hepatic carcinoma^31^, non-small cell lung cancer^32^, and Hodgkin’s lymphoma^33^. Nevertheless, effectiveness in only a minority of patients and resistance after initial response are usually observed^34^. Moreover, PD-1 or PD-L1 targeted antibody therapies may cause severe side effects, such as immune-related adverse events (irAEs) associated with inflammation and toxicity^35,36^. Gene therapy-guided immune checkpoint blockade could be used as an alternative strategy of reversing T-cell exhaustion while avoiding the side effects caused by PD-1/PD-L1-targeted antibody therapies.

**Fig. 1:**
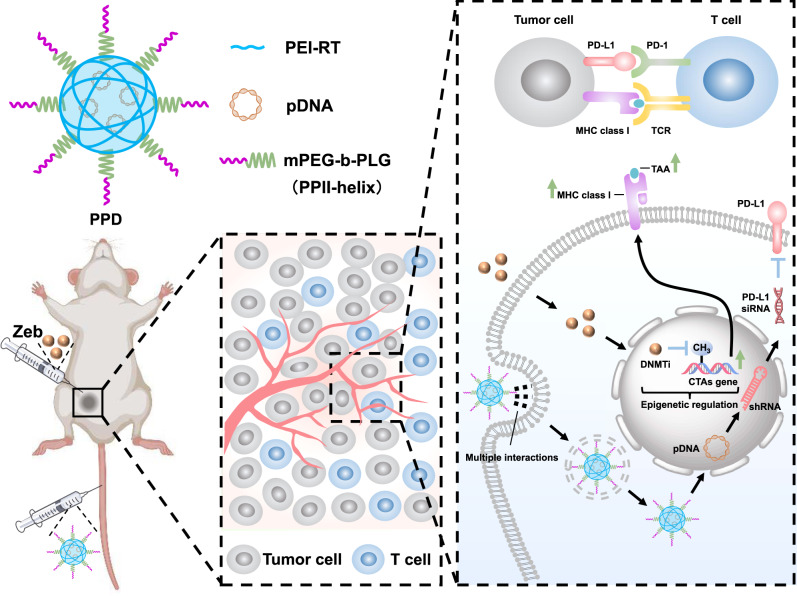
Illustration of combination of epigenetic regulation with gene therapy-mediated immune checkpoint blockade induces anti-tumour effects and immune response in vivo. CTAs: cancer testis antigens, DNMTi: DNA methyltransferase inhibitor, PPII-helix: polyproline II-helix, TAA: tumour-associated antigen, Zeb: zebularine. Multiple interactions include electrostatic, hydrogen bonding, and hydrophobic interactions.

Herein, an efficient strategy of utilizing epigenetic regulation combined with gene therapy-guided immune checkpoint blockade is described, which simultaneously increases the levels of tumour antigens, enhances the ability of APCs to present antigens and reverses T-cell exhaustion (Fig. 1). More specifically, zebularine (Zeb), a DNMTi, is paratumourally administered to tumour-bearing mice, to increase the levels of tumour antigens and boost the ability of APCs to present antigens to T cells; meanwhile, a polymeric gene delivery system (mPEG-b-PLG/PEI-RT3/DNA, abbreviated as PPD) is developed to mediate plasmid DNA encoding shPD-L1 (denoted as shPD-L1) delivery in vivo to reverse T-cell exhaustion. In vivo results show that PPD plus Zeb greatly inhibits tumour growth and efficiently prevents its relapse and metastasis by activating a systemic anti-tumour immune memory effect after treatment.

## Results

### Efficient polymeric gene delivery system

Polycationic gene carriers have drawn increasing attention because of their low immunogenicity, high loading efficiency, and abundant sources^37,38^. Among them, polyethylenimine with a molecular weight of 25 kDa (denoted as PEI25k) was recognized as the gold standard^39,40^; however, its severe cytotoxicity limited its clinical application^41^. Although low-molecular-weight polyethylenimine such as PEI1.8k (polyethyleneimine with a molecular weight of 1800 Da) had low cytotoxicity, its poor transfection could not live up to expectations. Herein, ‘molecular string’ *p*-toluene sulfonyl arginine (abbreviated as RT) was introduced onto PEI1.8k and its effect on the transfection capability and biocompatibility of PEI1.8k was studied.

Different amounts of ‘molecular string’ RT were introduced onto PEI1.8k through amide condensation and the products were named PEI-RTs (Supplementary Fig. 1). The ^1^H nuclear magnetic resonance (NMR) spectra indicated the successful synthesis of PEI-RTs (Supplementary Fig. 2). The number of grafted ‘molecular string’ RT was 4.5, 6.8, 10.2, and 13.0 for the polycations named as PEI-RT1, PEI-RT2, PEI-RT3, and PEI-RT4, respectively (Supplementary Table 1). DNA transfection of these polycations was then performed in various cancer cell lines (B16F10, 4T1, HeLa, and MCF-7). Once PEI1.8k was modified by the RT string, the transfection efficiency showed a remarkable increase in B16F10 cells. Different amounts of ‘molecular string’ RT-modified PEI1.8k exhibited significant differences in DNA transfection (Supplementary Fig. 3a). When the number of grafted ‘molecular string’ RT increased, the transfection efficiencies of PEI-RTs showed a tendency to increase first and then decrease. At the same mass ratio of carrier/DNA, PEI-RT3 had a considerably higher transfection efficiency than other PEI-RTs. Moreover, the optimal transfection efficiency of PEI-RT3/DNA (PD)at a mass ratio of 5/1 was 196-fold higher than that of PEI1.8k/DNA (ED) and 8.2-fold higher than that of PEI25k/DNA (Supplementary Fig. 3b). Similar results were obtained in 4T1, HeLa, and MCF-7 cells (Supplementary Figs. 4–6). The optimal transfection efficiencies of PD in 4T1, HeLa, and MCF-7 cells were 213-, 368-, and 583-fold higher than that of ED, respectively, and 5.2-, 6.5-, and 16.8-fold higher than that of PEI25k/DNA, respectively. Therefore, introducing ‘molecular string’ RT onto PEI1.8k could remarkably enhance the transfection efficiency of PEI1.8k in cancer cells.

**Fig. 2:**
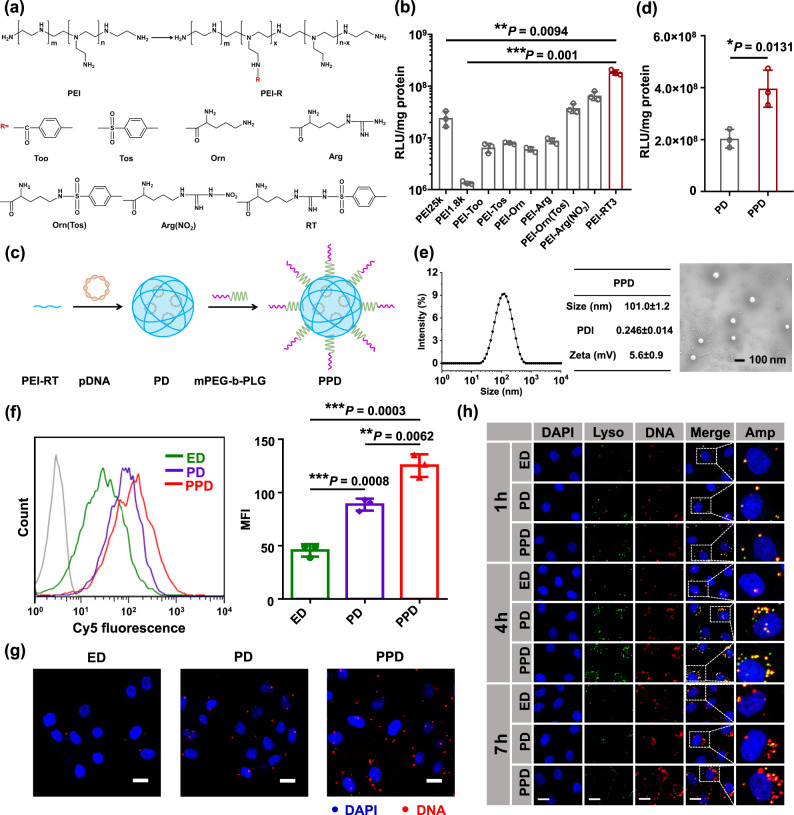
Construction of efficient polycationic gene carrier. **a** PEI modified with different ‘molecular strings’. **b** Optimal DNA transfection efficiency of PEI1.8k grafting with various ‘molecular strings’ in B16F10 cells. **c** Preparation diagram of PPD. **d** Optimal transfection efficiency of PPD and PD in B16F10 cells. **e** Hydrodynamic particle size and scanning electron microscopy image of PD. **f** Cellular uptake of PD and PPD at 3 h by flow cytometry in B16F10 cells. DNA was labeled with Cy5, ED was used as control. **g** Representative cellular uptake images of PPD, PD, and ED at 3 h in B16F10 cells, scale bar: 20 μm. **h** Representative endo/lysosomal escape images of PPD, PD, and ED at various time pointes (1, 4 and 7 h) in B16F10 cells, scale bar: 20 μm. Data in **b**, **d**, and **f** are presented as mean ± SD, *n* = 3 biologically independent samples. *P*-values are calculated by the two-tailed Student’s *t*-test in **b**, **d**, and **f** as indicated in the figures. *P < 0.05, **P < 0.01, and ***P < 0.001. A representative image of three independent samples from each group is shown in **e**, **g**, and **h**. ED: PEI1.8k/DNA, PD: PEI-RT3/DNA, PPD: mPEG-b-PLG/PEI-RT3/DNA.

To explain the mechanism underlying PEI-RT3-efficient transfection, different kinds of ‘molecular strings’ were introduced onto PEI1.8k (Fig. 2a and Supplementary Figs. 7–12). ^1^H NMR spectra indicated that these polycations were successfully synthesized and the ‘molecular string’ numbers of PEI-Too, PEI-Tos, PEI-Orn, PEI-Arg, PEI-Orn(Tos), and PEI-Arg(NO_2_) were 9.8, 10.2, 10.6, 10.4, 10.7, and 10.8, respectively (Supplementary Figs. 13–18 and Supplementary Table 2). The transfection efficiencies of these polycations were measured in different cancer cell lines (B16F10, 4T1, HeLa, and MCF-7). As shown in Fig. 2b and Supplementary Fig. 19, the transfection results of these polycations in B16F10 cells exhibited the following characteristics: (1) the transfection capabilities of PEI1.8k grafted with different ‘molecular strings’ exhibited remarkable differences and those of all grafted polycations were significantly enhanced in B16F10 cells. (2) For ‘molecular string’ containing only hydrophobic groups, their corresponding polycations such as PEI-Too and PEI-Tos showed more efficient gene transfection than PEI1.8k. Introducing hydrophobic groups (i.e., phenyl group) enhanced the hydrophobic interactions between polycationic carrier and cell membrane (or DNA), and contributed to cellular uptake, which was beneficial for gene transfection. (3) For ‘molecular string’ containing only hydrophilic cationic groups, their corresponding polycationic carriers showed remarkably improved gene transfection efficiency. For PEI-Arg, introducing Arg string increased the hydrogen-bonding interactions between carrier and cell membrane (or DNA), thus accelerating transfection. For PEI-Orn, introducing Orn string increased the positive charge density of carrier/DNA and contributed to endocytosis. (4) For cationic ‘molecular string’ containing hydrophobic groups such as RT, Arg(NO_2_), and Orn(Tos), the optimal transfection efficiencies of the corresponding polycations were considerably higher than that of PEI-Arg or PEI-Orn. The reason should be that introducing hydrophobic groups increased the hydrophobic interactions between carrier and cell membrane (or DNA), thereby boosting the cellular uptake. (5) For cationic ‘molecular string’ including hydrophobic groups, the only differences existed in the hydrophobic groups, such as PEI-RT3 and PEI-Arg(NO_2_), and their transfection efficiencies were dramatically different, the optimal transfection efficiency of PEI-RT3 was 2.8-fold higher than that of PEI-Arg(NO_2_). The hydrophobicity of *p*-toluene sulfonyl was stronger than that of nitro group, thus accelerating the cellular uptake of PD. (6) When the hydrophobic groups of ‘molecular string’ were the same and the difference simply lay in the category of amino acids, such as PEI-RT3 and PEI-Orn(Tos), the optimal transfection efficiency of PEI-RT3 was 4.92-fold higher than that of PEI-Orn(Tos), which was owning to the strong hydrogen-bonding interactions between guanidine group of PEI-RT3 and cell membrane (or DNA). (7) For cationic ‘molecular string’ including hydrophobic groups, such as PEI-RT3, PEI-Arg(NO_2_), and PEI-Orn(Tos), their optimal transfection efficiencies were considerably higher than that of the ‘golden standard’ PEI25k. (8) Moreover, DNA transfection was also determined in 4T1, HeLa, and MCF-7 cells, and similar results were obtained (Supplementary Figs. 20–25).

As various ‘molecular strings’ were introduced onto PEI1.8k, these polycations exhibited remarkable differences in gene transfection efficiency. This phenomenon is closely related to the physicochemical properties of the polycation/DNA complexes. The zeta potentials of polycations/DNA complexes were first measured at a mass ratio of 5/1. These polycations/DNA complexes exhibited significantly different zeta potentials at the same mass ratio (Supplementary Fig. 26). For the ‘molecular string’ containing only hydrophobic groups, such as PEI-Too and PEI-Tos, their corresponding carrier/DNA had much lower zeta potential than ED. This phenomenon resulted from the consumption of amino groups after introducing Too or Tos string, which caused a sharp decline in the positive charge density. For the cationic ‘molecular string’ containing only hydrophilic groups, such as PEI-Orn and PEI-Arg, the zeta potential of the corresponding carrier/DNA slightly changed compared with that of ED. This was because introducing these cationic ‘molecular string’ increased molecular weight of structural units and the zeta potential of carrier/DNA would not change significantly at the same mass ratio. For the cationic ‘molecular string’ containing hydrophobic groups, such as PEI-Arg(NO_2_) and PEI-RT3, their structural units had larger molecular weight than PLL-Arg. Therefore, the charge density of PEI-Arg(NO_2_)/DNA or PD was lower than that of PEI-Arg/DNA. For PEI-Orn(Tos), its ‘molecular string’ merely contained one positive charge and the zeta potential of PEI-Orn(Tos)/DNA was lower than that of PEI-Arg(NO_2_)/DNA or PD at the same mass ratio. According to gene transfection and zeta potential data, an appropriate positive surface charge is essential for efficient gene transfer.

Moreover, the particle sizes of carrier/DNA were determined (Supplementary Fig. 27). After different types of ‘molecular strings’ were introduced onto PEI1.8k, the particle sizes of all polycations/DNA were markedly smaller than those of ED at the same mass ratio. For the ‘molecular string’ containing only hydrophobic groups, such as PEI-Too and PEI-Tos, the enhanced hydrophobic interactions between carrier and DNA contributed to condensing DNA into the smaller nanoparticles. For the cationic ‘molecular string’ containing only hydrophilic groups, such as PEI-Orn, the particle size of PEI-Orn/DNA was considerably smaller than that of ED, whose reason was that the increased charge density facilitated DNA condensation into nanoparticles. In addition, PEI-Arg/DNA had a much smaller particle size than ED, because the guanidine groups of PEI-Arg tended to form strong hydrogen bonding with the phosphate groups of DNA, thus efficiently condensing DNA into smaller size. For PEI-Orn(Tos), the strong hydrophobic interactions between phenyl group of PEI-Orn(Tos) were conducive to DNA condensation. For PEI-Arg(NO_2_) and PEI-RT3, the efficient DNA condensation was due to the strong hydrogen bonding and hydrophobic interactions between polycations and DNA. Based on gene transfection and particle size results, the particle size of polycation/DNA was an important factor in gene transfer.

Transfection results revealed that PEI-RT3 possessed more efficient transfection than other polycations due to the multiple interactions (i.e., electrostatic, hydrogen bonding, and hydrophobic interactions) between PEI-RT3 and cell membrane (or DNA), which contributed to DNA loading, condensation, and endocytosis, thereby enhancing gene transfer. Therefore, PEI-RT3 was selected for further study.

Transfection efficiency and cytotoxicity are the two key factors to be considered when designing efficient gene carriers^42^. After the transfection efficiency was characterized, the cytotoxicity of carrier/DNA complexes was measured at various mass ratios. Unsurprisingly, at high mass ratios of 20/1 and 10/1, PEI25k/DNA exhibited serious cytotoxicity in B16F10 cells (Supplementary Fig. 28). However, PD showed negligible cytotoxicity even at mass ratio of 20/1. Similar results were obtained in 4T1, HeLa, and MCF-7 cells (Supplementary Figs. 29–31). On the basis of gene transfection and cytotoxicity results, PEI-RT3 could be used as powerful polycationic gene carrier with high transfection efficiency and low cytotoxicity.

Generally speaking, polycationic gene carriers have poor blood circulation time, which limits their further application in vivo^43^. In this study, mPEG-b-PLG was developed as a shielding material to augment the blood circulation of PD (Fig. 2c). The diblock copolymer mPEG-b-PLG was synthesized through the ring-opening polymerization of Glu(Bzl)-NCA initiated with mPEG-NH_2_ (Supplementary Fig. 32). ^1^H NMR spectra verified the successful preparation of Glu(Bzl)-NCA and mPEG-b-PLG with 17 structural units (Supplementary Figs. 33 and 34). mPEG-b-PLG had a narrow molecular weight distribution with a polymer distribution index of 1.08 (Supplementary Fig. 35). The effect of different amounts of mPEG-b-PLG on the transfection efficiency of PD was further determined in B16F10 cells. Surprisingly, the addition of mPEG-b-PLG dramatically promoted the gene transfection of PD at a certain range of mPEG-b-PLG/PEI-RT3 ratios (Supplementary Fig. 36); i.e., mPEG-b-PLG functioned as an enhancer of gene transfer of PD. The optimal transfection of PPD was obtained at a mass ratio of 1/5/1, which was twice that of PD in B16F10 cells (Fig. 2d and Supplementary Fig. 36). Some studies have indicated that the polyproline II (PPII)-helix conformation contributes to cellular uptake^44,45^ and circular dichroism (CD) spectra showed that mPEG-b-PLG adopted a PPII-helix conformation (Supplementary Fig. 37), which might result in enhanced gene transfection. Moreover, the optimal transfection efficiencies of PPD were 1.9, 1.9, and 2.2 times that of PD in 4T1, HeLa, and MCF-7 cells, respectively (Supplementary Figs. 38–40). The physicochemical properties of PPD were also characterized. The hydrodynamic diameter of PPD was 101.0 ± 1.2 nm by dynamic light scattering (DLS) system (Fig. 2e). In addition, the zeta potential of PPD was lower than that of PD, which was due to the shielding effect of negatively charged mPEG-b-PLG (Supplementary Fig. 41).

Nevertheless, a series of obstacles must be overcome during gene transfection. Among these, endocytosis is one of the main obstacles. DNA was labelled with Cy5 and flow cytometry assay was conducted in B16F10, 4T1, HeLa, and MCF-7 cells, to determine the cellular uptake efficiency of PPD. Unsurprisingly, the uptake efficiency of PD was significantly higher than that of ED in B16F10 cells (Fig. 2f). This phenomenon occurred because of the multiple interactions (electrostatic, hydrogen bonding, and hydrophobic interactions) between PEI-RT3 and cell membrane (or DNA) after the introduction of the RT string, which boosted the cellular uptake. Moreover, PPD exhibited more efficient endocytosis than PD, resulting from the PPII-helix conformation of mPEG-b-PLG. Similar endocytosis results were obtained in 4T1, HeLa, and MCF-7 cells (Supplementary Figs. 42–44). Fluorescent images further revealed that PD and PPD had considerably higher endocytosis than ED (Fig. 2g and Supplementary Figs. 45–47). Nevertheless, the zeta potential of PD and PPD was lower than that of ED (Supplementary Fig. 26), which was not conducive to the endocytosis of PD or PPD. In addition, the particle sizes of PD and PPD were considerably smaller than those of ED (Supplementary Fig. 27). A small particle size is detrimental to the cellular uptake to some extent^46^. Therefore, the low zeta potential and small size of PD and PPD counted against the cellular uptake. However, the endocytosis results were widely divergent, suggesting that multiple interactions and PPII-helix confirmation played a vital role in promoting the internalization efficiency of PPD.

After entry into cells, the successful endo/lysosomal escape of nanoparticles is crucial to efficient gene transfection^47,48^. Herein, the endo/lysosomal escape behavior of PPD was observed using confocal laser scanning microscopy (CLSM) in four cell lines (B16F10, 4T1, HeLa, and MCF-7 cells). Nuclei were stained with 4’,6-diamidino-2-phenylindole (DAPI). Endo/lysosome and DNA were labelled with Lysotracker Green (green) and Cy5 (red), respectively. Yellow fluorescent dots indicated the overlap of endo/lysosome (green) and carrier/DNA complexes (red), and reflected the colocation of endo/lysosome and complexes. Images were taken at different time points (1, 4, and 7 h). The yellow fluorescent dots were clearly observed at 1 h for PD and PPD groups in B16F10 cells, implying that PD or PPD entered the endo/lysosome (Fig. 2h). In addition, more yellow fluorescent dots were observed at 4 h, indicating that the number of PD or PPD that had entered endo/lysosome was increased. Remarkably, a sharp decline in the yellow fluorescent dots for PD and PPD group was observed from 4 to 7 h. This was because the fluorescence intensity of Lysotracker Green (green) channel for PD and PPD was significantly decreased at 7 h, which resulted from the rupture of endo/lysosome. Thus, the significant reduction of yellow fluorescent dots from 4 to 7 h confirmed that PD or PPD could escape from endo/lysosome. By comparison, only a few yellow fluorescent dots were observed in ED group throughout the process. Therefore, the endo/lysosomal escape ability of ED, PD, and PPD could not be directly compared using confocal fluorescent images. To evaluate the endo/lysosomal escape ability, correlation coefficient *R*-value from confocal fluorescent images was defined as colocalization degree of carrier/DNA and endo/lysosome. In other words, the greater *R*-value was, the more prominent colocalization degree was. Therefore, the endo/lysosomal escape ability of ED, PD, and PPD could be compared based on the extent of change in the *R*-value. The *R*-values of ED, PD, and PPD group first increased and then decreased in B16F10 cells (Supplementary Fig. 48). The reduced *R*-value from 4 to 7 h indicated that ED, PD, and PPD could escape from endo/lysosome. In particular, a more significant reduction in the *R*-value suggested that PD and PPD possessed better endo/lysosomal escape ability than ED. Moreover, similar results were obtained in 4T1, HeLa, and MCF-7 cells (Supplementary Figs. 49 and 54).

According to the above experimental results, introducing multiple interactions and PPII-helix conformation could synergistically promote endocytosis efficiency and endo/lysosomal escape, which eventually contributed to gene transfer in various cell lines. It was worth mentioning that PPD exhibited good biocompatibility (Supplementary Fig. 55) and this polymeric gene delivery system showed highly efficient RNA silencing (Supplementary Fig. 56). In light of DNA transfection and RNA-silencing results, this polymeric gene delivery system functioned as a versatile vehicle that could transport both pDNA and small interfering RNA (siRNA).

These results indicated that PPD possessed an efficient gene transfer in vitro. Nevertheless, the blood circulation time needed to be determined for the in vivo application of a polymeric gene delivery system. Herein, a pharmacokinetic study was performed to evaluate the blood circulation time of PPD in vivo. Carrier/Cy5-DNA nanoparticles were injected intravenously and the concentrations of Cy5-DNA were detected at different time points (0.2, 0.5, 1, 3, 5, 7.5, 12, and 24 h). The Cy5-DNA concentration of PPD group was considerably higher than that of the other groups (Supplementary Fig. 57) at the same time point. The half-life of circulatory DNA under individual conditions was calculated according to the pharmacokinetic assay (Supplementary Table 3). The half-life of circulatory DNA in PPD group was 0.71 h, which was remarkably longer than that of the other groups. Moreover, the area under the curve (AUC_0-t_) of PPD was 8.97 μg mL^−1^ h, which was also much higher than that of other groups. These results suggested that the shielding function of mPEG-b-PLG could significantly enhance the circulatory half-life of PD in vivo.

The biodistribution of carrier/DNA complexes in the main organs and tumour tissues was also evaluated by ex vivo fluorescence imaging after intravenous administration into B16F10 or 4T1 tumour-bearing mice. DNA, ED, and PD mainly accumulated in the kidney and liver (Supplementary Figs. 58 and 59) due to their poor circulatory half-life and easy clearance by the kidney and liver^49^. By contrast, PPD showed good accumulation in tumour tissue. This was mainly because PPD exhibited prolonged blood circulation time in vivo and could be passively targeted to tumour tissue through the enhanced permeability and retention (EPR) effect^50,51^. The distribution of PPD complexes within tumours was further characterized. PPD complexes were found to effectively accumulate in tumour tissue and penetrate within tumour (Supplementary Fig. 60a, b). Furthermore, to explore the gene transfection effect of carrier/DNA in vivo, the plasmid-encoding red fluorescent protein (RFP) was used as reporter gene and the transfection efficiency of carrier/DNA was measured by ex vivo fluorescence imaging after intravenous injection into B16F10 or 4T1 tumour-bearing mice. For the PPD group, considerably higher fluorescence intensity was obtained in tumour tissues than that in the main organs (heart, liver, spleen, lung, and kidney) (Supplementary Figs. 61 and 62), which might be because PPD effectively accumulated in tumour tissue through the EPR effect and was internalized by tumour cells for efficient gene transfection. Moreover, the expressed RFP was efficiently penetrated within the tumour tissue (Supplementary Fig. 63a, b). Compared with that for the DNA, ED, and PD groups, the RFP expression of tumour tissues was significantly higher for the PPD group, which was due to the prolonged circulatory half-time, effective accumulation in tumour tissue, and efficient endocytosis by tumour cells.

### Anti-tumour effect by PPD combined with Zeb

**Fig. 3:**
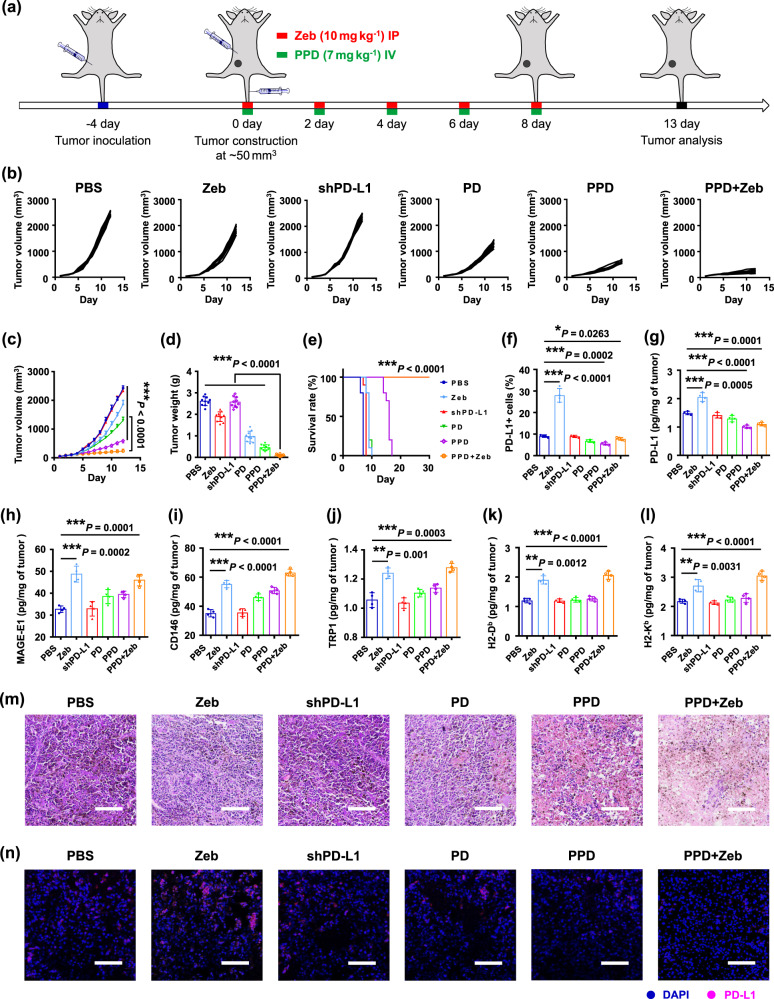
PPD combined with Zeb treatment for anti-tumour effect. **a** Schematic of PPD combined with Zeb therapy in B16F10 tumour-bearing mice. PPD was intravenously injected (denoted as IV) into B16F10 tumour-bearing mice, and Zeb was paratumourally injected (denoted as IP). **b** Individual and **c** average tumour growth kinetics in B16F10 tumour-bearing mice receiving different treatments. **d** Average tumour weight in B16F10 tumour-bearing mice receiving different treatments. **e** Survival time of tumour-bearing mice receiving different treatments as indicated. When the tumour volume of mice reached 1000 mm^3^, the mice were considered dead. **f** Percentages of PD-L1^+^ cells in tumour tissues of B16F10 tumour-bearing mice receiving different treatments. **g** Levels of PD-L1 protein expression in tumour tissues of B16F10 tumour-bearing mice receiving different treatments. Levels of tumour-associated antigen including **h** MAGE-E1, **i** CD146, and **j** TRP-1 in tumour tissues of B16F10 tumour-bearing mice receiving different treatments. Levels of MHC I molecules including **k** H2-D^b^ and **l** H2-K^b^ in tumour tissues of B16F10 tumour-bearing BABL/c mice receiving different treatments. **m** Representative H&E-stained images of tumour tissues of B16F10 tumour-bearing mice receiving different treatments, scale bar: 200 μm. **n** Representative fluorescence images of PD-L1 expression in tumour tissues of B16F10 tumour-bearing mice receiving different treatments, scale bar: 50 μm. Data in **b**–**l** are presented as mean ± SD, *n* = 10 biologically independent samples in **b**–**e**, *n* = 4 biologically independent samples in **f**–**l**, *P*-values are calculated by the two-tailed Student’s *t*-test in **b**–**l** as indicated in the figures. *P < 0.05, **P < 0.01, and ***P < 0.001. A representative image of three independent samples from each group is shown in **m** and **n**.

Owing to its highly efficient gene delivery in vitro and in vivo, PPD has been used as an anti-tumour therapy. The interaction between PD-1 on T cells and its ligand PD-L1 on tumour cells boosts T-cell exhaustion^52–54^. Therefore, weakening or eliminating the interaction between PD-1 and PD-L1 can reverse T-cell exhaustion and enhance T-cell-mediated tumour cell killing. Moreover, the recent studies reported that epigenetic therapy such as DNMTi not only upregulated the expression of tumour-associated antigens, but also increased MHC I expression via CTA (Supplementary Figs. 64, 65, 66a, b, 67a, b, and 68a, b), which ultimately mounted the visibility of tumour cells to the adaptive immune system^23,24^. Herein, the plasmid-encoding shPD-L1 (denoted as shPD-L1) was selected as the therapeutic gene, and PPD was intravenously administered into B16F10 tumour-bearing mice. Meanwhile, Zeb was injected paratumourally (Fig. 3a). As shown in Fig. 3b, c, different formulations exhibited various anti-tumour effects. The most significant tumour growth was observed in the phosphate buffer solution (PBS) and shPD-L1 groups. Tumour growth was slightly inhibited in the Zeb group. This was mainly because Zeb enhanced the expression of tumour-associated antigens (MAGE-E1, TRP-1, and CD146) in B16F10 tumour (Fig. 3h–j), induced DC maturation, and upregulated MHC I (H2-D^b^ and H2-K^b^) molecules after administration (Fig. 3k, l), which strengthened the antigen presentation ability of DCs and the recognition of tumour cells by T cells. However, DNMTi also upregulated the PD-L1 expression on tumour cells (Fig. 3f, g, n, Supplementary Figs. 66c, 68c, and 69), consequently compromising the killing function of T cells. Compared with the PD group, the PPD group exhibited a more efficient anti-tumour effect. In addition, mPEG-b-PLG/PEI-RT3/control shRNA plasmid (abbreviated as PPD-Control) inhibited hardly the tumour growth of B16F10 tumour-bearing mice (Supplementary Fig. 70). The reason should be that PPD could effectively accumulate in tumour tissues, efficiently mediate the endocytosis of shPD-L1 by tumour cells, and significantly downregulate the PD-L1 expression on tumour cells (Supplementary Fig. 71), all of which ultimately boosted T-cell-mediated tumour cell killing. Moreover, PPD plus Zeb showed more efficient anti-tumour effect than PPD or Zeb alone. The tumour weight results were consistent with tumour volume data. (Fig. 3d). The reason should be that combining these two relieved T cells immunosuppression and enhanced the recognition of tumour cells by T cells, thus synergistically promoting anti-tumour immunity.

Furthermore, to demonstrate the role of MHC I molecules in Zeb plus PPD treatment, the siRNA silencing MHC I molecules (abbreviated as siH2-K^b^) was designed. As shown in Supplementary Fig. 72, compared with the control (untreated) group, the relative H2-K^b^ mRNA expression in mPEG-b-PLG/PEI-RT3/siH2-K^b^ (denoted as PP/siH2-K^b^) group was significantly downregulated in B16F10 cells. Moreover, the anti-tumour effect of PPD plus Zeb accompany with MHC I depletion was studied in B16F10 tumour-bearing mice. PP/siH2-K^b^ complexes were injected intratumourally along with PPD plus Zeb treatment. The anti-tumour efficacy of PPD plus Zeb was weakened after MHC I depletion in B16F10 tumour model (Supplementary Fig. 73b, c, e). These results suggested that depletion of certain MHC I molecules provided the significant yet limited protection of tumour growth against Zeb plus PPD treatment.

Hematoxylin-eosin (H&E) staining images indicated that PPD plus Zeb exhibited the most significant tumour tissue destruction (Fig. 3m). Moreover, the body weight of mice in PPD plus Zeb group remained stable during the treatment (Supplementary Fig. 74). The results of liver function and renal function detection in serum revealed that the mice of PPD plus Zeb group showed no significant difference with age- and body-weight-matched healthy mice (Supplementary Figs. 75 and 76), indicating that PPD plus Zeb possessed good biocompatibility in vivo. Furthermore, the survival time of B16F10 tumour-bearing mice was studied after treatment (Fig. 3e). Compared with the PPD and Zeb groups alone, the mice in PPD plus Zeb group had a much longer survival time and did not die even after 30 days, suggesting that PPD plus Zeb combination therapy could greatly prolong the survival period of tumour-bearing mice.

Recently, it has been reported that PD-1 or PD-L1 targeted antibody therapies may cause severe side effects such as irAEs^35,36^. Among them, Th17 cells were upregulated in inflammatory organ tissues of autoimmune diseases, suggesting the percentage of Th17 cells could serve as a parameter to evaluate the irAEs of immune checkpoint inhibitor-mediated immunotherapy^55,56^. Herein, the anti-tumour efficacy and irAEs of PPD plus Zeb versus aPD-L1 plus Zeb were evaluated. Compared to PPD group, aPD-L1 hardly inhibited B16F10 tumour growth. Moreover, PPD plus Zeb group exhibited a more efficient anti-tumour effect than aPD-L1 plus Zeb group (Supplementary Fig. 77b, c). Furthermore, the percentage of Th17 (CD4^+^ IL17^+^) cells in the spleens of tumour-bearing mice was detected at the end of treatment. It was found that aPD-L1 and aPD-L1 plus Zeb group had considerably higher percentage of Th17 cells than the PBS group, which suggested the possibility of irAEs when the systematic administration of aPD-L1 monoclonal antibody in cancer therapy (Supplementary Fig. 77e). However, the percentages of Th17 cells in PPD and PPD plus Zeb groups were similar to those in PBS group.

Furthermore, compared with other DNMTi such as azacytidine (AC) and deoxyazacytidine (DAC), Zeb showed better biocompatibility in B16F10 and 4T1 cells (Supplementary Fig. 78a, b). Moreover, the anti-tumour effects of PPD plus AC or DAC were evaluated in the B16F10 tumour model. PPD plus AC or DAC showed a similar anti-tumour efficacy to PPD plus Zeb (Supplementary Fig. 79b, c). Moreover, when the dosage of AC or DAC was half that of Zeb, the body weights in PPD plus AC or PPD plus DAC groups were significantly reduced during treatment, whereas PPD plus Zeb group exhibited the negligible changes, indicating that Zeb had better in vivo biological safety than AC and DAC (Supplementary Fig. 79d).

### PPD combined with Zeb induced significant anti-tumour immune response

On the basis of the above anti-tumour results, PPD plus Zeb combination therapy exerted a remarkable anti-tumour effect. We speculated that the main reasons for this included two aspects. One was that Zeb treatment generated tumour-associated antigens and MHC I molecules, thus inducing DC maturation and enhancing the capability of DCs to present antigens to T cells. The other was that PPD treatment relieved the immunosuppression of T cells and augmented the CD8^+^ T-cell effector function.

**Fig. 4:**
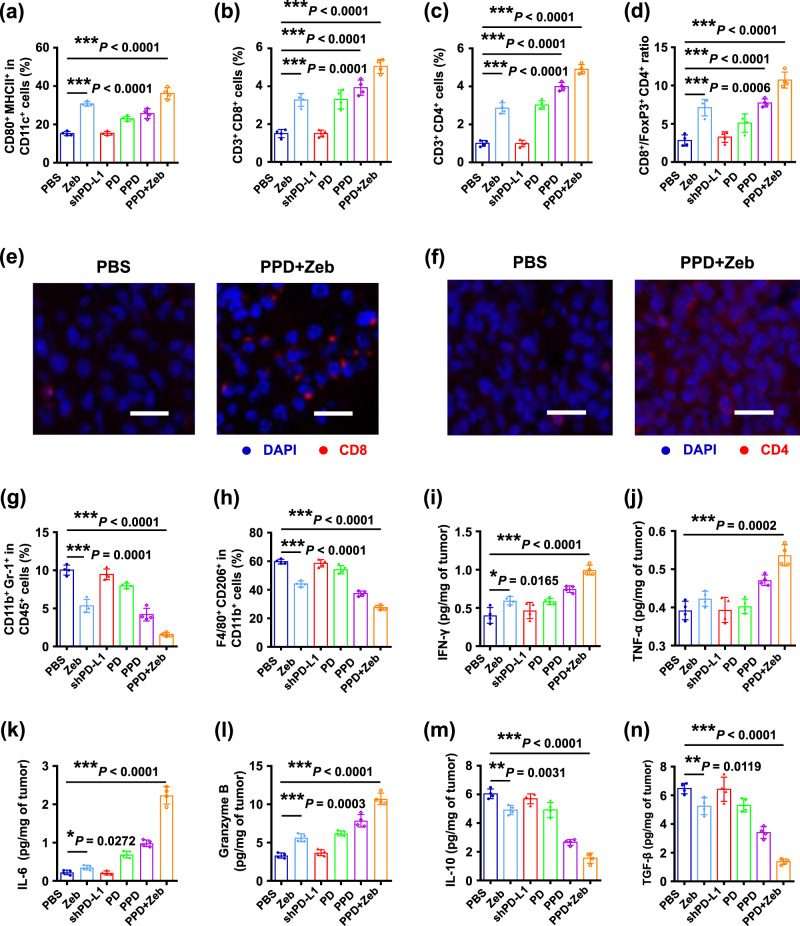
PPD combined with zebularine induced significant anti-tumour immune response. **a** Percentages of CD80^+^ MHC II^+^ dendritic cells gating on CD11c^+^ cells in lymph nodes of B16F10 tumour-bearing mice after different treatments. **b** Percentages of CD3^+^ CD8^+^ T cells gating on CD45^+^ cells in tumour of B16F10 tumour-bearing mice receiving different treatments. **c** Percentages of CD3^+^ CD4^+^ T cells gating on CD45^+^ cells in tumour of B16F10 tumour-bearing mice after treatment. **d** Ratios of CD8^+^T/Foxp3^+^ CD4^+^ T in tumours of B16F10 tumour-bearing mice receiving different treatments. **e** Representative immunofluorescence images of CD8^+^ T cells in tumours of B16F10 tumour-bearing mice after treatment, scale bar: 20 μm. **f** Representative immunofluorescence images of CD4^+^ T cells in tumours of B16F10 tumour-bearing mice after treatment, scale bar: 20 μm. **g** Percentages of CD11b^+^ Gr-1^+^ cells gating on CD45^+^ cells in tumours of B16F10 tumour-bearing mice receiving different treatments. **h** Percentages of CD206^+^F4/80^+^ cells gating CD11b^+^ cells in tumours of B16F10 tumour-bearing mice receiving different treatments. Levels of cytokine including **i** IFN-γ, **j** TNF-α, **k** IL-6, **l** Granzyme B, **m** IL-10, and **n** TGF-β in tumours of B16F10 tumour-bearing mice after treatment. Data in **a**–**d** and **g**–**n** are presented as mean ± SD, *n* = 4 biologically independent samples in **a**–**d** and **g**–**n**, *P*-values are calculated by the two-tailed Student’s *t*-test in **a**–**d** and **g**–**n** as indicated in the figures. *P < 0.05, **P < 0.01, and ***P < 0.001. A representative image of three independent samples from each group is shown in **e** and **f**.

To verify this conjecture, the immune cells in lymph nodes and tumour tissues were analyzed by flow cytometry after treatment. Mice receiving various formulations exhibited significantly different percentages of mature DCs (Fig. 4a and Supplementary Fig. 80). For PBS and shPD-L1 groups, the percentage of mature DCs (CD80^+^ MHC II^+^) was ~15%. Zeb group showed a higher percentage of DC maturation than PBS and shPD-L1 groups at 30%, which was twice that of PBS or shPD-L1 group. The reason should be that the tumour-associated antigens (MAGE-E1, CD146, and TRP-1) generated by Zeb induced DC maturation. In addition, the percentage of mature DCs increased in the lymph nodes in PPD group compared with that in the PBS or shPD-L1 group. This was because PD-L1 downregulation induced tumour cell death to a certain extent and encouraged the production of tumour-associated antigen for DC maturation. Moreover, the highest percentage of mature DCs in the spleen was observed in PPD plus Zeb group at almost 40%. These results indicated that PPD plus Zeb could effectively induce DC maturation.

To further verify that PPD plus Zeb can elicit in vivo anti-tumour immune response, CD8^+^ T and CD4^+^ T cells in the tumour tissues of B16F10 tumour-bearing mice were analyzed after treatment. The higher percentage of CD3^+^ CD8^+^ T cells in tumour tissues of Zeb and PPD groups was obtained compared with that in PBS or shPD-L1 groups (Fig. 4b and Supplementary Fig. 81). This finding indirectly indicated that Zeb or PPD had a moderate anti-tumour efficacy. Moreover, PPD plus Zeb group exhibited the most significant intratumoural infiltration of CD3^+^CD8^+^ T cells. Similarly, the immunofluorescence images of tumour tissues also showed that PPD plus Zeb group possessed notable CD8^+^ T-cell infiltration (Fig. 4e). Similar results were obtained for intratumoural CD4^+^ T-cell infiltration (Figs. 4c, f and Supplementary Fig. 82). Furthermore, the ratio of CD8^+^ T/Tregs (CD8^+^/FoxP3^+^CD4^+^) in PPD + Zeb group was considerably higher than that of PBS group (Fig. 4d) and the proportions of immunosuppressive cells including MDSCs (CD11b^+^Gr-1^+^in CD45^+^) and M2 cells (CD206^+^F4/80^+^ in CD11b^+^) were significantly decreased after PPD plus Zeb combination therapy (Fig. 4g, h and Supplementary Figs. 83 and 84). Remarkably, PPD plus Zeb combination therapy upregulated the levels of proinflammatory cytokines (interleukin (IL)-6, interferon (IFN)-γ, tumour necrosis factor (TNF)-α, and granzyme B (GzmB)) and downregulated the levels of immunosuppressive cytokines (IL-10 and transforming growth factor (TGF)-β) in tumour tissues (Fig. 4i–n). Moreover, to uncover major target genes, reverse-transcription-quantitative real-time PCR (RT-qPCR) assay of B16F10 tumour model was carried out after PPD plus Zeb treatment and the heatmap of mRNA expression of relevant genes was provided. As shown in Supplementary Fig 85, these CTA genes including *SSX-9*, *MAGE-A3*, *SSX-b2*, *Taf7l*, *Ctcfl*, and *Lipa* were significantly upregulated in PPD plus Zeb treatment compared with the PBS group. In addition, PPD plus Zeb treatment also increased the levels of MHC I molecules (H2-D^b^ and H2-K^b^) and immunoactivated cytokines including IFN-γ, TNF-α, GzmB, IL-6, and IL-1β. Moreover, PD-L1 and immunosuppressive cytokines including IL-10 and TGF-β were significantly downregulated after PPD plus Zeb treatment. These results suggested that PPD plus Zeb combination therapy could upregulate the levels of some CTA genes (*SSX-9*, *MAGE-A3*, *SSX-b2*, *Taf7l*, *Ctcfl*, and *Lipa*) accompanied by enhancement of MHC I (H2-D^b^ and H2-K^b^) and downregulate the PD-L1 expression in tumour tissue, resulting in an increase in immunoactivated cytokines and a decrease in immunosuppressive cytokines, all of which were beneficial to inhibit tumour growth. Based on the analysis of immunocytes, CTAs, and cytokines, PPD plus Zeb combination therapy could transform B16F10 tumours from an immunosuppressive phenotype into an immunoactivated phenotype.

### PPD combined with Zeb inhibited tumour relapse and initiated tumour-specific immune memory effect

**Fig. 5:**
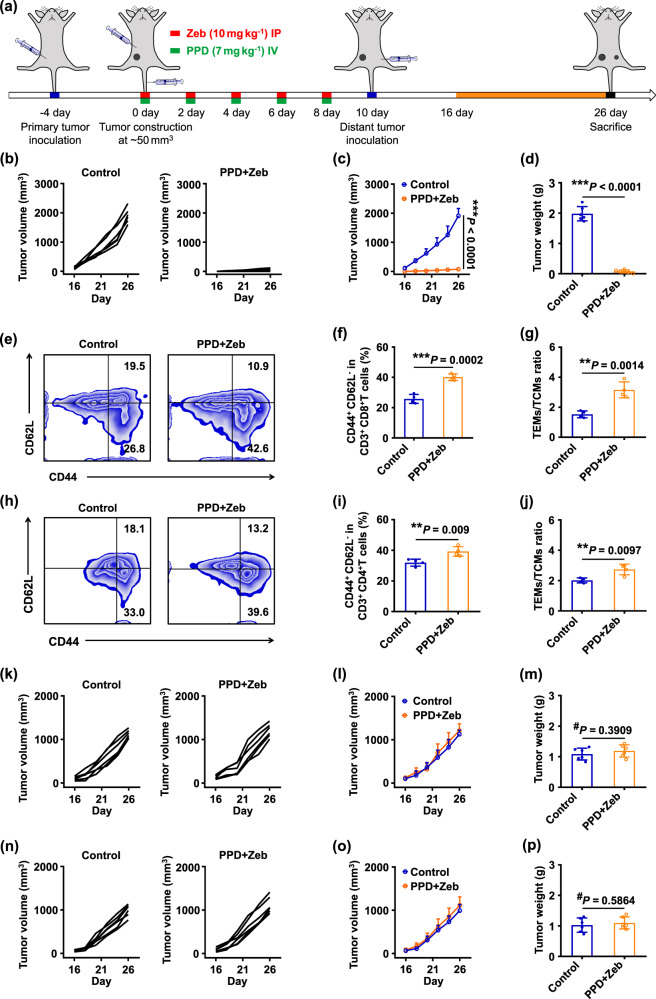
PPD combined with Zeb inhibited tumour relapse and initiated tumour-specific immune memory effect. **a** Therapeutic schedule for PPD plus Zeb-mediated inhibition of tumour relapse. **b** Individual and **c** average tumour growth kinetics in distant B16F10 tumour-bearing mice receiving PPD plus Zeb treatment. Age- and body-weight-matched healthy mice were used as control. **d** Average tumour weight of distant B16F10 tumour-bearing mice receiving PPD plus Zeb treatment. **e** Representative flow cytometric analysis of TEM (CD44^+^CD62L^−^) and TCM (CD44^+^ CD62L^+^) cells gating on CD8^+^ T cells in spleen of B16F10 tumour-bearing mice at the end of treatment. Relative quantification of **f** TEM (CD44^+^CD62L^−^) and **g** ratio of TEMs/TCMs gating on CD8^+^ T cells in the spleen of B16F10 tumour-bearing mice at the end of treatment. **h** Representative flow cytometric analysis of TEM (CD44^+^CD62L^−^) and TCM (CD44^+^CD62L^+^) cells gating on CD4^+^ T cells in the spleen of B16F10 tumour-bearing mice at the end of treatment. Relative quantification of **i** TEM and **j** ratio of TEMs/TCMs gating on CD4^+^ T cells in the spleen of B16F10 tumour-bearing mice at the end of treatment. **k** Individual and **l** average tumour growth kinetics in distant MC38 tumour-bearing mice receiving PPD plus Zeb treatment. Age- and body-weight-matched healthy mice were used as control. **m** Average tumour weight of distant MC38 tumour-bearing mice receiving PPD plus Zeb treatment. **n** Individual and **o** average tumour growth kinetics in distant LLC tumour-bearing mice receiving PPD plus Zeb treatment. Age- and body-weight-matched healthy mice were used as control. **p** Average tumour weight of distant LLC tumour-bearing mice receiving PPD plus Zeb treatment. Data in **c**, **d**, **f**, **g**, **i**, **j**, **l**, **m**, **o**, and **p** are presented as mean ± SD, *n* = 6 biologically independent samples in **c**, **d**, **l**, **m**, **o**, and **p**, *n* = 4 biologically independent samples in **f**, **g**, **i**, and **j**. *P*-values are calculated by the two-tailed Student’s *t*-test in **c**, **d**, **f**, **g**, **i**, **j**, **l**, **m**, **o**, and **p** as indicated in the figures. ^#^*P* > 0.05, *P < 0.05, **P < 0.01, and ***P < 0.001.

Relapse and metastasis are the main causes of cancer-related mortality^57,58^. Encouraged by the strongly stimulating in vivo anti-tumour immune response of PPD plus Zeb combination therapy, we wonder whether PPD plus Zeb combination therapy can prevent tumour relapse. Tumour re-challenge experiment was conducted to simulate the relapse (Fig. 5a). B16F10 tumour-bearing mice after PPD plus Zeb treatment were re-implanted with B16F10 tumour. We found that PPD plus Zeb combination therapy significantly inhibited tumour relapse (Fig. 5b, c). However, remarkable tumour growth was observed when B16F10 cells were implanted into age- and body-weight-matched healthy mice. Similar results were obtained in the tumour weight experiments (Fig. 5d). In addition, the body weight of PPD plus Zeb group did not show significant changes throughout the process (Supplementary Fig. 86). Taken together, these results indicated that PPD plus Zeb combination therapy could effectively inhibit tumour relapse.

To clarify the mechanism by which PPD plus Zeb inhibited tumour relapse, the proportions of memory T cells, including effector memory T cells (denoted as TEMs) and central memory T cells (denoted as TCMs) of CD8^+^ T and CD4^+^ T cells in the spleen were analyzed at the end of treatment. In CD3^+^ CD8^+^ T subtypes, the proportion of effector memory T cells (CD44^+^CD62^−^) in PPD plus Zeb group was considerably higher than that in the control group (Fig. 5e, f and Supplementary Fig. 87). In addition, the quantity ratio of TEMs/TCMs in PPD plus Zeb group was significantly greater than that in the control group (Fig. 5g). TEMs served as the immediate antigen-primed cells that entered peripheral tissues and mediated inflammatory reactions or cytotoxicity, whereas TCMs could not exert cytotoxic function until TCMs effectively differentiated into TEMs^59^. Moreover, TEMs/TCMs in CD3^+^CD4^+^T subtypes exhibited similar results (Fig. 5h–j). On the basis of these data, PPD plus Zeb could initiate efficient immune memory response by generating high levels of TEMs, which remarkably inhibited tumour relapse. However, once B16F10 tumour-bearing mice receiving PPD plus Zeb combination treatment were re-implanted with MC38 (murine colon carcinoma) or LLC (Lewis lung cancer) tumour, PPD plus Zeb-treated animals showed a negligible difference compared with the control group in tumour volume and tumour weight (Fig. 5k–p). Moreover, the body weight of PPD plus Zeb group did not show significant changes throughout the process (Supplementary Figs. 88 and 89). This phenomenon demonstrated that tumour growth could not be effectively inhibited when PPD plus Zeb-treated B16F10 tumour-bearing mice were re-implanted with MC38 or LLC tumour.

Furthermore, when 4T1 tumour-bearing mice receiving PPD plus Zeb treatment were re-implanted with 4T1 tumour, PPD plus Zeb-treated animals could significantly inhibit tumour relapse by inducing an efficient immune memory response (Supplementary Fig. 90a–j). Nevertheless, when 4T1 tumour-bearing mice after PPD plus Zeb treatment were re-implanted with CT26 tumour, the tumour growth curves and tumour weight of distant tumours showed negligible differences between PPD plus Zeb and control groups (Supplementary Fig. 90k–m). In addition, the body weight of PPD plus Zeb group showed negligible changes during the process (Supplementary Figs. 91 and 92). These results verified that PPD plus Zeb initiated a tumour-specific immune memory response and suggested its potential for personalized immunotherapy.

### PPD combined with Zeb prevented tumour metastasis

**Fig. 6:**
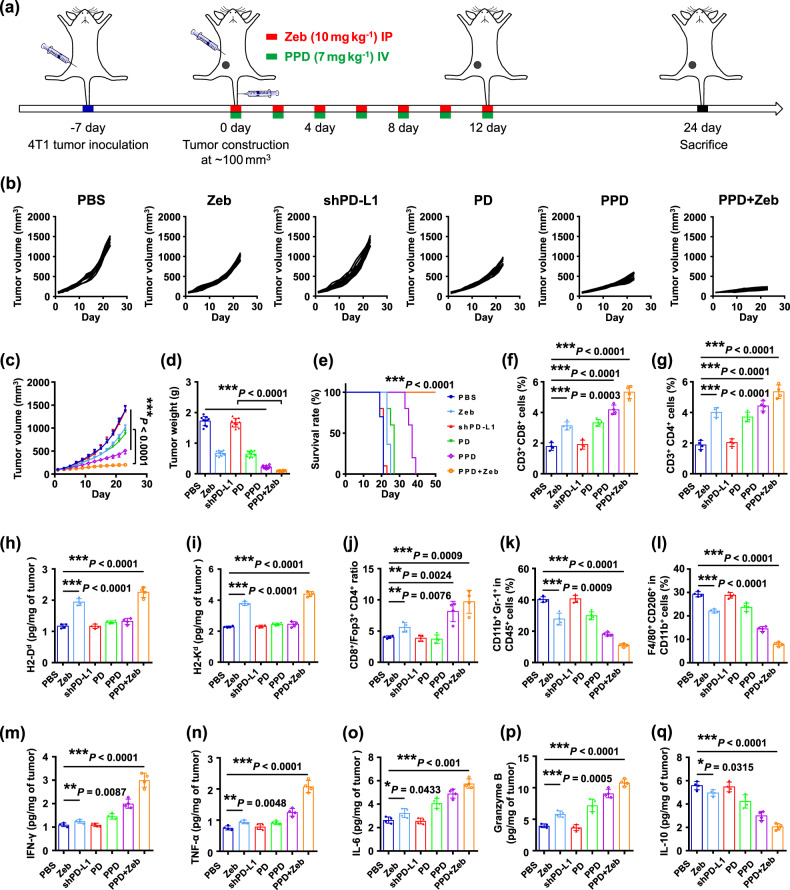
PPD combined with Zeb induced systemic anti-tumour immune response and inhibited 4T1 tumour growth. **a** Therapeutic schedule for PPD plus Zeb-mediated inhibition of 4T1 tumour growth. **b** Individual and **c** average tumour growth kinetics in 4T1 tumour-bearing mice receiving different treatments. **d** Average tumour weight in 4T1 tumour-bearing mice receiving different treatments. **e** Survival time of 4T1 tumour-bearing mice receiving different treatments as indicated. When the tumour volume of tumour-bearing mice arrived at 1000 mm^3^, the mice was defined as dead. **f** Percentages of CD3^+^CD8^+^T cells gating on CD45^+^ cells in tumours of 4T1 tumour-bearing mice receiving different treatments. **g** Percentages of CD3^+^CD4^+^ T cells gating on CD45^+^ cells in tumours of 4T1 tumour-bearing mice receiving different treatments. Levels of MHC I molecules including **h** H2-D^d^ and **i** H2-K^d^ in tumours of 4T1 tumour-bearing mice receiving different treatments. **j** Ratios of CD8^+^T/Foxp3^+^CD4^+^T in tumours of 4T1 tumour-bearing mice receiving different treatments. **k** Relative quantification of CD11b^+^Gr-1^+^ cells gating on CD45^+^ cells in tumours of 4T1 tumour-bearing mice receiving different treatments. **l** Relative quantification of CD206^+^F4/80^+^ cells gating on CD11b^+^ cells in tumours of 4T1 tumour-bearing mice receiving different treatments. Levels of cytokine including **m** IFN-γ, **n** TNF-α, **o** Granzyme B, **p** IL-10, and **q** TGF-β in tumours of 4T1 tumour-bearing mice receiving different treatments. Data in **c**–**q** are presented as mean ± SD, *n* = 10 biologically independent samples in **c**–**e**, *n* = 4 biologically independent samples in **f**–**q**. *P*-values are calculated by the two-tailed Student’s *t*-test in **c**–**q** as indicated in the figures. *P < 0.05, **P < 0.01, and ***P < 0.001.

Based on the above results, PPD plus Zeb combination therapy could induce a systemic anti-tumour immune response and evoke tumour-specific immune memory effect, which efficiently inhibited tumour growth and relapse. Moreover, we wondered whether PPD plus Zeb combination therapy could effectively inhibit the metastasis of highly invasive tumours. Triple-negative breast cancer (TNBC) is characterized by estrogen receptor-, progesterone receptor-, and HER2-negative, which is extremely aggressive with a high risk of metastasis^60,61^. Herein, an orthotopic 4T1 breast cancer model was established as a TNBC model^62,63^ for exploring tumour growth and metastasis after PPD plus Zeb combination therapy (Fig. 6a). Compared with PBS and PPD-Control groups, PPD group showed remarkable inhibition of 4T1 tumour growth (Fig. 6b–d and Supplementary Fig. 93b–d). Moreover, compared with the other groups, PPD plus Zeb group exhibited the most significant tumour inhibition effect (Fig. 6b–d) and the H&E staining results showed that PPD plus Zeb combination therapy caused the most serious destruction of tumour tissue (Supplementary Fig. 94). Moreover, no significant changes in body weight were observed during treatment (Supplementary Figs. 93e and 95). In addition, the liver and renal function of 4T1 tumour-bearing mice in PPD plus Zeb group were normal after treatment (Supplementary Figs. 96 and 97). Furthermore, according to the recorded survival time of tumour-bearing mice, PPD plus Zeb-treated animals were still alive even on the 30th day, whereas the mice of other groups had all died (Fig. 6e). This finding revealed that PPD plus Zeb combination therapy greatly extended the survival period of 4T1 tumour-bearing mice.

Immune cells in lymph nodes and tumour tissues were analyzed. PPD plus Zeb group had considerably higher proportion of mature DCs than the other groups in lymph nodes (Supplementary Figs. 98 and 99). Moreover, the CTLs infiltrating in 4T1 tumour-bearing mice were further explored after treatment. Remarkably, PPD plus Zeb combination therapy enormously enhanced the infiltration of CD8^+^T and CD4^+^T cells (Fig. 6f, g and Supplementary Figs. 100 and 101), which indirectly indicated that PPD plus Zeb combination therapy could initiate an efficient anti-tumour immune response. On the one hand, Zeb could contribute to DC maturation and MHC I molecules expression to enhance antigen presentation ability of DCs, which was conducive to the recognition of tumour cells by T cells (Fig. 6h, i). On the other hand, PPD downregulated the PD-L1 expression on tumour cells (Supplementary Figs. 102–104), thus relieving the immunosuppression of T cells by checkpoint blockade and boosting T-cell-mediated tumour killing.

Moreover, immunosuppressive cells such as Tregs, MDSCs, and M2 cells in tumour tissues were further analyzed (Fig. 6j–l and Supplementary Figs. 105 and 106). Compared with the other groups, PPD plus Zeb group had the smallest proportion of Tregs, MDSCs, and M2 cells. In addition, proinflammatory cytokines including IL-6, GzmB, IFN-γ, and TNF-α were upregulated whereas anti-inflammatory cytokines such as IL-10 and TGF-β were downregulated after PPD plus Zeb combination treatment (Fig. 6m–q and Supplementary Fig. 107). According to the above results, PPD plus Zeb combination therapy could elicit a significant adaptive immune response in 4T1 tumour-bearing mice and evoke efficient anti-tumour immunity.

**Fig. 7:**
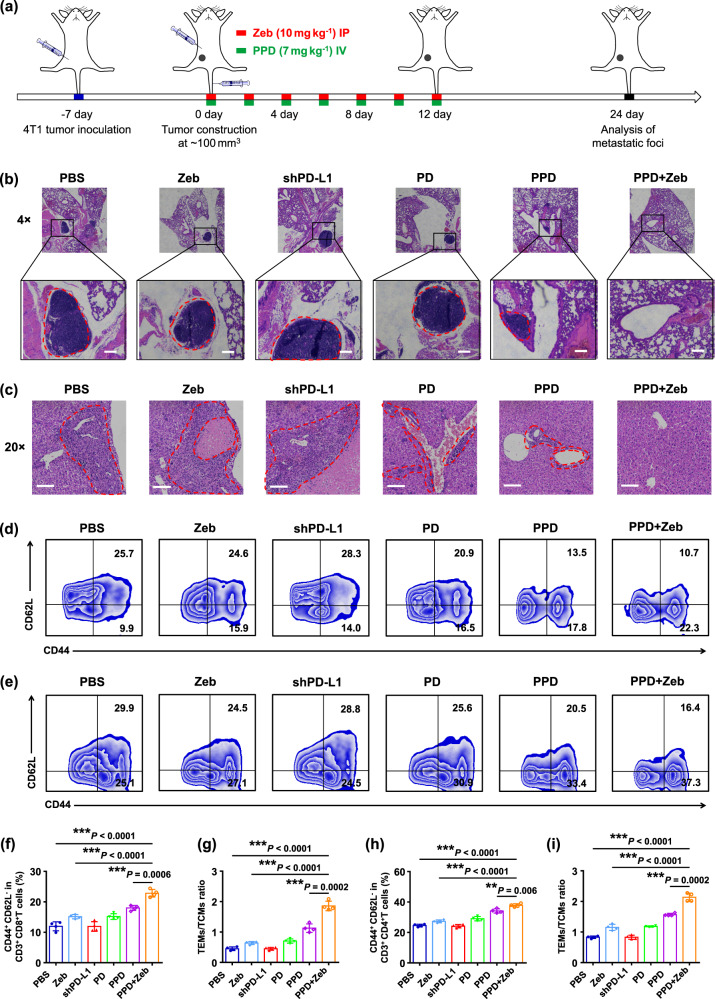
PPD combined with Zeb inhibited 4T1 tumour metastasis. **a** Therapeutic schedule for PPD plus Zeb-mediated inhibition of 4T1 tumour metastasis. **b** Representative H&E-stained lung slice images at the end of treatment, the red dashed area indicated tumour metastasis, scale bar: 100 μm. **c** Representative H&E-stained liver slice images at the end of treatment, the red dashed area indicated tumour metastasis, scale bar: 100 μm. **d** Representative flow cytometric analysis of TEM (CD44^+^CD62L^−^) and TCM (CD44^+^CD62L^+^) cells gating on CD8^+^ T cells in spleen of 4T1 tumour-bearing mice at the end of the treatment. **e** Representative flow cytometric analysis of TEM (CD44^+^CD62L^−^) and TCM (CD44^+^CD62L^+^) cells gating on CD4^+^ T cells in spleen of 4T1 tumour-bearing mice at the end of the treatment. Relative quantification of **f** TEMs and **g** ratio of TEMs**/**TCMs gating on CD8^+^T cells in the spleen of 4T1 tumour-bearing mice at the end of the treatment. Relative quantification of **h** TEMs and **i** ratio of TEMs**/**TCMs gating on CD4^+^ T cells in the spleen of 4T1 tumour-bearing mice at the end of the treatment. Data in **f**–**i** are presented as mean ± SD, *n* = 4 biologically independent samples in **f**–**i**. *P*-values are calculated by the two-tailed Student’s *t*-test in **f**–**i** as indicated in the figures. **P < 0.01 and ***P < 0.001. A representative image of three independent samples from each group is shown in **b** and **c**.

The lung and liver metastases of 4T1 tumour-bearing mice were further studied after treatment (Fig. 7a). Similar to that in PBS group, the obvious lung and liver metastasis was observed in Zeb, shPD-L1, and PPD groups (Fig. 7b, c). On the contrary, no metastases in the lung and liver were observed after combined treatment with PPD plus Zeb, and H&E staining images showed the normal histological morphology in lung and liver. This phenomenon demonstrated PPD plus Zeb could arouse systemic anti-tumour immune response and effectively inhibit the metastasis of extremely aggressive tumour. Furthermore, the mice treated with PPD plus Zeb were intravenously reinjected with 4T1 cells, and no lung and liver metastases were observed (Supplementary Figs. 108 and 109). The reason should be that PPD plus Zeb combination therapy greatly evoked a systemic immune memory effect (Fig. 7d–i) and effectively induced effector memory T cells to kill tumour cells, which finally prevented the lung and liver metastasis of tumour cells.

On the basis of the above results, PPD plus Zeb combination therapy not only significantly inhibited tumour growth by initiating an adaptive immune response, but also effectively prevented tumour relapse and metastasis by evoking a systemic anti-tumour immune memory effect.

### Tumour immunotherapy in humanized mice

**Fig. 8:**
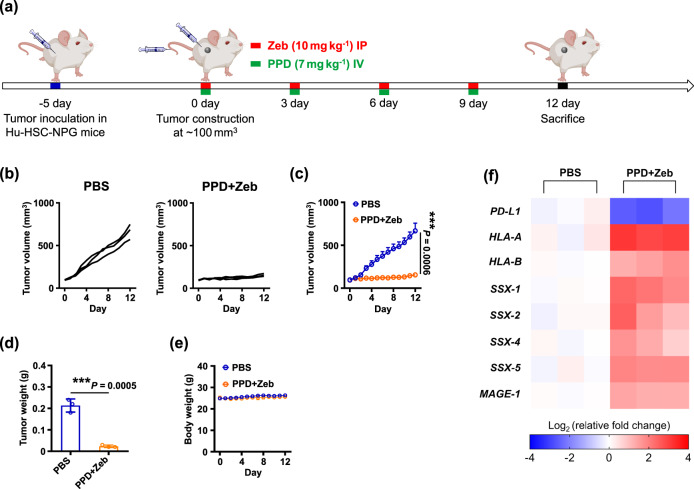
PPD combined with Zeb inhibited tumour growth in humanized mice. **a** Therapeutic schedule for PPD plus Zeb-mediated inhibition of tumour growth. **b** Individual and **c** average tumour growth kinetics in MDA-MB-231 tumour-bearing mice receiving PPD plus Zeb treatment. PBS was used as control. **d** Average tumour weight of MDA-MB-231 tumour-bearing mice receiving PPD plus Zeb treatment. **e** Average body weight changes of MDA-MB-231 tumour-bearing mice receiving PPD plus Zeb treatment. **f** Heatmap of relative mRNA expression of PD-L1, MHC I (HLA-A and HLA-B), and CTAs (SSX-1, SSX-2, SSX-4, SSX-5, and MAGE-1) in tumour tissues of MDA-MB-231 tumour-bearing mice at the end of treatment. Data in **c**–**e** are presented as mean ± SD, *n* = 3 biologically independent samples in **c**–**e**. *P*-values are calculated by the two-tailed Student’s *t*-test in **c** and **d** as indicated in the figures, ***P < 0.001.

Furthermore, to verify PPD plus Zeb-mediated anti-tumour effect in humanize mouse model, a humanized plasmid-encoding shPD-L1 (abbreviated as hu-shPD-L1) was designed. According to the RT-qPCR assay, mPEG-b-PLG/PEI-RT3/hu-shPD-L1 (denoted as PPD) significantly downregulated PD-L1 expression on MDA-MB-231 cells, whereas mPEG-b-PLG/PEI-RT3/humanized control shRNA (denoted as PPD-Control) did not significantly lower the level of PD-L1 on MDA-MB-231 cells (Supplementary Fig. 110). Moreover, MDA-MB-231 tumour-bearing Hu-HSC-NPG (humanized (Hu)-hematopoietic stem cells transplanted (HSC)-non-obese diabetes, protein kinase DNA-activated catalytic with severe combined immune deficiency, null IL-2 receptor gamma chain (NPG)) model was constructed to further evaluate the anti-tumour effect of PPD plus Zeb in humanized mice. As shown in Fig. 8b, c, compared with PBS group, PPD plus Zeb combination therapy significantly inhibited the tumour growth. Moreover, the tumour weight data showed a consistent therapeutic effect (Fig. 8d). The body weight of PPD plus Zeb group did not show significant changes throughout the process (Fig. 8e). Furthermore, the relevant genes in tumour tissues were detected by RT-qPCR assay. Compared with PBS group, the level of PD-L1 in PPD plus Zeb group was significantly downregulated (Fig. 8f). Moreover, MHC I molecules (HLA-A and HLA-B) and CTA genes (*SSX-1*, *SSX-2*, *SSX-4*, *SSX-5*, and *MAGE-1*) were remarkably upregulated after PPD plus Zeb treatment. These results suggested that PPD plus Zeb combination therapy not only downregulated PD-L1 expression on tumour cells to relieve the immunosuppression of T cells, but also induced the enhancement of MHC I molecules on tumour cells via CTA, thereby ultimately mounting the visibility of tumour cells to the adaptive immune system in a humanized mouse model. According to the anti-tumour effect in humanized mice, PPD plus Zeb combination therapy has broad prospects for clinical application.

## Discussion

We developed a polycationic gene delivery system (named as PPD) by introducing multiple interactions (electrostatic, hydrogen bonding, and hydrophobic interactions) and PPII-helix conformation. The anti-tumour effects of this polymeric gene delivery system in vivo were explored. With the plasmid-encoding shPD-L1 as a therapeutic gene, PPD mediating shPD-L1 delivery combined with epigenetic regulation (DNMTi, Zeb) synergistically relieved the immune escape mechanism of tumour cells, thus promoting an in vivo anti-tumour immune response and enhancing the efficacy of tumour treatment. This treatment could also initiate a systemic anti-tumour immune response and a durable immune memory effect that effectively inhibited tumour relapse and metastasis, respectively. Moreover, PPD plus Zeb combination therapy could also significantly inhibit tumour growth in humanized mice. Epigenetic regulation combined with gene therapy-mediated immune checkpoint blockade was carried out to relieve the immune escape mechanism of tumour cells. This strategy provides a therapeutic scheme for cancer immunotherapy and opens up a platform for cancer treatment.

## Methods

### Materials

Branched PEI25k and PEI1.8k was purchased from Sigma (USA). Boc-Orn(Boc)-OH, Boc-Arg(Pbf)-OH, Boc-Orn(Tos)-OH, Boc-Arg(Tos)-OH, and H-Glu(Bzl)-OH were purchased from GL Biochem Ltd (Shanghai, China). Boc-Arg(NO_2_)-OH was purchased from Enlai Biological Technology Co., Ltd (Chengdu, Sichuan). Trifluoroacetic acid (TFA) and triphosgene were obtained from Xiya Reagent Co., Ltd. *N*, *N*-dimethyl formamide (DMF) and anhydrous ether were obtained from Beijing Chemical Factory. *P*-toluene formyl chloride, *p*-toluene sulfonyl chloride, diisopropylethylamine (DIPEA), and 1-hydroxybenzotriazoleanhydrous (HOBT) were obtained from Dibai Chemical Technology Co., Ltd (Shanghai, China). EDC hydrochloride (EDCI) was obtained from J&K Scientific Ltd (Beijing, China). *N*-hydroxysuccinimide was obtained from TCI Co., Ltd (Shanghai, China). Then, 33% HBr in CH_3_COOH was obtained from ACROS. mPEG-NH_2_ (Mn = 2000) was obtained from Beijing Jenkem Technology Co., Ltd (Beijing, China). Dialysis bag was obtained from Yuanye Biological Technology Co., Ltd (Shanghai, China). Calf thymus DNA was obtained from Sigma (USA). The plasmid DNA (pDNA) expressing mouse shPD-L1 (named as shPD-L1) was obtained by Sangon (Shanghai, China). The sense strand sequence for shPD-L1 was 5′-CCCACAUAAAAAACAGUUGdTdT-3′. The pDNA expressing control shRNA was named as Control shRNA. The sense strand sequence for Control shRNA was 5′-GTTCTCCGAACGTGTCACGTT-3′. The pDNA expressing human shPD-L1 (named as hu-shPD-L1) was obtained by Genepharama (Shanghai, China), the sense sequence for hu-shPD-L1 was 5′-GCCGACTACAAGCGAATTACTT-3′. Zeb was obtained from TCI Co., Ltd (Shanghai, China). AC and DAC were obtained from J&K Scientific Ltd (Beijing, China). DAPI was obtained from Sigma. CCK-8 was obtained from Beyotime Biotechnology Co., Ltd (Shanghai, China). Plasmid-encoding luciferase (pGL3), luciferin substrate, and cell lysate were obtained from Promega (Madison, USA). GL3 luciferase-siRNA double strands (sense: 5′-CUUACGCUGAGUACUUCGAdTdT-3′, antisense: 5′-UCGAAGUACUCAGCGUAAGdTdT-3′) and nonspecific control siRNA double strands (sense: 5′-AGCUUCAUGAGUCGCAUUCdTdT-3′ and antisense: 5′-GAAUGCGACUCAUGAAGCUdTdT-3′) were obtained from FASMAC (Japan). H2-K^b^ siRNA double stands (sense: 5′-CCAGACCUCUGAUCUGUCUdTdT-3′ and antisense: 5′-AGACAGAUCAGAGGUCUGGdTdT-3′) and control siRNA double strands (sense: 5′-UUCUCCGAACGUGUCACGUdTdT-3′ and 5′-ACGUGACACGUUCGGAGAAdTdT-3′) were obtained Genepharama (Shanghai, China). RT-qPCR primers were obtained from Sangon (Shanghai, China), and the primer sequences were shown in Supplementary Table 4. RNA extraction Kit, RNA reverse-transcription Kit, and qPCR Kit were obtained from TIANGEN Co., Ltd (Beijing, China). All fluorescence-labelled antibodies used for flow cytometry detection was obtained from eBioscience or Biolegend; the detailed information was shown in Supplementary Table 5. Cells were measured by Guava EasyCyte flow cytometer and BD FCAS Celesta flow cytometer. The slides were observed with confocal microscope (ZEISS LSM780). Enzyme-linked immunosorbent assay (ELISA) kits of tumour-associated antigens (MAGE-E1, CD146, and TRP-1) were obtained from Jiancheng Biotechnology Co., Ltd (Nanjing, China). ELISA kits of cytokines (IL-6, IFN-γ, TNF-α, GzmB, IL-10, TGF-β, and PD-L1) and MHC I molecules were purchased from eBioscience (CA, USA). The contents of tumour-associated antigens, cytokines, and MHC I molecules were measured according to the manufacturer’s instructions.

### Synthesis of PEI-RT3

PEI1.8k (1.0 g, 0.556 mmol) was dissolved in 10 mL of deionized water. The mixture of Boc-Arg (Tos)-OH (2.38 g, 5.56 mmol), HOBT (1.13 g, 8.33 mmol), and EDCI (1.60 g, 8.33 mmol) were dissolved with 50 mL of DMF and stirred at room temperature for 1 h. Then PEI1.8k solution and DIPEA (1.08 g, 8.33 mmol) were added and stirred at room temperature for 72 h. After that, the mixture solution was dialyzed (dialysis bag: cut-off 3500 Da), and lyophilized, then the white solid was obtained. The white solid was added into 30 mL of TFA and reacted at room temperature for 4 h. Then the mixture solution was precipitated with cold anhydrous ether, filtered, and dried in vacuum, dialyzed (dialysis bag: cut-off 3500 Da), and lyophilized, and the white flocculent solid was obtained (1.62 g, 59.34% yield). ^1^H NMR (400 MHz, δ, p.p.m., D_2_O/CD_3_CN = 1/1 (v/v)): 7.65 (d, 21.2H), 7.28 (d, 20.4H), 3.88 (t, 10.2H), 2.52–3.62 (m, 188.0H), 2.33 (s, 30.7H), 1.76 (m, 20.3H), 1.50 (m, 20.5H). The synthesis procedure of other PEI-RTs containing different numbers of RT string was similar.

### Synthesis of PEI-Too

PEI1.8k (1.0 g, 0.556 mmol) was dissolved in 10 mL of deionized water. *P*-toluoyl chloride (859.7 mg, 5.56 mmol) was dissolved in 8 mL of THF. The *p*-toluoyl chloride solution was added into the ice bath of PEI1.8k solution drop by drop. Then DIPEA (1.44 mg, 11.12 mmol) was added and reacted in ice bath for 12 h. After that, the mixture solution was dialyzed (dialysis bag: cut-off 3500 Da) for 72 h, lyophilized, and the white solid was obtained. (1.18 g, 67.06% yield). ^1^H NMR (400 MHz, δ, p.p.m., D_2_O/CD_3_CN = 1/1 (v/v)): 7.62 (d, 17.9H), 7.21 (d, 20.7H), 2.42–3.60 (m, 197.0H).

### Synthesis of PEI-Tos

PEI1.8k (1.0 g, 0.556 mmol) was dissolved in 10 mL of deionized water. *P*-toluene sulfonyl chloride (1.06 g, 5.56 mmol) was dissolved in 12 mL of THF. *P*-toluene sulfonyl chloride solution was added into the ice bath of PEI1.8k solution drop by drop. Then DIPEA (1.44 g, 11.12 mmol) was added and reacted in ice bath for 12 h. After that, the mixture solution was dialyzed (dialysis bag: cut-off 3500 Da) for 72 h, lyophilized, and the white solid was obtained. (1.34 g, 68.37% yield). ^1^H NMR (400 MHz, δ, p.p.m., D_2_O/CD_3_CN = 1/1 (v/v)): 7.65 (d, 21.4H), 7.32 (d, 20.2H), 2.18–3.25 (m, 198.6H).

### Synthesis of PEI-Orn

PEI1.8k (1.0 g, 0.556 mmol) was dissolved in 10 mL of deionized water. The mixture of Boc-Orn(Boc)-OH (1.85 g, 5.56 mmol), HOBT (1.13 g, 8.33 mmol), and EDCI (1.60 g, 8.33 mmol) were dissolved with 50 mL of DMF and stirred at room temperature for 1 h. Then PEI1.8k solution and DIPEA (1.08 g, 8.33 mmol) were added and stirred at room temperature for 72 h. After that, the mixture solution was dialyzed (dialysis bag: cut-off 3500 Da), lyophilized, and the white solid was obtained. The white solid was added into 30 mL of TFA and reacted at room temperature for 4 h. Then the mixture solution was precipitated with cold anhydrous ether, filtered and dried in vacuum, dialyzed (dialysis bag: cut-off 3500 Da) and lyophilized, the white flocculent solid was obtained (1.08 g, 65.93% yield). ^1^H NMR (400 MHz, δ, p.p.m., D_2_O/CD_3_CN = 1/1 (v/v)): 3.57 (t, 10.6H), 2.52–3.40 (m, 189.2H), 1.55–1.80 (m, 40.3H).

### Synthesis of PEI-Arg

PEI1.8k (1.0 g, 0.556 mmol) was dissolved in 10 mL of deionized water. The mixture of Boc-Arg(Pbf)-OH (2.93 g, 5.56 mmol), HOBT (1.13 g, 8.33 mmol), and EDCI (1.60 g, 8.33 mmol) were dissolved with 60 mL of DMF and stirred at room temperature for 1 h. Then PEI1.8k solution and DIPEA (1.08 g, 8.33 mmol) were added and stirred at room temperature for 72 h. After that, the mixture solution was dialyzed (dialysis bag: cut-off 3500 Da), lyophilized, and the white solid was obtained. The white solid was added into 40 mL of TFA and reacted at room temperature for 4 h. Then the mixture solution was precipitated with cold anhydrous ether, filtered and dried in vacuum, dialyzed (dialysis bag: cut-off 3500 Da) and lyophilized, the white flocculent solid was obtained (1.04 g, 50.66% yield). ^1^H NMR (400 MHz, δ, p.p.m., D_2_O/CD_3_CN = 1/1 (v/v)): 3.97 (t, 10.4H), 2.62–3.80 (m, 188.8H), 1.85 (t, 19.8), 1.58 (m, 21.3H).

### Synthesis of PEI-Orn(Tos)

PEI1.8k (1.0 g, 0.556 mmol) was dissolved in 10 mL of deionized water. The mixture of Boc-Orn(Tos)-OH (2.15 g, 5.56 mmol), HOBT (1.13 g, 8.33 mmol), and EDCI (1.60 g, 8.33 mmol) were dissolved with 50 mL of DMF and stirred at room temperature for 1 h. Then PEI1.8k solution and DIPEA (1.08 g, 8.33 mmol) were added and stirred at room temperature for 72 h. After that, the mixture solution was dialyzed (dialysis bag: cut-off 3500 Da), lyophilized, and the white solid was obtained. The white solid was added into 40 mL of TFA and reacted at room temperature for 4 h. Then the mixture solution was precipitated with cold anhydrous ether, filtered and dried in vacuum, dialyzed (dialysis bag: cut-off 3500 Da) and lyophilized, the white solid was obtained (1.41 g, 56.53% yield). ^1^H NMR (400 MHz, δ, p.p.m., D_2_O/CD_3_CN = 1/1 (v/v)): 7.65 (d, 21.2H), 7.36 (d, 20.3H), 3.62 (t, 10.7H), 2.24-3.46 (m, 221.5H), 1.21–1.62 (m, 40.8H).

### Synthesis of PEI-Arg(NO_2_)

PEI1.8k (1.0 g, 0.556 mmol) was dissolved in 10 mL of deionized water. The mixture of Boc-Arg(NO_2_)-OH (1.75 g, 5.56 mmol), HOBT (1.13 g, 8.33 mmol), and EDCI (1.60 g, 8.33 mmol) were dissolved with 60 mL of DMF and stirred at room temperature for 1 h. Then PEI1.8k solution and DIPEA (1.08 g, 8.33 mmol) were added and stirred at room temperature for 72 h. After that, the mixture solution was dialyzed (dialysis bag: cut-off 3500 Da), lyophilized, and the white solid was obtained. The white solid was added into 40 mL of TFA and reacted at room temperature for 4 h. Then the mixture solution was precipitated with cold anhydrous ether, filtered and dried in vacuum, dialyzed (dialysis bag: cut-off 3500 Da) and lyophilized, the white solid was obtained (1.39 g, 66.38% yield). ^1^H NMR (400 MHz, δ, p.p.m., D_2_O/CD_3_CN = 1/1 (v/v)): 3.76 (t, 10.8H), 2.42–3.56 (m, 189.6H), 1.75 (t, 21.8), 1.56 (t, 21.3H).

### Synthesis of Glu(OBzl)-NCA

To obtain mPEG-b-PLG, Glu (OBzl)-NCA needed to be synthesized. Briefly, H-L-Glu (OBzl)-OH (35.6 g, 0.15 mol) and triphosgene (19.7 g, 0.1 mol) were added into a dry three-mouth flask, then 500 mL of THF was added and reacted for 2 h at 50 °C until the solution was clear. Then the mixture solution is naturally cooled to room temperature and precipitated with 2 L of cold hexane, filtered, the white powder was dissolved with 250 mL of ethyl acetate, washed with cold deionized water for three times, dried with anhydrous magnesium sulfate, and stored overnight at −20 °C. Then the mixture solution was filtered and the filtrate was dried under vacuum condition, recrystallized with THF and hexane, the white solid was obtained (30.2 g, 76.53% yield). ^1^H NMR (400 MHz, δ, p.p.m., CDCl_3_): 7.33-7.45 (m, 5H), 6.44 (s, 1H), 5.16 (s, 2H), 4.28 (t, 1H), 2.25–2.38 (m, 2H), 2.08–2.20 (m, 2H).

### Synthesis of mPEG-b-PLG

Glu(OBzl)-NCA (1.315 g, 5 mmol) was dissolved in 20 mL of DMF, mPEG_2000_-NH_2_ (0.5 g, 0.25 mmol) was added into the above solution, and reacted for 72 h at room temperature. Then the mixture solution was dialyzed against deionized water for 72 h (dialysis bag: cut-off 2000 Da), lyophilized, and the white solid was obtained. The white solid was dissolved in 20 mL of TFA, 33% HBr in CH_3_COOH (4 mL solution/1 g solid) was added slowly and reacted for 4 h at room temperature, the mixture solution was precipitated with anhydrous ether, filtered, dried under vacuum condition, dialyzed for 72 h (cut-off 2000 Da), lyophilized, and the white solid was obtained (0.74 g, 64.63% yield). ^1^H NMR (400 MHz, δ, p.p.m., CF_3_COOD): 4.58 (t, 17.0H), 3.67 (t, 180.0H), 3.33 (m, 2.97H), 2.42 (t, 34.6H), 1.80–2.22 (m, 35.7H).

### Gel permeation chromatography assay

To obtain the molecular weight and molecular weight distribution of PEI-RT3 or mPEG-b-PLG, gel permeation chromatography measurements were carried with a Waters 515 chromatography column. Then, 0.1 M of NaNO_3_ was used as the eluent for mPEG-b-PLG determination, respectively. Molecular weights were calculated by a calibration curve of linear mPEG2k standards.

### Preparation of nanoparticles

The preparation of carrier/DNA complexes was described as the following steps. Then, 50 μL of DNA (1 mg mL^−1^) was mixed with 250 μL of PEI-RT3 solution (1 mg mL^−1^). After that, 1700 μL of deionized water was added. After 20 s vortex, the above mixture solution was incubated for 20 min at room temperature to obtain PD complexes. The other carrier/DNA complexes were prepared in a similar manner. For the preparation of PPD complexes, 50 μL of DNA (1 mg mL^−1^) was mixed with 250 μL of PEI-RT3 solution (1 mg mL^−1^), then 1650 μL of deionized water was added. After 20 s vortex, the above mixture solution was incubated for 20 min at room temperature, then 50 μL of mPEG-b-PLG was added. After 20 s vortex, the above mixture solution was incubated for 20 min at room temperature to obtain PPD complexes.

### Determination of particle size and zeta potential

Zeta potential and particle size of PPD and other carrier/DNA complexes was characterized with zeta potential/BI-90Plus particle size analyzer (Brookhaven, USA). Individual data were shown based on quintuplicate independent experiments. In addition, DLS technique was also used to determinate hydrodynamic particle size of nanoparticles with Zetasizer Pro (Malvern, USA). The morphologies of PPD and PD were characterized with scanning electron microscopy (SEM) containing XL-30ESEM-FEG SEM system (SEI, USA).

### CD spectra assay

The CD spectra of mPEG-b-PLG was measured with J-815 CD spectrometer (Easton, MD, USA). The sample of mPEG-b-PLG was prepared at concentration of 0.2 mg mL^−1^ in deionized water. The pH value of mPEG-b-PLG solution was adjusted to the desired value (pH 6.8 or 7.4). The mean residue molar ellipticity was calculated wth the following formulas: Ellipticity ([θ] in deg cm^2^ dmol^−1^) = (millidegrees × mean residue weight)/(path length in millimetres × concentrations of polypeptide in mg mL^−1^). The CD spectra were measured at room temperature.

### Cell culture

B16F10 (TCM36), 4T1, HeLa (TCHu187), MCF-7 (TCHu74), and Huh-7 (TCHu182) cells were purchased from Shanghai Cell Bank (Chinese Academy of Sciences). The cells were cultured in DMEM medium containing 10% (v/v) fetal bovine serum at 37 °C in 5% (v/v) carbon dioxide (CO_2_) (Thermo Forma Incubator, USA)

### DNA transfection

#### In vitro

DNA transfection assay was conducted in B16F10, 4T1, HeLa, and MCF-7 cells. Luciferase plasmid DNA (pGL3) was used as reporter gene. Cells were seeded in 96-well plate at a density of 10^4^ cells per well and incubated at 37 °C in 5% CO_2_ overnight. Then culture medium was replaced with fresh culture medium at a volume of 180 μL per well. Carrier/pDNA complexes at different mass ratios (20/1, 10/1, 5/1, 2.5/1, and 1/1) were added into cells containing plates (0.2 μg pDNA per well) and incubated for 48 h. the cells was lysed with 50 μL of cell lysate per well and frozen at −80 °C for 30 h. After melting at room temperature, 25 μL of cell lysate per well was mixed with 25 μL of luciferase substrate. Relative light units (RLUs) were obtained by a luminometer and normalized to total protein content (BCA protein assay kit, Sigma). The luciferase activity was expressed as RLU/mg protein) and the data were shown as mean ± SD based on triplicate independent experiments.

### Gene silencing

#### In vitro

Huh-7 Luc cells were seeded in a 96-well plate at a density of 10^4^ cells per well and incubated for 24 h. The culture medium was replaced with fresh culture medium and carrier/siRNA complexes was added (0.2 μg siRNA per well) and incubated for 48 h. Then the cells were lysed with 50 μL of cell lysate per well and frozen at −80 °C for 30 min. After melting at room temperature, 25 μL of cell lysate per well was mixed with 25 μL of luciferase substrate. RLUs were obtained by luminometer and normalized to total protein content (BCA protein assay kit, Sigma). The luciferase activity was expressed as RLU/mg protein) and the relative luciferase activity was calculated by comparing untreated control cells. The gene silencing data were shown as mean ± SD based on triplicate independent experiments.

### Cytotoxicity assay

The cytotoxicity of carrier/pDNA was characterized by CCK-8 assay. B16F10 cells (or other kinds of cells) were seeded in a 96-well plate at a density of 10^4^ per well and incubated with 24 h. After that, the culture medium was replaced with fresh culture medium at a volume of 180 μL per well. Carrier/pDNA complexes at different mass ratios (20/1, 10/1, 5/1, 2.5/1, and 1/1) were added into cells containing plates (0.2 μg pDNA per well) and incubated for 24 h. Then the culture medium was replaced with 10% (v/v) CCK-8 containing culture medium and incubated for 1 h. The cells were detected at 450 nm and the absorption value was obtained by Bio-Rad 680 microplate reader. Cell viability (%) was calculated based on this equation: cell viability (%) = (*A*_sample_/*A*_control_) × 100, where *A*_sample_ and *A*_control_ were the absorbance values of sample well and control well, respectively. The data were shown as mean ± SD based on triplicate independent experiments.

### Cellular uptake

The cellular uptake efficiency of carrier/DNA complexes was detected by flow cytometry. Cells were seeded in 24-well plates at a density of 10^5^ cells per well and incubated for 24 h. After that, the culture medium was replaced with fresh culture medium and carrier/Cy5-DNA complexes were added and incubated for 3 h. Then the cells were digested, centrifuged (0.1 g), and washed with PBS for three times. The cells were detected with Guava EasyCyte low cytometer. In addition, CLSM was carried out to observe the cellualr uptake of carrier/DNA directly. Cells were seeded on a coverslip containing six-well plates at a density of 10^5^ cells per well and incubated for 24 h. The culture medium was replaced with fresh medium. Carrier/Cy5-DNA complexes were added and incubated for 3 h. Then cells were washed with PBS for three times and fixed for 10 min by 4% paraformaldehyde. Nucleus was stained with DAPI for 10 min. The coverslips were taken out, placed on glass slides and enclosed with glycerol. The prepared samples were observed with CLSM (ZEISS LSM780).

### Endo/lysosomal escape

B16F10, 4T1, HeLa, or MCF-7 cells were seeded in six-well plates containing coverslips at a density of 1 × 10^5^ cells per well and incubated for 24 h. Then the culture medium was replaced with fresh culture medium and carrier/Cy5-DNA complexes (1 mg Cy5-DNA per well) were added and incubated for 1, 4, and 7 h. After that, the cells were washed with PBS and fixed with 4% paraformaldehyde for 10 min. Nucleus were stained with 1 mg mL^−1^ of DAPI (1.5 μL per well) for 10 min, and endo/lysosome were stained with 75 nM of Lysotracker Green for 1 h. Finally, the coverslips were taken out and placed on the glass slides, enclosed with glycerol. The samples were observed by CLSM (ZEISS LSM780, Germany) and the correlation coefficient *R*-values were obtained from CLSM images (ZEN software).

### Construction of tumour model

Six-week-old female C57BL/6 and BALB/c mice were obtained from Vital River Company (Beijing, China). All animal procedures were strictly perfromed based on the Guidelines for the Care and Use of Laboratory Animals of Jilin University and approved by the Animal Ethics Committee of Jilin University. B16F10 cells were large scale expanded in vitro, and cells suspensions in PBS (100 μL, 2.0 × 10^7^ cells/mL) were transplanted into the left flank of mice and tumour growth was monitored. For primary B16F10 tumour therapy, the solid tumour model was formed after 4 days, and the tumour volumes were about 50 mm^3^. The second tumour as distant tumour model (100 μL, 2.0 × 10^7^ cells/mL) was inoculated into the right flank of mice after 10 days. For distribution and gene transfection in B16F10 tumour-bearing mice, solid B16F10 tumour model was formed after 8 days and tumour volumes were about 500 mm^3^. For anti-tumour efficacy study, solid B16F10 tumour model was formed after 4 days and the tumour volumes were about 50 mm^3^. In addition, for the construction of 4T1 tumour model, 4T1 cells were large scale expanded in vitro and cell suspensions in PBS (100 μL, 2.0 × 10^7^ cells/mL) were transplanted into the left flank of mice and tumour growth was monitored. For primary 4T1 tumour therapy, the solid tumour model was formed after 7 days and the tumour volumes were about 100 mm^3^, and the second tumour as distant tumour model (100 μL, 2.0 × 10^7^ cells/mL) was inoculated into the right flank of mice after 14 days. For distribution and gene transfection in 4T1 tumour-bearing mice, solid 4T1 tumour model was formed after 12 days and tumour volumes were about 500 mm^3^. For anti-tumour efficacy study, solid 4T1 tumour model was formed after 7 days and the tumour volumes were about 100 mm^3^.

### Pharmacokinetics assay

The mice were administered intravenously with 200 μL of Cy5-DNA, PEI1.8k/Cy5-DNA (5/1), PEI-RT3/Cy5-DNA (5/1), and mPEG-b-PLG/PEI-RT3/Cy5-DNA (1/5/1), respectively. The blood was collected by tain vein and transferred to heparinized centrifuge tubes. Then the blood was diluted with delionied water and then transferred into a black 96-well plate. The sample was detected at *λ*_ex_ = 640 nm and *λ*_em_ = 680 nm with a Tecan infinate M200 Microplate Reader. The untreated well was used as control. The concentration of sample was calculated based on Cy5-DNA calibration curves.

### In vivo distribution

The distribution of carrier/DNA complexes in vivo was detected and analyzed by ex vivo bioluminescence imaging assay. Then, 200 μL of carrier/Cy5-DNA (1 mg Cy5-DNA/1 kg body weight) was administered intravenously into B16F10 (or 4T1) tumour-bearing mice. After 24 h, mice were killed, and tumour and major organs (heart, liver, spleen, lung, and kidney) were collected and imaged with Maestro In Vivo Imaging System (Cambridge Research & Instrumentation, USA).

### Distribution within tumour

B16F10 or 4T1 tumour-bearing mice were intravenously administered with mPEG-b-PLG/PEI-RT/Cy5-DNA (PPD) complexes. After 24 h, mice were killed and tumour tissue was collected. The frozen sections of tumour were stained with DAPI and observed under fluorescence microscope for the distribution analysis of carrier/DNA complexes.

### In vivo transfection

Transfection activity of carrier/pDNA in vivo was measured by ex vivo bioluminescence imaging assay. pDNA encoding red fluorescence protein was served as a reporter gene. Then, 200 μL of pDNA, PEI1.8k/pDNA, PEI-RT3/pDNA, and mPEG-b-PLG/PEI-RT3/pDNA were intravenously injected into B16F10 (or 4T1) tumour-bearing mice, respectively (1 mg pDNA/1 kg body weight). After 48 h, the mice were killed, tumour and main organs (heart, liver, spleen, lung, and kidney) were collected and analyzed with Maestro In Vivo Imaging System (Cambridge Research & Instrumentation, USA).

### Transfection within tumour

To characterize the distribution of the expressed protein within tumours, the plasmid-encoding RFP was used as reporter gene, and B16F10 or 4T1 tumour were intravenously administered with mPEG-b-PLG/PEI-RT/*p*DNA complexes. After 48 h, mice were killed and tumour tissue was collected. The frozen sections of tumour were stained with DAPI and observed under fluorescence microscope for the distribution analysis of expressed protein.

### PD-L1 expression on various cells in tumour tissue

To characterize gene transfection of carrier/shPD-L1 in different cell types in the tumour microenvironment, the levels of PD-L1 expressed on various cell lines in tumours were studied. GFP-B16F10 cells were selected to construct the tumour model. When the tumour volume arrived at 200 mm^3^, mPEG-b-PLG/PEI-RT/shPD-L1 (PPD) ternary complexes were administered intravenously to the tumour-bearing mice. Then the mice were killed and tumours were collected and the levels of PD-L1 in DCs, macrophages, and tumour cells in tumour tissue were analyzed by flow cytometry.

### Tumour treatment

pDNA encoding shPD-L1 (named as shPD-L1) was a therapeutic gene. For B16F10 tumour treatment, 60 female C57BL/6 mice (average tumour volume, 50 mm^3^) were randomly divided into 6 groups (10 mice per group), namely PBS, Zeb, shPD-L1, PD, PPD, and PPD plus Zeb. Tumour-bearing mice were intravenously administered with 200 μL of PBS, shPD-L1, PD, and PPD every other day for a total of five times, respectively. Dose of shPD-L1 was 1 mg kg^−1^. For Zeb and PPD plus Zeb group, Zeb was paratumourally administered to tumour-bearing mice every other day for a total of five times. Dose of Zeb was 10 mg kg^−1^. Body weight was recorded every day for 12 days. Tumour size was measured every day for 12 days and calculated according to the equation: *V* = *L* × *W*^2^ × 0.5, where ‘*V*’ represents tumour volume, ‘*L*’ represents the longer diameter, and ‘*W*’ represents the shorter diameter. In addition, during various DNMTi treatments, for PPD plus AC or PPD plus DAC, AC or DAC was paratumourally administered to tumour-bearing mice every other day for a total of five times, and dose of AC or DAC was 5 mg kg^−1^. Furthermore, during aPD-L1 involved tumour therapy, for aPD-L1 or aPD-L1 plus PPD group, aPD-L1 was injected intraperitoneally into tumour-bearing mice every other day for a total of five times, dose of aPD-L1 was 7.5 mg kg^−1^. After treatment, mice were killed and spleens were collected for the flow cytometry analysis of Th17 cells. During exploring the role of MHC I molecules in Zeb plus PPD treatment, for PPD plus Zeb plus PP/siH2-K^b^ group, the dose of siH2-K^b^ was 1 mg kg^−1^. For 4T1 tumour treatment, 60 female BABL/c (average tumour volume, 100 mm^3^) were randomly divided into 6 groups (10 mice per group), namely PBS, Zeb, shPD-L1, PD, PPD, and PPD plus Zeb. Tumour-bearing mice were intravenously administered with 200 μL of PBS, shPD-L1, PD, and PPD every other day for a total of seven times respectively. Dose of shPD-L1 was at 1 mg kg^−1^. For Zeb and PPD plus Zeb group, Zeb was paratumourally administered to tumour-bearing mice every other day for a total of seven times. Dose of Zeb was 10 mg kg^−1^. Body weight was recorded every day for 24 days. Tumour size was measured every day for 24 days and calculated according to the equation: *V* = *L* × *W*^2^ × 0.5.

### In vivo safety assessment

To evaluate in vivo safety of PPD complexes, the blood of tumour-bearing mice was collected after treatment. Age- and weight-matched healthy mice were used as control. The blood was centrifuged at 0.9 × *g* for 15 min and the serum was collected. Liver function markers alkaline phosphatase, alanine aminotransferase, aspartate aminotransferase (ALP, ALT, AST) and renal function markers creatinine, uric acid, blood urea nitrogen (CRE, UA, BUN) in serum were measured by ELISA assay (Lengton, Shanghai, China).

### Survival observation

The survival rate of tumour-bearing mice was carried out both in B16F10 and 4T1 tumour models. For B16F10 tumour treatment, 60 female C57BL/6 mice (average tumour volume, 50 mm^3^) were randomly divided into 6 groups (10 mice per group), namely, PBS, Zeb, shPD-L1, PD, PPD, and PPD plus Zeb. Tumour-bearing mice were intravenously administered with 200 μL of PBS, Zeb, shPD-L1, PD, PPD, or PPD every other day for a total of five times, respectively. Dose of shPD-L1 was 1 mg kg^−1^. For Zeb and PPD plus Zeb group, Zeb was paratumourally administered to tumour-bearing mice every other day for a total of five times. Tumour size was measured every day. When the tumour volume of mice arrived at 1000 m^3^, the mice was defined as dead. For 4T1 tumour treatment, 60 female BABL/c (average tumour volume, 100 mm^3^) were randomly divided into 6 groups (10 mice per group), namely PBS, Zeb, shPD-L1, PD, PPD, and PPD plus Zeb. Tumour-bearing mice were intravenously administered with 200 μL of PBS, shPD-L1, PD, and PPD every other day for a total of seven times, respectively. Dose of shPD-L1 was 1 mg kg^−1^. For Zeb and PPD plus Zeb group, Zeb was paratumourally administered to tumour-bearing mice every other day for a total of seven times. Dose of Zeb was 10 mg kg^−1^. Tumour size was measured every day. When the tumour volume of mice arrived at 1000 m^3^, the mice was defined as dead.

### Reverse-transcription-quantitative real-time PCR reaction

To measure the relative levels of genes in cancer cells (A549, HepG2, B16F10, and MDA-MB-231 cells) and tumour tissues, the RT-qPCR assay was carried out. Total RNA of cancer cells or tumour tissues were extracted from cancer cells by RNA extraction kit and RNA content was measured. Then RNA reverse-transcription assay was conducted by RNA reverse-transcription kit and acquired cDNA content was measured. Finally, qPCR assay of acquired cDNA was conducted with SYBR green technology by qPCR Kit and analyzed with Roche Light Cycler.

### Tumour relapse study

The tumour relapse in B16F10 (or 4T1) tumour-bearing mice was studied. Six-week-old female C57BL/6 mice (six mice per group) was injected with B16F10 cells (100 μL, 2.0 × 10^7^ cells/mL) was transplanted into the left flank of mice. The solid B16f10 tumour model was formed after 4 days and mice were treated as we designed. After treatment, B16F10 or MC38 or LLC cells (100 μL, 2.0 × 10^7^ cells/mL) were transplanted into the right flank of mice as designed to study the tumour relapse. Right flank tumour was measured every other day and calculated according to the equation: *V* = *L* × *W*^2^ × 0.5. Moreover, 6-week-old female BABL/c mice (eight mice per group) was injected with 4T1 cells (100 μL, 2.0 × 10^7^ cells/mL) was transplanted into the left flank of mice. The solid 4T1 tumour model was formed after 7 days and mice were treated as we designed. After treatment, 4T1 or CT26 cells (100 μL, 2.0 × 10^7^ cells/mL) cells were transplanted into the right flank of mice as designed to study the tumour relapse. Right flank tumour was measured every other day and calculated according to the equation: *V* = *L* × *W*^2^ × 0.5.

### Tumour metastasis study

Six-week-old female BABL/c mice (12 mice for PPD and other groups were based on 6 mice per group) was injected with 4T1 cells (100 μL, 2.0 × 10^7^ cells/mL) was transplanted into the left flank of mice. The solid tumour model was formed after 7 days and mice were treated as we designed. After treatment, half of mice in PPD group and all mice in other groups were killed, the liver, spleen and lung were collected and further analyzed. In addition, the remaining mice in PPD were intravenously administered with 4T1 cells (100 μL, 2.0 × 10^6^ cells/mL). After 7 days, the liver and lung were collected and analyzed.

### Humanized mouse model

Hu-HSC-NPG model was obtained from Beijing Vitalstar Biotechnology Co., Ltd, and the construction process of this model was as follows: CD34^+^ cells were isolated and purified from human umbilical cord blood. NPG female mice aged 4 weeks were irradiated (1.6 Gy) for 4 H, then CD34^+^ cells were intravenously administered at dose of 1 × 10^5^ cells per mice. After 20 weeks, the contents of human and mouse derived CD45^+^ cells in mouse peripheral blood were detected by flow cytometry to test the qualified rate of Hu-HSC-NPG reconstruction (namely, the percentage of human CD45^+^ cells exceeded 25%). The relevant data were presented in Supplementary Table 6 Then, Hu-HSC-NPG mice were inoculated subcutaneously with MDA-MB-231 cells at dose of 2 × 10^6^ cells per mice, and Hu-HSC-NPG mice were randomly divided into two groups (three mice per group): PBS, PPD plus Zeb. When the average tumour volumes reached 50 mm^3^, Hu-HSC-NPG mice were administered with PPD plus Zeb or PBS every two days for total five times.

### Reporting summary

Further information on research design is available in the Nature Research Reporting Summary linked to this article.

## Supplementary information

Supplementary Information

Peer Review File

Reporting Summary

## Data Availability

All the data supporting this study are available in the manuscript and its Supplementary Information file. Source data are provided with this paper.

## Source data

Source Data
