# Supplementary material for: Combination of epigenetic regulation with gene therapy-mediated immune checkpoint blockade induces anti-tumour effects and immune response in vivo
Source: Nat Commun. 2021 Nov 18;12:6742. doi: 10.1038/s41467-021-27078-x (PMC8602287; doi:10.1038/s41467-021-27078-x)
Supplement: Supplementary file 1 — Supplementary Information [file 41467_2021_27078_MOESM1_ESM.pdf]

Supplementary Information

Combination of epigenetic regulation with gene therapy-mediated immune checkpoint blockade induces anti-tumour effects and immune response in vivo

Huapan Fang[1, 2, 3, 4, †], Zhaopei Guo[1, †], Jie Chen[1, 2, 3], Lin Lin[1, 2, 3], Yingying Hu[1, 2, 3], Yanhui Li[*, 5], Huayu Tian[*, 1, 2, 3], Xuesi Chen[1, 2, 3]

[1] Key Laboratory of Polymer Ecomaterials, Changchun Institute of Applied Chemistry, Chinese Academy of Sciences, Changchun 130022, China

[2] University of Science and Technology of China, Hefei 230026, China

[3] Jilin Biomedical Polymers Engineering Laboratory, Changchun 130022, China

[4] Institute of Functional Nano and Soft Materials (FUNSOM), Jiangsu Key Laboratory for Carbon Based Functional Materials & Devices, Soochow University, Suzhou, 215123, Jiangsu, China

[5] School of Materials Science and Engineering, Changchun University of Science and Technology, Changchun 130022, China

† These authors contributed equally: Huapan Fang and Zhaopei Guo. *Email: thy@ciac.ac.cn; lyh2008@cust.edu.cn

Figures and Tables

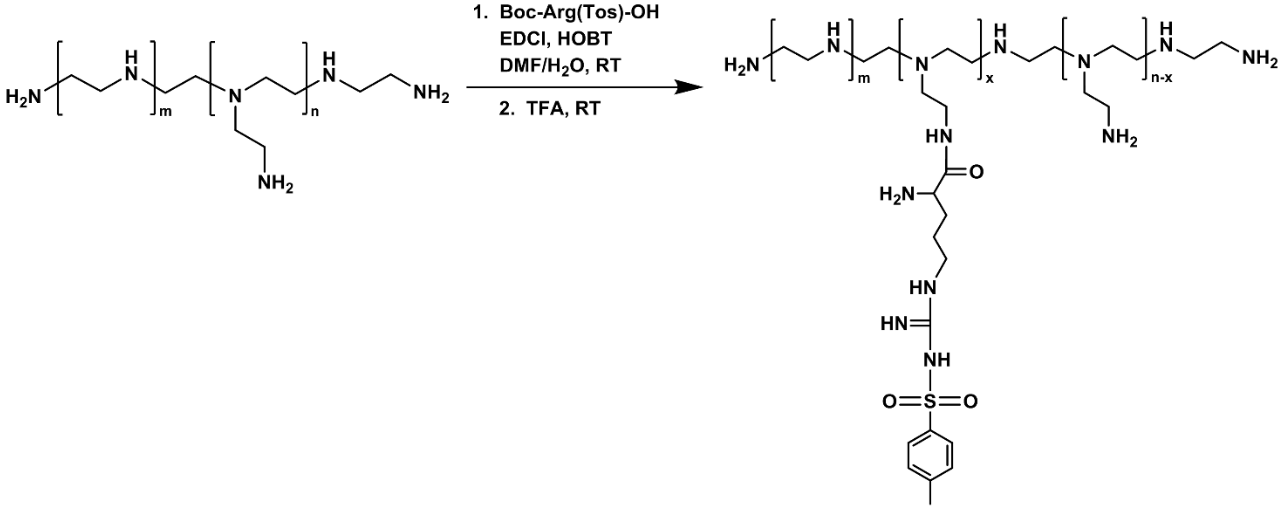

Supplementary Fig. 1 Synthesis route of PEI-RT.

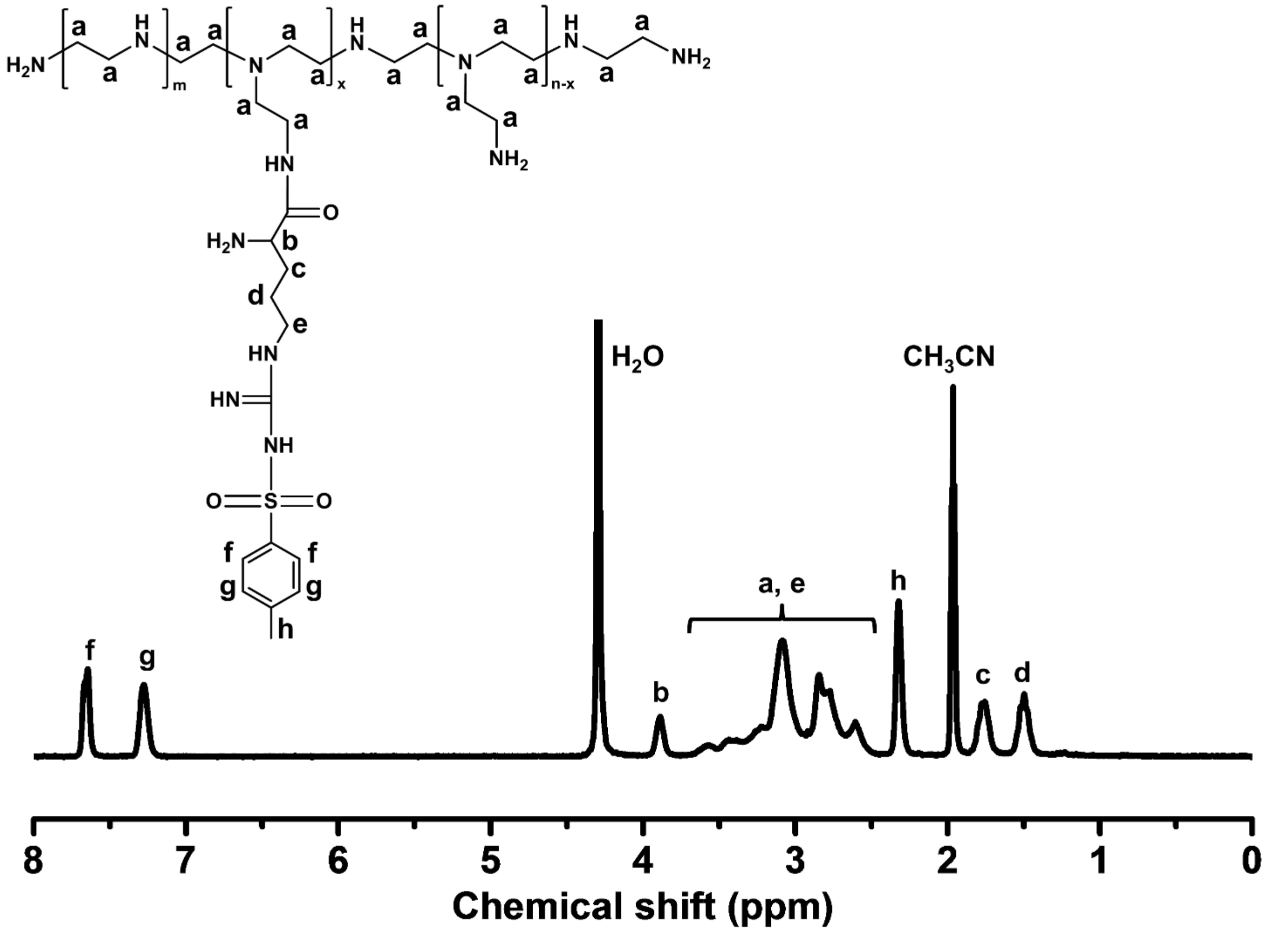

Supplementary Fig. 2 ^{1}H NMR spectrum of PEI-RT3 (400 MHz, D_2O/CD_3CN (v/v=1/1)).

Supplementary Table 1 The composition of PEI-RTs.

Polymer	Feed ratio (mole ratio) Boc-Arg(Tos)-OH/PEI1.8k	Grafted number of RT[a]
PEI-RT1	6:1	4.5
PEI-RT2	8:1	6.8
PEI-RT3	10:1	10.2
PEI-RT4	12:1	13.0

[a]Calculated based on ^{1}H NMR.

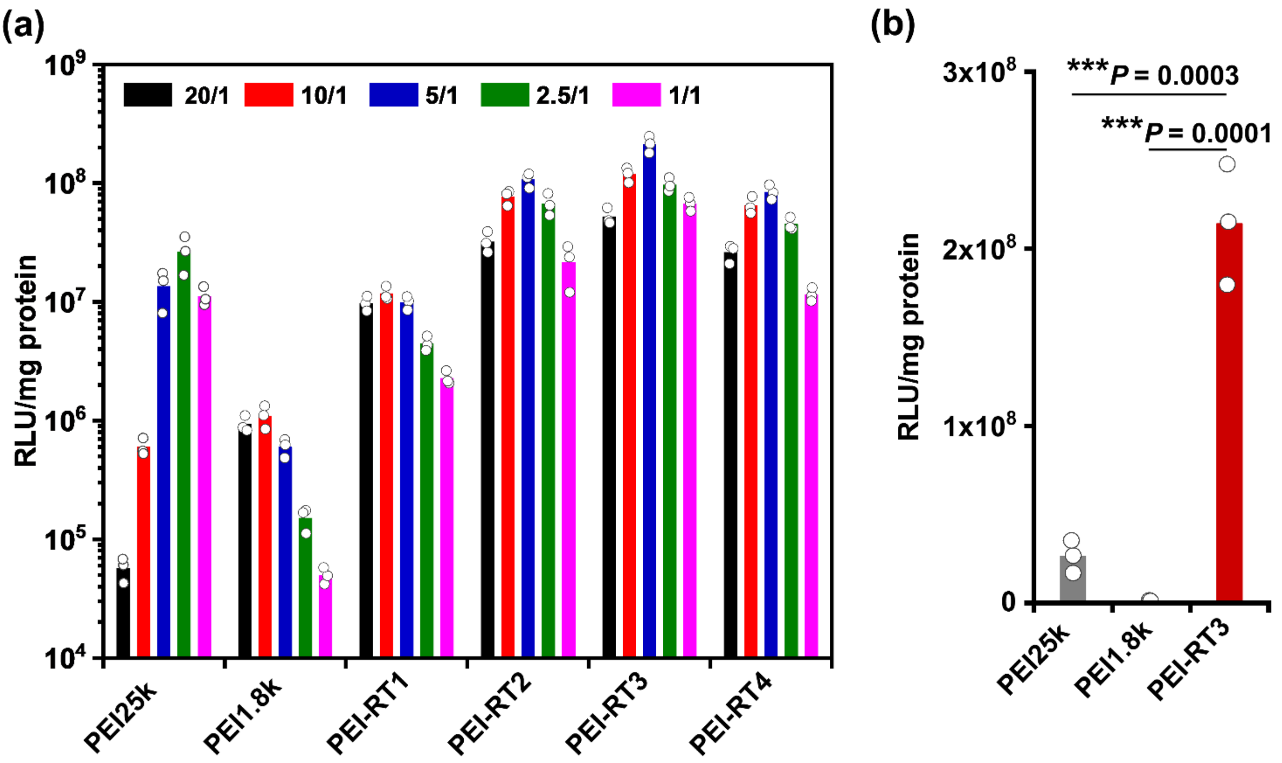

Supplementary Fig. 3 (a) DNA transfection of PEI-RTs containing different numbers of RT in B16F10 cells. Data are presented as mean ± SD (n=3). **(b)** The optimal transfection efficiency of PEI-RT3, PEI1.8k, and PEI25k in B16F10 cells. Data are presented as mean ± SD (n=3), ***P<0.001. Data in **Supplementary Fig. a** and **b** are presented as mean ± SD, n=3 biologically independent samples in **Supplementary Fig. a** and **b**. P values are calculated by the two-tailed student's t-test in **Supplementary Fig. b** as indicated in the figure, ***P<0.001.

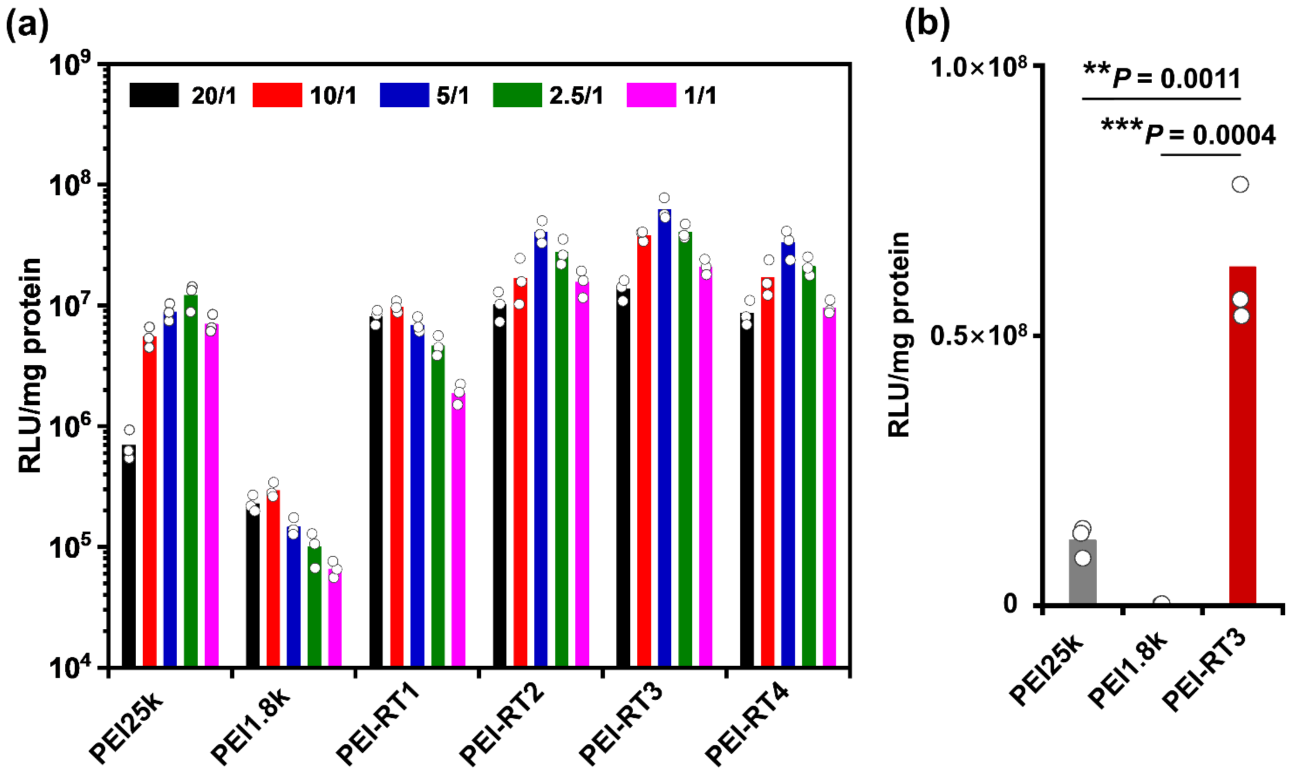

Supplementary Fig. 4 (a) DNA transfection of PEI-RTs containing different numbers of RT in 4T1 cells. **(b)** The optimal transfection efficiency of PEI-RT3, PEI1.8k, and PEI25k in 4T1 cells. Data in **Supplementary Fig. a** and **b** are presented as mean ± SD, n=3 biologically independent samples in **Supplementary Fig. a** and **b**. *P* values are calculated by the two-tailed student's t-test in **Supplementary Fig. b** as indicated in the figure, $^{**}P<0.01$ and $^{***}P<0.001$.

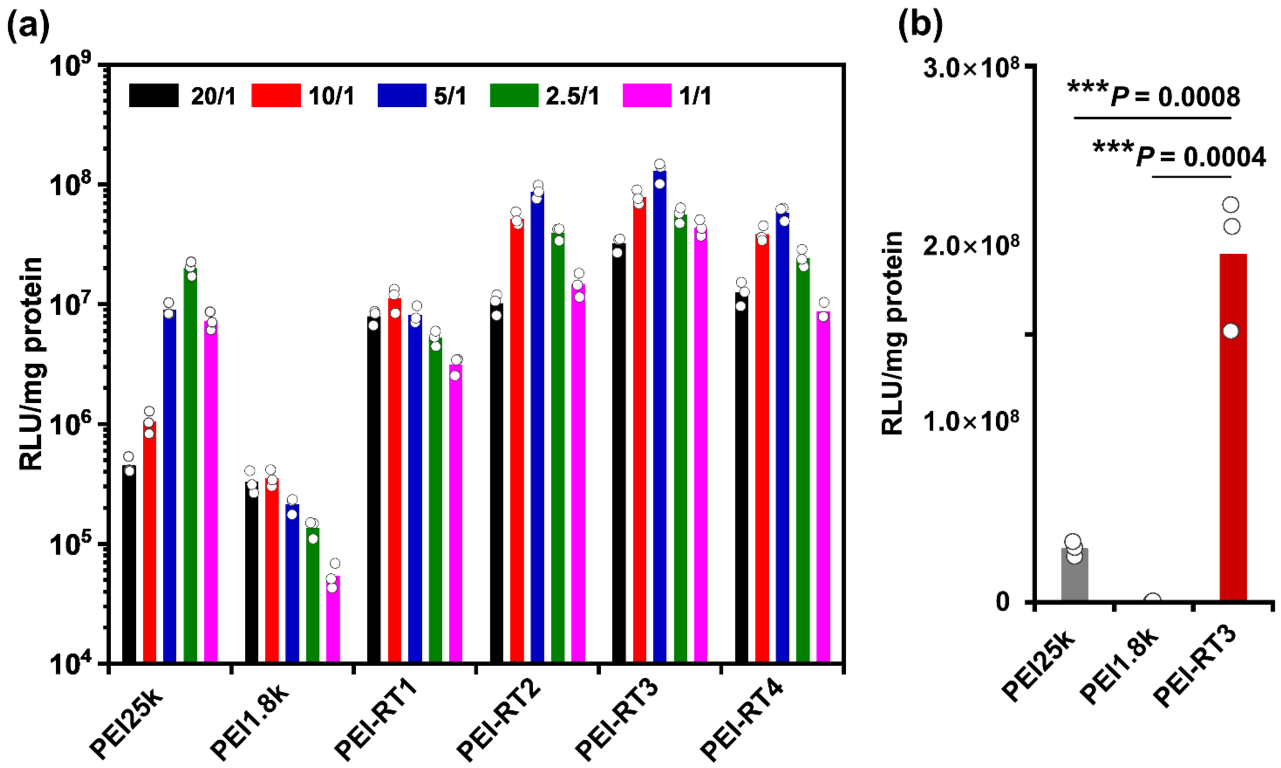

Supplementary Fig. 5 (a) DNA transfection of PEI-RTs containing different numbers of RT in HeLa cells. **(b)** The optimal transfection efficiency of PEI-RT3, PEI1.8k, and PEI25k in HeLa cells. Data in **Supplementary Fig. a** and **b** are presented as mean ± SD, n=3 biologically independent samples in **Supplementary Fig. a** and **b**. *P* values are calculated by the two-tailed student's t-test in **Supplementary Fig. b** as indicated in the figure, ****P*<0.001.

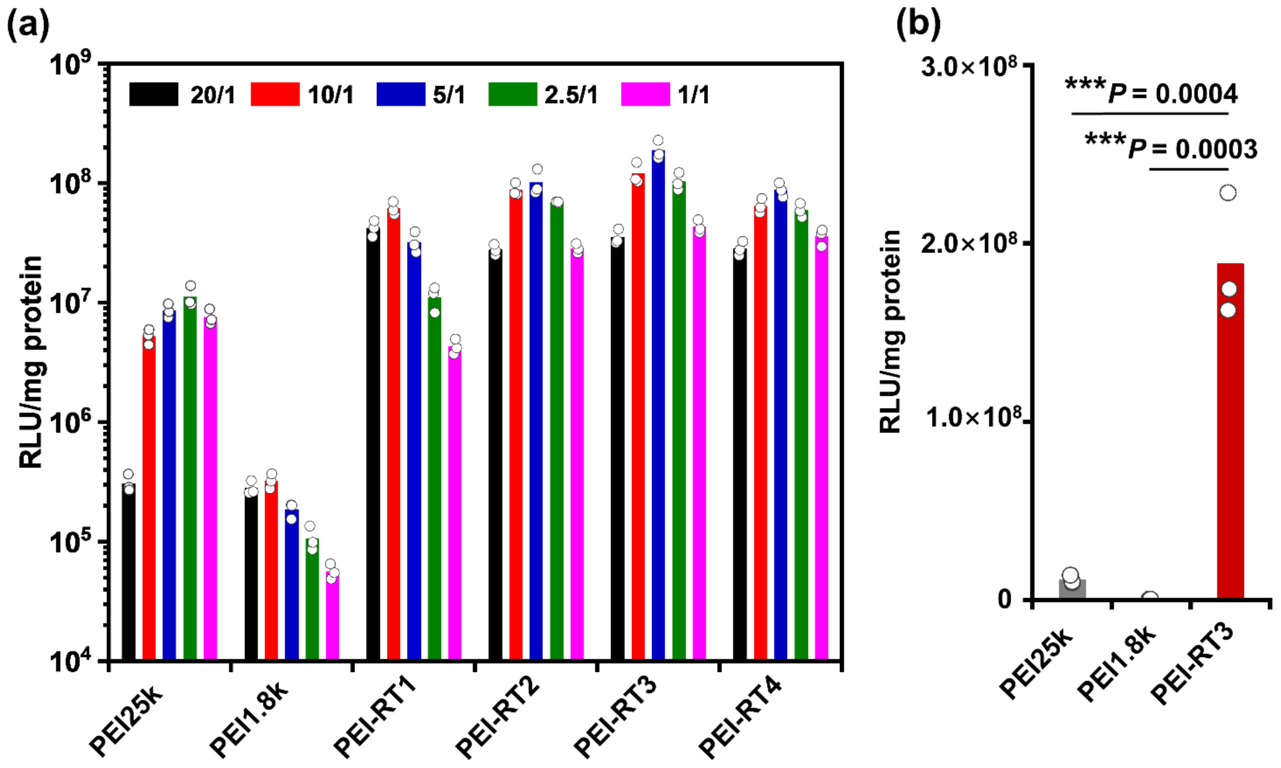

Supplementary Fig. 6 (a) DNA transfection of PEI-RTs containing different numbers of RT in MCF-7 cells. **(b)** The optimal transfection efficiency of PEI-RT3, PEI1.8k, and PEI25k in MCF-7 cells. Data in **Supplementary Fig. a** and **b** are presented as mean ± SD, n=3 biologically independent samples in **Supplementary Fig. a** and **b**. *P* values are calculated by the two-tailed student's t-test in **Supplementary Fig. b** as indicated in the figure, ****P*<0.001.

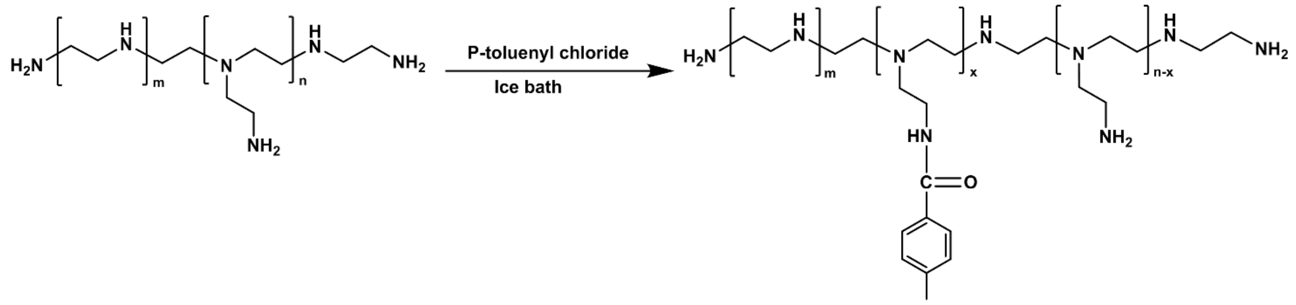

Supplementary Fig. 7 Synthesis route of PEI-Too.

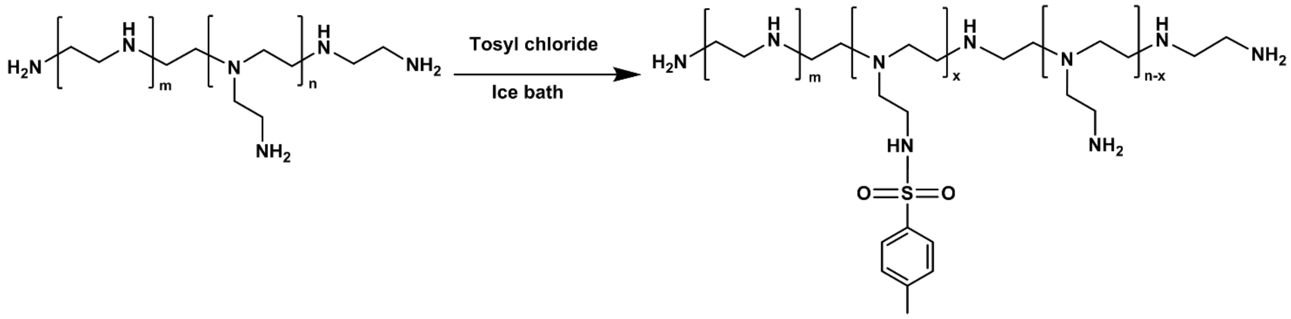

Supplementary Fig. 8 Synthesis route of PEI-Tos.

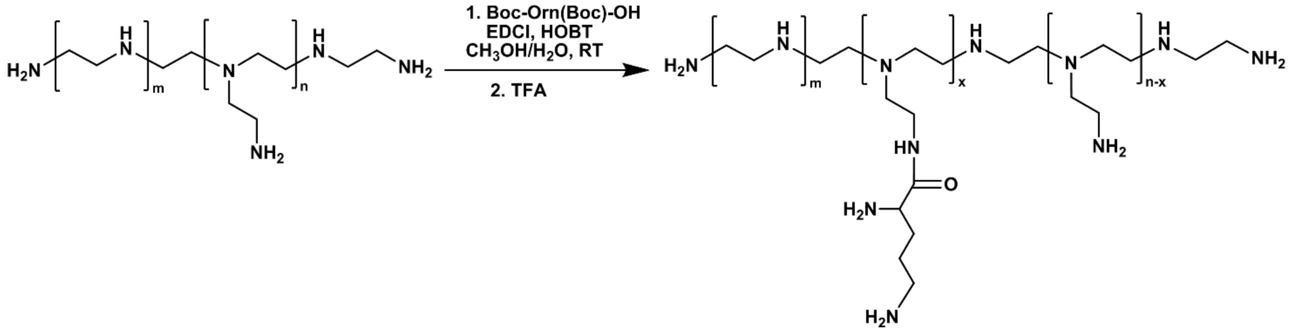

Supplementary Fig. 9 Synthesis route of PEI-Orn.

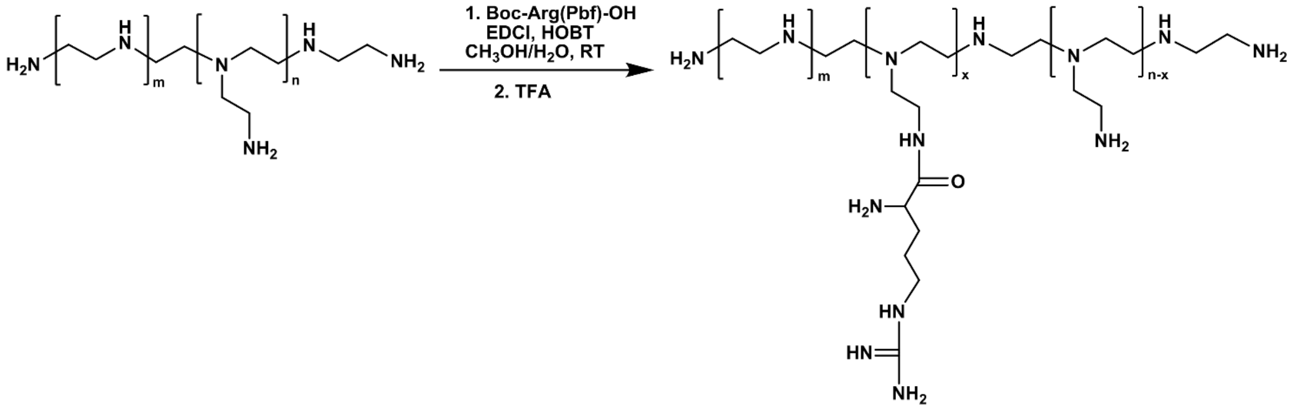

Supplementary Fig. 10 Synthesis route of PEI-Arg.

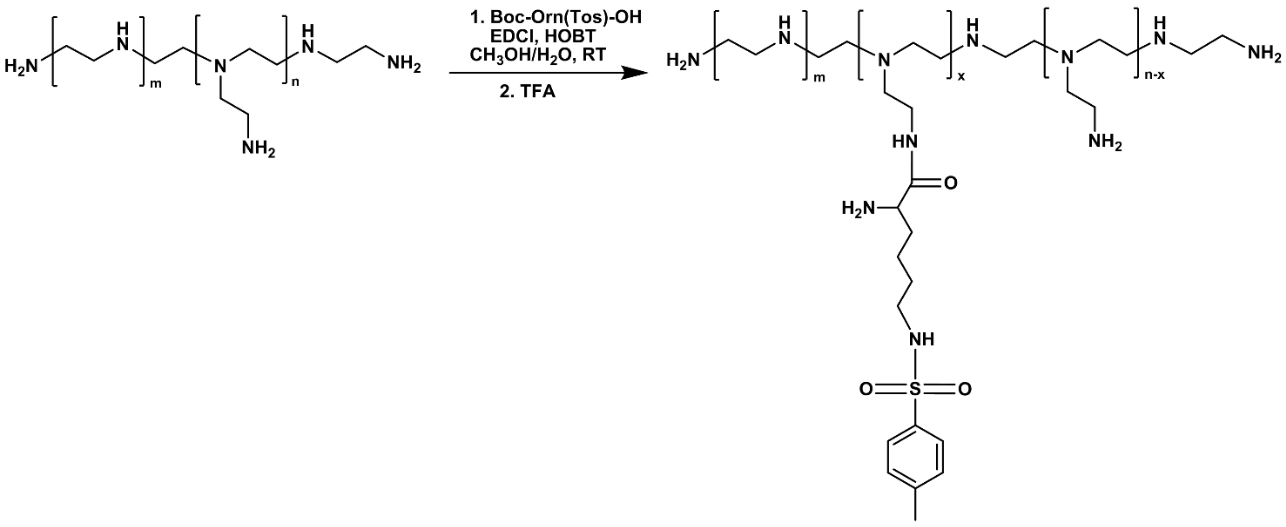

Supplementary Fig. 11 Synthesis route of PEI-Orn(Tos).

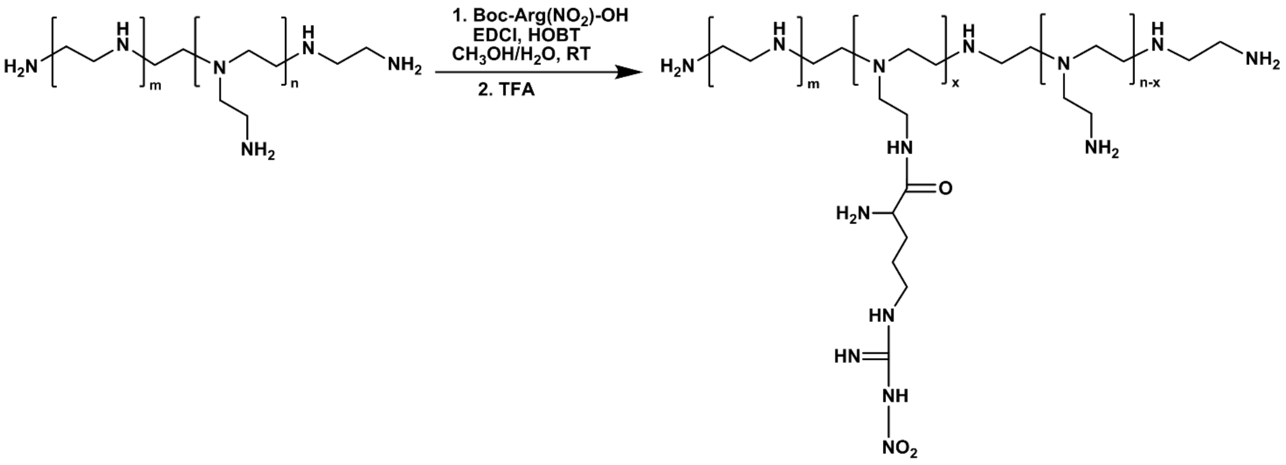

Supplementary Fig. 12 Synthesis route of PEI-Arg(NO_2).

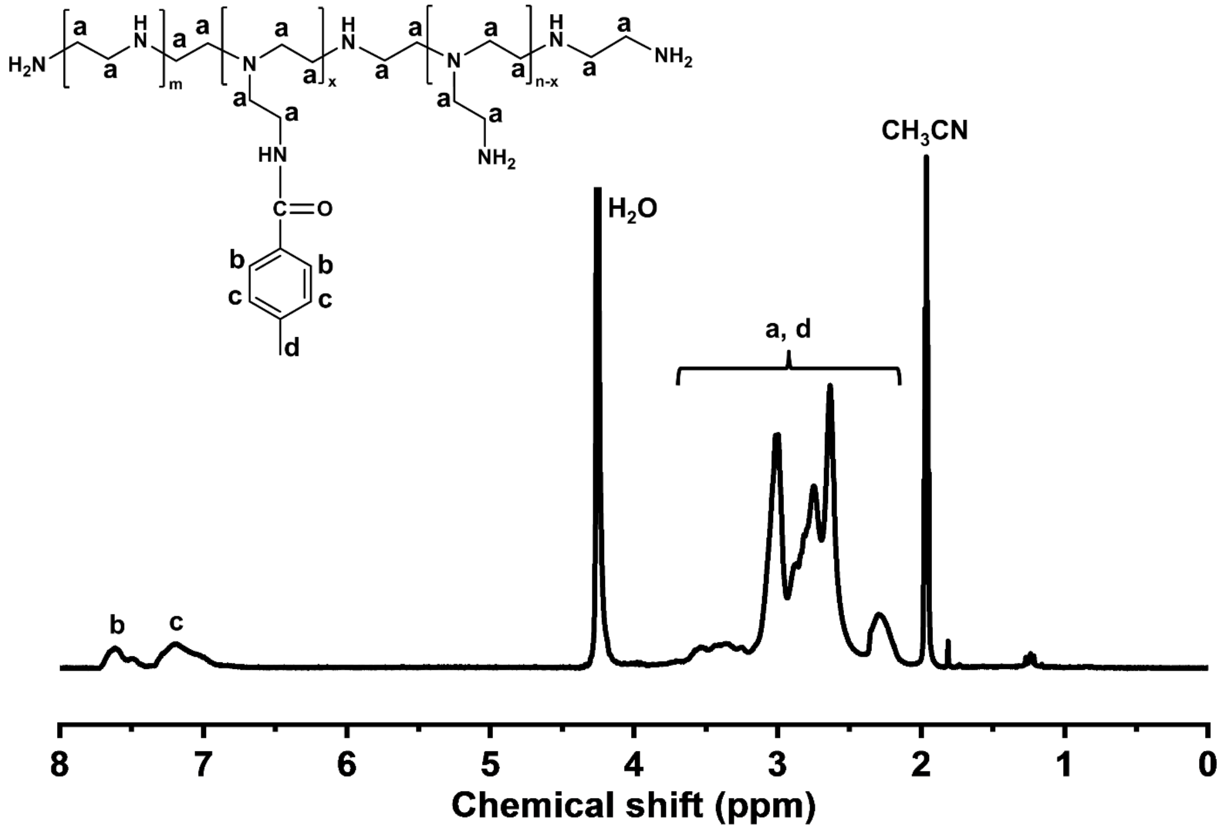

Supplementary Fig. 13 ^{1}H NMR spectrum of PEI-Too (400 MHz, D_2O/CD_3CN (v/v=1/1)).

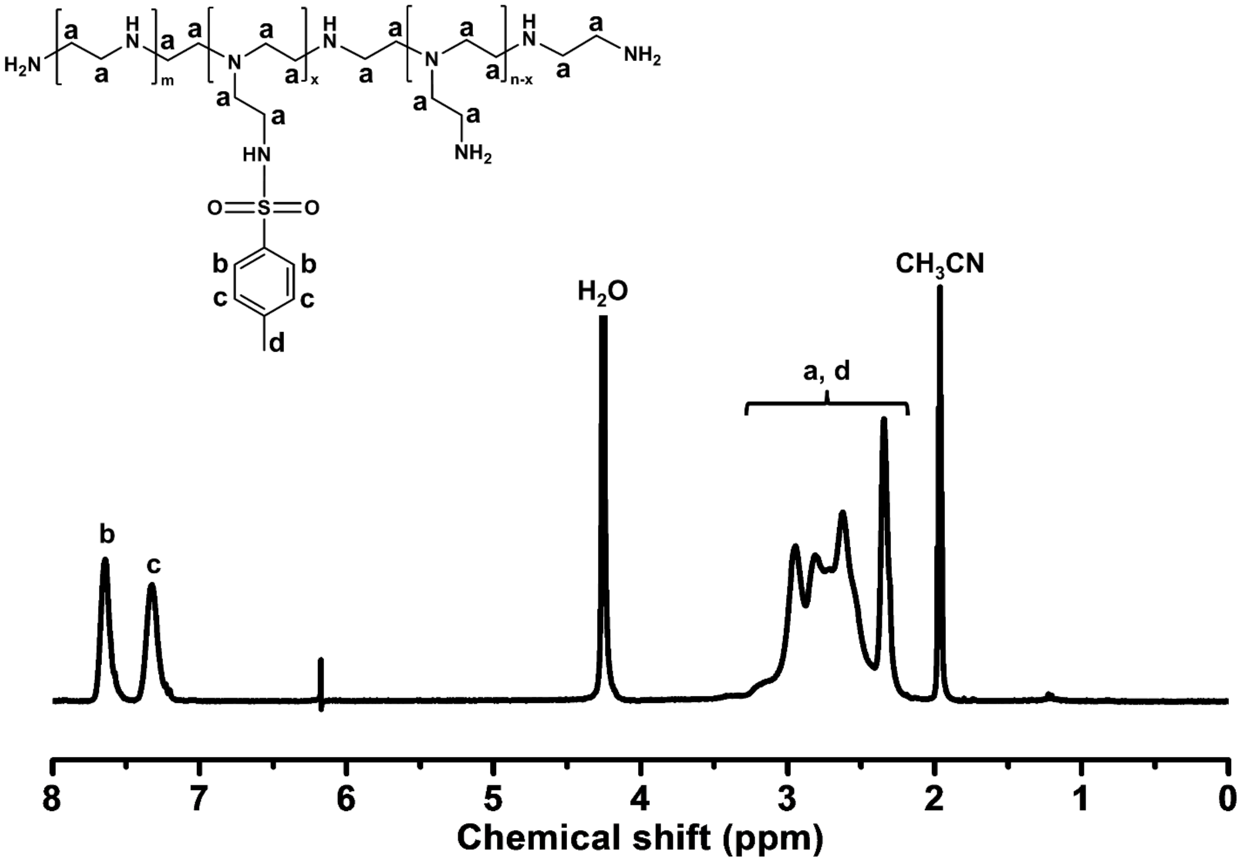

Supplementary Fig. 14 ^{1}H NMR spectrum of PEI-Tos (400 MHz, D_2O/CD_3CN (v/v=1/1)).

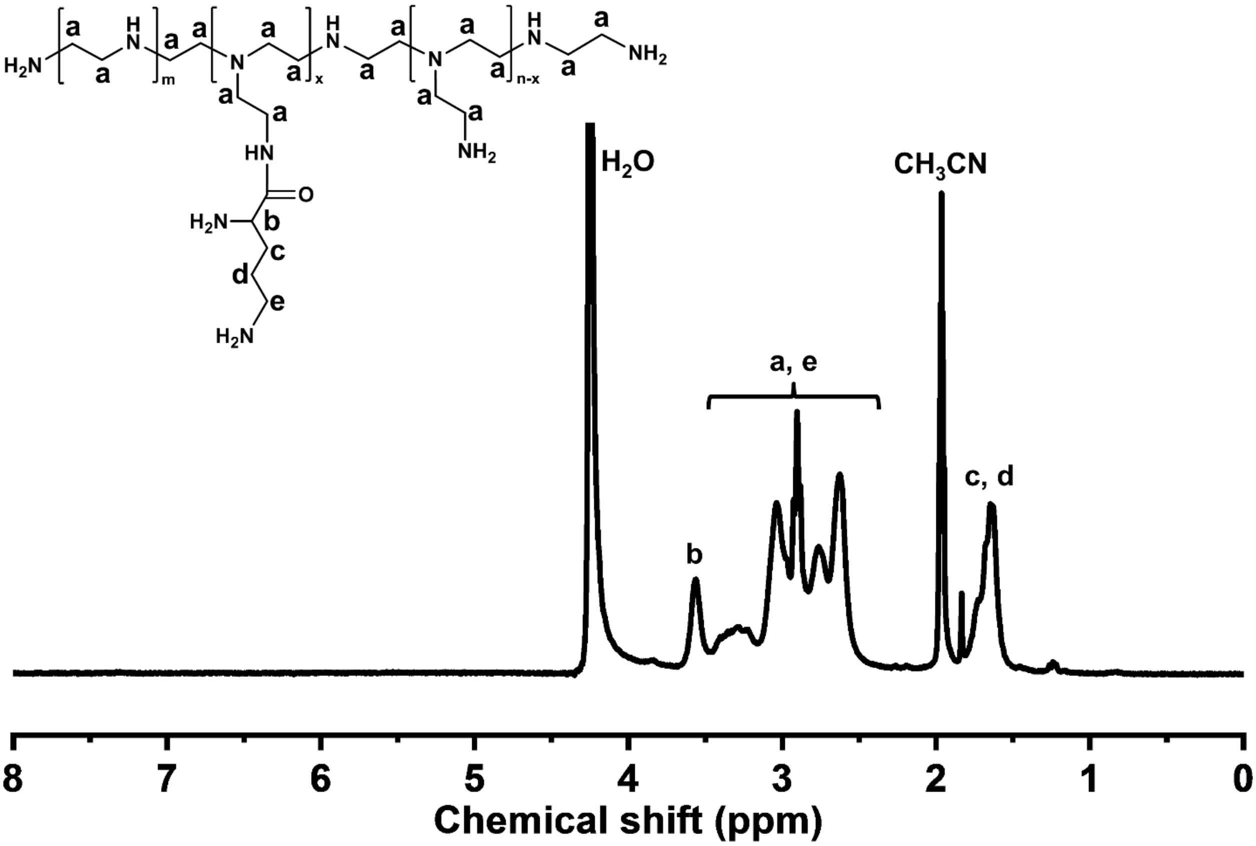

Supplementary Fig. 15 ^{1}H NMR spectrum of PEI-Orn (400 MHz, D_2O/CD_3CN (v/v=1/1)).

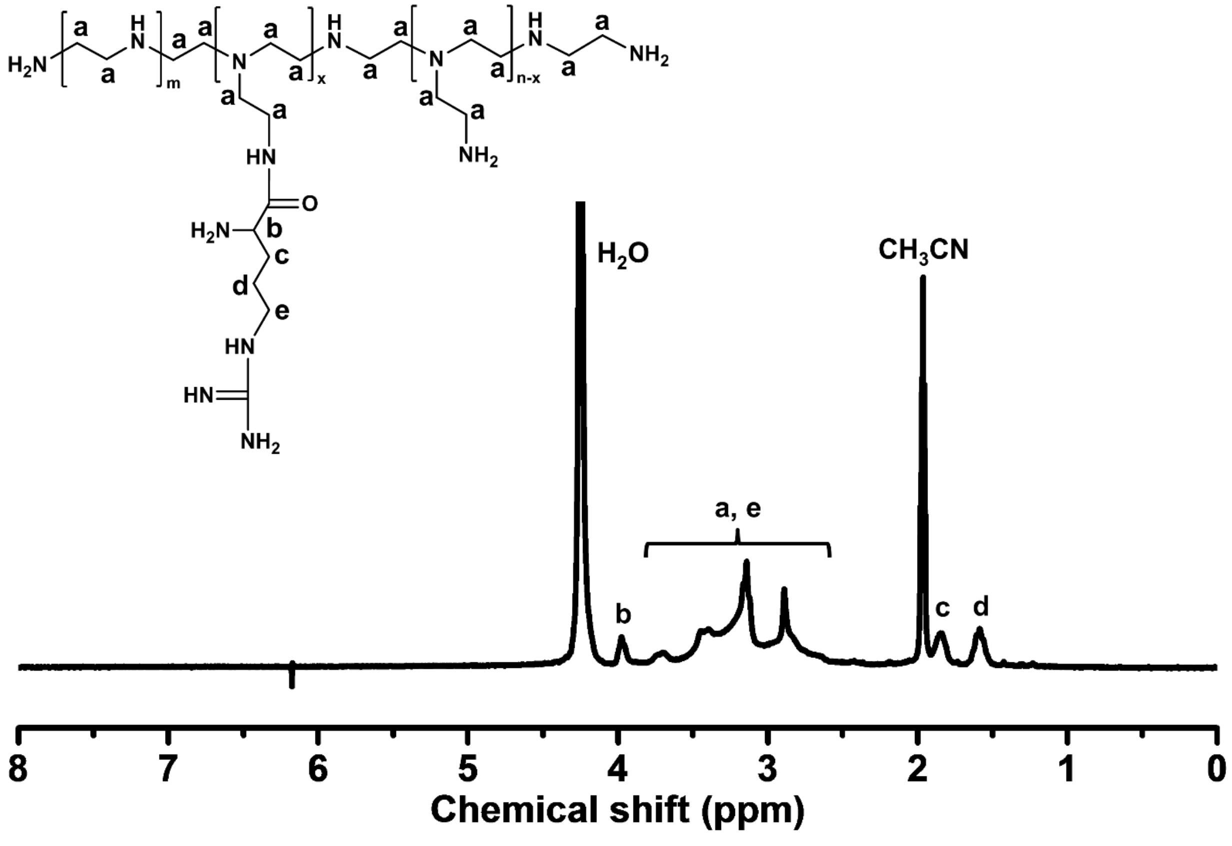

Supplementary Fig. 16 ^{1}H NMR spectrum of PEI-Arg (400 MHz, D_2O/CD_3CN (v/v=1/1)).

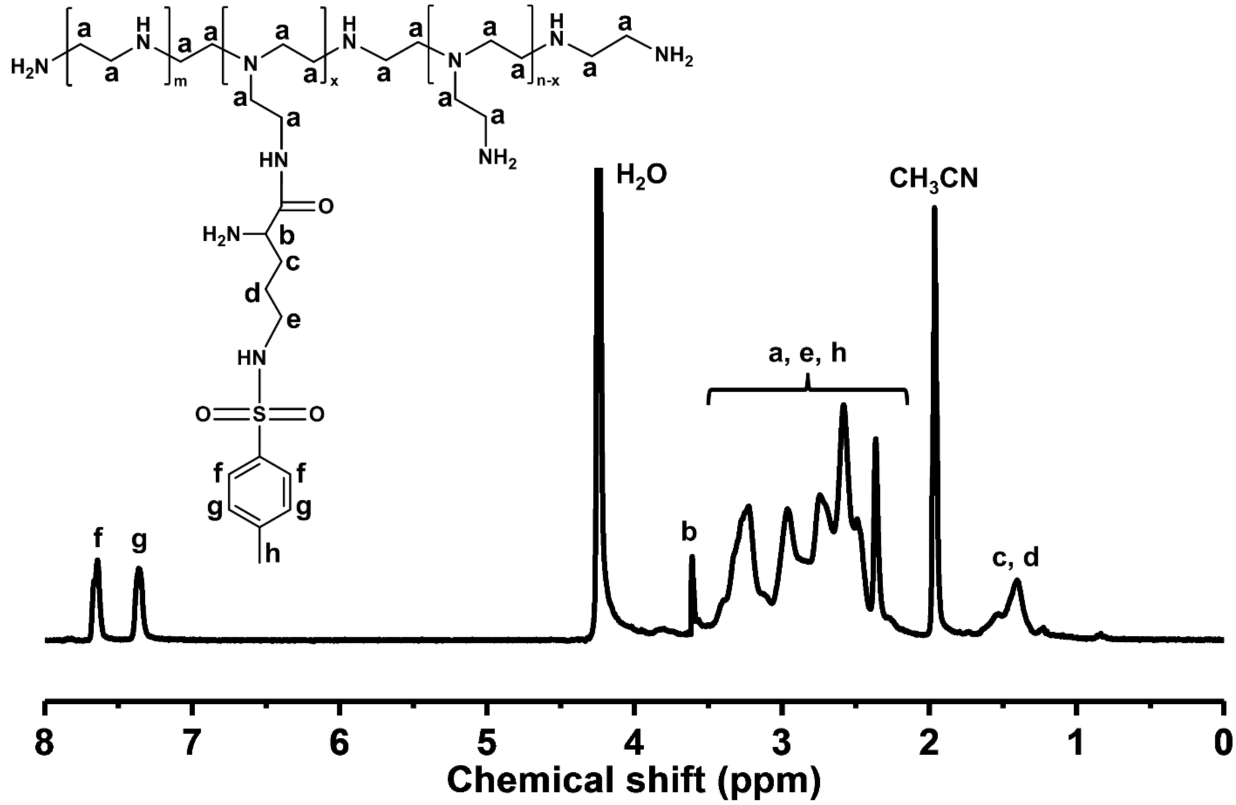

Supplementary Fig. 17 ^{1}H NMR spectrum of PEI-Orn(Tos) (400 MHz, D_2O/CD_3CN (v/v=1/1)).

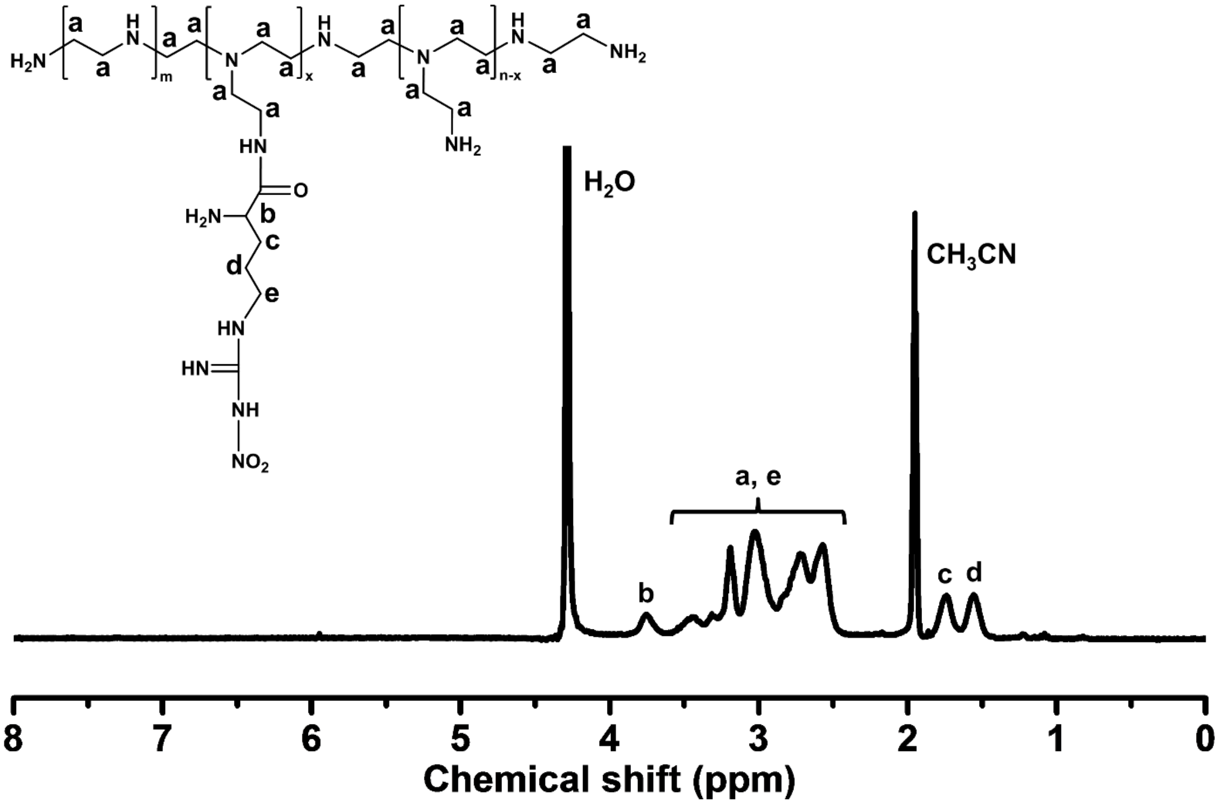

Supplementary Fig. 18 ^{1}H NMR spectrum of PEI-Arg(NO_2) (400 MHz, D_2O/CD_3CN (v/v=1/1)).

Supplementary Table 2 Different types of molecular strings grafted onto PEI1.8k and the corresponding grafting number.

Polymer	Feed ratio (mole ratio) R/PEI1.8k	Grafted number of R[a]
PEI-Too	10:1	9.8
PEI-Tos	10:1	10.2
PEI-Orn	10:1	10.6
PEI-Arg	10:1	10.4
PEI-Orn(Tos)	10:1	10.7
PEI-Arg(NO_2)	10:1	10.8

[a]Calculated based on ^{1}H NMR.

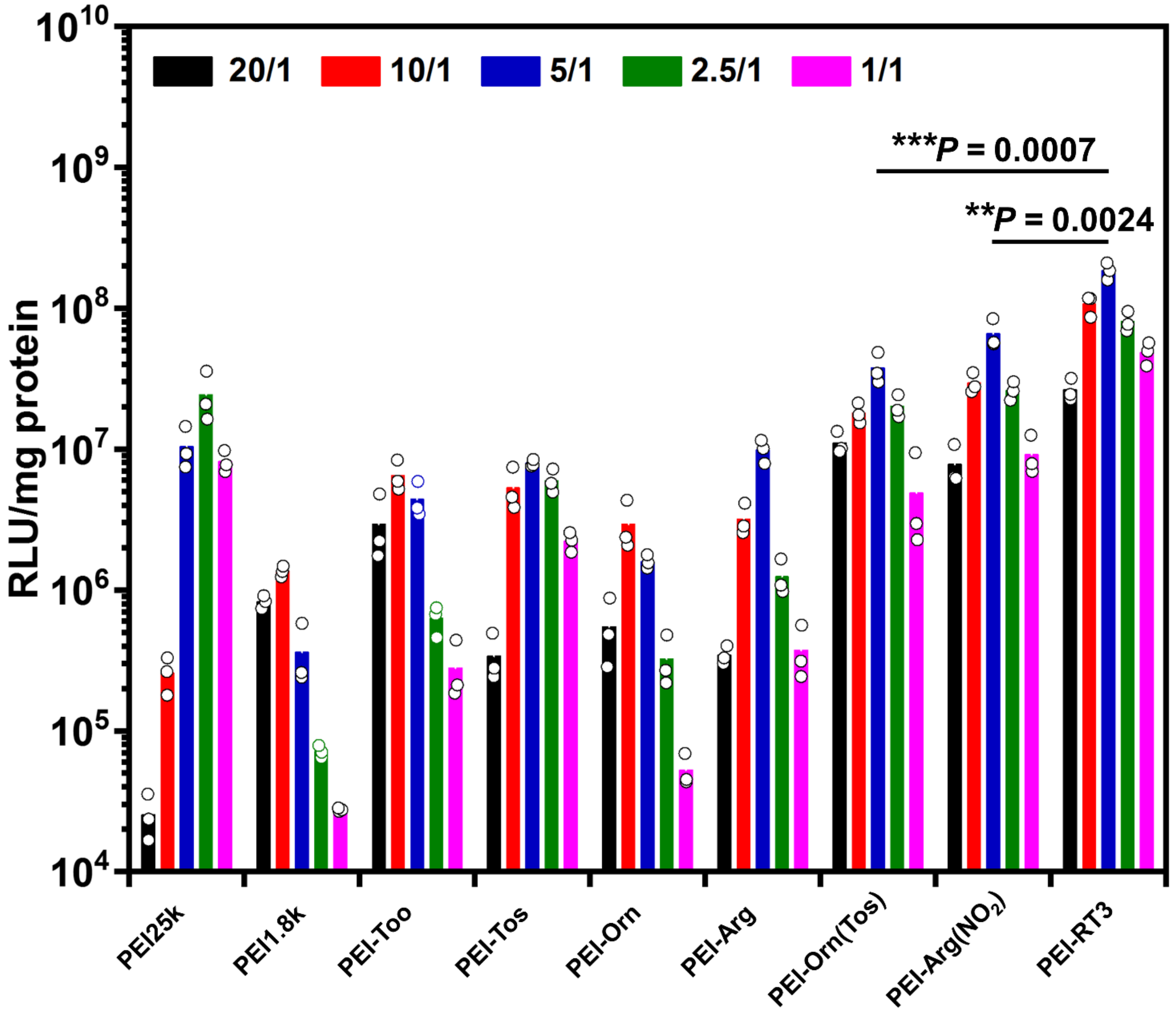

Supplementary Fig. 19 DNA transfection of PEI1.8k grafting with various molecular strings at different mass ratios of carrier/DNA in B16F10 cells. Data are presented as mean ± SD, n=3 biologically independent samples. *P* values are calculated by the two-tailed student's t-test as indicated in the figure, $^{**}P<0.01$ and $^{***}P<0.001$.

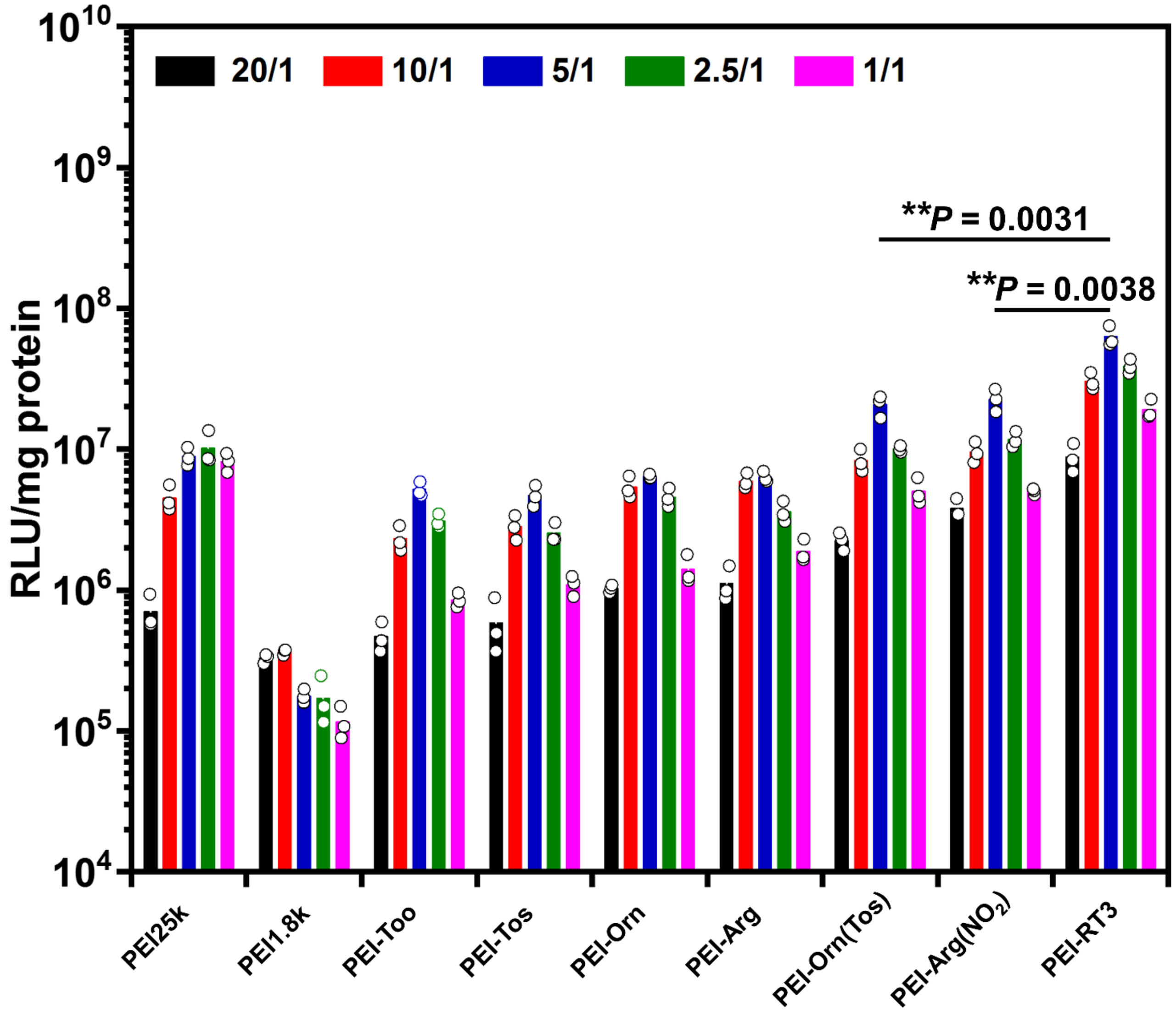

Supplementary Fig. 20 DNA transfection of PEI1.8k grafting with various molecular strings at different mass ratios of carrier/DNA in 4T1 cells. Data are presented as mean ± SD, n=3 biologically independent samples. *P* values are calculated by the two-tailed student's t-test as indicated in the figure, $^{**}P<0.01$.

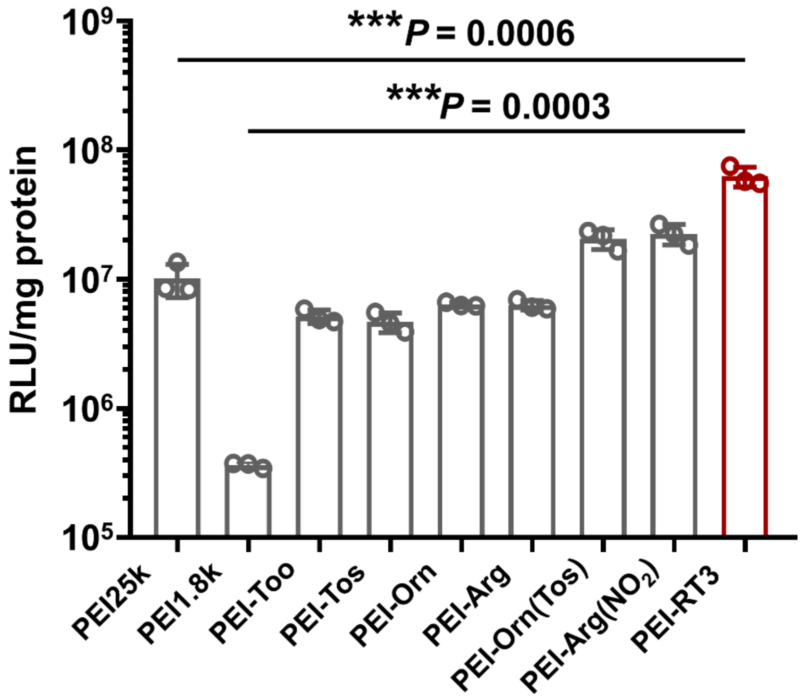

Supplementary Fig. 21 Optimal DNA transfection efficiency of PEI1.8k grafting with various molecular strings in 4T1 cells. Data are presented as mean ± SD, n=3 biologically independent samples. *P* values are calculated by the two-tailed student's t-test as indicated in the figure, ***P<0.001.

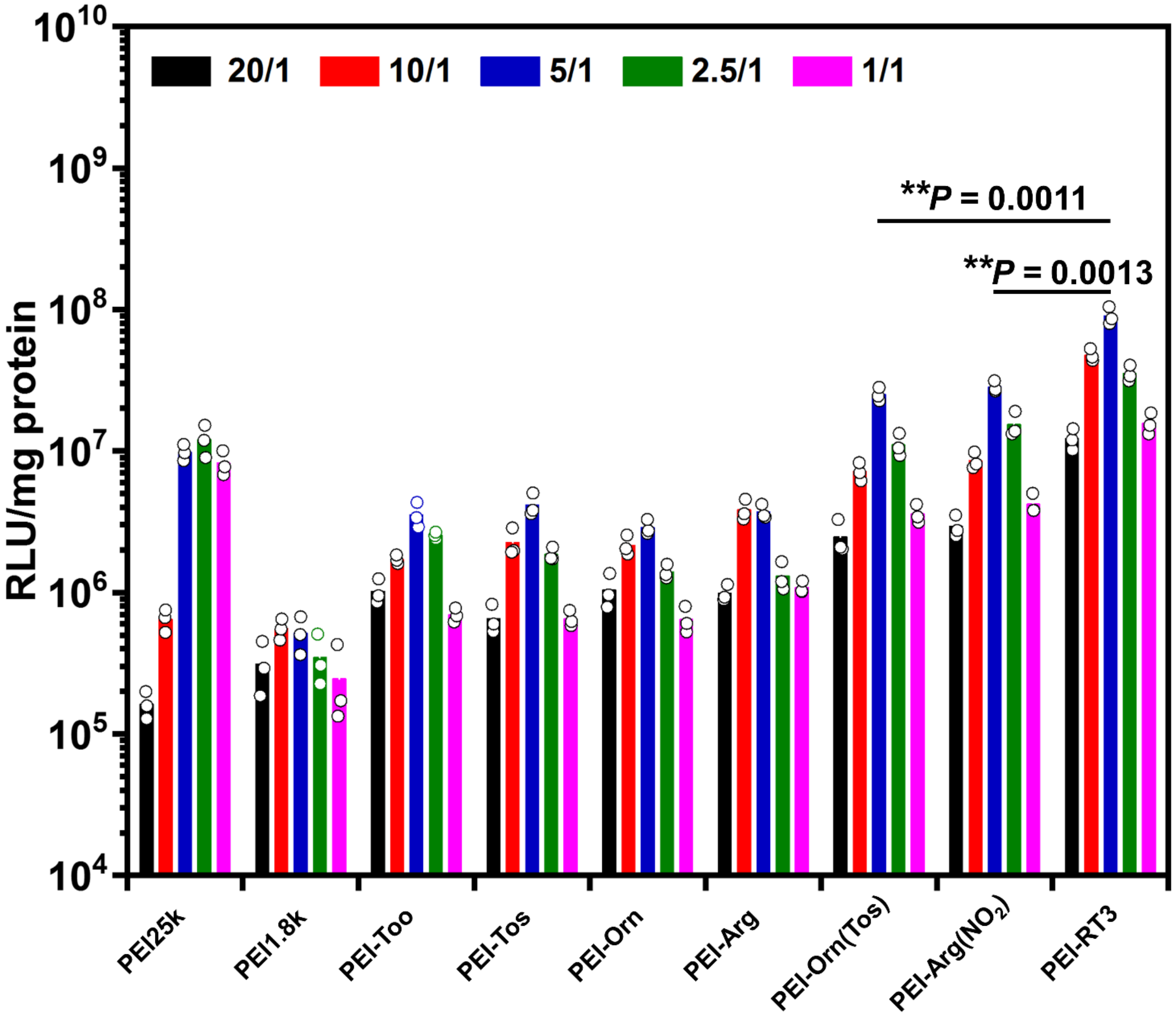

Supplementary Fig. 22 DNA transfection of PEI1.8k grafting with various molecular strings at different mass ratios of carrier/DNA in HeLa cells. Data are presented as mean ± SD, n=3 biologically independent samples. *P* values are calculated by the two-tailed student's t-test as indicated in the figure, $^{**}P<0.01$.

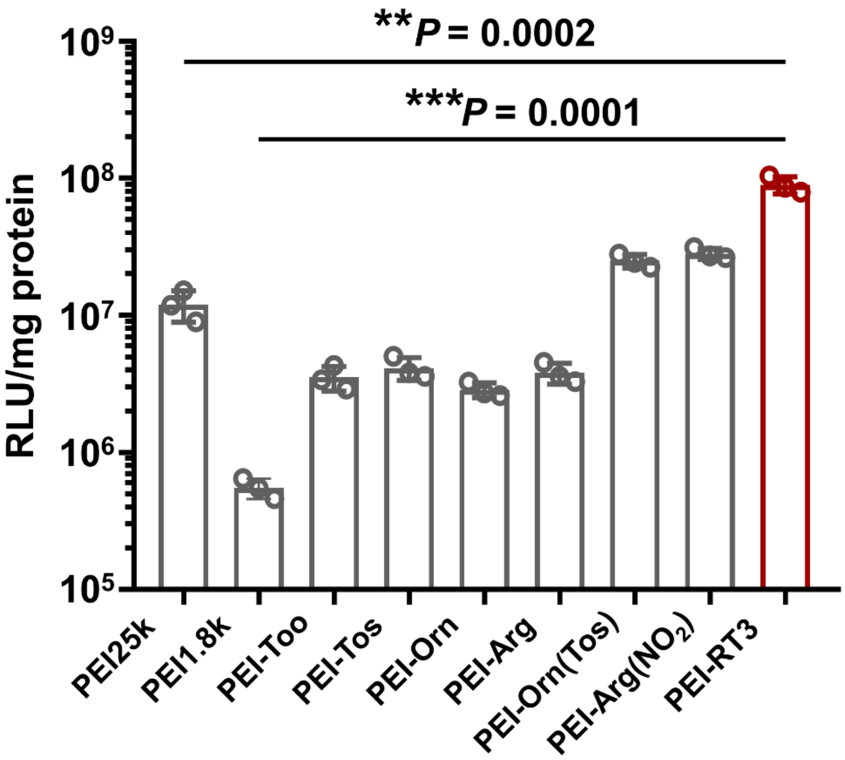

Supplementary Fig. 23 Optimal DNA transfection efficiency of PEI1.8k grafting with various molecular strings in HeLa cells. Data are presented as mean ± SD, n=3 biologically independent samples. *P* values are calculated by the two-tailed student's t-test as indicated in the figure, $^{**}P<0.01$ and $^{***}P<0.001$.

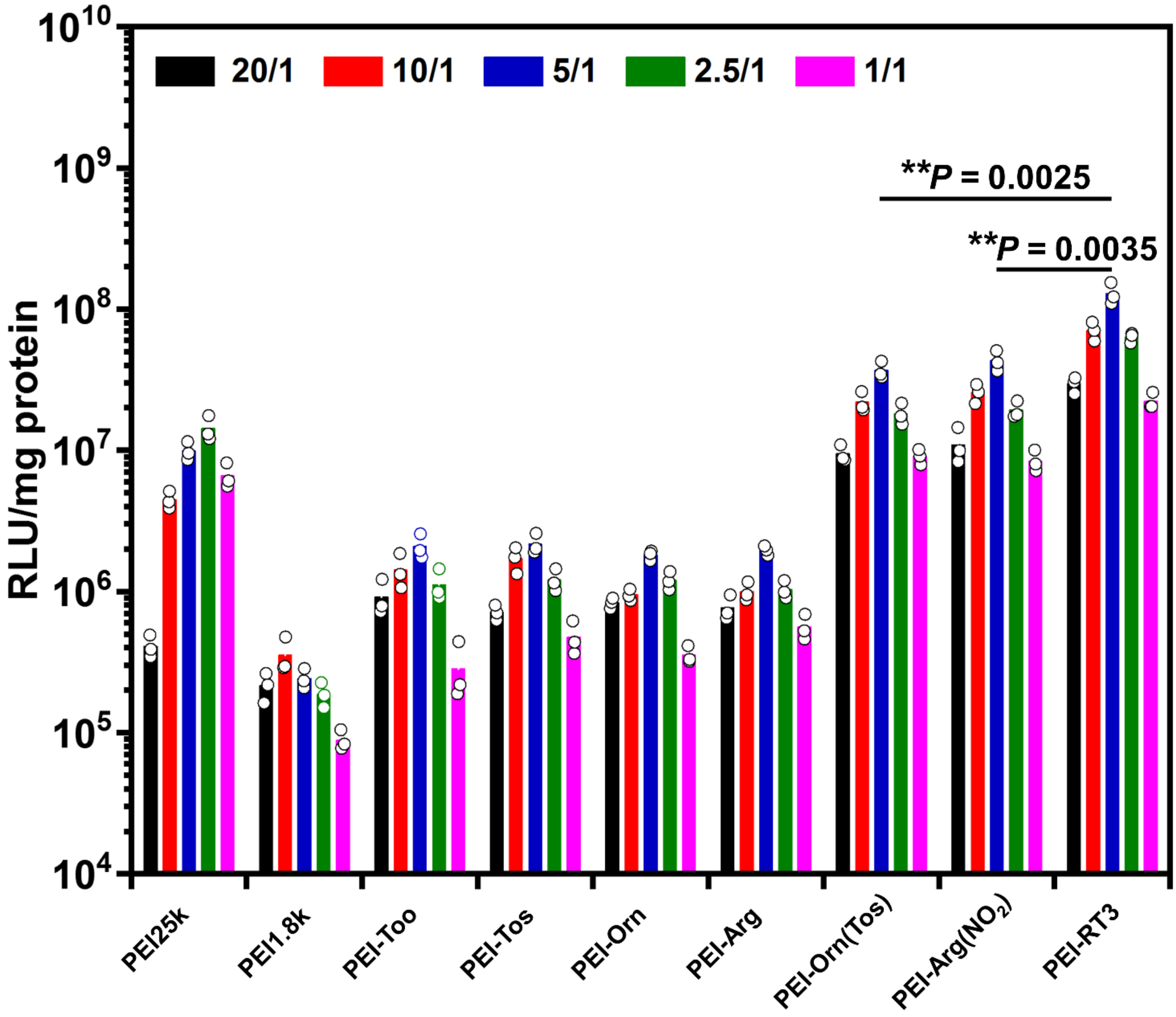

Supplementary Fig. 24 DNA transfection of PEI1.8k grafting with various molecular strings at different mass ratios of carrier/DNA in MCF-7 cells. Data are presented as mean ± SD (n=3). Data are presented as mean ± SD, n=3 biologically independent samples. *P* values are calculated by the two-tailed student's t-test as indicated in the figure, $^{**}P<0.01$.

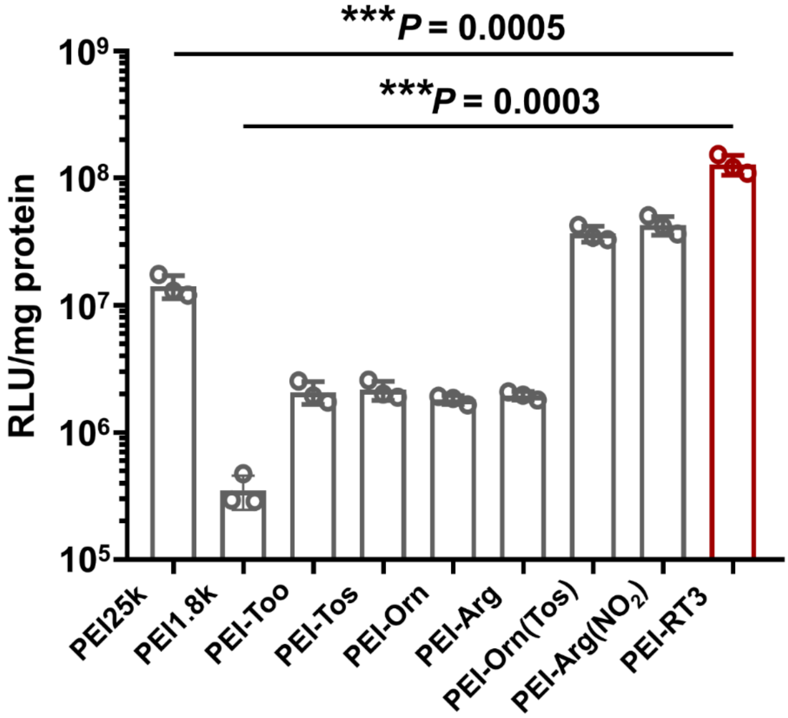

Supplementary Fig. 25 Optimal DNA transfection efficiency of PEI1.8k grafting with various molecular strings in MCF-7 cells. Data are presented as mean ± SD, n=3 biologically independent samples. *P* values are calculated by the two-tailed student's t-test as indicated in the figure, ****P*<0.001.

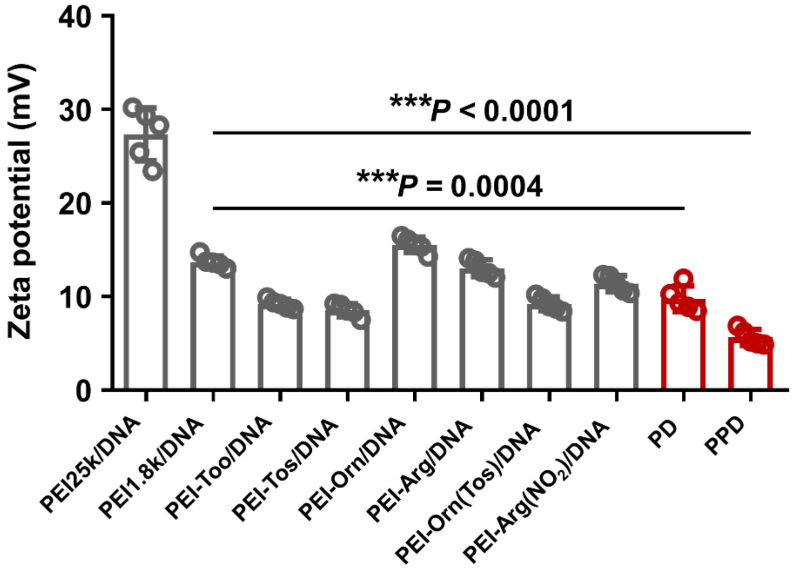

Supplementary Fig. 26 Zeta potential of polycation/DNA complexes at mass ratio of 5/1. Data are presented as mean ± SD, n=5 biologically independent samples. *P* values are calculated by the two-tailed student's t-test as indicated in the figure, ****P*<0.001.

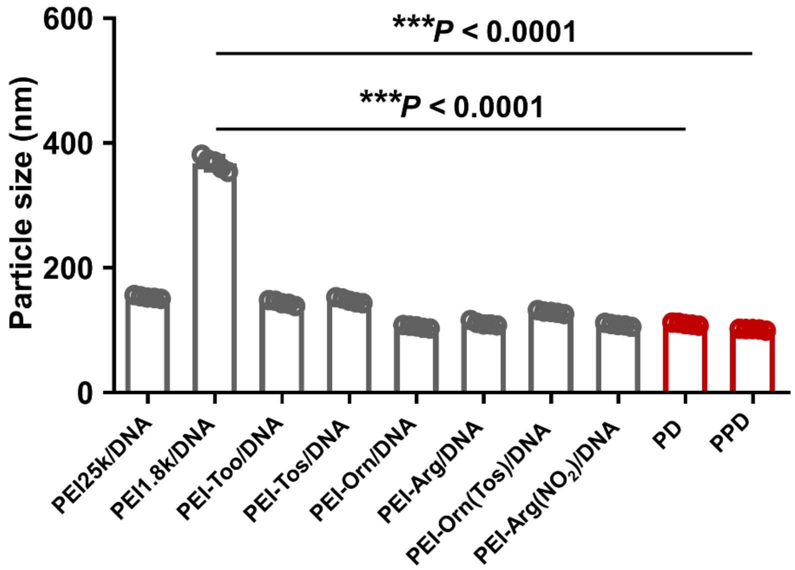

Supplementary Fig. 27 Particle size of polycation/DNA complexes at mass ratio of 5/1. Data are presented as mean ± SD (n=5). Data are presented as mean ± SD, n=5 biologically independent samples. *P* values are calculated by the two-tailed student's t-test as indicated in the figure, ***P<0.001.

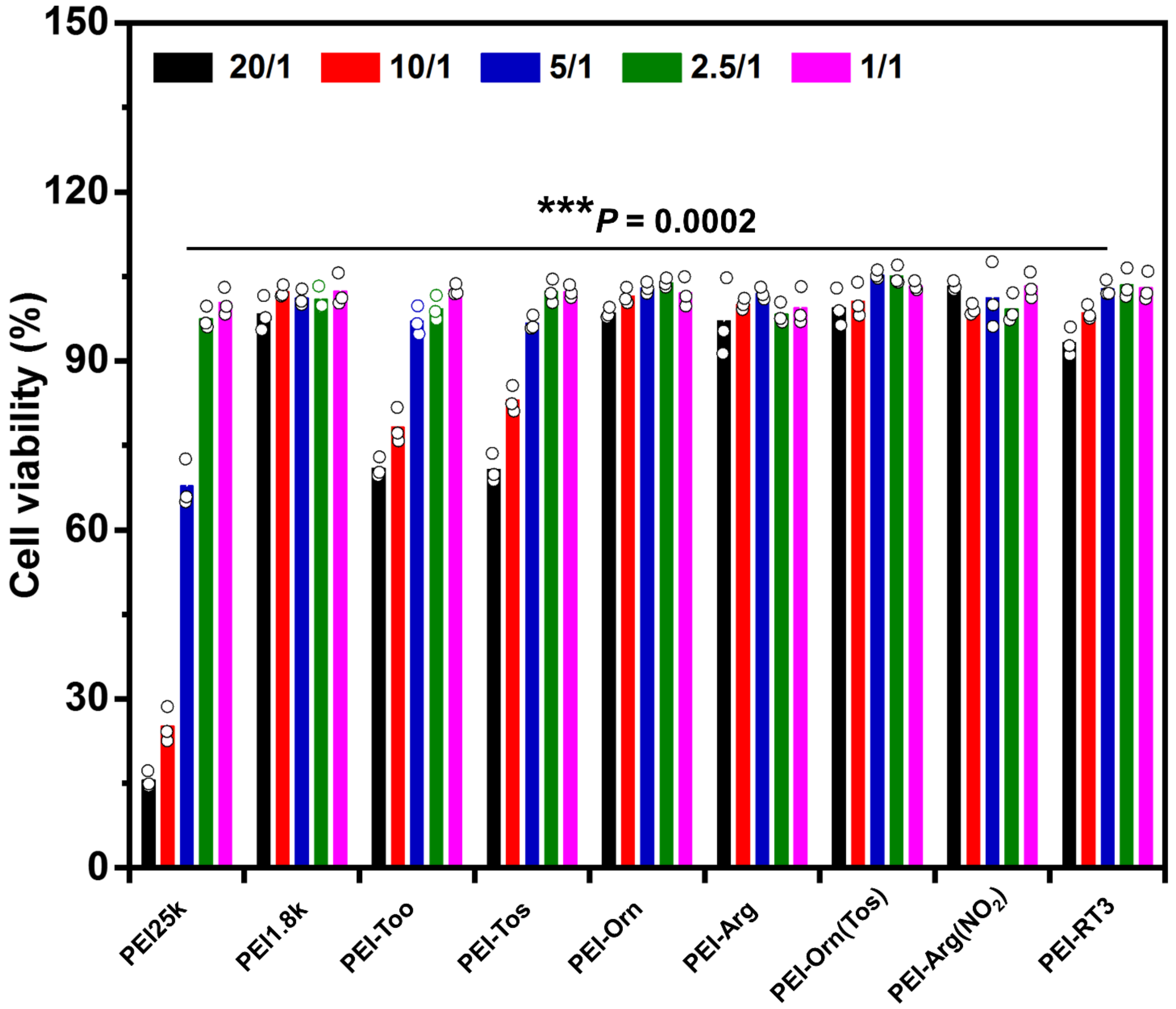

Supplementary Fig. 28 The cell viability of PEI-RT3/DNA and other polycation/DNA complexes at different mass ratios in B16F10 cells. Data are presented as mean ± SD, n=3 biologically independent samples. *P* values are calculated by the two-tailed student's t-test as indicated in the figure, ****P*<0.001.

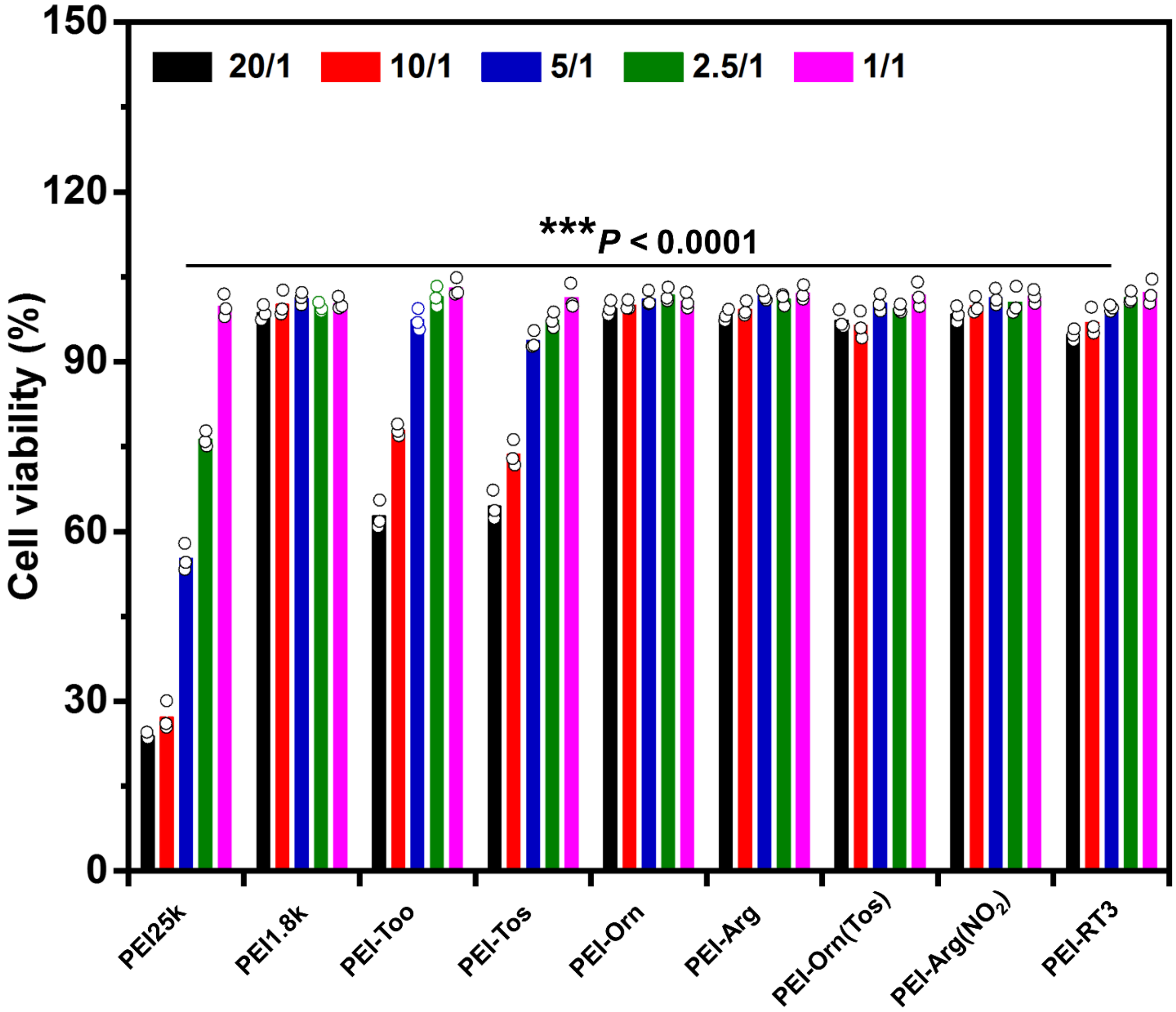

Supplementary Fig. 29 The cell viability of PEI-RT3/DNA and other polycation/DNA complexes at different mass ratios in 4T1 cells. Data are presented as mean ± SD, n=3 biologically independent samples. *P* values are calculated by the two-tailed student's t-test as indicated in the figure, ***P<0.001.

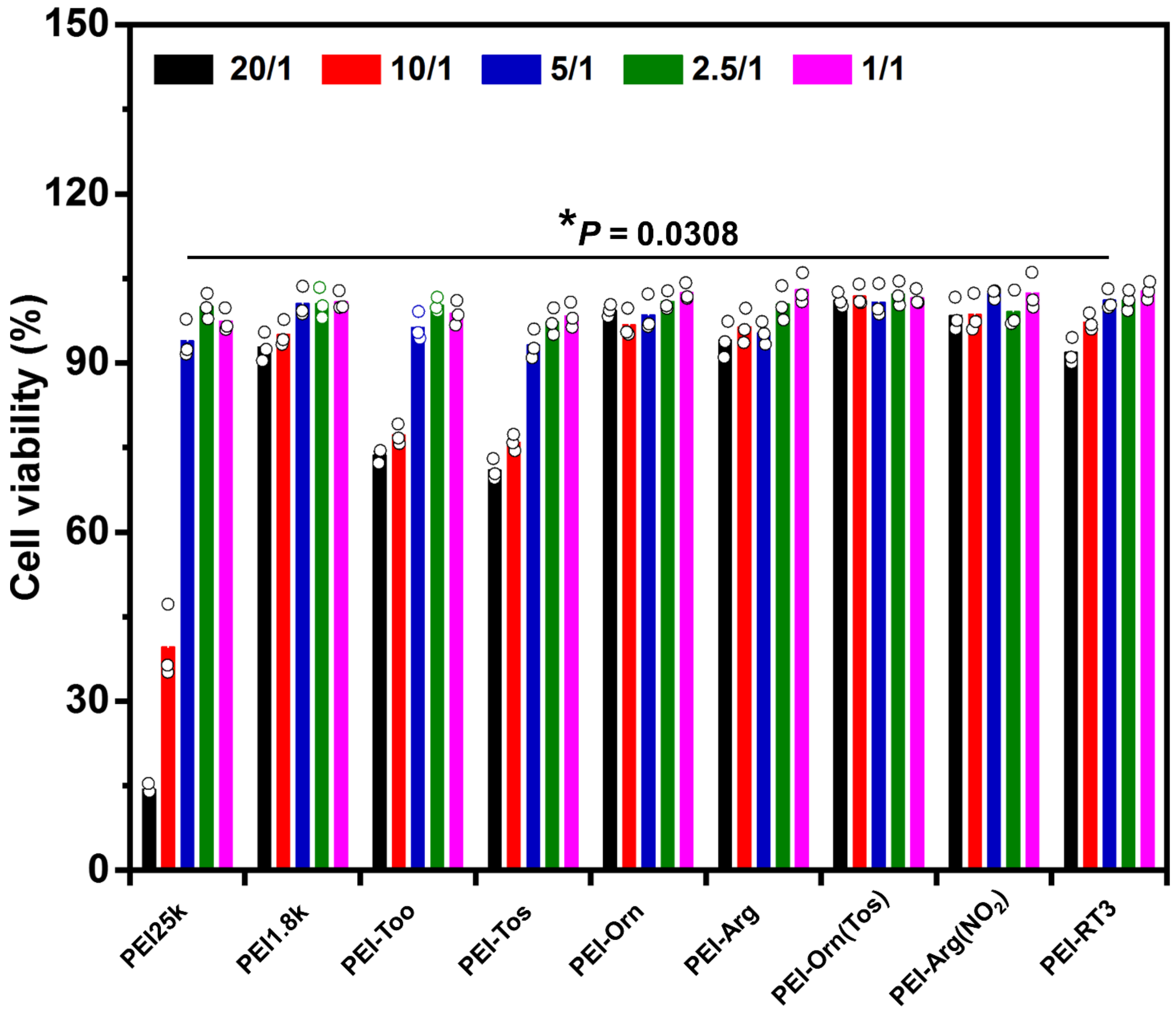

Supplementary Fig. 30 The cell viability of PEI-RT3/DNA and other polycation/DNA complexes at different mass ratios in HeLa cells. Data are presented as mean ± SD, n=3 biologically independent samples. *P* values are calculated by the two-tailed student's t-test as indicated in the figure, $*P<0.05$.

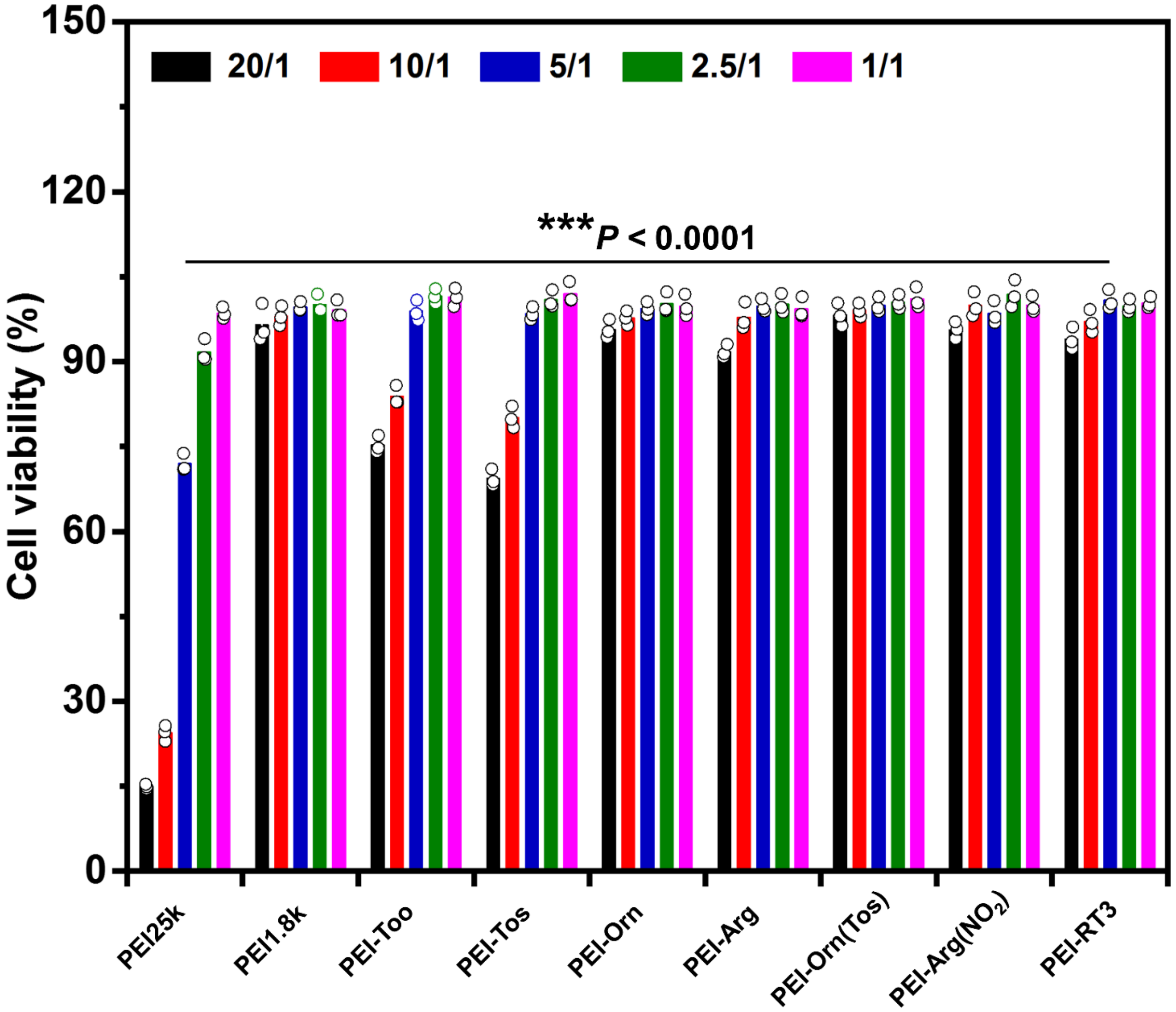

Supplementary Fig. 31 The cell viability of PEI-RT3/DNA and other polycation/DNA complexes at different mass ratios in MCF-7 cells. Data are presented as mean ± SD, n=3 biologically independent samples. *P* values are calculated by the two-tailed student's t-test as indicated in the figure, ****P*<0.001.

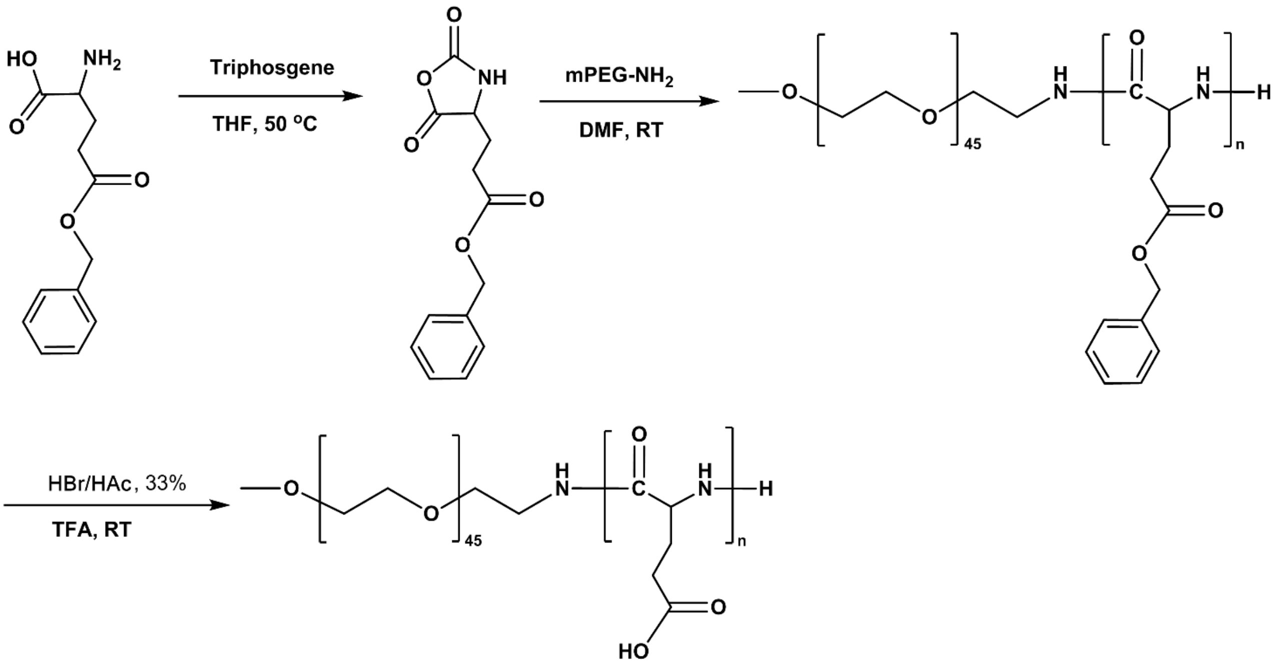

Supplementary Fig. 32 Synthesis route of mPEG-b-PLG.

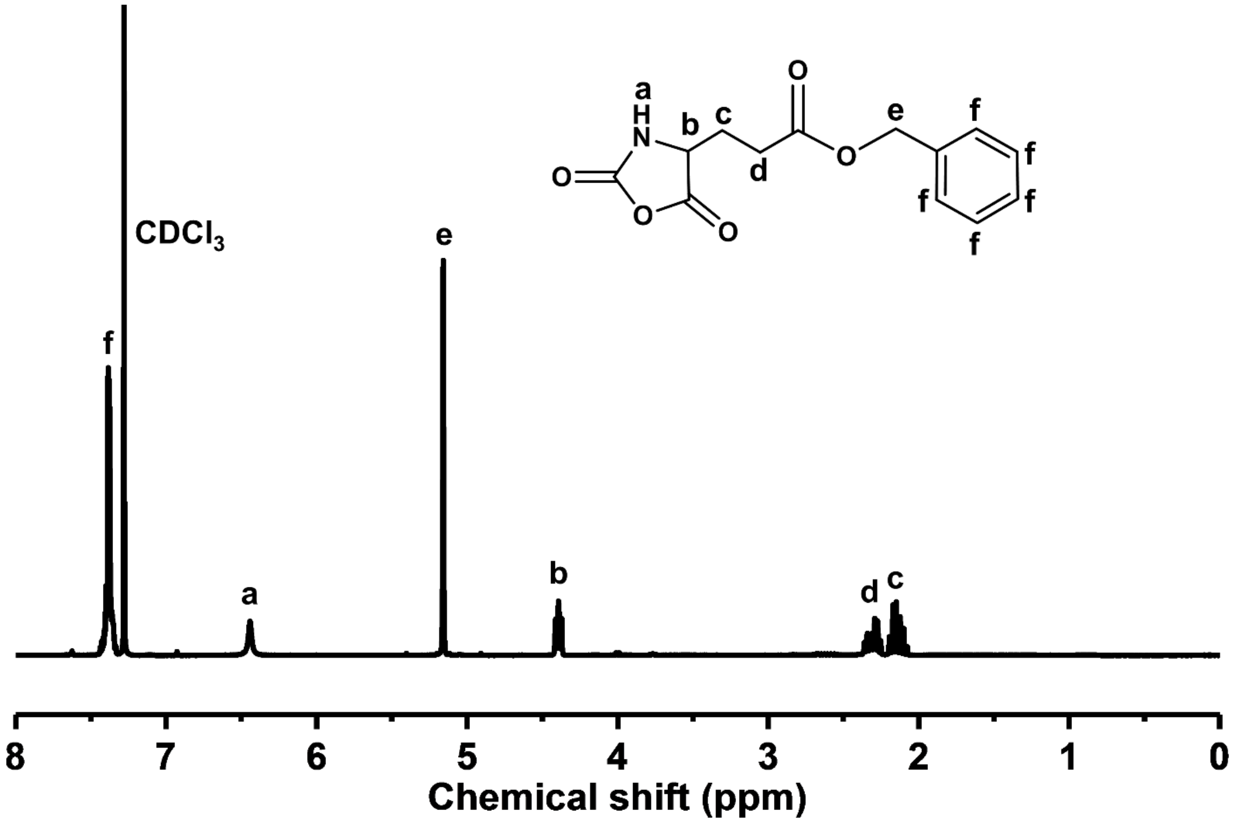

Supplementary Fig. 33 ^{1}H NMR spectrum of Glu(OBzl)-NCA (400 MHz, $CDCl_3$).

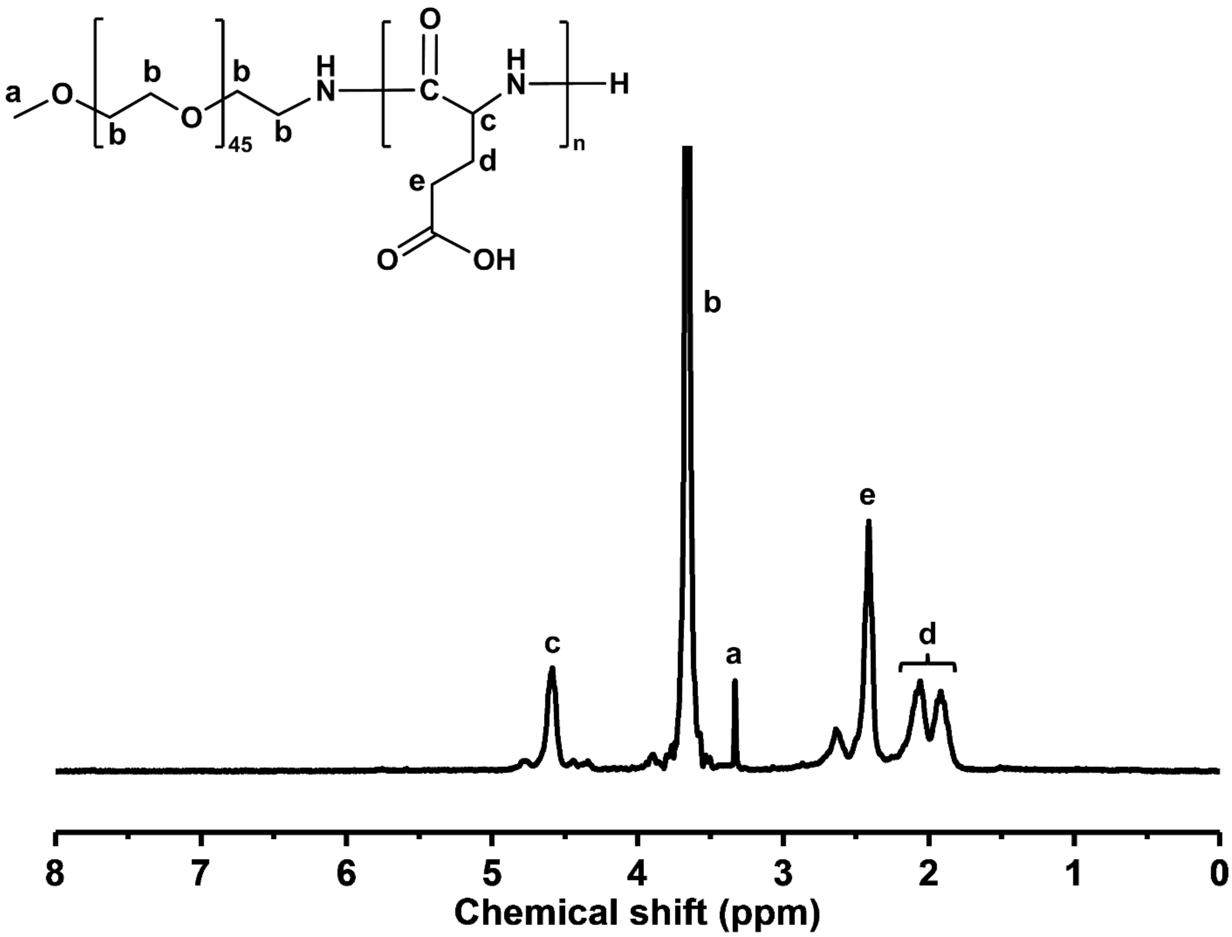

Supplementary Fig. 34 ^{1}H NMR spectrum of mPEG-b-PLG (400 MHz, CF_3COOD).

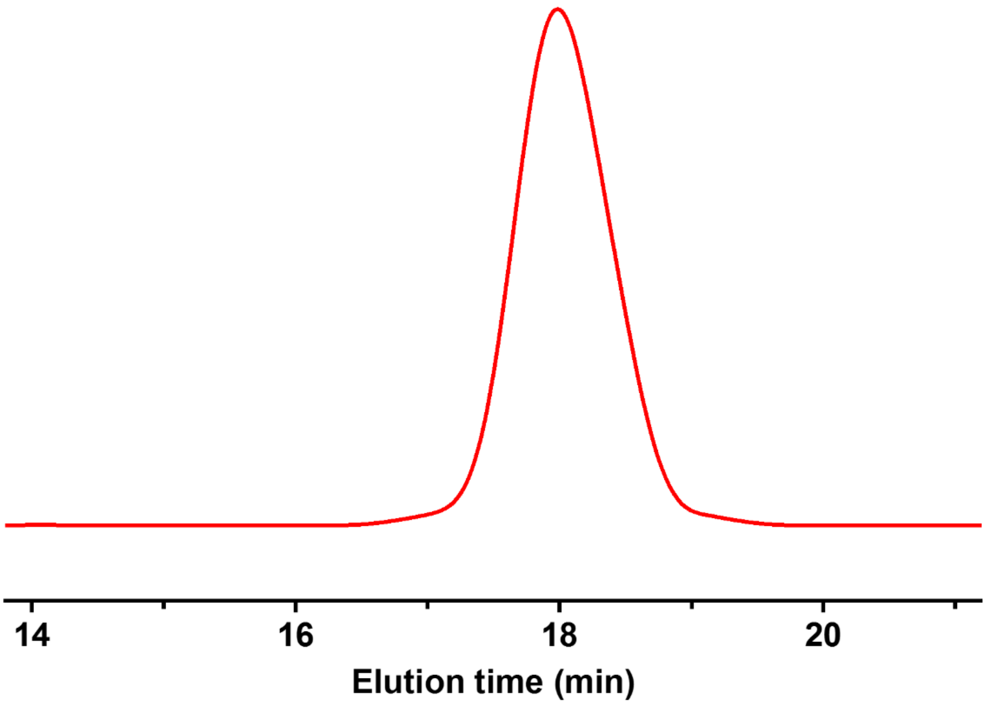

Supplementary Fig. 35 GPC trace of mPEG-b-PLG (PDI=1.08).

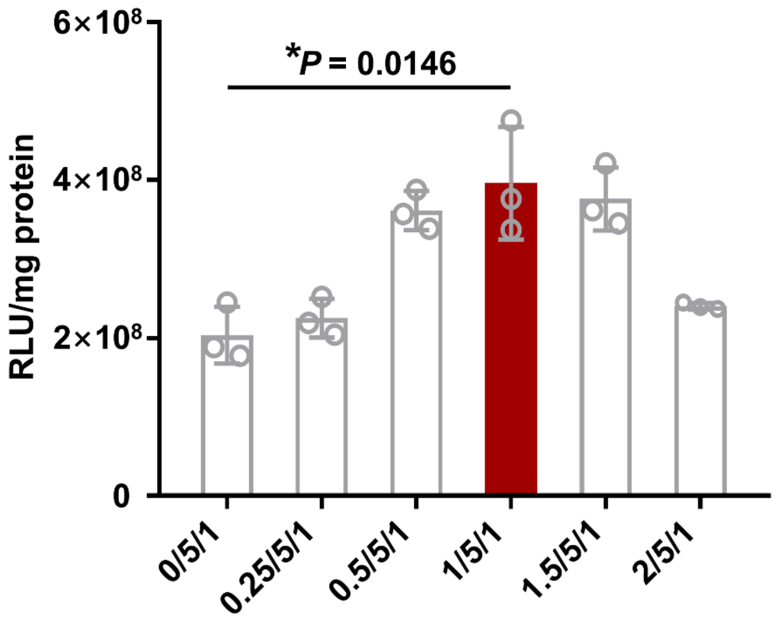

Supplementary Fig. 36 Transfection efficiency of mPEG-b-PLG/PEI-RT3/DNA at different mass ratios in B16F10 cells. Data are presented as mean ± SD, n=3 biologically independent samples *P* values are calculated by the two-tailed student's t-test as indicated in the figure, *P<0.05.

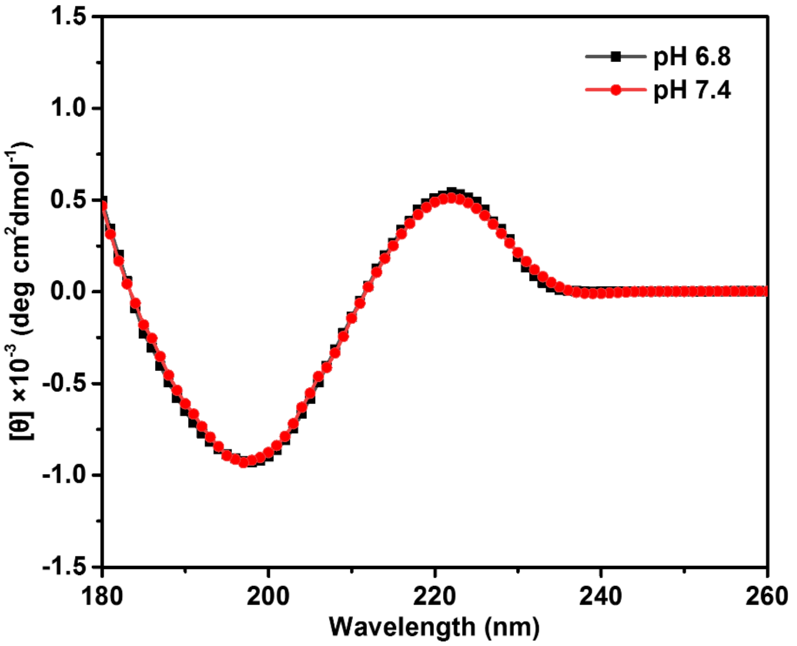

Supplementary Fig. 37 The CD spectra of mPEG-b-PLG at pH 6.8 and 7.4.

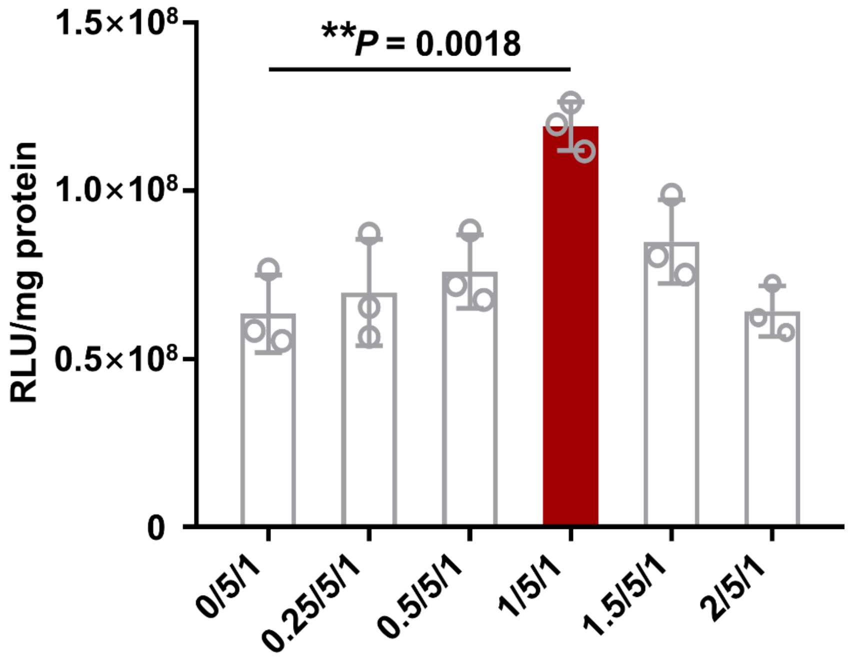

Supplementary Fig. 38 Transfection efficiency of mPEG-b-PLG/PEI-RT3/DNA at different mass ratios in 4T1 cells. Data are presented as mean ± SD, n=3 biologically independent samples. *P* values are calculated by the two-tailed student's t-test as indicated in the figure, ***P*<0.01.

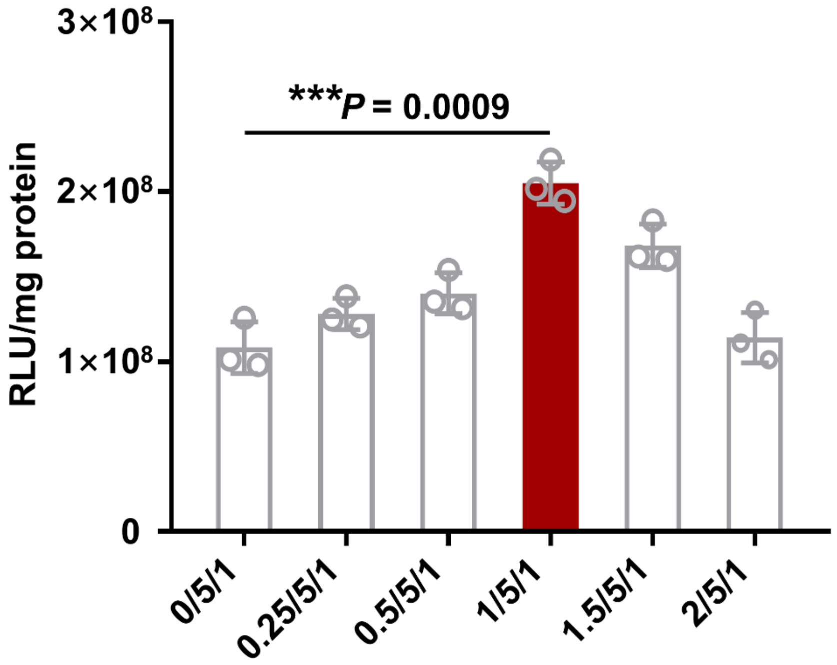

Supplementary Fig. 39 Transfection efficiency of mPEG-b-PLG/PEI-RT3/DNA at different mass ratios in HeLa cells. Data are presented as mean ± SD, n=3 biologically independent samples. *P* values are calculated by the two-tailed student's t-test as indicated in the figure, ****P*<0.001.

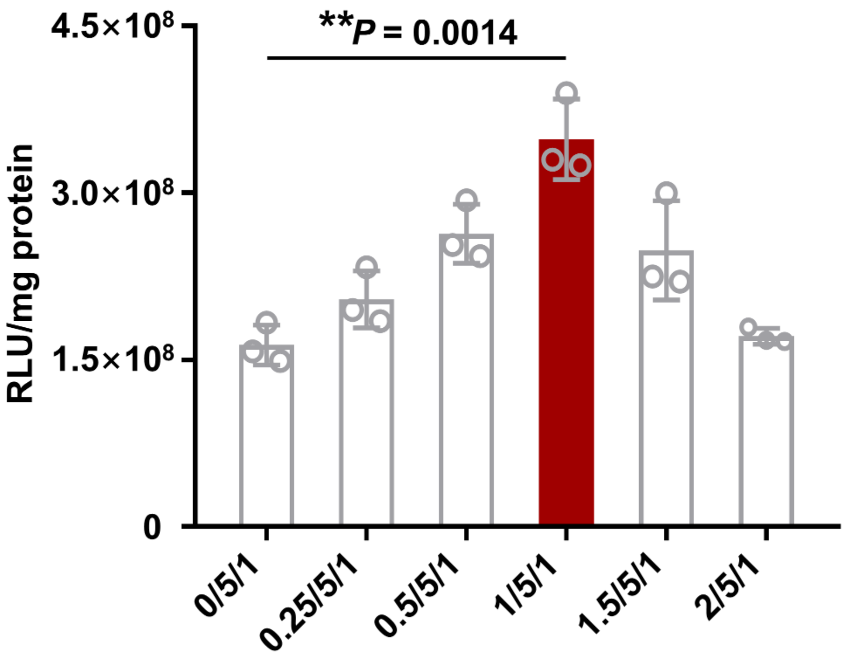

Supplementary Fig. 40 Transfection efficiency of mPEG-b-PLG/PEI-RT3/DNA at different mass ratios in MCF-7 cells. Data are presented as mean ± SD, n=3 biologically independent samples. *P* values are calculated by the two-tailed student's t-test as indicated in the figure, ***P*<0.01.

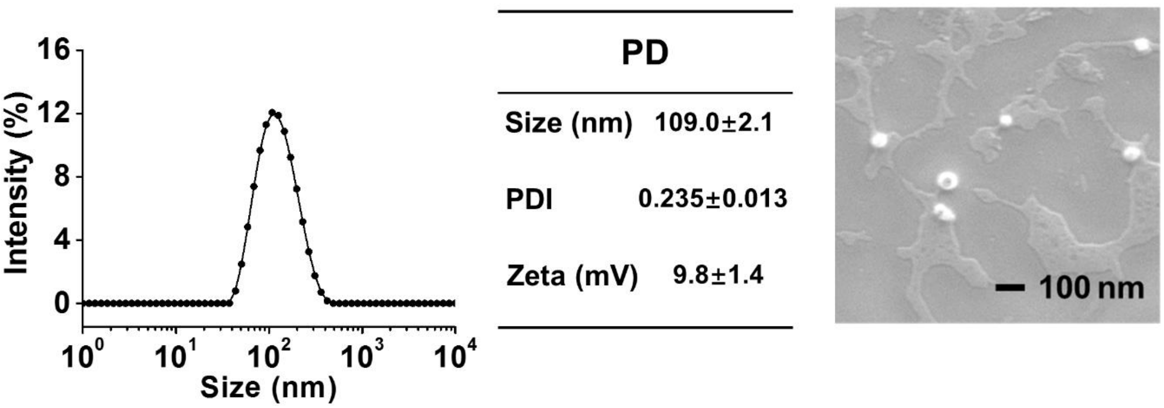

Supplementary Fig. 41 Hydrodynamic particle size, zata potential, and representive scanning electron microscopy image of PD. A representative image of three independent samples from each group is shown in the figure. PD: PEI-RT/DNA.

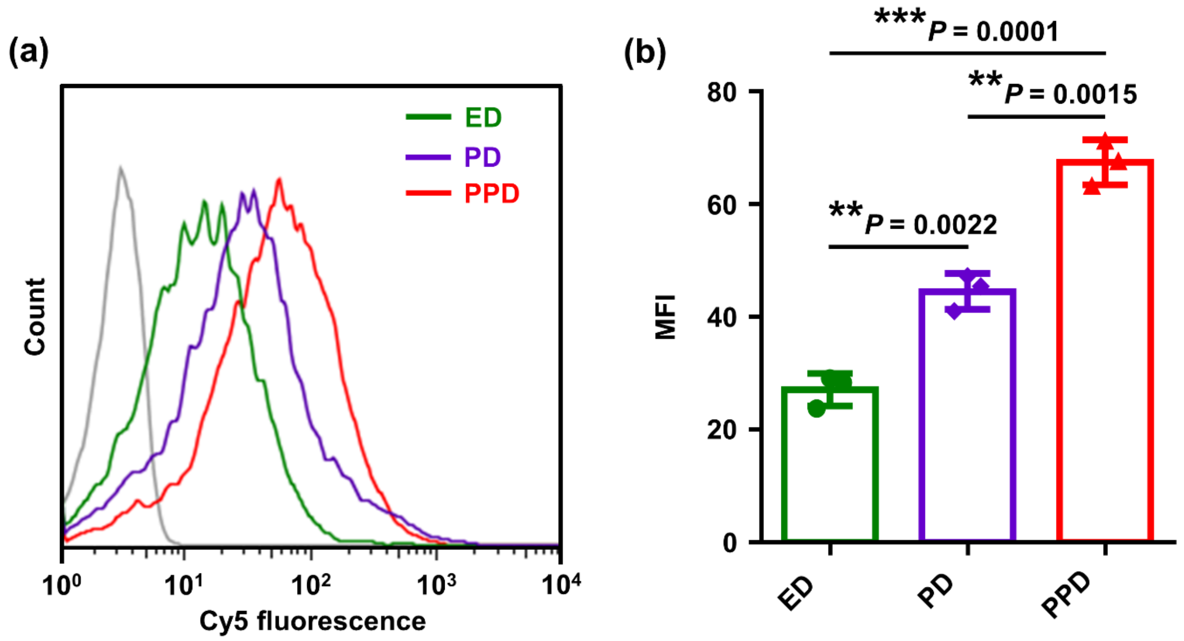

Supplementary Fig. 42 (a) Representative cellular uptake of ED, PD, and PPD by flow cytometry in 4T1 cells. **(b)** The corresponding mean flourence intencity (MFI) of 4T1 cells incubated with ED, PD, or PPD. Data in **Supplementary Fig. b** are presented as mean ± SD, n=3 biologically independent samples in **Supplementary Fig. b**. *P* values are calculated by the two-tailed student's t-test in **Supplementary Fig. b** as indicated in the figure, ***P*<0.01 and ****P*<0.001. ED: PEI1.8k/DNA, PD: PEI-RT3/DNA, PPD: mPEG-b-PLG/PEI-RT3/DNA.

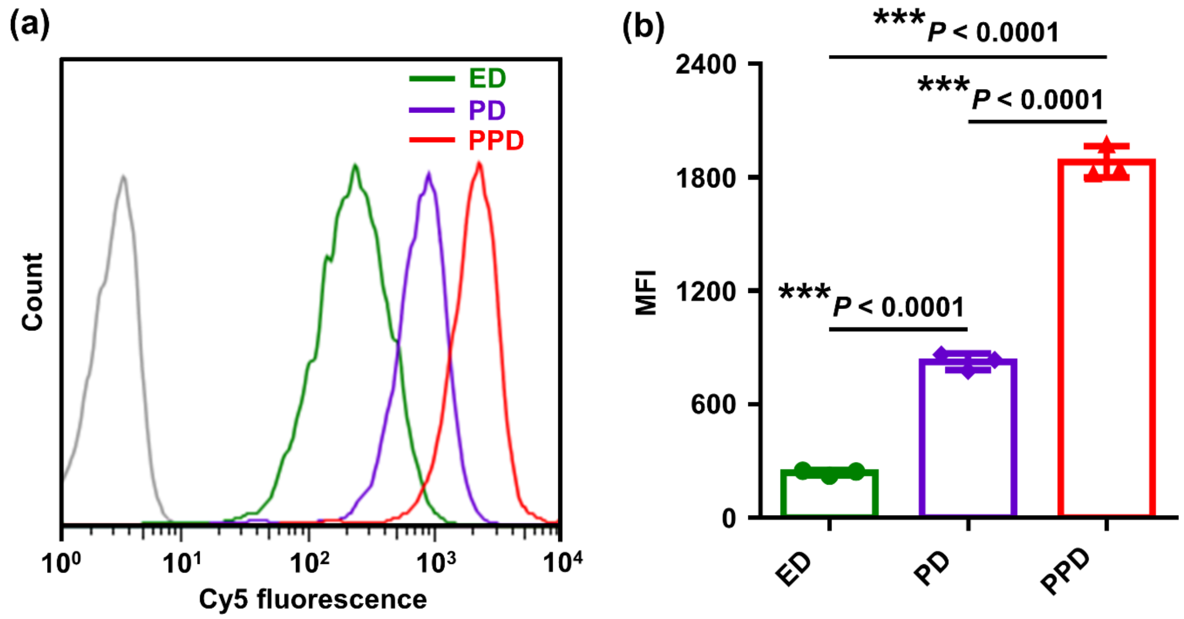

Supplementary Fig. 43 (a) Representative cellular uptake of ED, PD, and PPD by flow cytometry in HeLa cells. **(b)** The corresponding mean flourence intencity (MFI) of HeLa cells incubated with

ED, PD, or PPD. Data in **Supplementary Fig. b** are presented as mean ± SD, n=3 biologically independent samples in **Supplementary Fig. b**. *P* values are calculated by the two-tailed student's t-test in **Supplementary Fig. b** as indicated in the figure, ***P*<0.01 and ****P*<0.001.

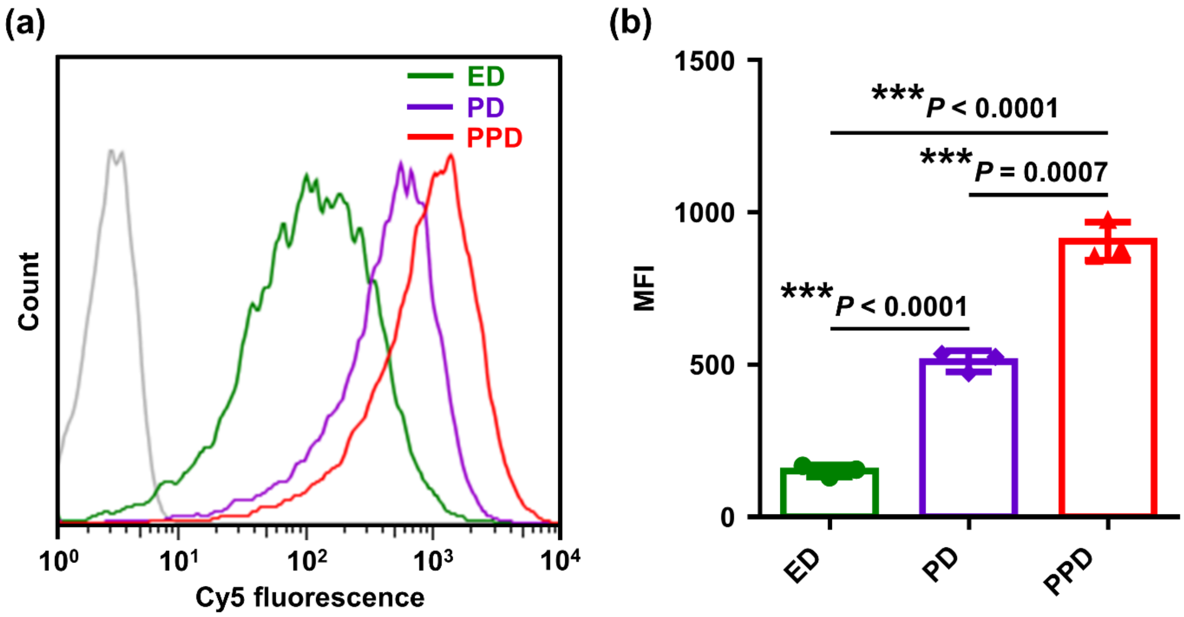

Supplementary Fig. 44 (a) Representative cellular uptake of ED, PD, and PPD by flow cytometry in MCF-7 cells. **(b)** The corresponding mean flourence intencity (MFI) of MCF-7 cells incubated with ED, PD, or PPD. Data in **Supplementary Fig. b** are presented as mean ± SD, n=3 biologically independent samples in **Supplementary Fig. b**. *P* values are calculated by the two-tailed student's t-test in **Supplementary Fig. b** as indicated in the figure, ****P*<0.001.

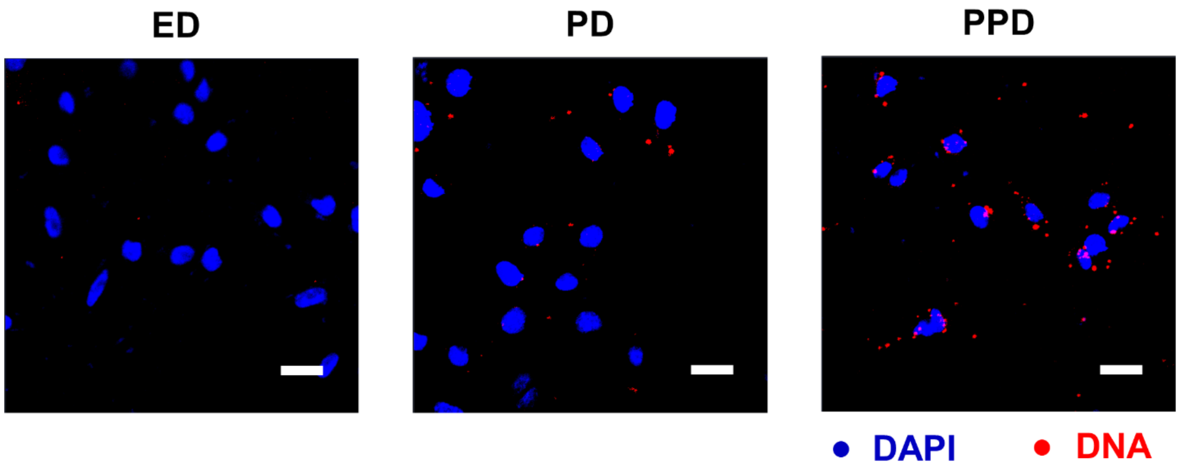

Supplementary Fig. 45 Representative cellular uptake images of PPD, PD, and ED in 4T1 cells, scale bar: 20 μm. A representative image of three independent samples from each group is shown in the figure.

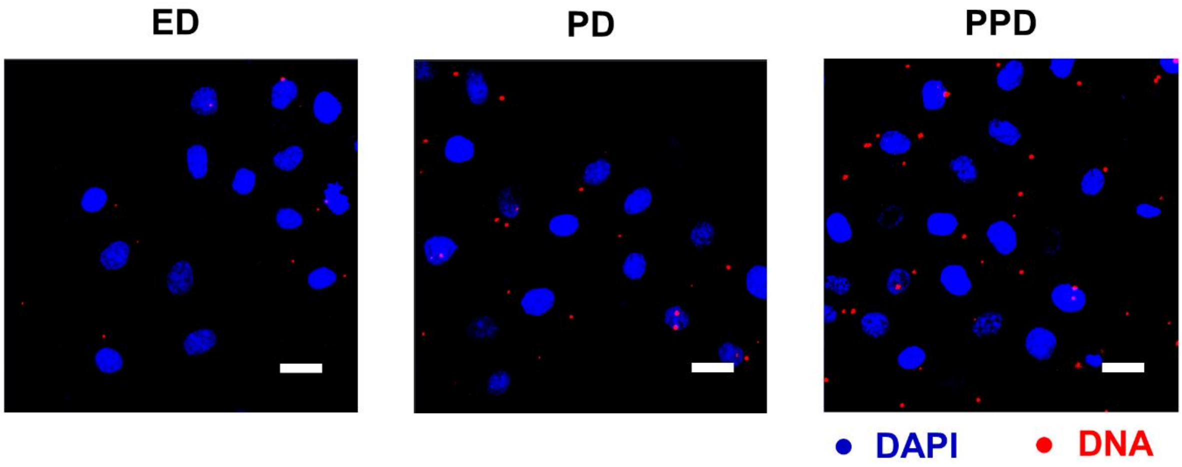

Supplementary Fig. 46 Representative cellular uptake images of PPD, PD, and ED in HeLa cells, scale bar: 20 μm. A representative image of three independent samples from each group is shown in the figure.

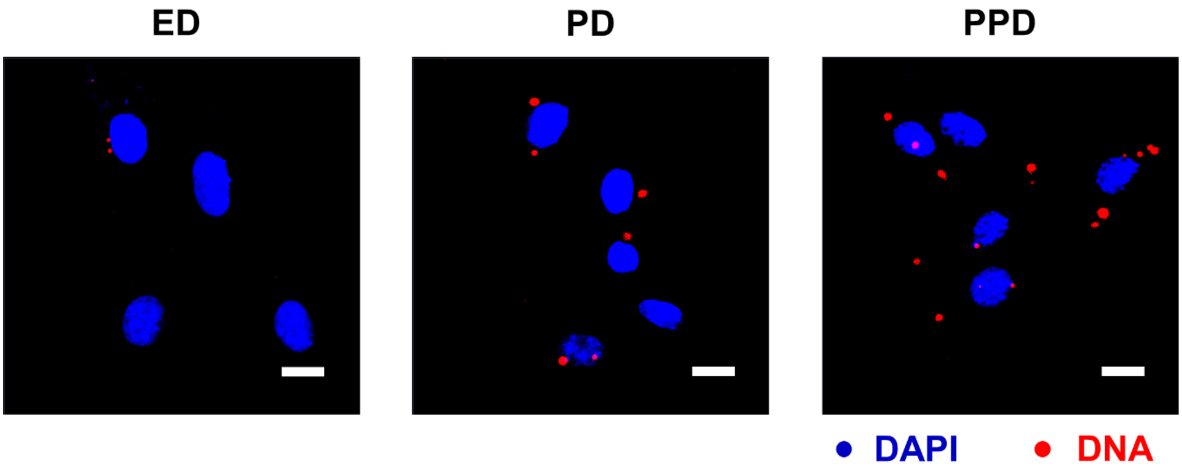

Supplementary Fig. 47 Representative cellular uptake images of PPD, PD, and ED in MCF-7 cells, scale bar: 20 μm. A representative image of three independent samples from each group is shown in the figure.

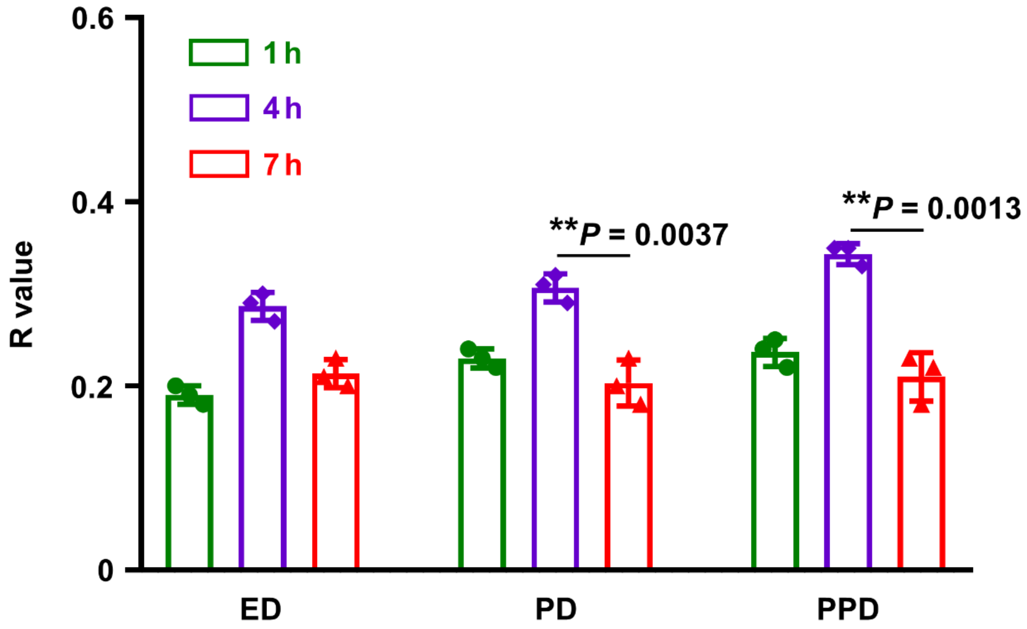

Supplementary Fig. 48 Correlation coefficient R value obtained from the CLSM images for endo/lysosomal escape ability evaluation in B16F10 cells. Data are presented as mean ± SD, n=3 biologically independent samples. *P* values are calculated by the two-tailed student's t-test as indicated in the figure, $**P<0.01$.

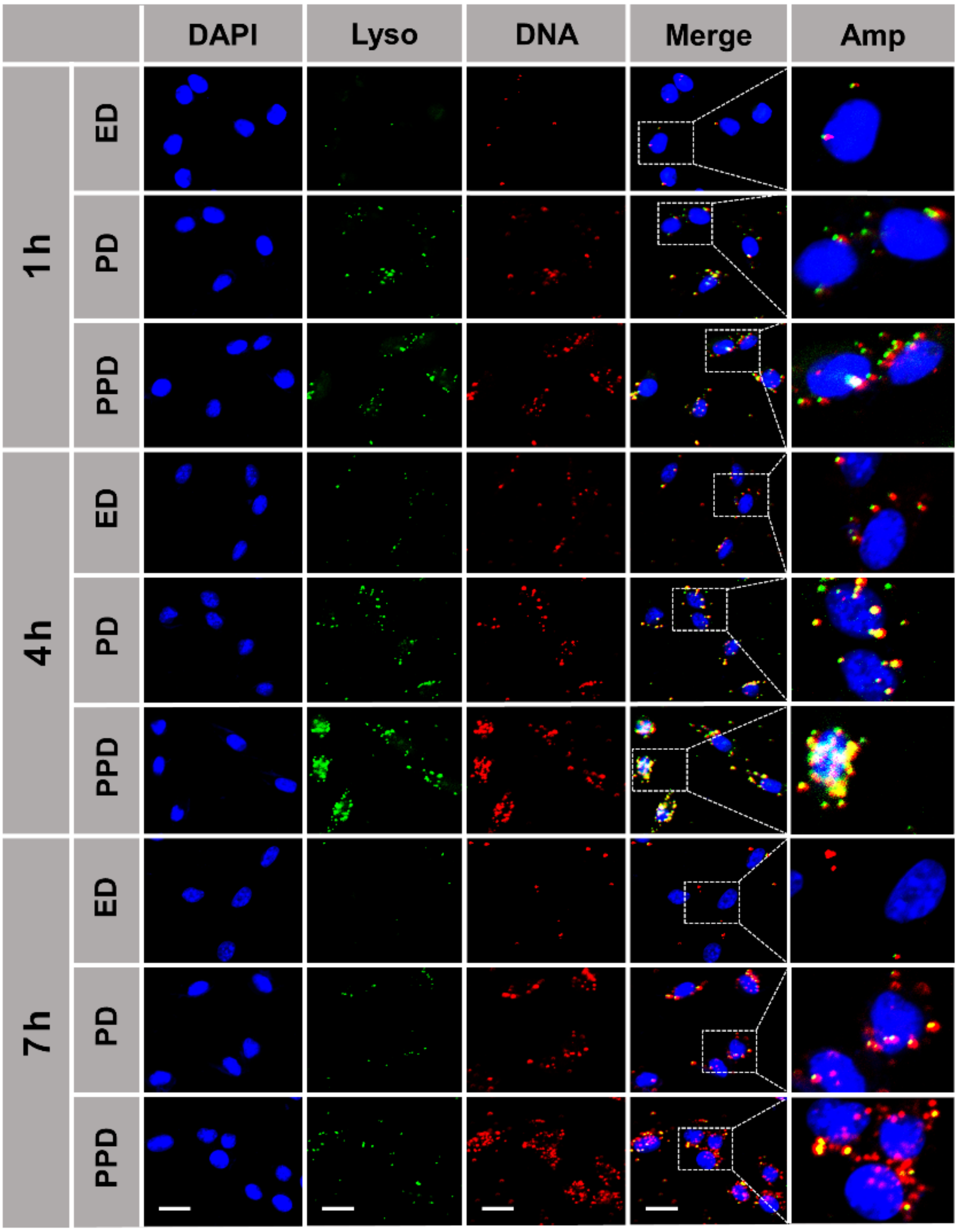

Supplementary Fig. 49 Representative endo/lysosomal escape images of PPD, PD, and ED at various time pointes (1, 4, and 7 h) in 4T1 cells, scale bar: 20 μm. A representative image of three independent samples from each group is shown in the figure.

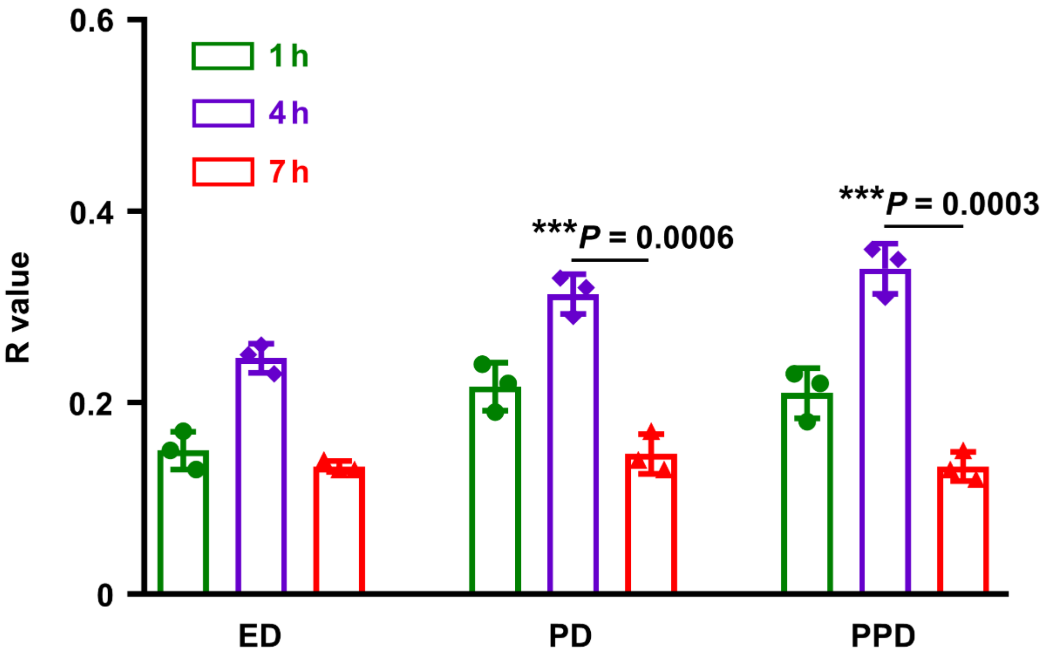

Supplementary Fig. 50 Correlation coefficient R value obtained from the CLSM images for endo/lysosomal escape ability evaluation in 4T1 cells. Data are presented as mean ± SD, n=3 biologically independent samples. *P* values are calculated by the two-tailed student's t-test as indicated in the figure, ****P*<0.001.

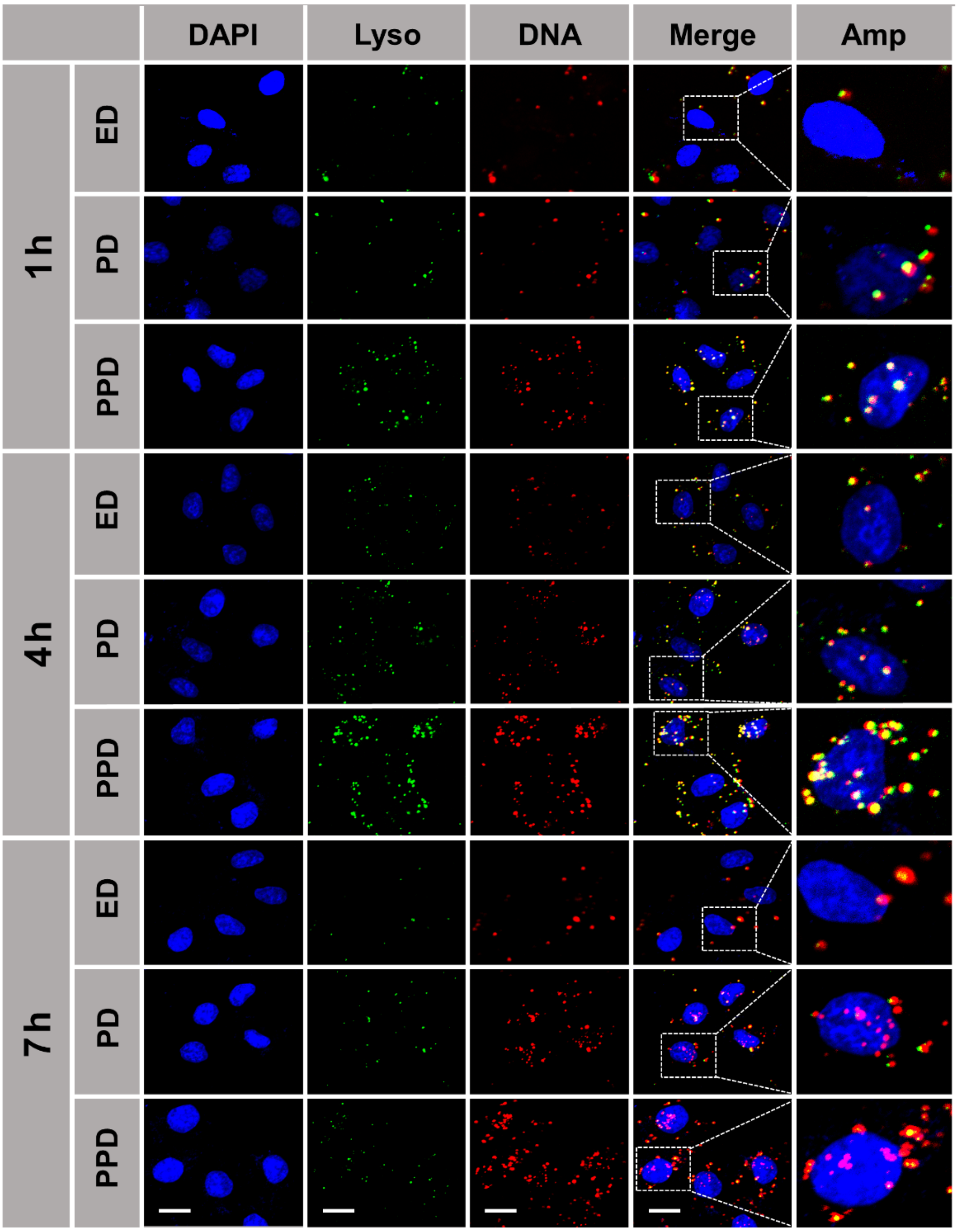

Supplementary Fig. 51 Representative endo/lysosomal escape images of PPD, PD, and ED at various time pointes (1, 4, and 7 h) in HeLa cells, scale bar: 20 μm. A representative image of three independent samples from each group is shown in the figure.

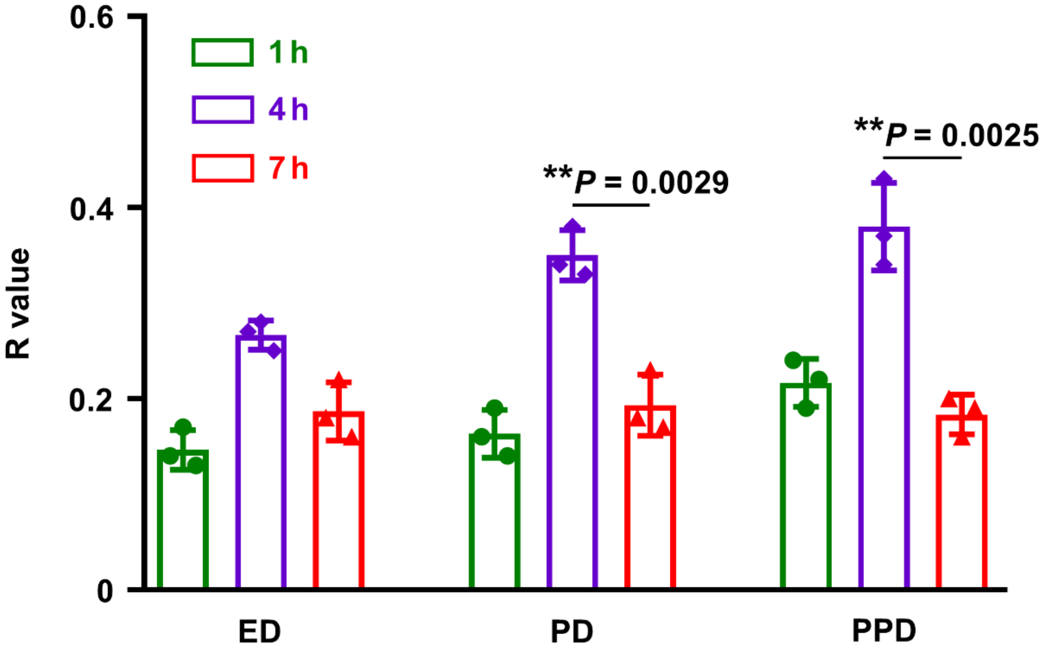

Supplementary Fig. 52 Correlation coefficient R value obtained from the CLSM images for endosomal escape ability evaluation in HeLa cells. Data are presented as mean ± SD, n=3 biologically independent samples. *P* values are calculated by the two-tailed student's t-test as indicated in the figure, $^{**}P<0.01$.

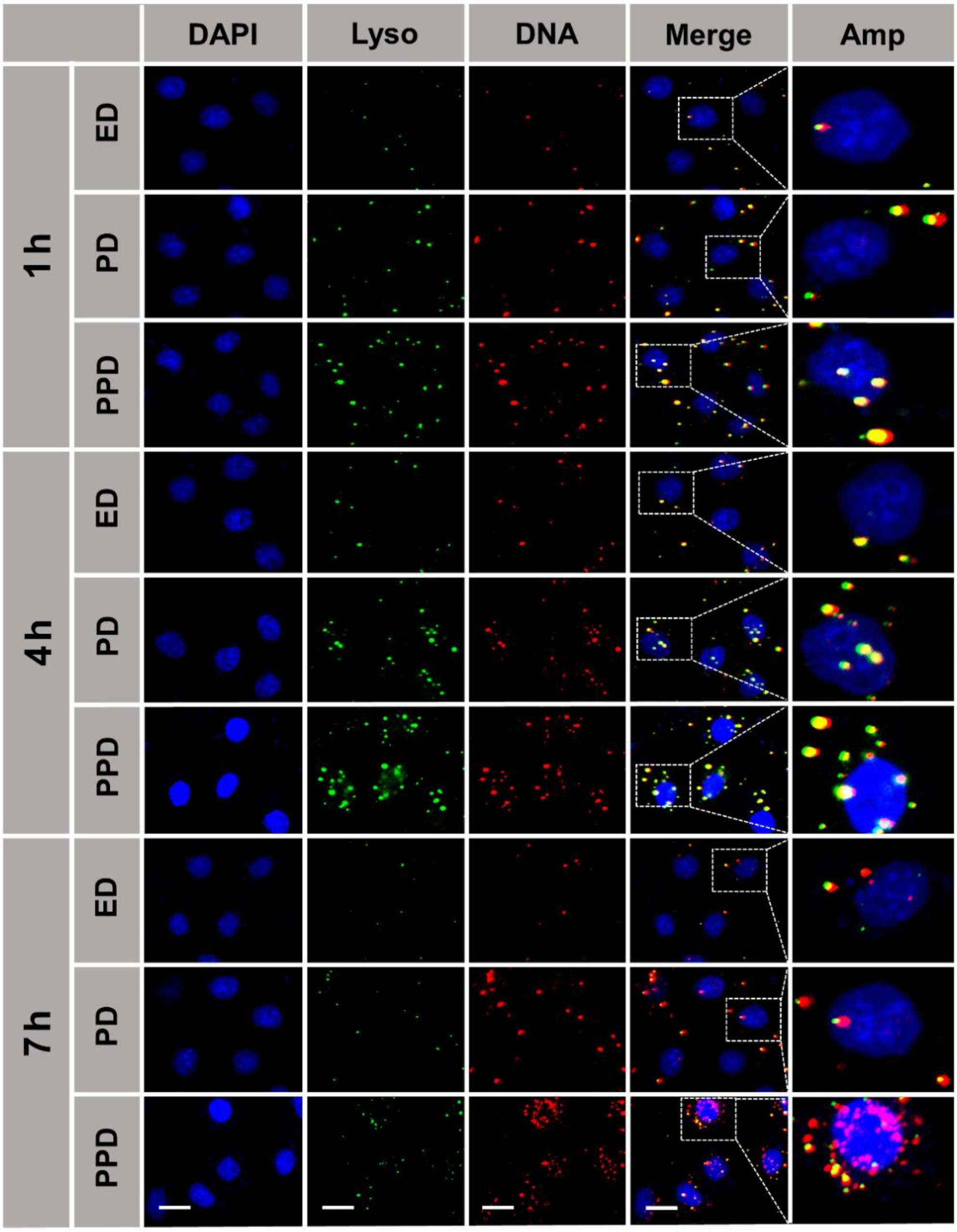

Supplementary Fig. 53 Representative endo/lysosomal escape images of PPD, PD, and ED at various time pointes (1, 4, and 7 h) in MCF-7 cells, scale bar: 20 μm. A representative image of three independent samples from each group is shown in the figure.

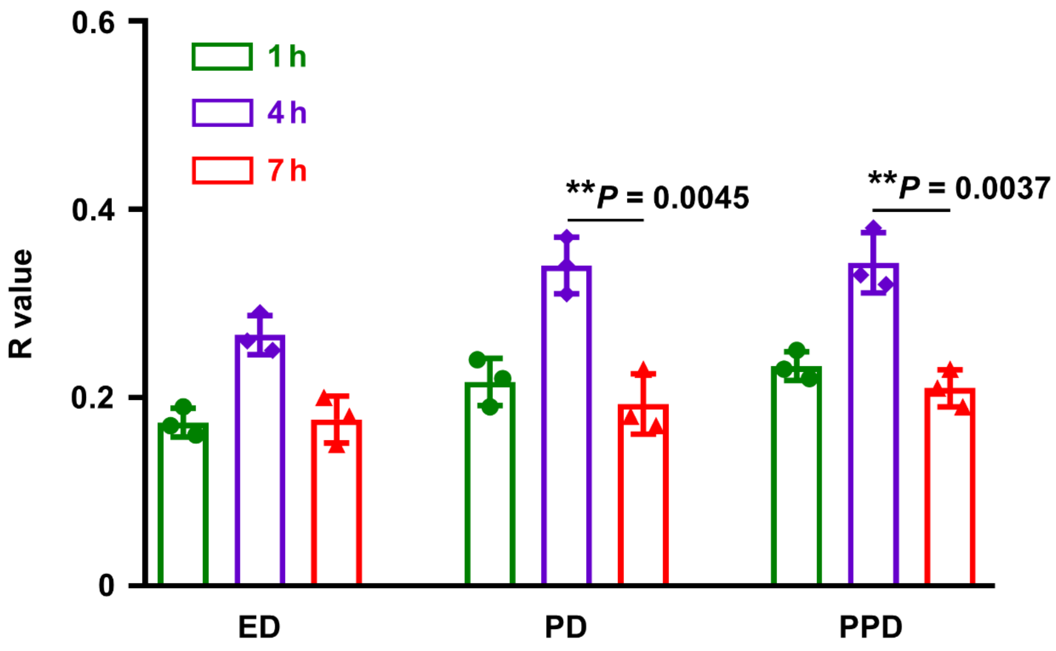

Supplementary Fig. 54 Correlation coefficient R value obtained from the CLSM images for endo/lysosomal escape ability evaluation in MCF-7 cells. Data are presented as mean ± SD, n=3 biologically independent samples. *P* values are calculated by the two-tailed student's t-test as indicated in the figure, $^{**}P<0.01$.

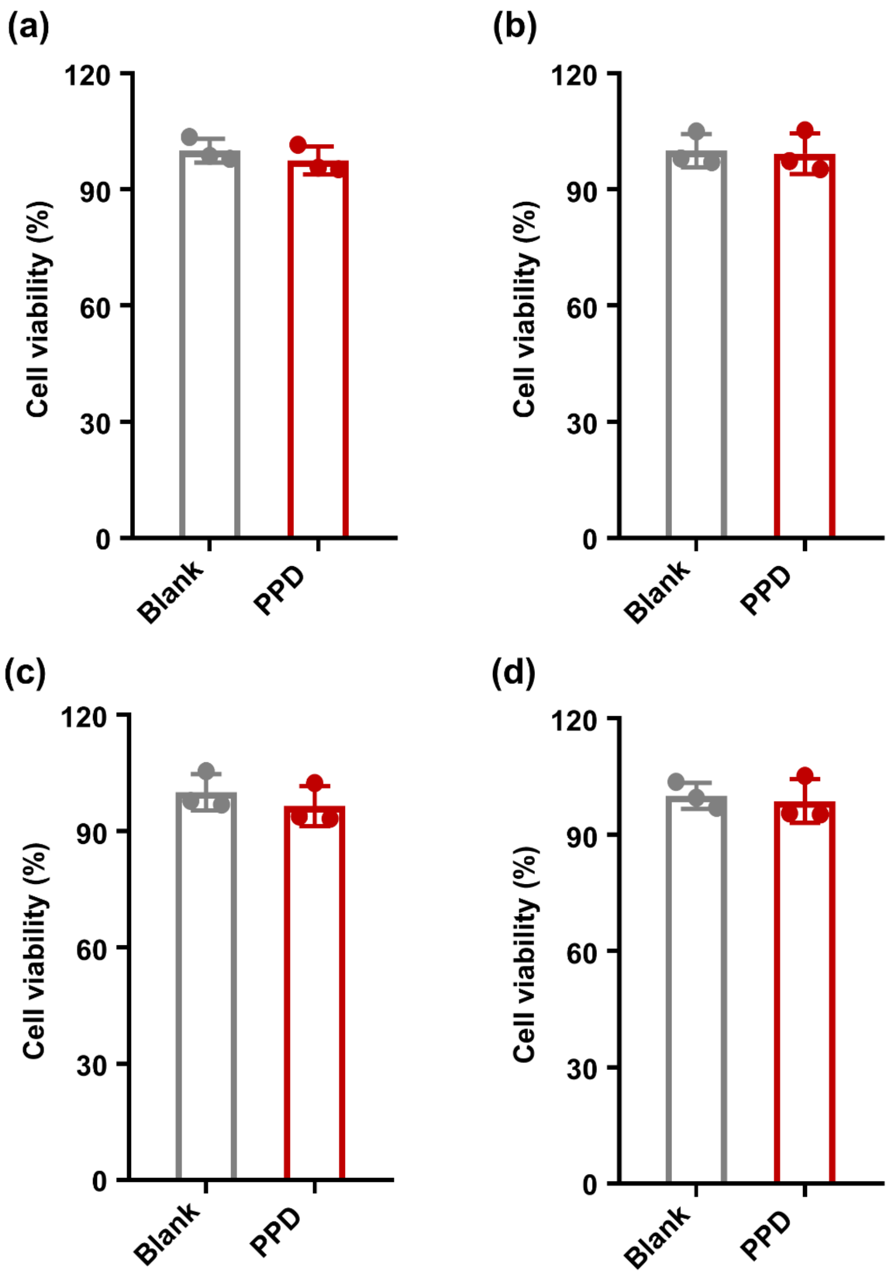

Supplementary Fig. 55 The cell viability of PPD in **(a)** B16F10, **(b)** 4T1, **(c)** HeLa, and (d) MCF-7 cells. Data are presented as mean ± SD, n=3 biologically independent samples.

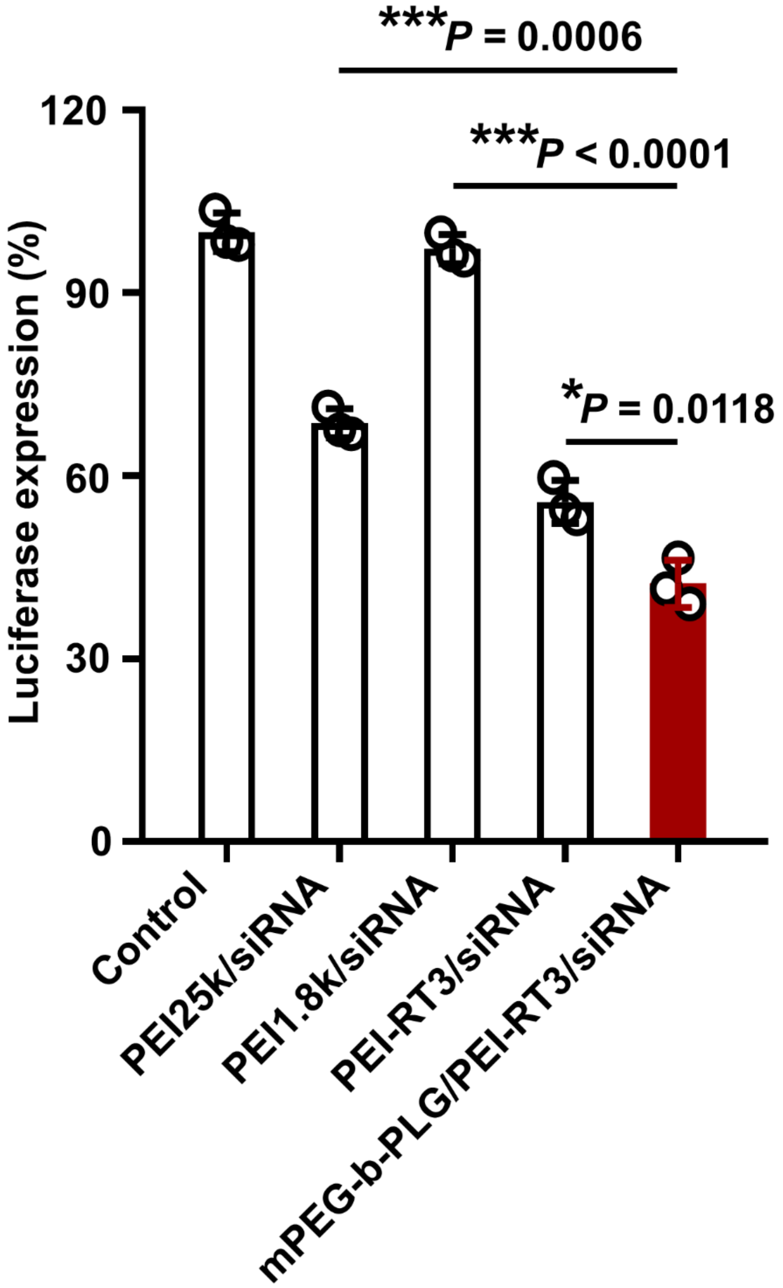

Supplementary Fig. 56 Gene silience efficiency of carrier/siRNA in Huh-7 Luc cells. Data are presented as mean ± SD, n=3 biologically independent samples. *P* values are calculated by the two-tailed student's t-test as indicated in the figure, *P<0.05 and ***P<0.001.

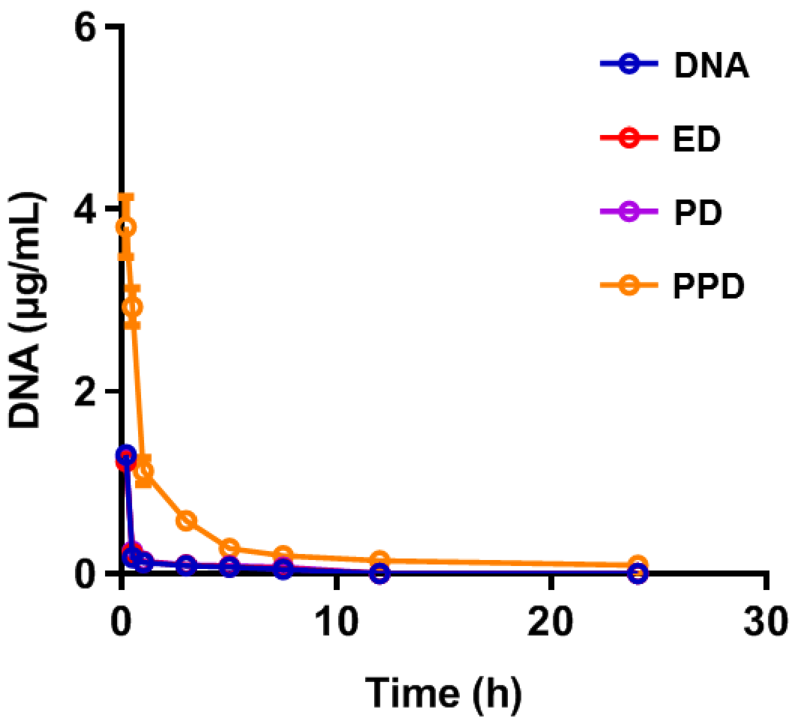

Supplementary Fig. 57 The amount of DNA in the blood versus time after intravenous adminstration with DNA, ED, PD, and PPD, respectively. Data are presented as mean ± SD, n=3 biologically independent samples.

Supplementary Table 3 Pharmacokinetic parameters of free DNA, ED, PD, and PD, PPD.

Entry	$t_{1/2}$ [a] (h)	$AUC_{0\text{-}t}$ [b] (μg/mL h)
DNA	0.13 ± 0.02	1.45 ± 0.02
ED	0.16 ± 0.02	1.59 ± 0.02
PD	0.16 ± 0.03	1.77 ± 0.06
PPD	0.71 ± 0.13	8.97 ± 0.61

[a] $t_{1/2}$: half-life.

[b] $AUC_{0\text{-}t}$: area under the DNA concentration-time curve from 0 to 24 h in plasma.

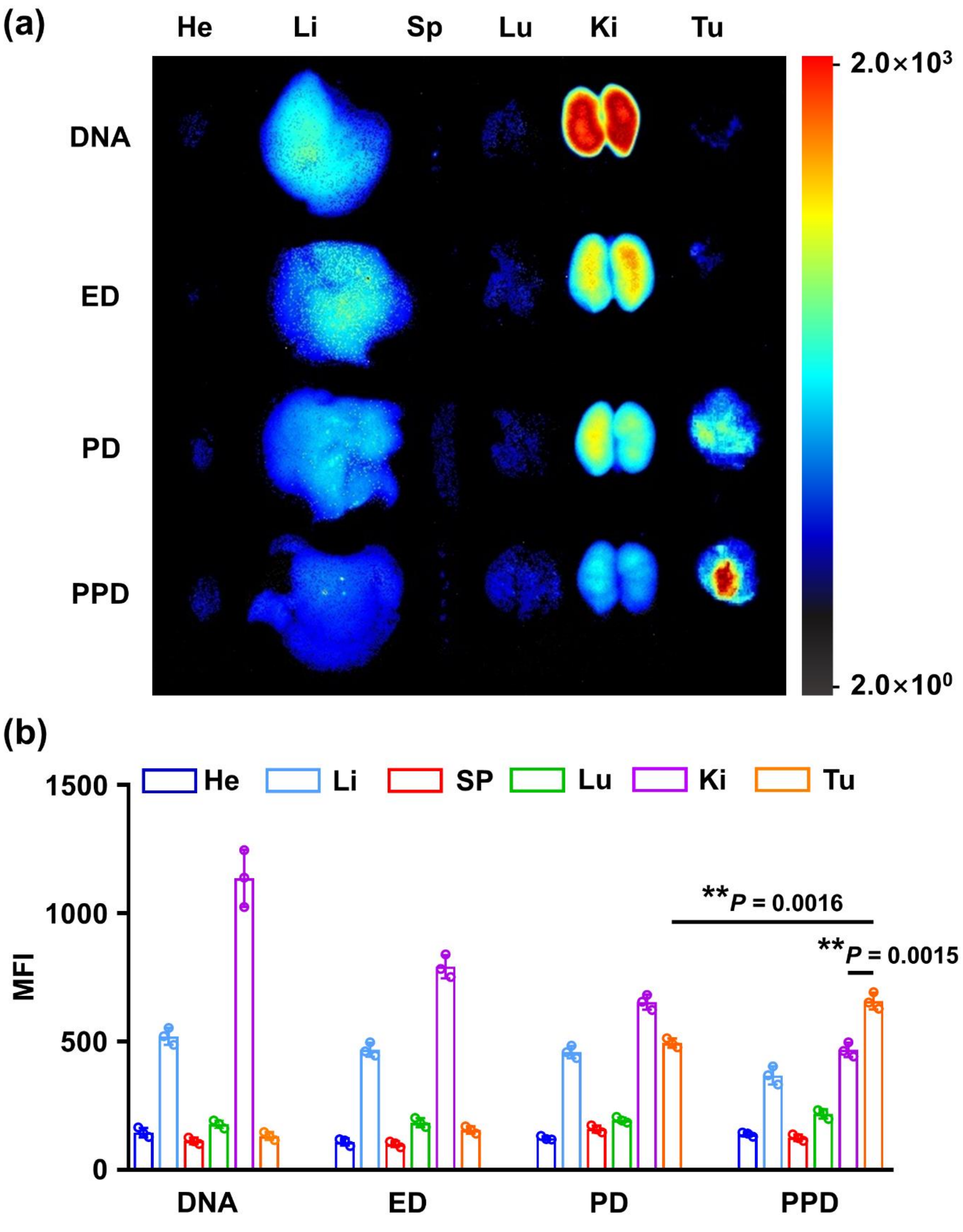

Supplementary Fig. 58 The biodistribution of B16F10 tumour-bearing mice after intravenously administered with PPD. (a) Representative images of biodistribution in heart, liver, spleen, lung, kidney, and tumour after B16F10 tumour-bearing mice intravenously administered with DNA, ED, PD, and PPD, respectively. **(b)** Mean fluorescence intensity (MFI) of biodistribution in heart, liver, spleen, lung, kidney, and tumour after B16F10 tumour-bearing mice intravenously administered with DNA, ED, PD, and PPD, respectively. Data in **Supplementary Fig. b** are presented as mean ± SD,

n=3 biologically independent samples in **Supplementary Fig.b**. *P* values are calculated by the two-tailed student's t-test in **Supplementary Fig. b** as indicated in the figure, **P<0.01.

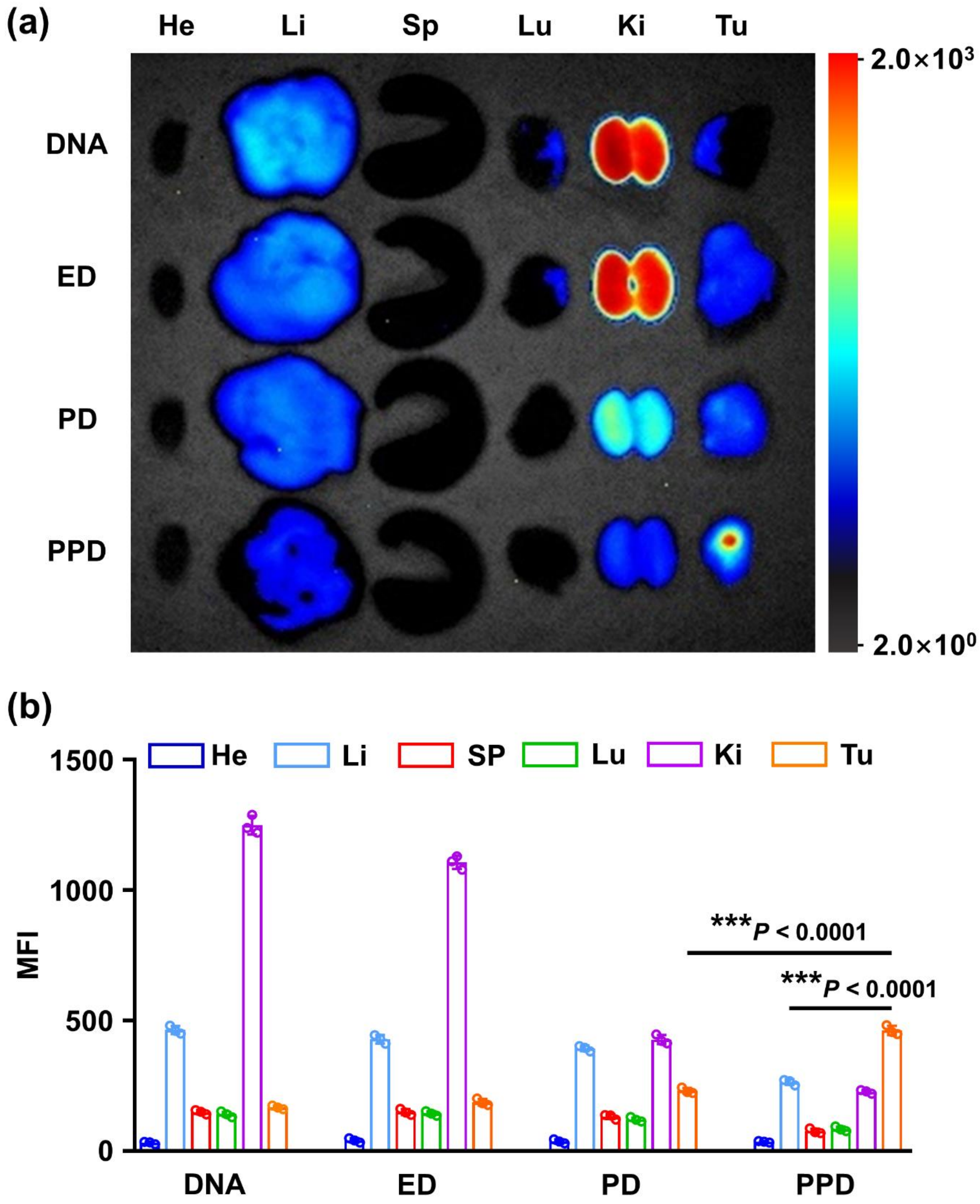

Supplementary Fig. 59 The biodistribution of 4T1 tumour-bearing mice after intravenously administered with PPD. (a) Representative images of biodistribution in heart, liver, spleen, lung, kidney, and tumour after 4T1 tumour-bearing mice intravenously administered with DNA, ED, PD, and PPD, respectively. **(b)** Mean fluorescence intensity (MFI) of biodistribution in heart, liver, spleen,

lung, kidney, and tumour after 4T1 tumour-bearing mice intravenously administered with DNA, ED, PD, and PPD, respectively. Data in **Supplementary Fig. b** are presented as mean ± SD, n=3 biologically independent samples in **Supplementary Fig.b**. *P* values are calculated by the two-tailed student's t-test in **Supplementary Fig. b** as indicated in the figure, ***P<0.001.

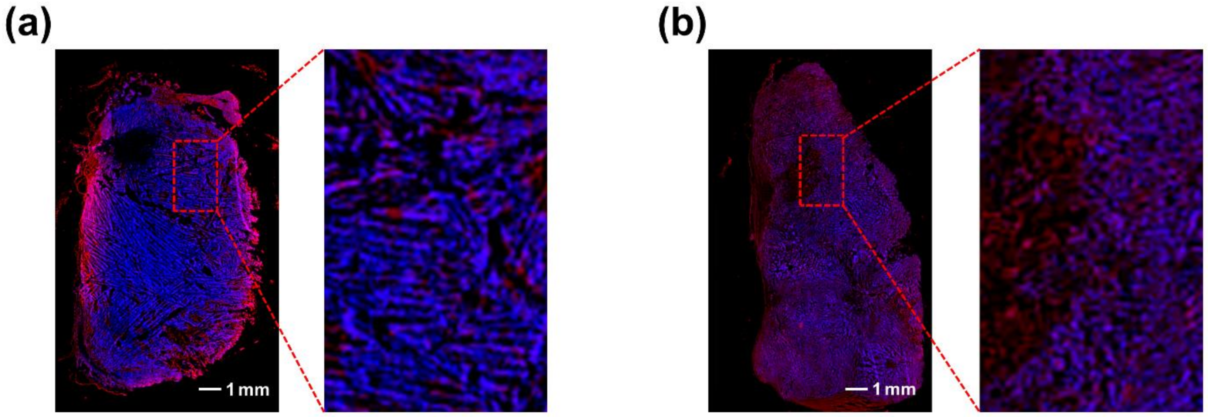

Supplementary Fig. 60 The distribution of PPD complexes within **(a)** B16F10 and **(b)** 4T1 tumour after intravenously administration. A representative image of three independent samples from each group is shown in the figure.

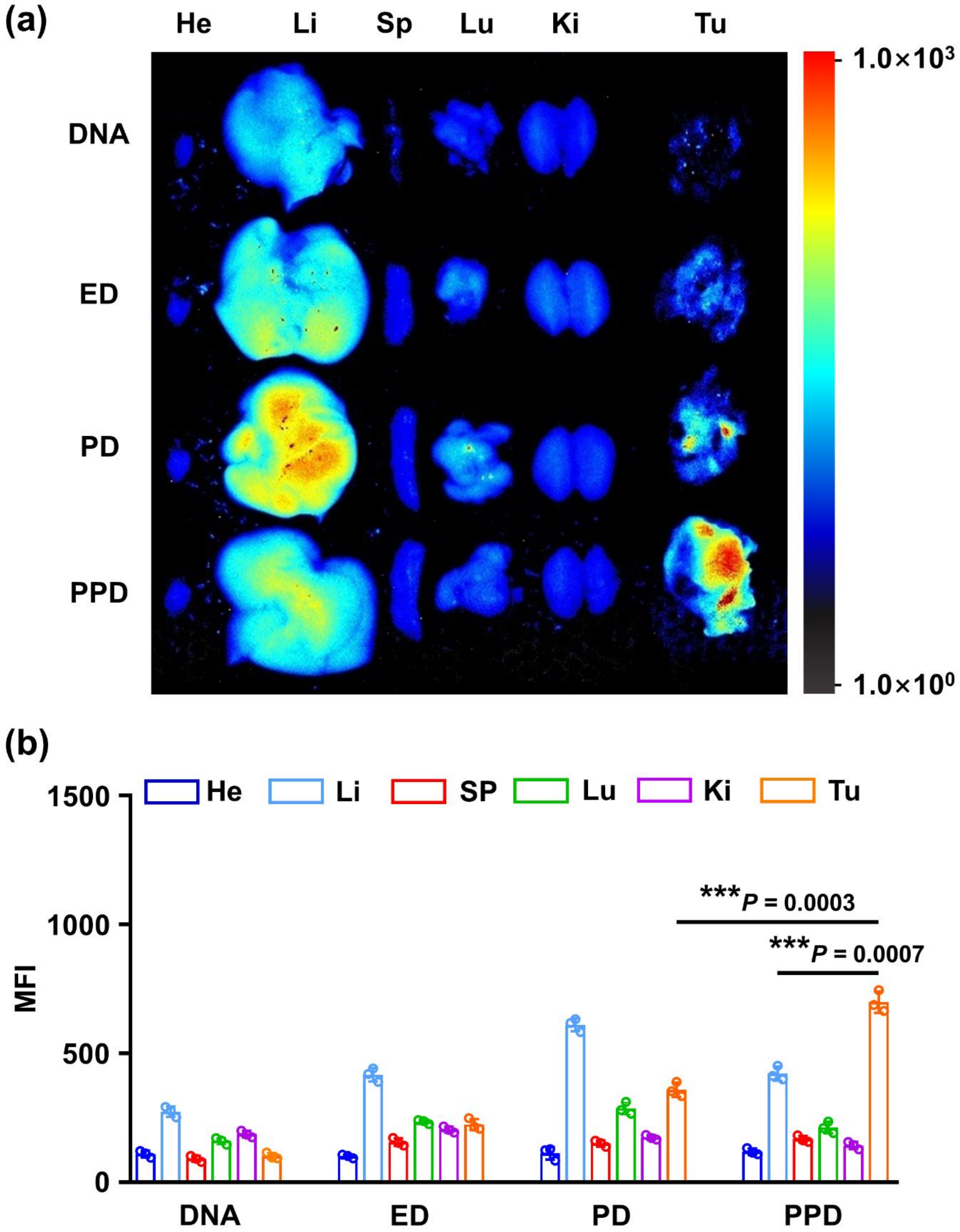

Supplementary Fig. 61 The red fluorescence protein expression of B16F10 tumour-bearing mice after intravenously administered with PPD. (a) Representative images of red fluorescence protein expression in heart, liver, spleen, lung, kidney, and tumour after B16F10 tumour-bearing mice intravenously administered with DNA, ED, PD, and PPD, respectively. **(b)** Mean fluorescence intensity (MFI) of the in vivo transfection in heart, liver, spleen, lung, kidney, and tumour after B16F10 tumour-bearing mice intravenously administered with DNA, ED, PD, and PPD, respectively.

Data in **Supplementary Fig. b** are presented as mean ± SD, n=3 biologically independent samples in **Supplementary Fig.b**. *P* values are calculated by the two-tailed student's t-test in **Supplementary Fig. b** as indicated in the figure, ****P*<0.001.

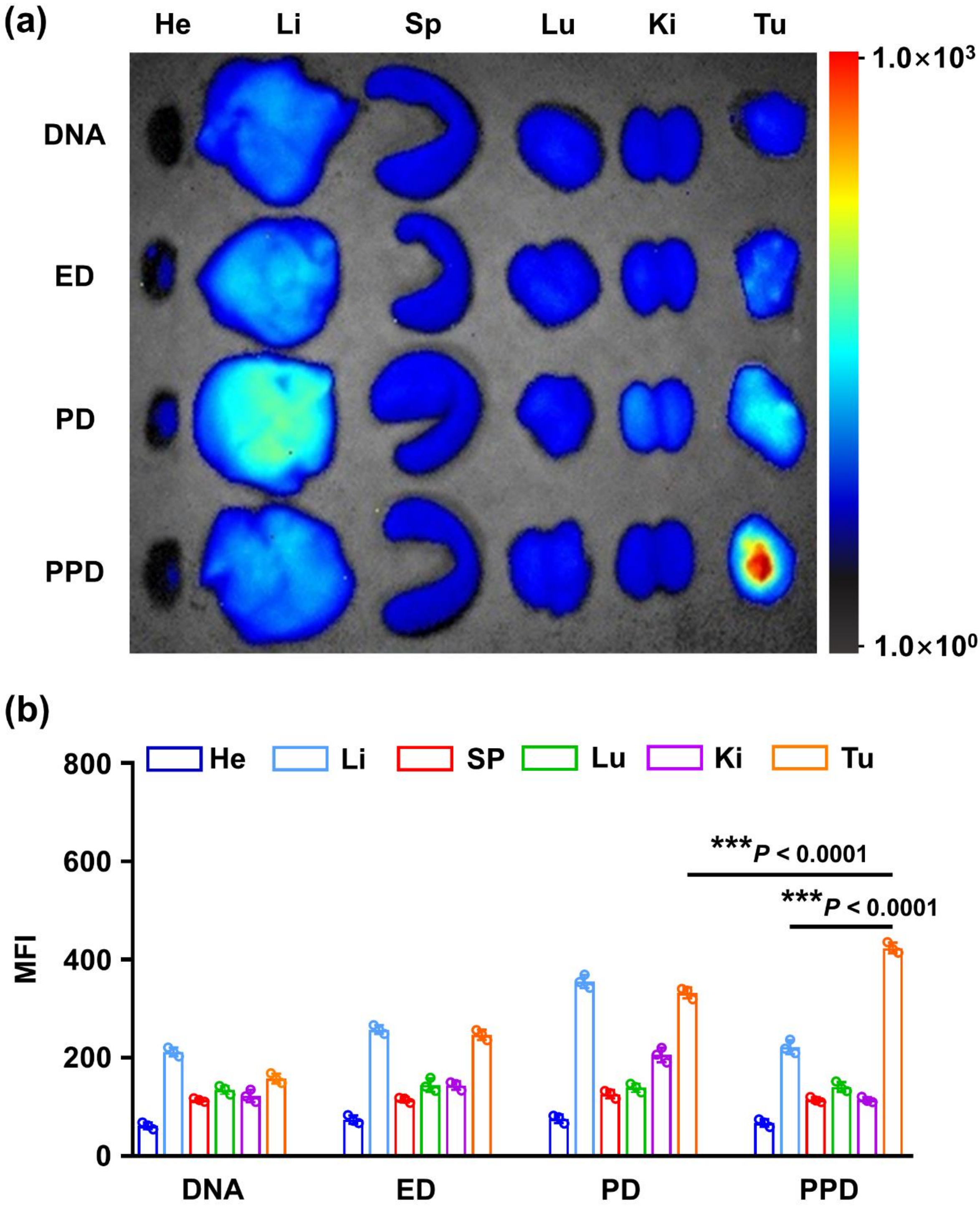

Supplementary Fig. 62 The red fluorescence protein expression of 4T1 tumour-bearing mice after intravenously administered with PPD. (a) Representative images of red fluorescence protein expression in heart, liver, spleen, lung, kidney, and tumour after 4T1 tumour-bearing mice intravenously administered with DNA, ED, PD, and PPD, respectively. **(b)** Mean fluorescence

intensity (MFI) of the in vivo transfection in heart, liver, spleen, lung, kidney, and tumour after 4T1 tumour-bearing mice intravenously administered with DNA, ED, PD, and PPD, respectively. Data in **Supplementary Fig. b** are presented as mean ± SD, n=3 biologically independent samples in **Supplementary Fig. b**. *P* values are calculated by the two-tailed student's t-test in **Supplementary Fig. b** as indicated in the figure, ***P<0.001.

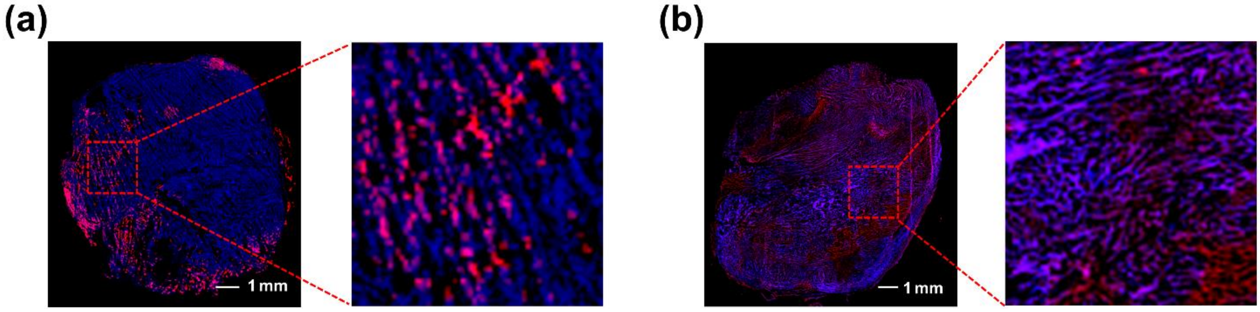

Supplementary Fig. 63 The distribution of expressed red fluorescent protein within **(a)** B16F10 and **(b)** 4T1 tumour after intravenously administration. A representative image of three independent samples from each group is shown in the figure.

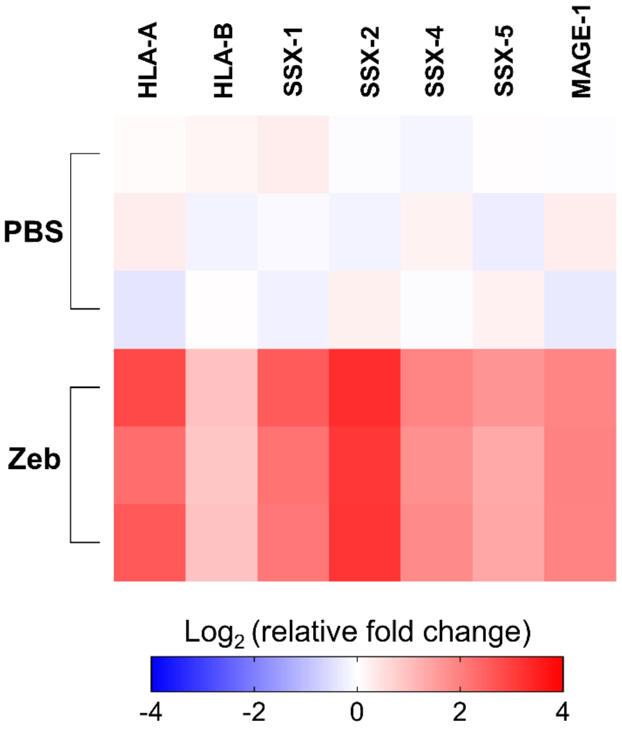

Supplementary Fig. 64 Heatmap of relative mRNA expression of MHC I (HLA-A and HLA-B） and CTA genes (SSX-1, SSX-2, SSX-4, SSX-5, MAGE-1) in A549 cells treated with 0.5 mg/mL Zeb.

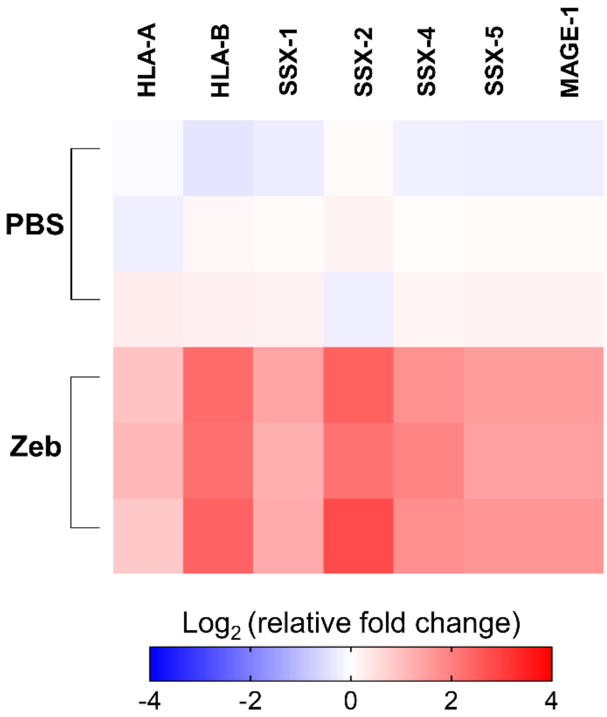

Supplementary Fig. 65 Heatmap of relative mRNA expression of MHC I (HLA-A and HLA-B） and CTA genes (SSX-1, SSX-2, SSX-4, SSX-5, MAGE-1) in HepG2 cells treated with 0.5 mg/mL Zeb.

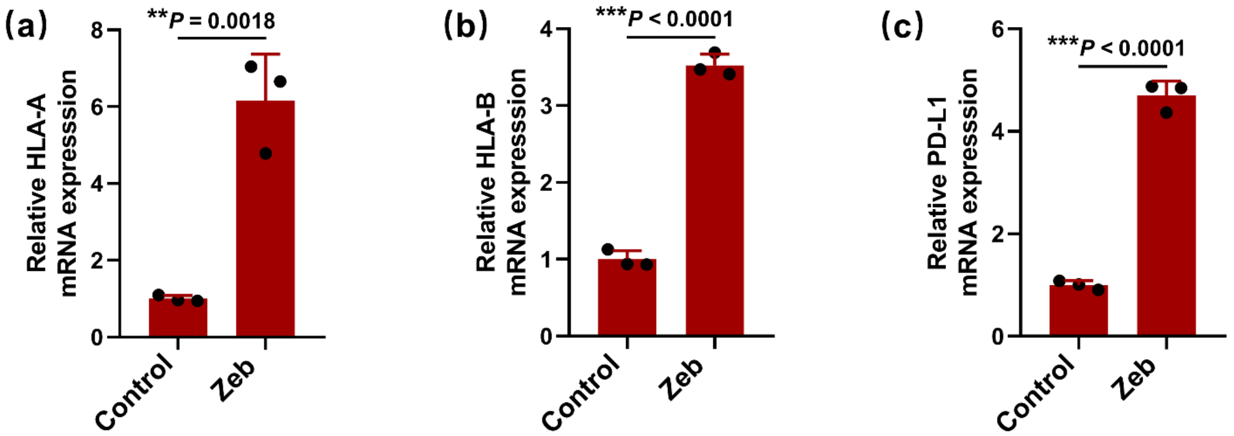

Supplementary Fig. 66 (a) Relative HLA-A mRNA, **(b)** HLA-B mRNA, and **(c)** PD-L1 mRNA expression in HeLa cells treated with 0.5 mg/mL of Zeb by RT-qPCR assay. Data in **Supplementary Fig. a-c** are presented as mean ± SD, n=3 biologically independent samples in **Supplementary Fig. a-c**. *P* values are calculated by the two-tailed student's t-test in **Supplementary Fig. a-c** as indicated in the figure, $^{**}P<0.01$ and $^{***}P<0.001$.

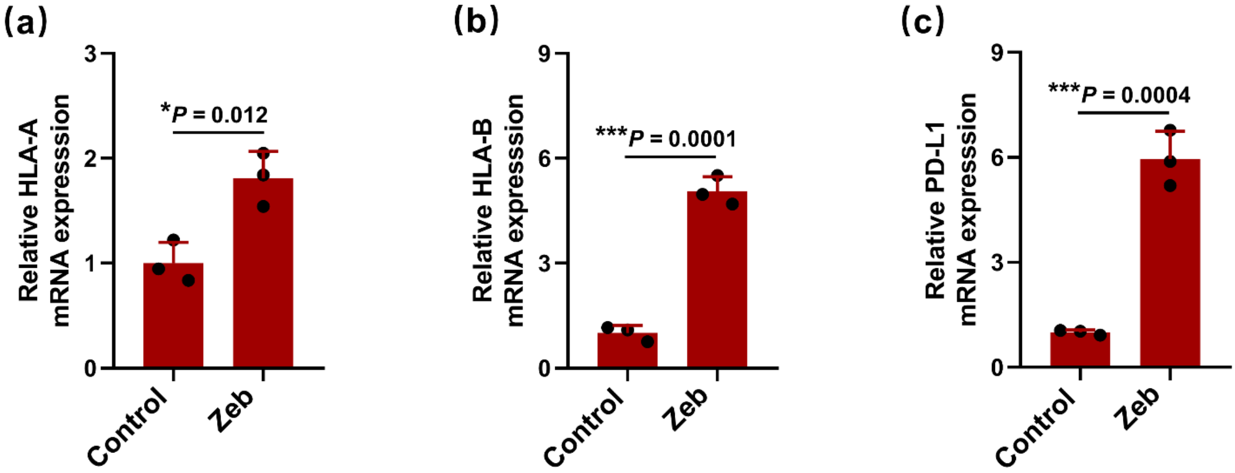

Supplementary Fig. 67 (a) Relative HLA-A mRNA, **(b)** HLA-B mRNA, and **(c)** PD-L1 mRNA expression in HepG2 cells treated with 0.5 mg/mL of Zeb by RT-qPCR assay. Data in **Supplementary Fig. a-c** are presented as mean ± SD, n=3 biologically independent samples in **Supplementary Fig. a-c**. *P* values are calculated by the two-tailed student's t-test in **Supplementary Fig. a-c** as indicated in the figure, *P<0.05 and ***P<0.001.

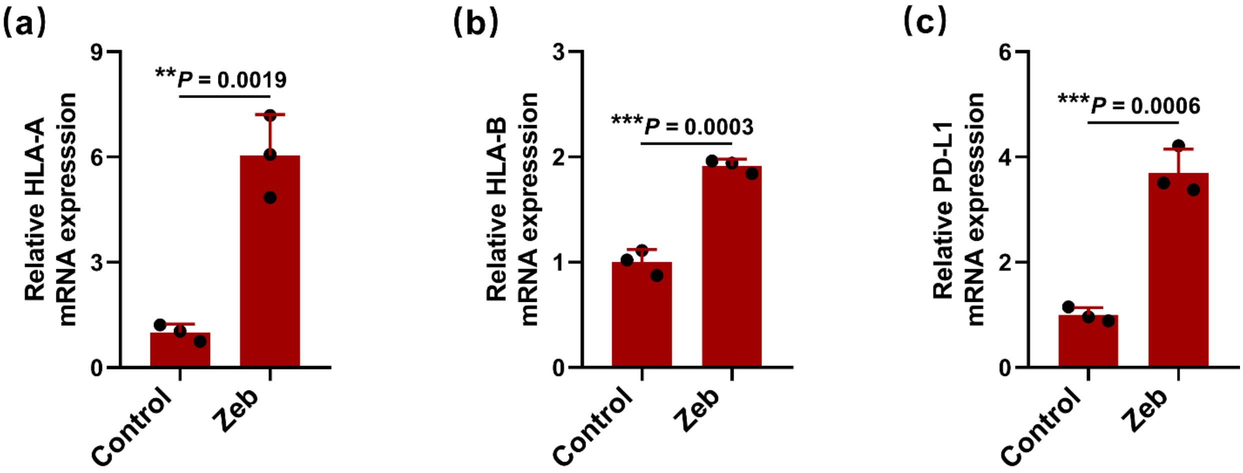

Supplementary Fig. 68 (a) Relative HLA-A mRNA, **(b)** HLA-B mRNA, and **(c)** PD-L1 mRNA expression in A549 cells treated with 0.5 mg/mL of Zeb by RT-qPCR assay. Data in **Supplementary Fig. a-c** are presented as mean ± SD, n=3 biologically independent samples in **Supplementary Fig. a-c**. *P* values are calculated by the two-tailed student's t-test in **Supplementary Fig. a-c** as indicated in the figure, **P<0.01 and ***P<0.001.

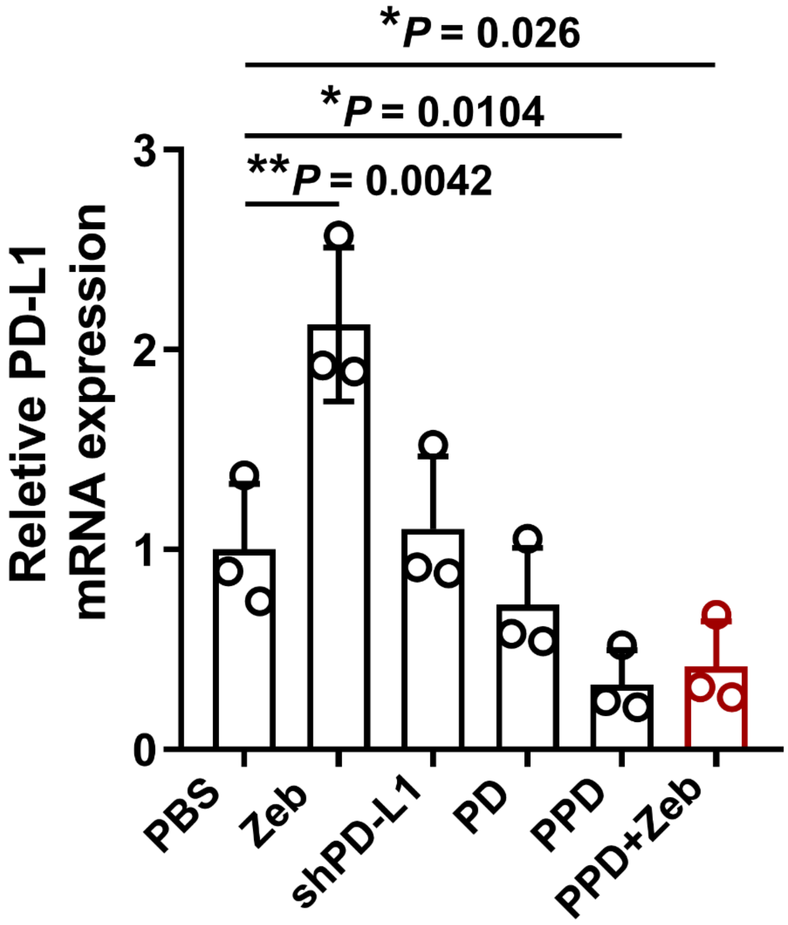

Supplementary Fig. 69 Relative PD-L1 mRNA expression in tumour tissue of B16F10 tumour-bearing mice at the end of the treatment by RT-qPCR assay. Data are presented as mean ± SD, n=3 biologically independent samples. *P* values are calculated by the two-tailed student's t-test as indicated in the figure, *P<0.05 and **P<0.01.

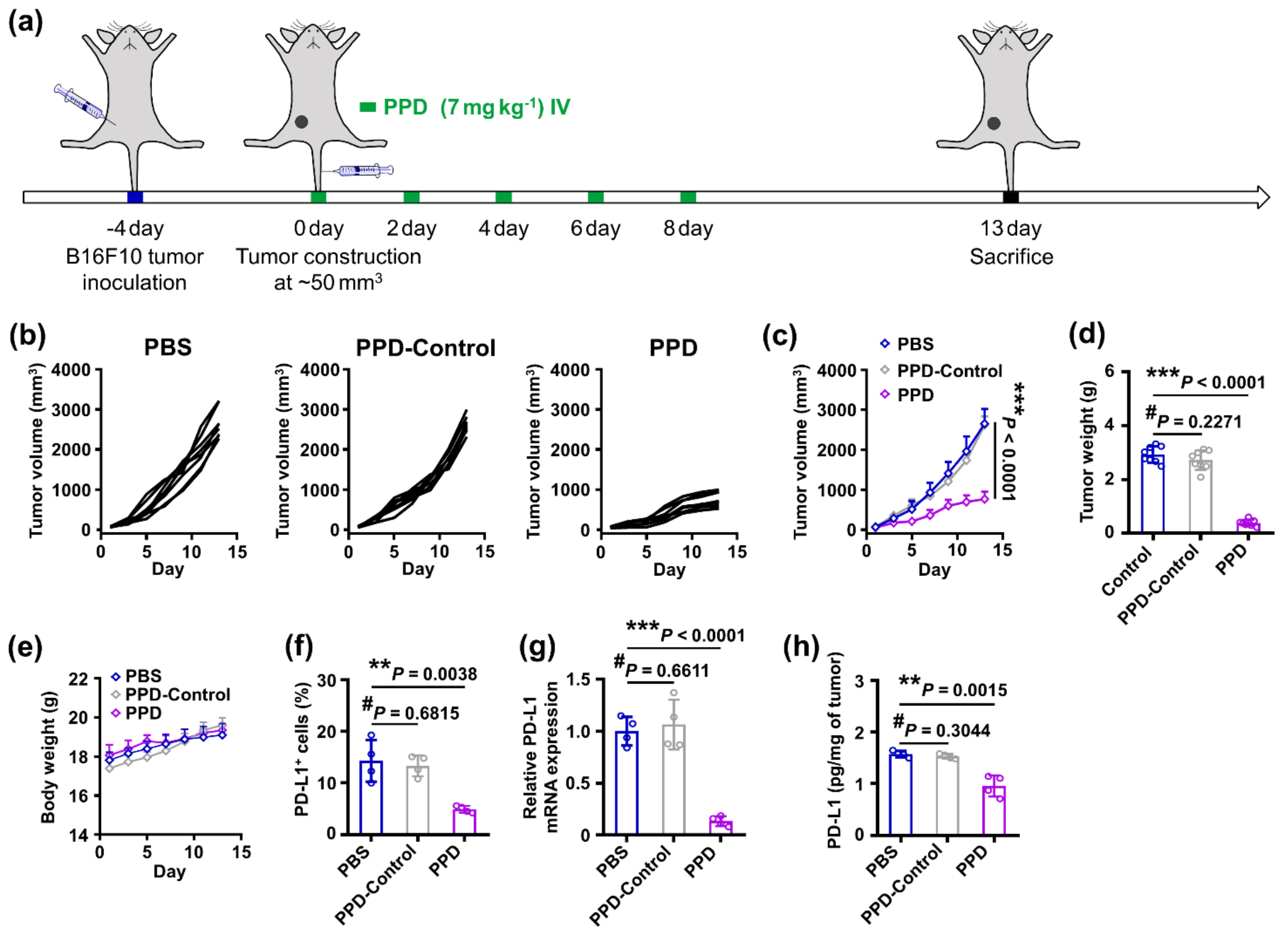

Supplementary Fig. 70 The anti-tumour effect of PPD and PPD-Control in B16F10 tumour-bearing mice. (a) Therapeutic schedule for PPD mediated inhibition of B16F10 tumour growth. **(b)** Individual and **(c)** average tumour growth kinetics in B16F10 tumour-bearing mice receiving different treatments. **(d)** Average tumour weight of B16F10 tumour-bearing mice at the end of treatment, PBS and PPD-Control were served as control groups. **(e)** Average body weight changes of B16F10 bearing mice receiving different treatments. **(f)** Percentages of PD-L1$^+$ cells in tumour tissues of B16F10 tumour-bearing mice at the end of treatment. **(g)** Relative PD-L1 mRNA expression in tumour tissue of B16F10 tumour-bearing mice at the end of the treatment by RT-qPCR assay. **(h)** Levels of PD-L1 protein expression in tumour tissues of B16F10 tumour-bearing mice at the end of the treatment. Data in **Supplementary Fig. c-h** are presented as mean ± SD, n=8 biologically independent samples in **Supplementary Fig. c-e**, n=4 biologically independent samples in **Supplementary Fig. f-h**. *P* values are calculated by the two-tailed student's t-test in **Supplementary Fig. c, d, and f-h** as indicated in the figure, $^{\#}P>0.05$, $^{**}P<0.01$ and $^{***}P<0.001$.

To characterize gene transfection of carrier/shPD-L1 in different cell types in the tumour microenvironment, the levels of PD-L1 expressed on various cell lines in tumours were studied. GFP-B16F10 cells were selected to construct the tumour model. mPEG-b-PLG/PEI-RT/*p*shPD-L1 (**PPD**) ternary complexes were administered intravenously to the tumour-bearing mice (**Supplementary Fig. 72a**). Then the mice were sacrificed and tumours were collected, the levels of PD-L1 in DCs, macrophages and tumour cells in tumour tissue were analyzed by flow cytometry. As shown in **Supplementary Fig. 72b&c**, PD-L1 was also expressed on DCs (CD11c+ MHC II^+) and macrophages ($CD11b^+$ $F4/80^+$) in tumour tissue. Compared with PBS group, the percentages of PD-L1 on DCs and macrophages in PPD group did not change significantly. However, the level of PD-L1 on tumour cells (GFP^+) in PPD group was much lower than that in PBS group (**Supplementary Fig. 72d**). These results suggested that PPD could effectively downregulate the level of PD-L1 on tumour cells instead of DCs and macrophages in the tumour microenvironment.

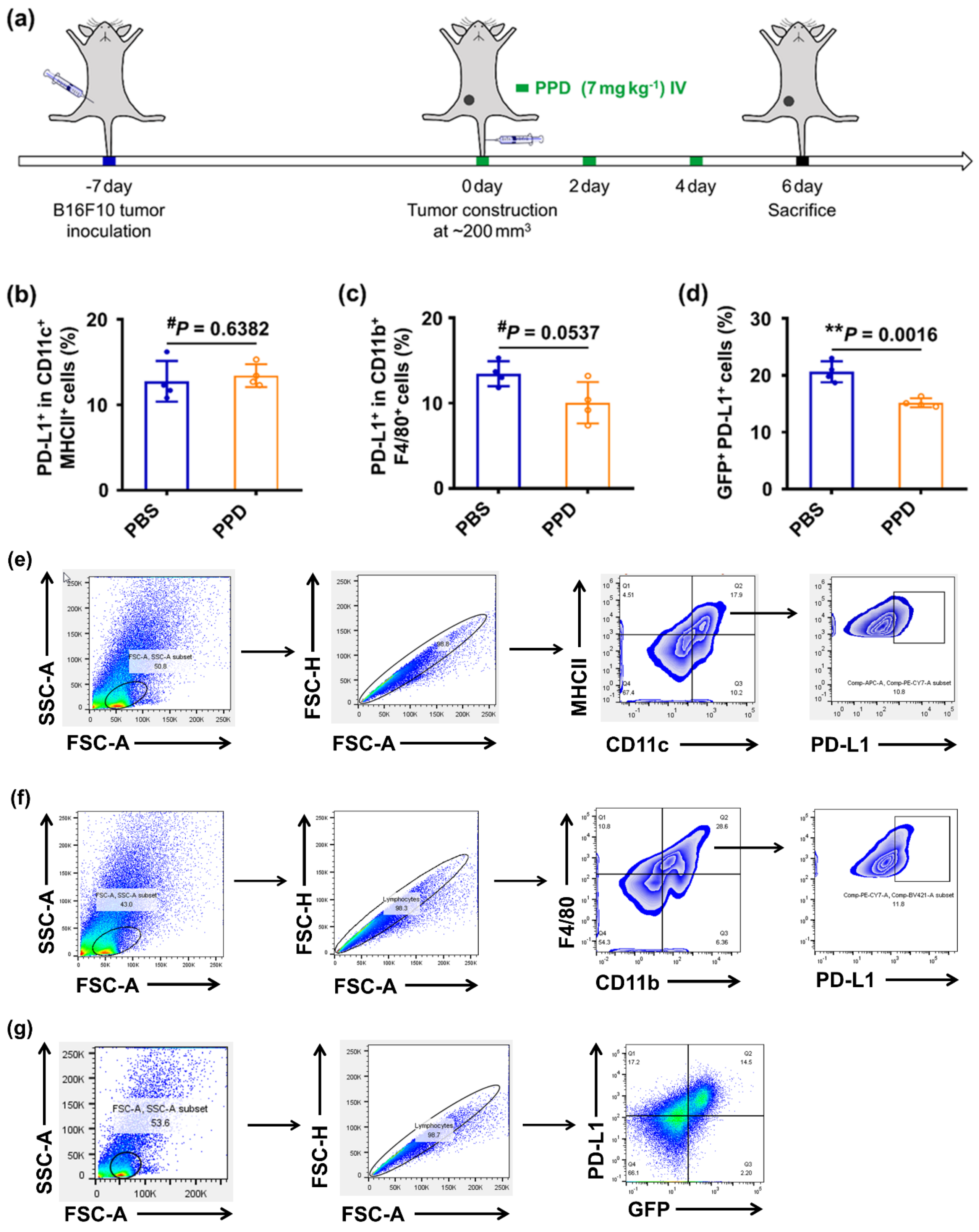

Supplementary Fig. 71 The levels of PD-L1⁺ cells in various cells in tumour tissue of B16F10 tumour-bearing mice receiving PPD treatment. (a) Therapeutic schedule for PPD mediated PD-L1 down regulation in B16F10 tumour-bearing mice. **(b)** Percentages of PD-L1$^+$ cells in DC (CD11c$^+$MHC II$^+$) cells in tumour tissue. **(c)** Percentages of PD-L1$^+$ cells in Macrophage (CD11b$^+$F4/80$^+$) cells in tumour tissue. **(d)** Percentages of GFP$^+$PD-L1$^+$ cells in tumour tissue. Data

in **Supplementary Fig. b-d** are presented as mean ± SD, n=3 biologically independent samples in **Supplementary Fig. b-d**. *P* values are calculated by the two-tailed student's t-test in **Supplementary Fig. b-d** as indicated in the figure, $^{\#}P$>0.05 and $^{**}P$<0.01. **(e)** Flow cytometry gating strategy for the analysis of PD-L1$^+$ cells in CD11c$^+$ MHCII$^+$ cells within tumor tissue of B16F10 tumor-bearing mice receiving PPD treatment. (f) Flow cytometry gating strategy for the analysis of PD-L1$^+$ cells in CD11b$^+$ F4/80$^+$ cells within tumor tissue of B16F10 tumor-bearing mice receiving PPD treatment. **(g)** Flow cytometry gating strategy for the analysis of GFP$^+$ PD-L1$^+$ cells in tumor tissue of B16F10 tumor-bearing mice receiving PPD treatment.

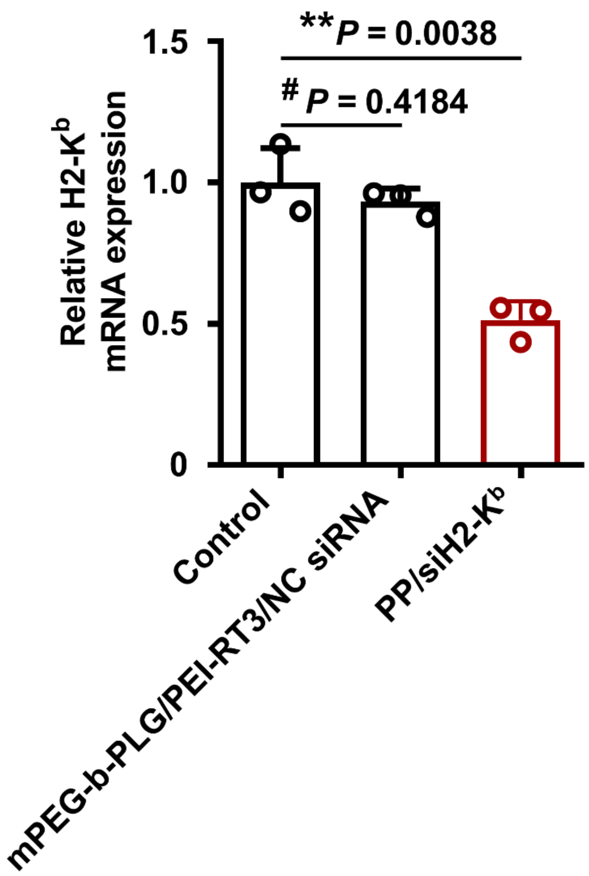

Supplementary Fig. 72 Relative H2-K^b mRNA expression in B16F10 cells treated with PP/siH2-K^b complexes by RT-qPCR assay. Data are presented as mean ± SD, n=3 biologically independent samples. *P* values are calculated by the two-tailed student's t-test as indicated in the figure, $^{\#}P$>0.05 and $^{**}P$<0.01.

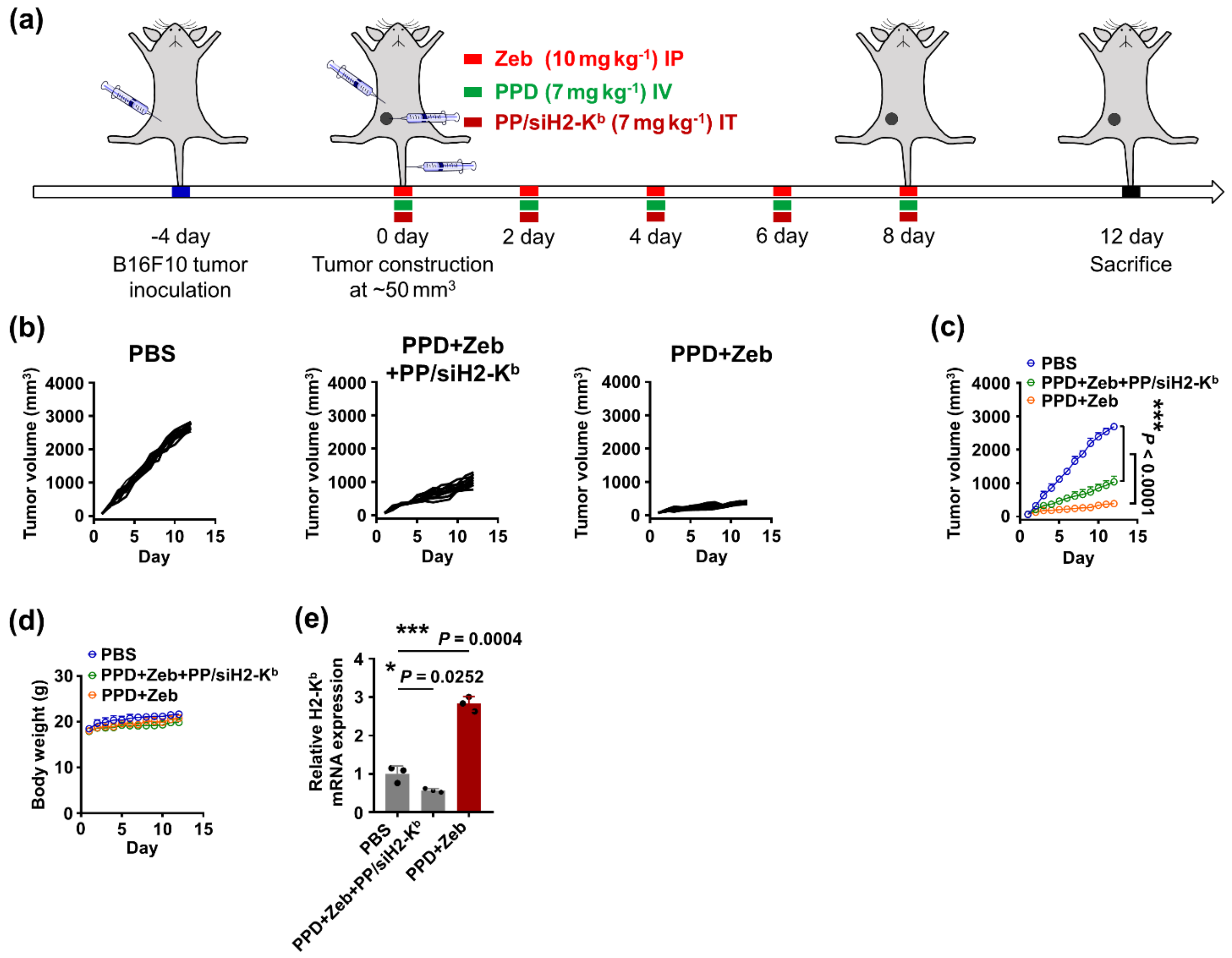

Supplementary Fig. 73 PPD plus Zeb accompanying with MHC I depletion mediated tumour therapy in B16F10 tumour-bearing mice. **(a)** Therapeutic schedule for PPD plus Zeb accompanying with MHC I depletion mediated B16F10 tumour-bearing mice. **(b)** Individual and **(c)** average tumour growth kinetics in B16F10 tumour-bearing mice receiving various treatments. **(d)** Average body weight changes of B16F10 tumour-bearing mice receiving different treatments. **(e)** Relative H2-K^{b} mRNA in B16F10 tumour-bearing mice after treatment by RT-qPCR assay. Data in **Supplementary Fig. c**-e are presented as mean ± SD, n=8 biologically independent samples in **Supplementary Fig. c** and **d**, n=3 biologically independent samples in **Supplementary Fig. e**. *P* values are calculated by the two-tailed student's t-test in **Supplementary Fig. c** and **e** as indicated in the figures, **P*<0.05, ***P*<0.01, and ****P*<0.001.

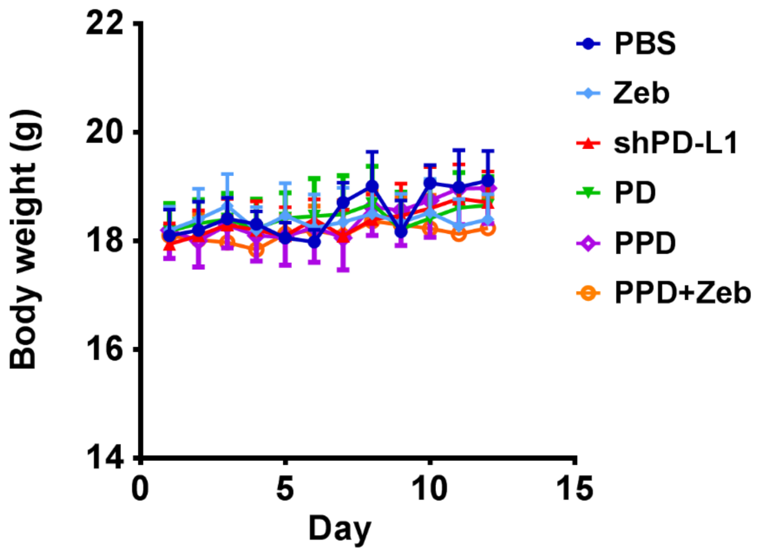

Supplementary Fig. 74 Average body weight changes of B16F10 bearing mice treated with different formulations. Data are presented as mean ± SD, n=10 biologically independent samples.

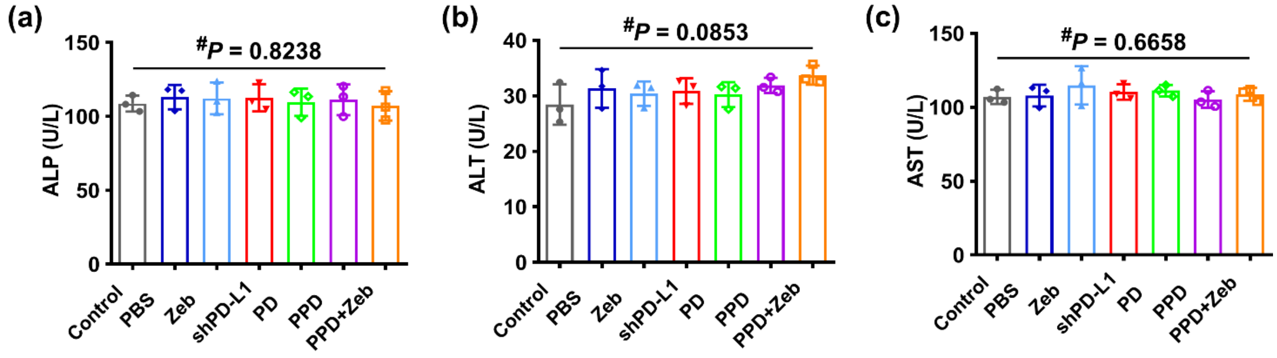

Supplementary Fig. 75 **T**he blood levels of liver function markers including **(a)** alkaline phosphatase (ALP), **(b)** alanine aminotransferase (ALT), and **(c)** aspartate aminotransferase (AST) of C57/c mice after treated with different formulations (PBS, Zeb, shPD-L1, PD, PPD, and PPD plus Zeb). Age- and weight-matched healthy C57 mice were used as control. Data are presented as mean ± SD, n=3 biologically independent samples in **Supplementary Fig. a-c**. *P* values are calculated by the two-tailed student's t-test as indicated in the figure, $^{\#}P$>0.05.

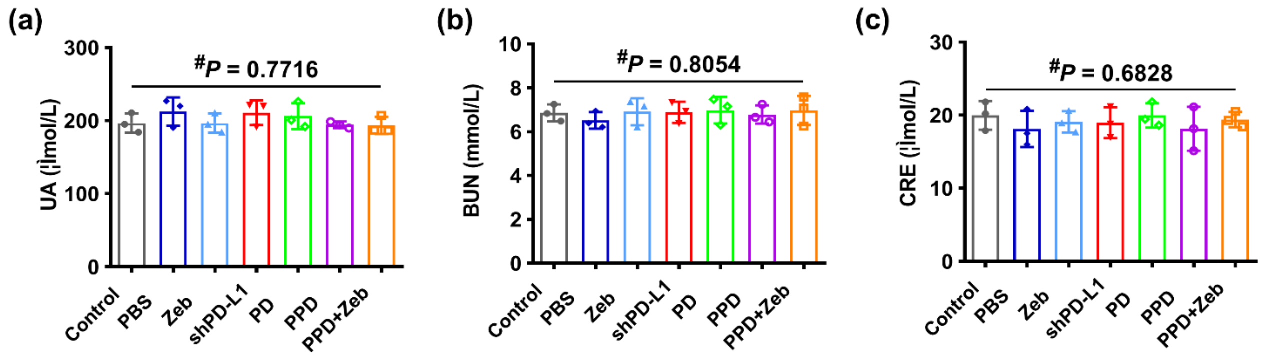

Supplementary Fig. 76 The blood levels of renal function markers including **(a)** uric acid (UA), **(b)** blood urea nitrogen (BUN), and **(c)** creatinine (CRE) of C57/c mice after treated with different formulations (PBS, Zeb, shPD-L1, PD, PPD, and PPD plus Zeb). Age- and weight-matched healthy C57/c mice were used as control. Data are presented as mean ± SD, n=3 biologically independent samples in **Supplementary Fig. a-c**. *P* values are calculated by the two-tailed student’s t-test as indicated in the figure, $^{\#}P>0.05$.

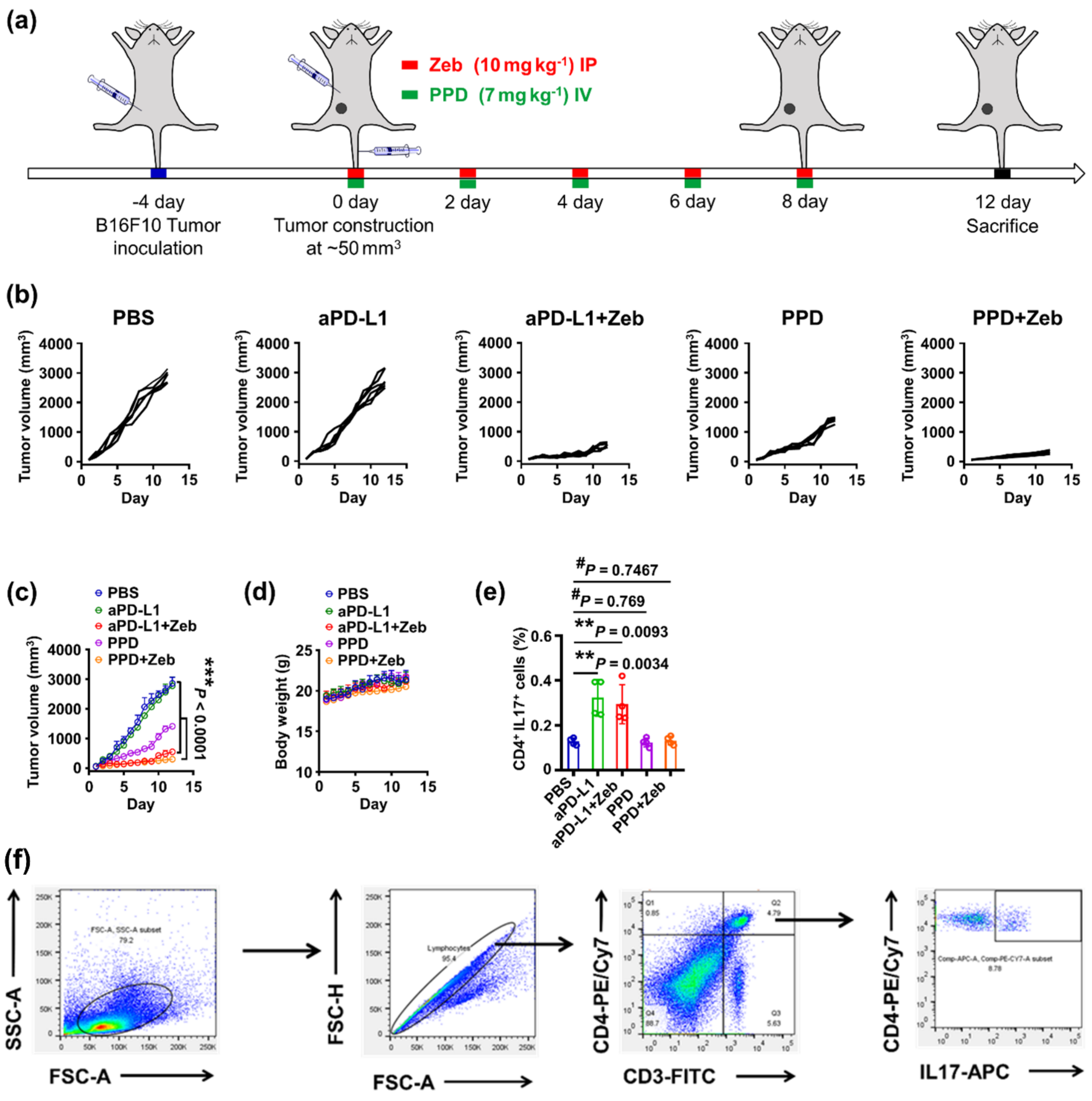

Supplementary Fig. 77 PPD plus Zeb or aPD-L1 plus Zeb mediated inhibition of B16F10 tumour growth. (a) Therapeutic schedule for PPD plus Zeb or aPD-L1 plus Zeb mediated inhibition of B16F10 tumour growth. **(b)** Individual and **(c)** average tumour growth kinetics in B16F10 tumour-bearing mice receiving various treatments. (d) Average body weight changes of B16F10 tumour-bearing mice receiving different treatments. **(e)** Percentages of Th17 cells in the splenocytes of B16F10 tumour-bearing mice receiving various treatments on day 12. Data in **Supplementary Fig. c**-e are presented as mean ± SD, n=6 biologically independent samples in **Supplementary Fig. c** and **d**, n=4 biologically independent samples in **Supplementary Fig. e**. *P* values are calculated by the two-tailed student's t-test in **Supplementary Fig. c** and **e** as indicated in the figures, $^{\#}P>0.05$,

P<0.01, and ***P<0.001. **(f) Flow cytometry gating strategy for the analysis of Th17 cells in the splenocytes.

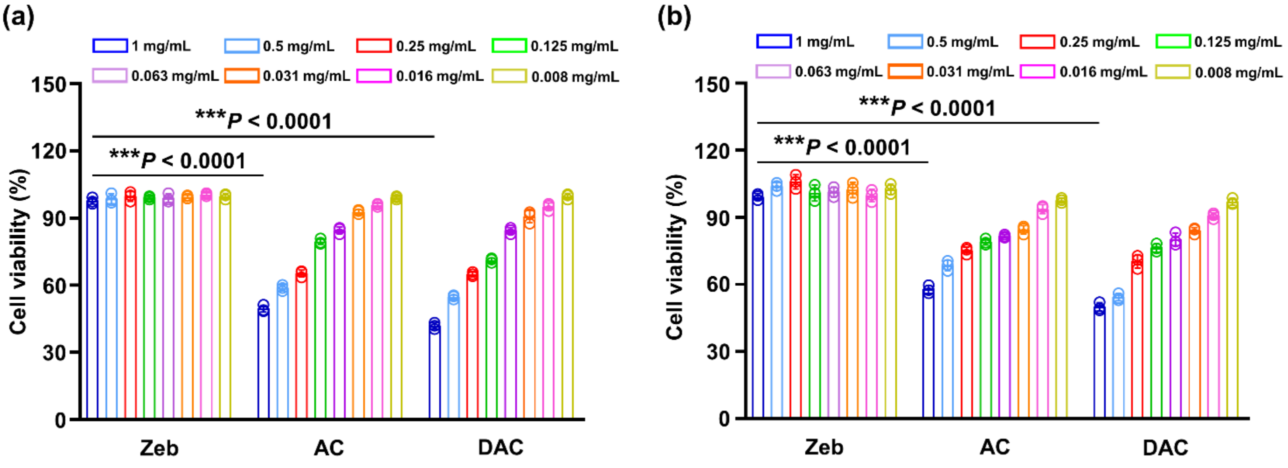

Supplementary Fig. 78 (i.e.,) The cell viability of Zeb, AC, and DAC at various concentrations in **(a)** B16F10 and **(b)** 4T1 cells. Data in **Supplementary Fig. a** and **b** are presented as mean ± SD, n=3 biologically independent samples in **Supplementary Fig. a** and **b**. *P* values are calculated by the two-tailed student's t-test in **Supplementary Fig. a** and **b** as indicated in the figures, ***P<0.0001.

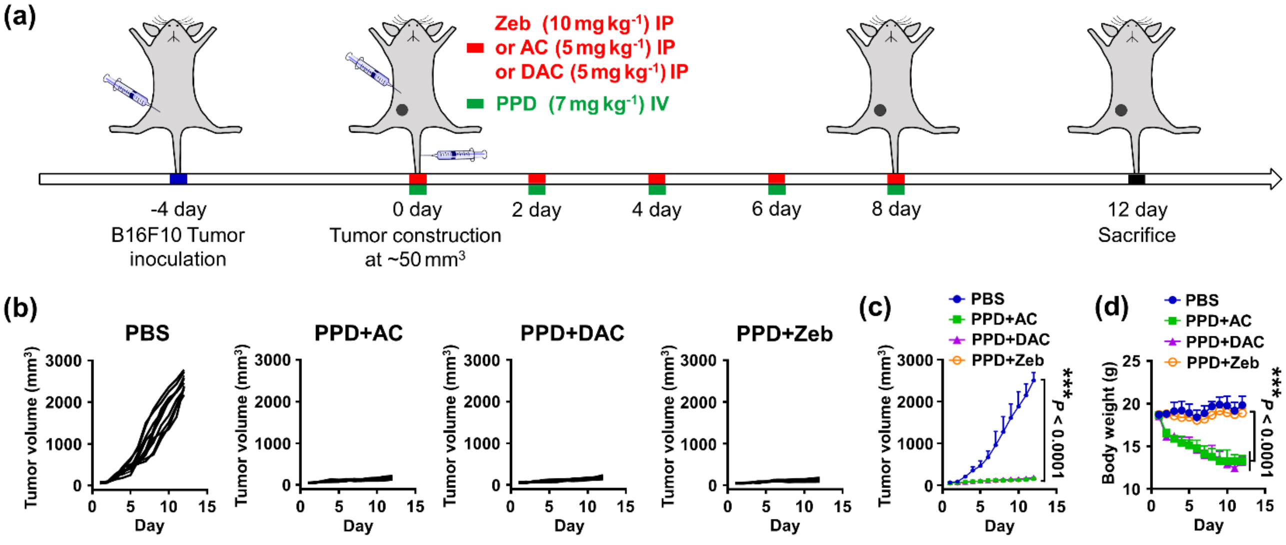

Supplementary Fig. 79 PPD plus Zeb, AC or DAC mediated inhibition of B16F10 tumour growth. (a) Therapeutic schedule for PPD plus DNMTi mediated B16F10 tumour-bearing mice. **(b)** Individual and **(c)** average tumour growth kinetics in B16F10 tumour-bearing mice receiving various treatments. **(d)** Average body weight changes of B16F10 tumour-bearing mice receiving different treatments. Data in **Supplementary Fig. c** and **d** are presented as mean ± SD, n=10 biologically

independent samples in **Supplementary Fig. c** and **d**, *P* values are calculated by the two-tailed student's t-test in **Supplementary Fig. c** and **d** as indicated in the figures, ****P*<0.001.

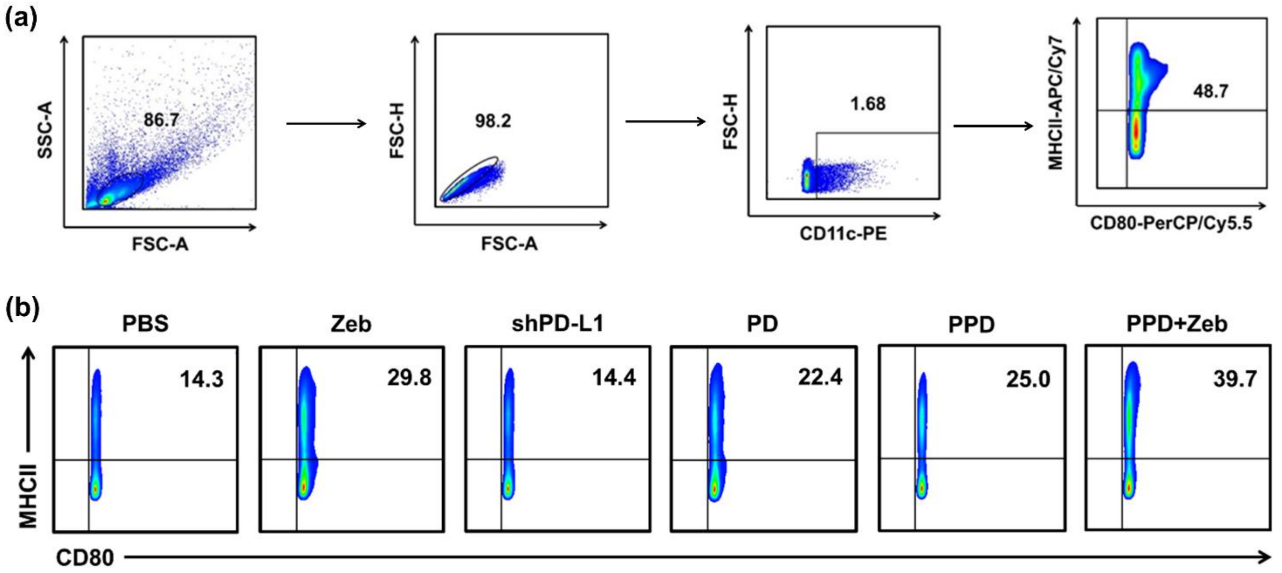

Supplementary Fig. 80 (a) Flow cytometry gating strategy for the analysis of DC maturation *in vivo*. **(b)** Representative flow cytometric analysis of $CD80^+$ $MHCII^+$ dendritic cells gating on $CD11c^+$ cells in lymph nodes of B16F10 tumour-bearing mice after treatment. Additional description: The gating strategies of other kinds of cells including $CD3^+CD8^+$, $CD3^+CD4^+$, M2-type macrophages, MDSCs, Tregs and PD-L1$^+$ cells were similar with the above strategies, and the differences were merely the markers expressed inside on the surface of the cell lines.

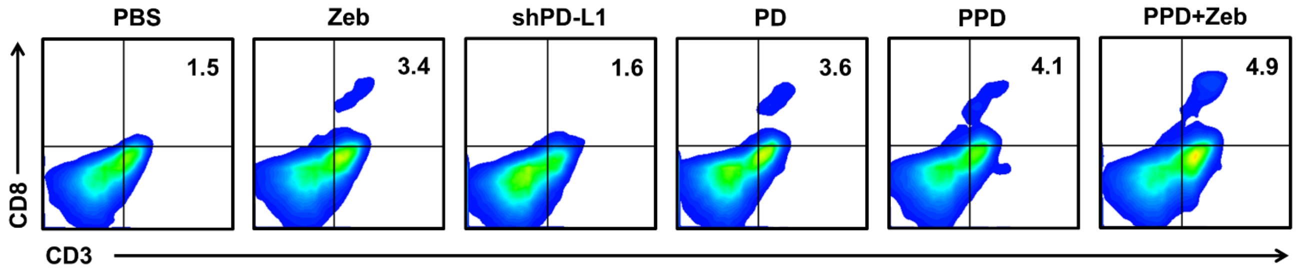

Supplementary Fig. 81 Representative flow cytometric analysis of $CD3^+$ $CD8^+$ T cells gating on $CD45^+$ cells in tumour tissue of B16F10 tumour-bearing mice after treatment.

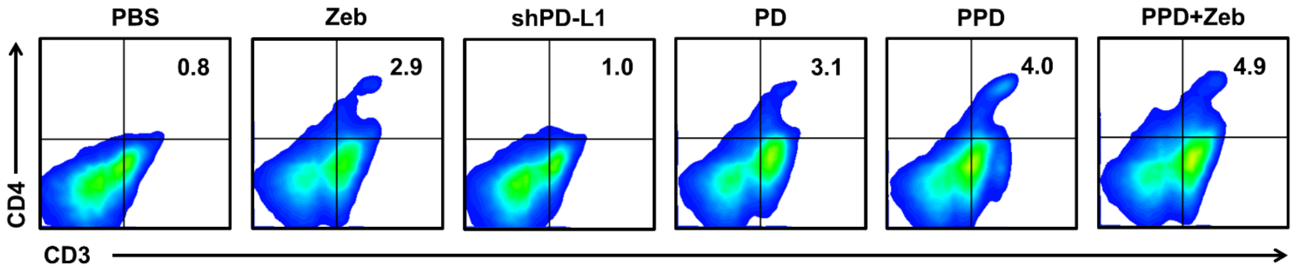

Supplementary Fig. 82 Representative flow cytometric analysis of $CD3^+$ $CD4^+$ T cells gating on $CD45^+$ cells in tumour tissue of B16F10 tumour-bearing mice after treatment.

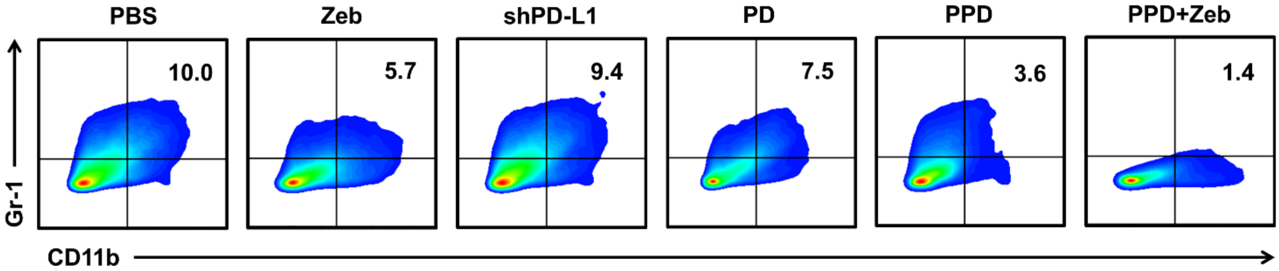

Supplementary Fig. 83 Representative flow cytometric analysis of $CD11b^+$ $Gr\text{-}1^+$ cells gating on $CD45^+$ cells in tumours of B16F10 tumour-bearing mice after treatment.

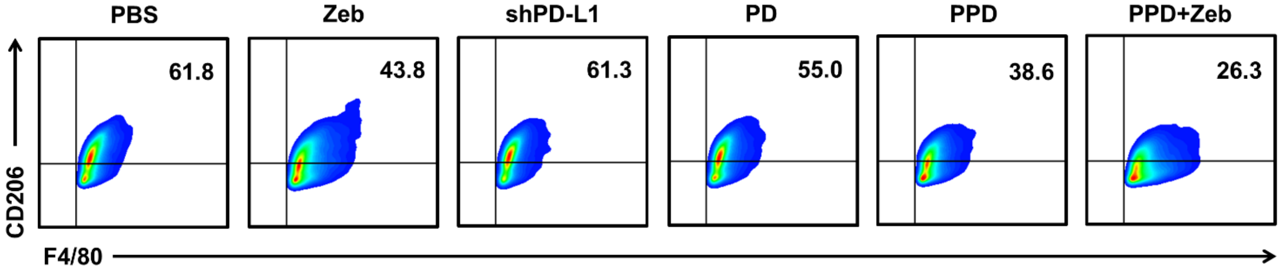

Supplementary Fig. 84 Representative flow cytometric analysis of $F4/80^+$ $CD206^+$ cells gating $CD11b^+$ cells in tumours of B16F10 tumour-bearing mice after treatment.

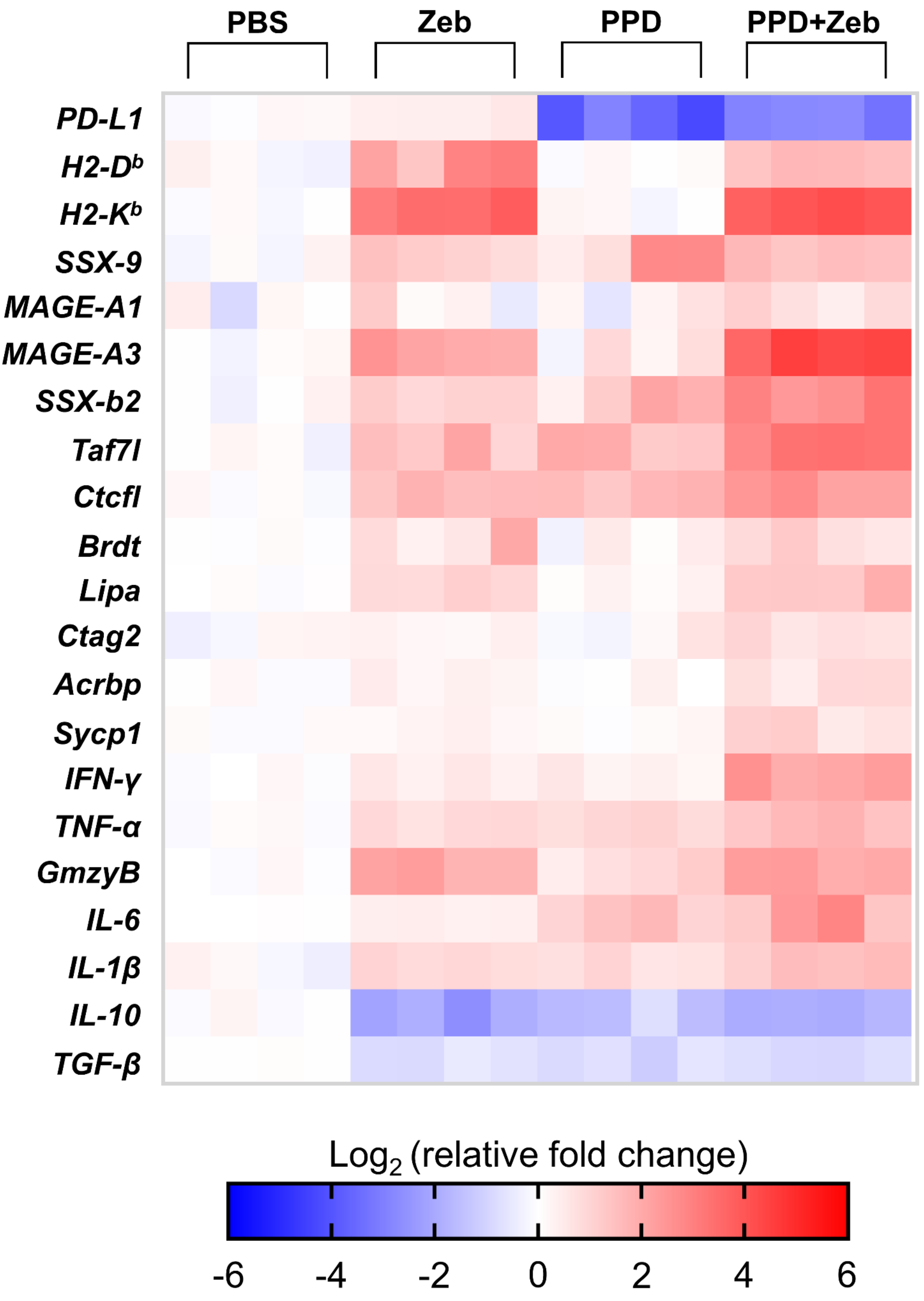

Supplementary Fig. 85 Heatmap of relative mRNA expression of PD-L1, MHC I (H2-D^b and H2-K^b), CTAs (SSX-9, SSX-b2, MAGE-A1, MAGE-A3, Tf7al, Ctcfl, Brdt, Lipa, Ctag2, Acrbp, and

Sycp1), and immunoactivated cytokines (IFN-γ, TNF-α, GmzyB, IL-6, and IL-1β) and immunosuppressive cytokines (IL-10 and TGF-β) in tumour tissues of B16F10 tumour-bearing mice at the end of treatment.

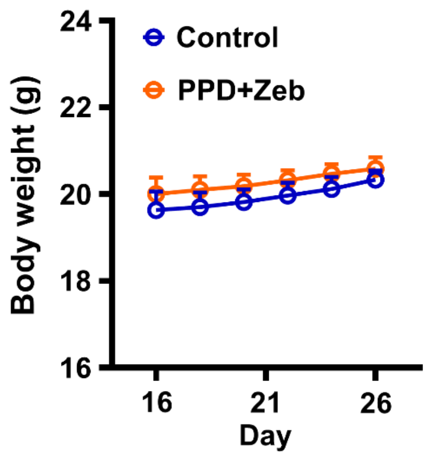

Supplementary Fig. 86 Average body weight changes of distant B16F10 tumour-bearing mice receiving PPD plus Zeb treatment. Data are presented as mean ± SD, n=6 biologically independent samples.

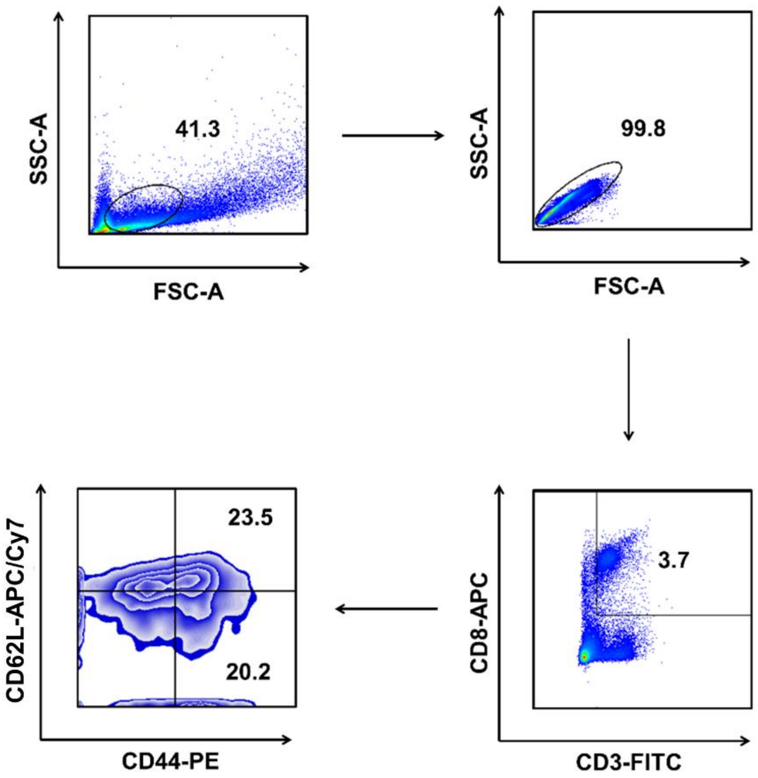

Supplementary Fig. 87 Flow cytometry gating strategy for the analysis of TEMs ($CD44^+CD62L^-$) within $CD3^+CD8^+$ T cells *in vivo*.

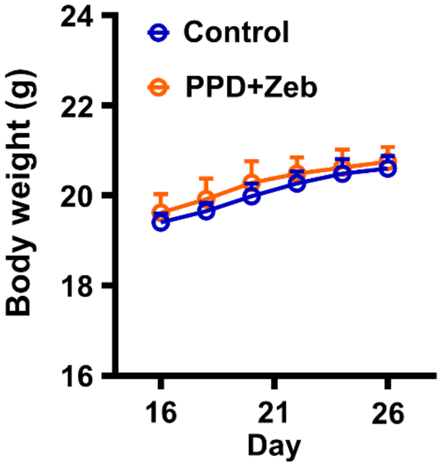

Supplementary Fig. 88 Average body weight changes of distant MC38 tumour-bearing mice receiving PPD plus Zeb treatment. Data are presented as mean ± SD, n=6 biologically independent samples.

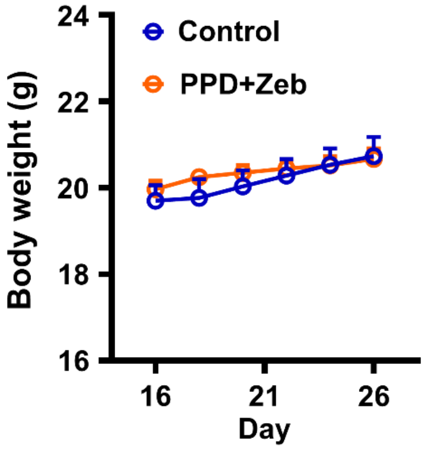

Supplementary Fig. 89 Average body weight changes of distant LLC tumour-bearing mice receiving PPD plus Zeb treatment. Data are presented as mean ± SD, n=6 biologically independent samples.

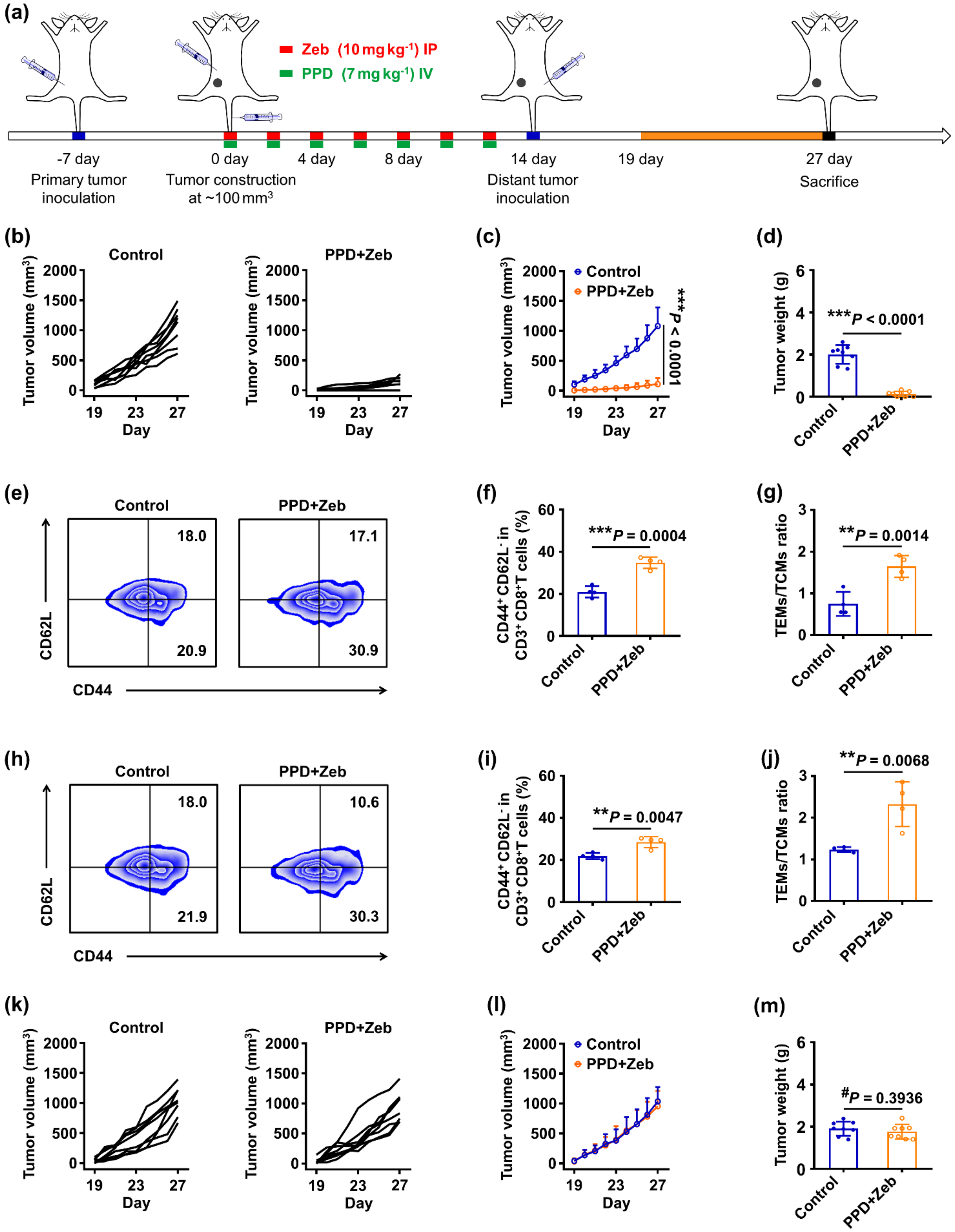

Supplementary Fig. 90 PPD combined with Zeb inhibited 4T1 tumour relapse and initiated tumour specific immune memory effect. (a) Therapeutic schedule for PPD plus Zeb mediated inhibition of tumour relapse. **(b)** Individual and **(c)** average tumour growth kinetics in distant 4T1

tumour-bearing mice receiving PPD plus Zeb treatment. Age- and body-weight-matched healthy mice were used as control. **(d)** Average tumour weight of distant 4T1 tumour-bearing mice receiving PPD plus Zeb treatment. **(e)** Representative flow cytometric analysis of TEM ($CD44^+$ $CD62L^-$) and TCM ($CD44^+$ $CD62L^{+)}$ cells gating on $CD8^+$ T cells in spleen of 4T1 tumour-bearing mice at the end of treatment. Relative quantification of **(f)** TEM cells gating on $CD8^+$T cells and **(g)** ratio of TEMs/TCMs gating on $CD8^+$T cells in the spleen of 4T1 tumour-bearing mice at the end of treatment. **(h)** Representative flow cytometric analysis of TEM ($CD44^+$ $CD62L^-$) and TCM ($CD44^+$ $CD62L^{+)}$ cells gating on $CD4^+$ T cells in spleen of 4T1 tumour-bearing mice at the end of treatment. Relative quantification of **(i)** TEM cells gating on $CD4^+$T cells and **(j)** ratio of TEMs/TCMs gating on $CD4^+$T cells in the spleen of 4T1 tumour-bearing mice at the end of treatment. **(k)** Individual and **(l)** average tumour growth kinetics in distant CT26 (murine colon carcinoma) tumour-bearing mice receiving PPD plus Zeb treatment. Age- and body-weight-matched healthy mice were used as control. **(m)** Average tumour weight of distant CT26 tumour-bearing mice receiving PPD plus Zeb treatment. Data in **Supplementary Fig. c, d, f, g, i, j, l, m** are presented as mean ± SD, n=8 biologically independent samples in **Supplementary Fig. c, d, l** and **m**, n=4 biologically independent samples in **Supplementary Fig. f, g, i** and **j**. *P* values are calculated by the two-tailed student's t-test in **Supplementary Fig. c, d, f, g, i, j, m** as indicated in the figures. $^{\#}P>0.05$, $^{**}P<0.01$, and $^{***}P<0.001$.

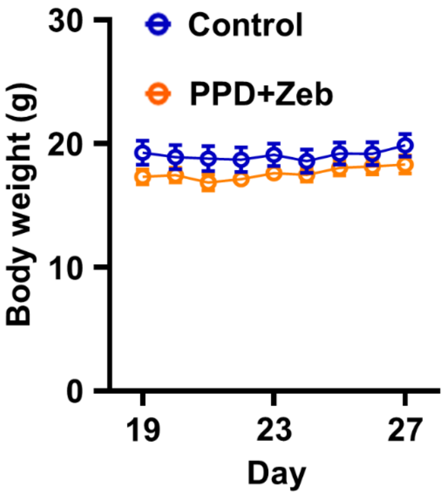

Supplementary Fig. 91 Average body weight changes of distant 4T1 tumour-bearing mice receiving PPD plus Zeb treatment. Data are presented as mean ± SD, n=8 biologically independent samples.

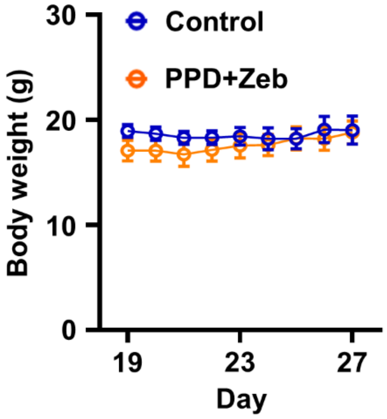

Supplementary Fig. 92 Average body weight changes of distant CT26 tumour-bearing mice receiving PPD plus Zeb treatment. Data are presented as mean ± SD, n=8 biologically independent samples.

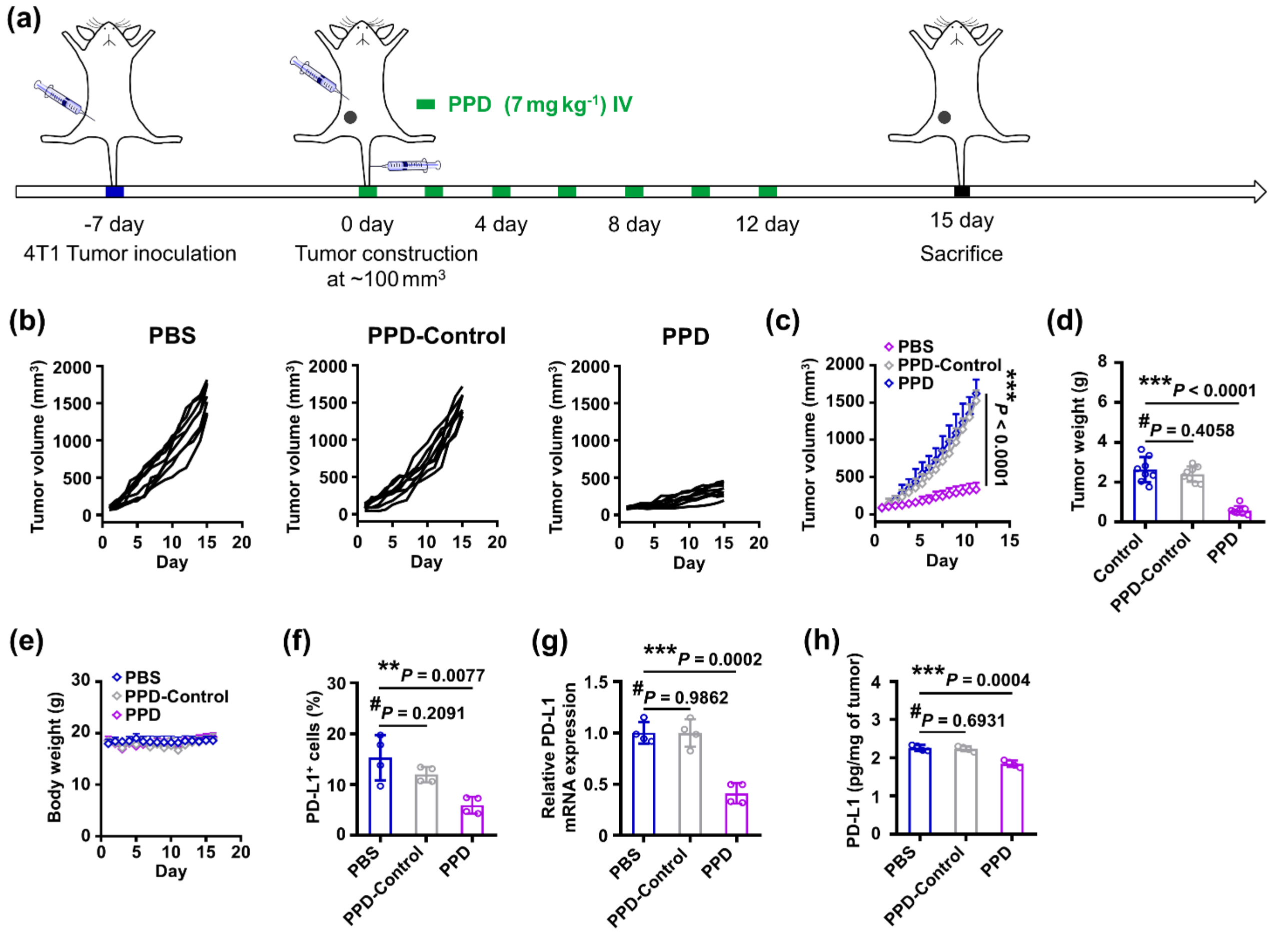

Supplementary Fig. 93 The anti-tumour effect of PPD and PPD-Control in 4T1 tumour-bearing mice. **(a)** Therapeutic schedule for PPD mediated inhibition of 4T1 tumour growth. **(b)** Individual and **(c)** average tumour growth kinetics in 4T1 tumour-bearing mice receiving different treatments. (d) Average tumour weight of 4T1 tumour-bearing mice at the end of treatment, PBS and PPD-Control were served as control groups. **(e)** Average body weight changes of 4T1 bearing mice

receiving different treatments. **(f)** Percentages of PD-L1$^+$ cells in tumour tissues of 4T1 tumour-bearing mice at the end of treatment. **(g)** Relative PD-L1 mRNA expression in tumour tissue of 4T1 tumour-bearing mice at the end of the treatment by RT-qPCR assay. **(h)** Levels of PD-L1 protein expression in tumour tissues of 4T1 tumour-bearing mice at the end of the treatment. Data in **Supplementary Fig. c-h** are presented as mean ± SD, n=8 biologically independent samples in **Supplementary Fig. c-e**. n=4 biologically independent samples in **Supplementary Fig. f-h**. *P* values are calculated by the two-tailed student's t-test in **Supplementary Fig. c, d,** and **f-h** as indicated in the figures. $^{\#}P>0.05$, $^{**}P<0.01$, and $^{***}P<0.001$.

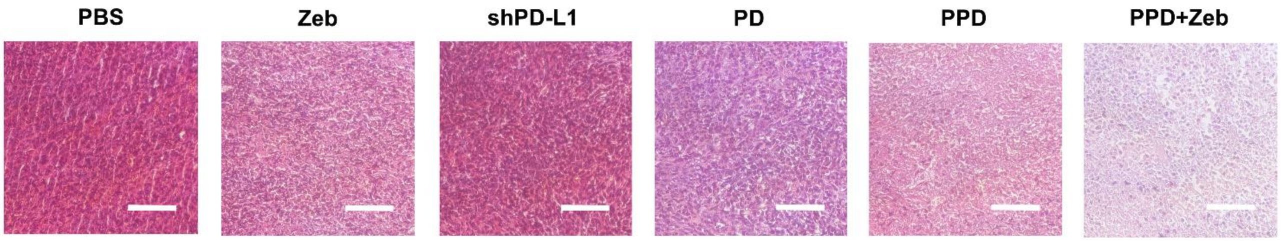

Supplementary Fig. 94 Representative H&E stained images of tumour tissues of 4T1 tumour-bearing mice receiving different treatments. Scale bar: 200 μm. A representative image of three biologically independent samples from each group is shown in the figure.

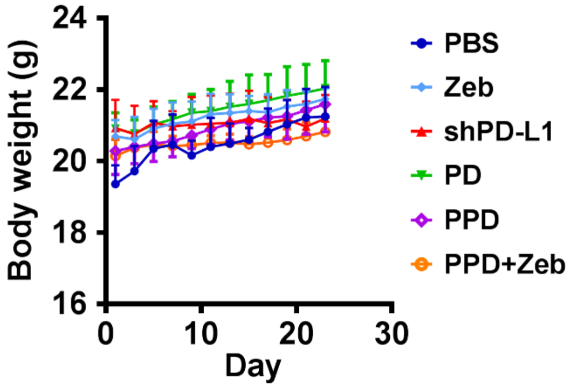

Supplementary Fig. 95 Average body weight changes of 4T1 bearing mice during treatment. Data are presented as mean ± SD, n=10 biologically independent samples.

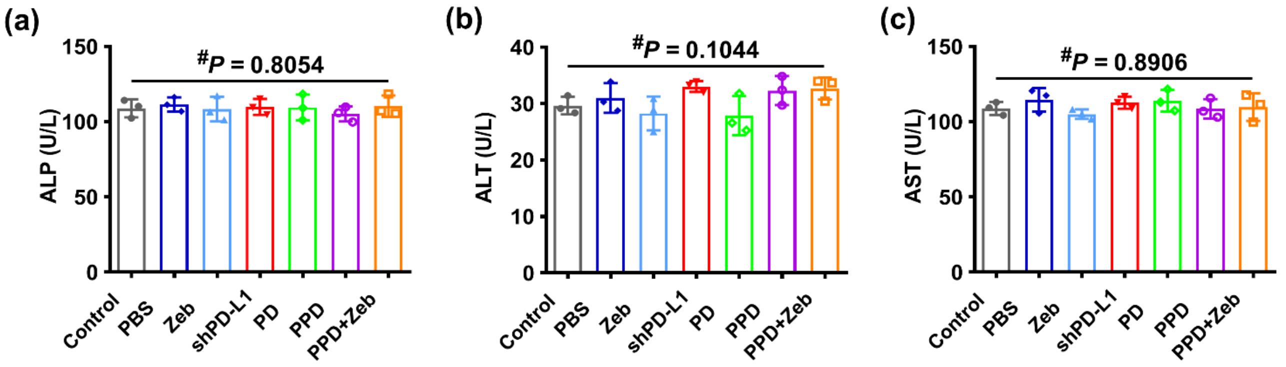

Supplementary Fig. 96 The blood levels of liver function markers including **(a)** ALP, **(b)** ALT, and **(c)** AST of BABL/c mice after treated with different formulations (PBS, Zeb, shPD-L1, PD, PPD, and PPD plus Zeb). Age- and weight-matched healthy BABL/c mice were used as control. Data are presented as mean ± SD, n=3 biologically independent samples in **Supplementary Fig. a-c**. *P* values are calculated by the two-tailed student's t-test as indicated in the figure, $^{\#}P>0.05$.

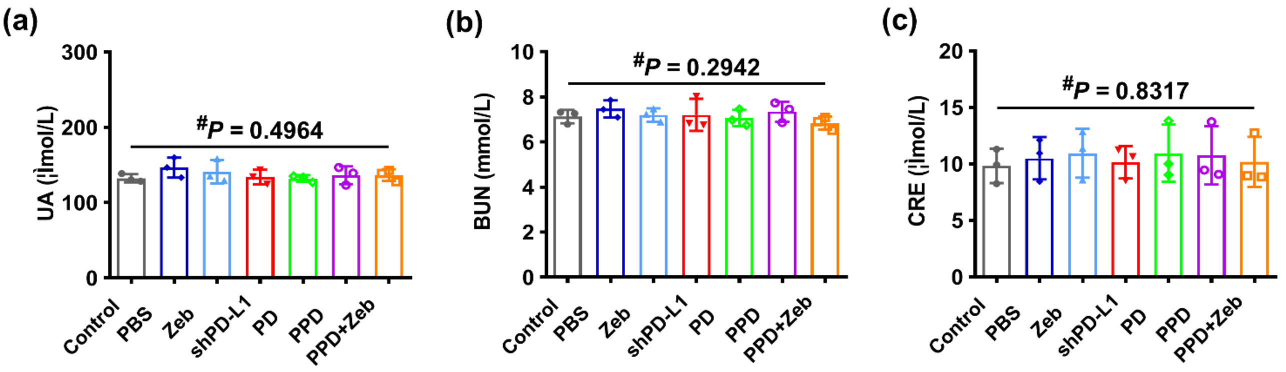

Supplementary Fig. 97 The blood levels of renal function markers including **(a)** uric acid (UA), **(b)** blood urea nitrogen (BUN), and **(c)** creatinine (CRE) of BABL/c mice after treated with different formulations (PBS, Zeb, shPD-L1, PD, PPD, and PPD plus Zeb). Age- and body-weight-matched healthy BABL/c mice were used as control. Data are presented as mean ± SD, n=3 biologically independent samples in **Supplementary Fig. a-c**. *P* values are calculated by the two-tailed student's t-test as indicated in the figure, $^{\#}P>0.05$.

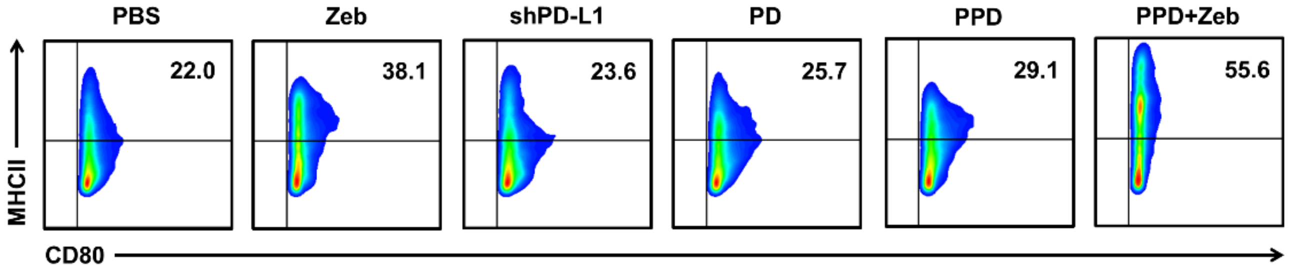

Supplementary Fig. 98 Representative flow cytometric analysis of $CD80^{+}MHCII^{+}$ dendritic cells gating on $CD11c^{+}$ cells in lymph nodes of 4T1 tumour-bearing mice after treatment.

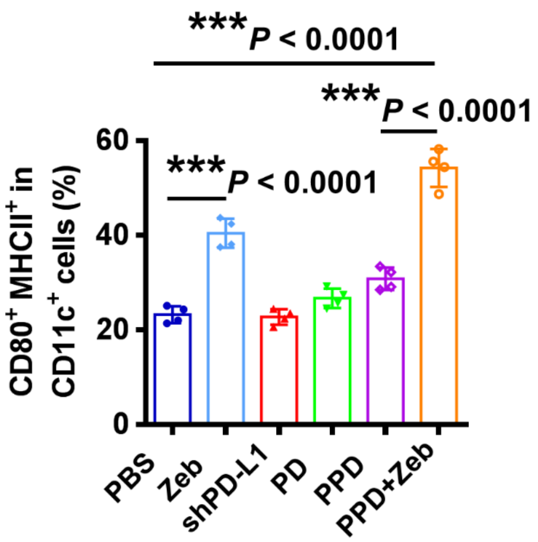

Supplementary Fig. 99 The percentage of $CD80^+$ $MHCII^+$ dendritic cells gating on $CD11c^+$ cells in lymph nodes of 4T1 tumour-bearing mice after treatment. Data are presented as mean ± SD, n=4 biologically independent samples. *P* values are calculated by the two-tailed student's t-test as indicated in the figure. ***P*<0.01 and ****P*<0.001.

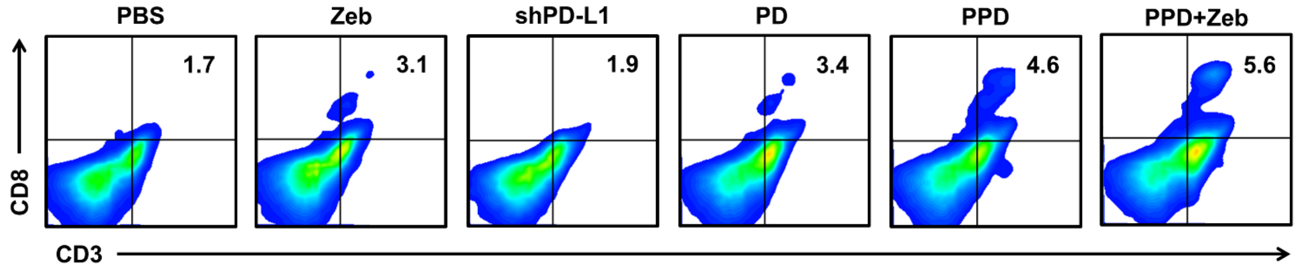

Supplementary Fig. 100 Representative flow cytometric analysis of $CD3^+CD8^+$ T cells gating on $CD45^+$ cells in tumour of 4T1 tumour-bearing mice after treatment.

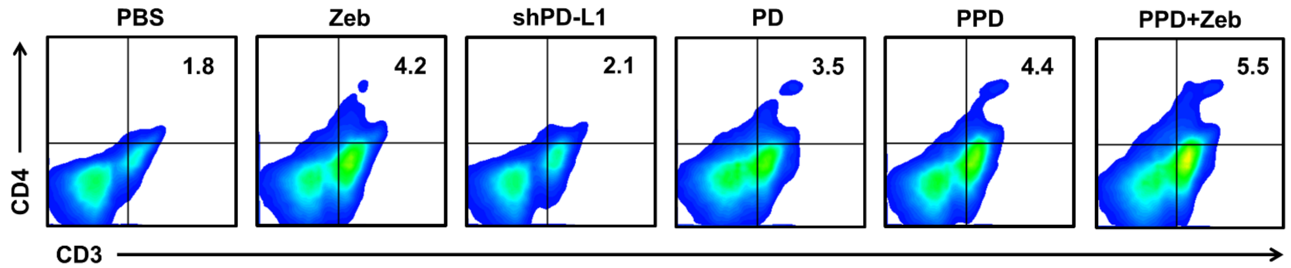

Supplementary Fig. 101 Representative flow cytometric analysis of $CD3^+CD4^+$ T cells gating on $CD45^+$ cells in tumours of 4T1 tumour-bearing mice after treatment.

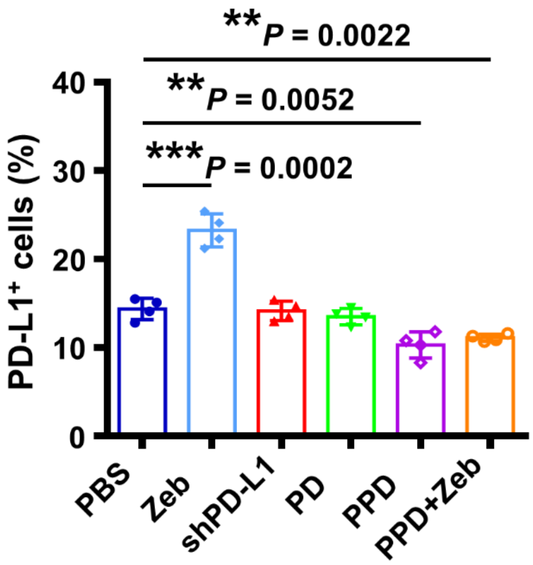

Supplementary Fig. 102 Percentage of PD-L1^{+} cells in tumour tissues of 4T1 tumour-bearing mice receiving different treatments. Data are presented as mean ± SD, n=4 biologically independent samples. *P* values are calculated by the two-tailed student's t-test as indicated in the figure, ***P*<0.01 and ****P*<0.001.

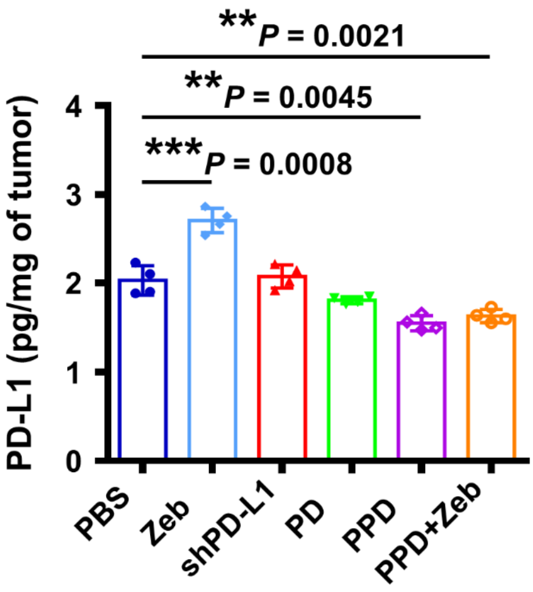

Supplementary Fig. 103 Levels of PD-L1 protein expression in tumour tissues of 4T1 tumour-bearing mice receiving different treatments. Data are presented as mean ± SD, n=4 biologically independent samples. *P* values are calculated by the two-tailed student's t-test as indicated in the figure, ***P*<0.01 and ****P*<0.001.

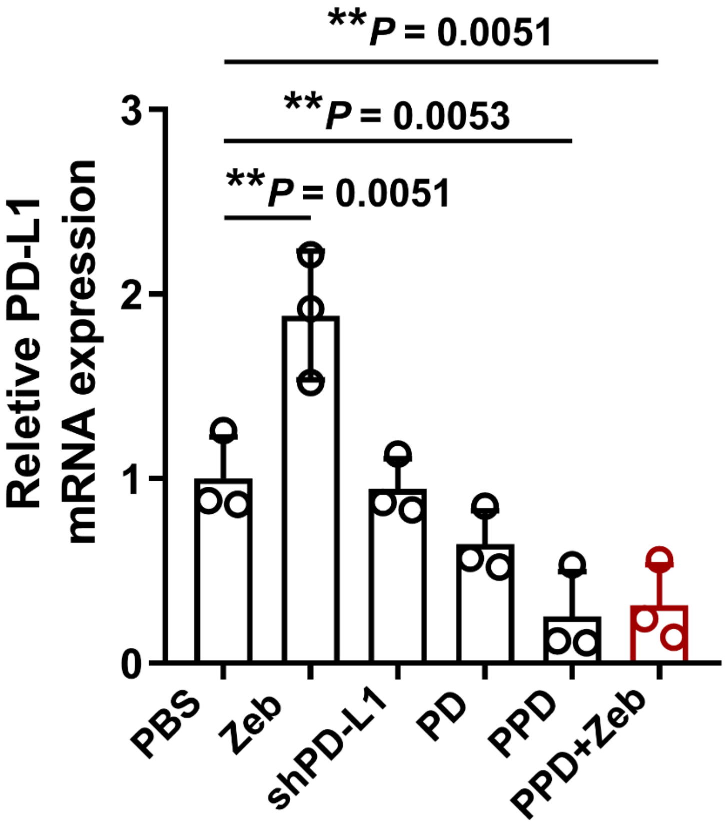

Supplementary Fig. 104 Relative PD-L1 mRNA expression in tumour tissue of 4T1 tumour-bearing mice at the end of the treatment by RT-qPCR assay. Data are presented as mean ± SD, n=3 biologically independent samples. *P* values are calculated by the two-tailed student's t-test as indicated in the figure, $^{**}P<0.01$.

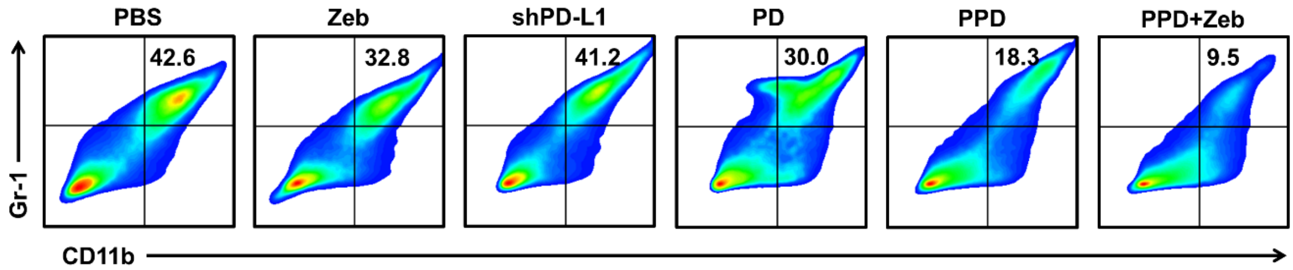

Supplementary Fig. 105 Representative flow cytometric analysis of $CD11b^+$ $Gr\text{-}1^+$ cells gating on $CD45^+$ cells in tumours of 4T1 tumour-bearing mice after treatment.

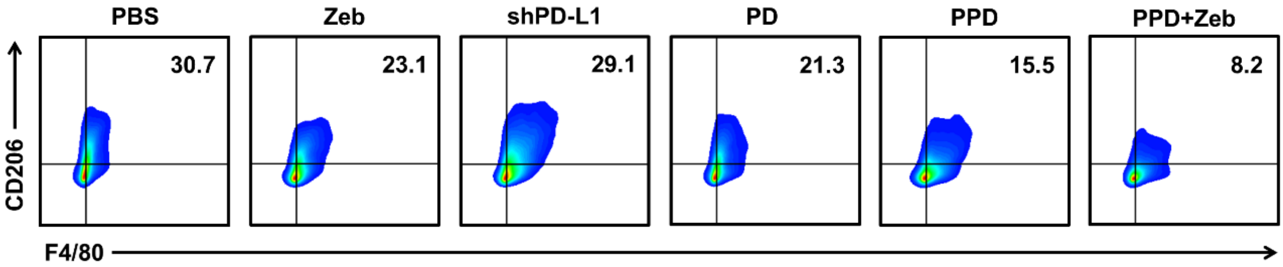

Supplementary Fig. 106 Representative flow cytometric analysis of F4/80$^+$ CD206$^+$ cells gating on CD11b$^+$ cells in tumours of 4T1 tumour-bearing mice after treatment.

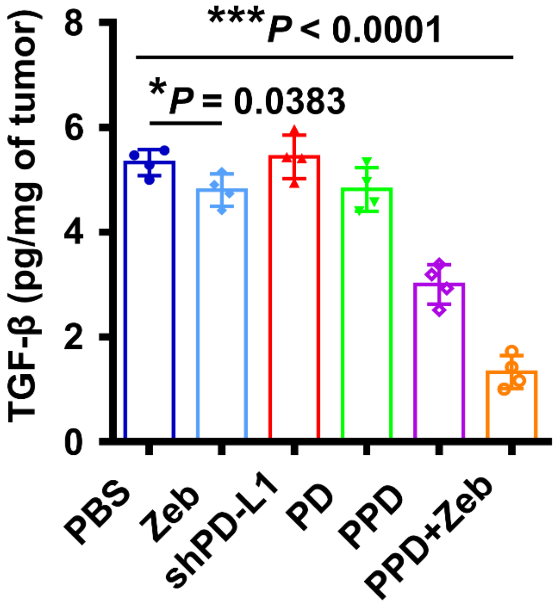

Supplementary Fig. 107 Levels of TGF-β in tumours of 4T1 tumour-bearing mice after treatment. Data are presented as mean ± SD, n=4 biologically independent samples. *P* values are calculated by the two-tailed student's t-test as indicated in the figure, *P<0.05 and ***P<0.001.

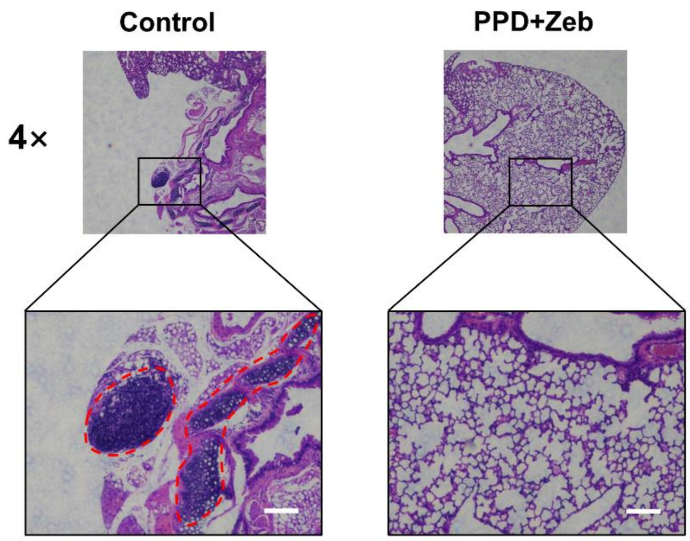

Supplementary Fig. 108 Representive H&E staining images of lung tissue of 4T1 tumour-bearing mice after intravenously administration with 4T1 cells. the red dashed area indicated tumour metastasis, scale bar: 100 μm. A representative image of three biologically independent samples from each group is shown in the figure.

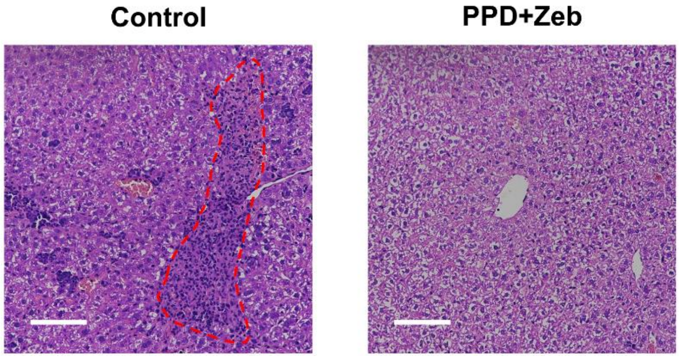

Supplementary Fig. 109 Representive H&E staining images of liver tissue of 4T1 tumour-bearing mice after intravenously administration with 4T1 cells. the red dashed area indicated tumour metastasis, scale bar: 100 μm. A representative image of three biologically independent samples from each group is shown in the figure.

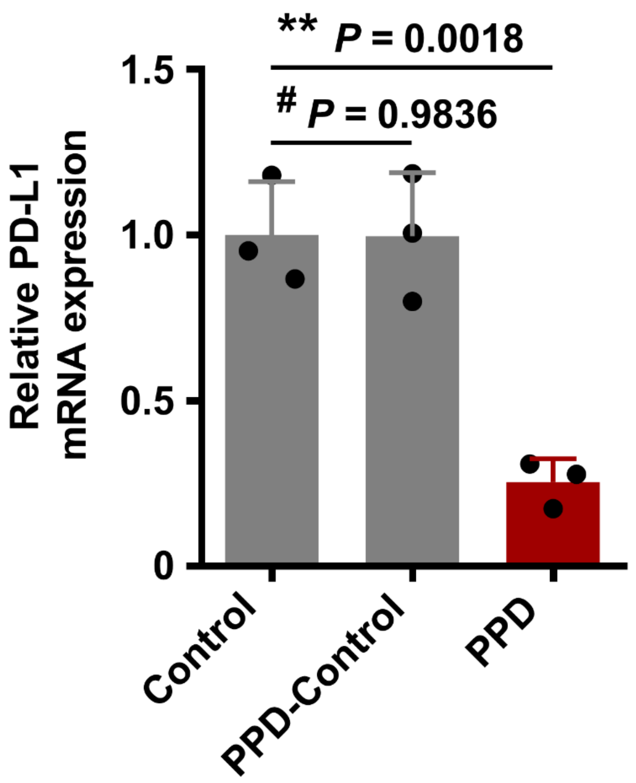

Supplementary Fig. 110 Relative PD-L1 mRNA expression in MDA-MB-231 cells treated with PPD by RT-qPCR assay. Data are presented as mean ± SD, n=3 biologically independent samples. *P* values are calculated by the two-tailed student's t-test as indicated in the figure, #*P*>0.05, **P*<0.05, and ***P*<0.01.

Supplementary Table 4 Primer sequences used for RT-qPCR in this study.

Gene	Forward primer	Reverse primer
Mouse GAPDH	CCCTTAAGAGGGATGCTGCC	TACGGCCAAATCCGTTCACA
Mouse IFN-γ	ACAGCAAGGCGAAAAAGGATG	TGGTGGACCACTCGGATGA
Mouse IL-1β	TGATTCAAGGGGACATTAGGCA	ACCAATTCATCCCCCACCACG
Mouse TNF-α	TTCTGCAAAGGGAGAGTGGTC	TGAAGGTAGGAAGGCCTGAGAT
Mouse IL-6	CTCTGCAAGAGACTTCCATCCA	AAGTAGGGAAGGCCGTGGTT
Mouse TGF-β	GTCCTTGCCCTCTACAACCA	GTTGGACAACTGCTCCACCT

Mouse IL-10	GCTCTTACTGACTGGCATGAG	CGCAGCTCTAGGAGCATGTG
Mouse GzmB	TGTGAAGCCAGGAGATGTGTGCTA	TCAGCTCAACCTCTTGTAGCGTG T
Mouse PD-L1	CCAGCCACTTCTGAGCATGA	CTTCTCTTCCCACTCACGGG
H2-D^b	CACATCCACACTGAACATAAG	TCTCCTGAAGTCTCCTCTG
H2-K^b	TAGGATCCATGGTACCGTGCACGCT GCTCCTGCTGT	CGGTCGACTCAAAGCTTCGCTAG AGAATGAGGGTC
Ctcfl	GGATAAGTGATGGATAAGTGAG	GCCTGGTCTACAGAGTGA
Brdt	GGTTCACATAGCAATCTTCAG	GCAATACCATCTTCTTACTCTC
Lipa	GGTTCCGAATGTGTAGTCT	CCTGTCTGTATGAGCCAATA
Ctag2	TCTTCCGAACATCCATCAAT	ATTCACTCCTCCAGTAACAC
Acrbp	CTCCATCGCTCTCAGACT	AGGTAATCTTCAAGGCACTC
Sycp1	CAGTCATCAGCGAAGATTG	GCTTCAGGTGATTCCAGTA
MAGE-A1	GCGAGATGAGATATACAGGA	CAACAATGGAATCAGTGACT
MAGE-A3	TCATCTCTGAGGAGTTTGTG	AGGAGTTGGAGCATCTGT
Mouse SSX-9	TGACATCGGTGACAATAAGT	ATCGTCTTCTTCCTCTTCAG
Mouse SSX-b2	TGGTAAGGAGCAGGACAA	GGTTCAGTAATCATCGTCTTC
Mouse Taf7l	GTGACGACGATGATGATGA	TGTAATCTTGGTCTGCTTCA
Human β-actin	CAACCGCGAGAAGATGACC	TAGCACAGCCTGGATAGCAA
Human PD-L1	TCGTTGTGCTTGAACCCTT	CATCTGAATCTCGAAACCTCC
Human MAGE-1	CGGCCGAAGGAACCTGACCCAG	GCTGGAACCCTCACTGGGTTGCC
Human SSX-1	CTAAAGCATCAGAGAAGAGAAGC	AGATCTCTTATTAATCTTCTCAG AAA
Human SSX-2	GTGCTCAAATACCAGAGAAGATC	TTTTGGGTCCAGATCTCTCGTG

Human SSX-4	AAATCGTCTATGTGTATATGAAGCT	GGGTCGCTGATCTCTTCATAAAC
Human SSX-5	GTTCTCAAATACCACAGAAGATG	CTCTGCTGGCTTCTCGGGCG
HLA-A	TCAGATAGAAAAGGAGGGAGTTACA	ACAAGCTGTGAGGGACACAT
HLA-B	CCTGAGATGGGAGCCGTCTT	CTCCGATGACCACAACTGCT

Supplementary Table 5 Monoclonal antibodies used for flow cytometry in this study.

Antibody	Antibody dilutions	Source	Catalog number	Clone number
Anti-CD8 -APC	1:200	eBioscience	17-0081-83	53-6.7
Anti-CD3-FITC	1:200	eBioscience	11-0031-85	145-2C11
Anti-CD4-PE/Cy7	1:200	eBioscience	25-0047-42	SK-3
Anti-CD45-PerCP/Cy5.5	1:200	eBioscience	45-0451-82	30-F11
Anti-CD11b-APC	1:200	eBioscience	45-4801-82	BM8
Anti-CD206-PE/Cy7	1:200	eBioscience	25-2069-41	19.2
Anti-Gr-1-PE	1:200	eBioscience	MA1-83934	RB6-8C5
Anti-MHC II-APC/eFluor 780	1:200	eBioscience	47-5321-80	M5/114.15.2
Anti-CD11c-PE	1:200	eBioscience	12-0114-82	N418
Anti-CD80-APC	1:200	eBioscience	17-0801-82	16-10A1
Anti-Foxp3-eFluor660	1:200	eBioscience	50-5773-82	FJK-16s
Anti-CD44-PerCP/Cy5.5	1:200	eBioscience	45-0441-82	IM7
Anti-CD62L-APC/Cy7	1:200	eBioscience	A15409	MEL-14
Anti-IL17-APC	1:200	eBioscience	17-7177-81	eBio17B7

Anti-PD-L1-PE	1:200	eBioscience	12-5983-42	MIH1
Anti-CD11c-Brv421	1:200	Biolegend	117329	N418
Anti-F4/80-Brv421	1:200	Biolegend	123137	BM8

Supplementary Table 6 Relevant parameters of Hu-HSC-NPG mice.

Entry	$mCD45^+$ %MNCs [a]	$hCD45^+$ %MNCs [b]	$hCD3^+$ $\%hCD45^+$
1	46.61%	53.39%	53.03%
2	32.66%	67.34%	39.80%
3	62.07%	37.93%	37.10%
4	56.63%	43.37%	62.23%
5	32.10%	67.90%	51.10%
6	47.59%	52.41%	60.07%

[a] The percentages of mouse $CD45^+$ cells in peripheral blood mononuclear cells (MNCs).

[b] The percentages of human $CD45^+$ cells in peripheral blood mononuclear cells.

[c] The percentages of human $CD3^+$ cells in human $CD45^+$ cells.
