# Supplementary material for: Combination of epigenetic regulation with gene therapy-mediated immune checkpoint blockade induces anti-tumour effects and immune response in vivo
Source: Nat Commun. 2021 Nov 18;12:6742. doi: 10.1038/s41467-021-27078-x (PMC8602287; doi:10.1038/s41467-021-27078-x)
Supplement: Supplementary file 2 — Peer Review File [file 41467_2021_27078_MOESM2_ESM.pdf]

nature portfolio

Peer Review File

Combination of epigenetic regulation with gene therapy-mediated immune checkpoint blockade induces anti-tumour effects and immune response in vivo

Open Access This file is licensed under a Creative Commons Attribution 4.0 International License, which permits use, sharing, adaptation, distribution and reproduction in any medium or format, as long as you give appropriate credit to the original author(s) and the source, provide a link to the Creative Commons license, and indicate if changes were made. In the cases where the authors are anonymous, such as is the case for the reports of anonymous peer reviewers, author attribution should be to 'Anonymous Referee' followed by a clear attribution to the source work. The images or other third party material in this file are included in the article's Creative Commons license, unless indicated otherwise in a credit line to the material. If material is not included in the article's Creative Commons license and your intended use is not permitted by statutory regulation or exceeds the permitted use, you will need to obtain permission directly from the copyright holder. To view a copy of this license, visit http://creativecommons.org/licenses/by/4.0/.

Reviewers' Comments:

Reviewer #1:
Remarks to the Author:
The paper by Fang et al with the title ‘Epigenetic regulation with gene immunotherapy to treate tumor and inhibit its relapse and metastasis reports on the development of a novel formulation for gene delivery. This plasmid carrier was then i.v. injected in mice to show efficacy in shRNA-silencing and in combination with zebularine (DNMT inhibitor) demonstrated anti-tumor immune effects. The authors describe in this extremely lengthy paper 55 supplementary figures, two main figures and 7 pages text how they developed the polycationic gene carrier using in vitro cell transfection systems. The in vivo anti-tumor application is explained in the remainder of the paper (in total 92 suppl figures and 7 main figures). Peritumoral injection of Zeb induces antigen processing and presentation capacity and PD-L1 expression of tumor cells, maturation markers on APC and more T cell infiltration. Addition of PD-L1 silencing plasmids in the novel vehicle mediated strong tumor outgrowth delays, protective immunity to distant tumors in two tumor models. The vehicle was delivered systemically via i.v. injections and reached the tumor site. This latter is possible for mRNA-based gene delivery systems, but not yet for plasmid DNA.
The total amount of data is enormous and the in vivo data appear convincing, but are rather descriptive. Extensive analysis has been performed without really showing mode-of-action.

Main concerns:
1. In the legend at page 15 it is stated that B16F10 tumors were transplanted into BALB/c mice, however B16F10 is derived from C57BL/6 animals. I presumed this is a typo, since tumorimmunology experiments should be performed in syngeneic systems in order to study tumor-specific immune responses and not allo-MHC responses. However, on page 19 they stated that B16F10 tumor bearing mice were re-implanted with 4T1 tumors, which are derived from BALB/c animals, so I seriously doubt if these studies were performed in syngeneic mice.
2. Conclusions are based on description of the findings and based on association, without really showing intervention or proper controls. Importantly, no control shRNA plasmid was included in the in vivo studies. So, we even can not conclude that the induced tumor immunity was actually improved by down modulation of PD-L1 or just by the DNA/vehicle itself.
3. Argumentation is really poor throughout the paper, e.g. DNMT inhibitors upregulate expression of tumor antigens… via cancer testis antigens signalling,…. (p2). At multiple times explanations of the results are given, based on associations.

Reviewer #2:
Remarks to the Author:
In this manuscript, Tian and coworkers have developed a combination immunotherapy strategy to treat tumors. They synthesized and prepared a complex formulation, mPEG-b-PLG/PEI-RT/DNA, to deliver plasmid encoding shPD-L1 to tumor cells, aiming to suppress the expression of PD-L1 on tumor cells and therefore enhance the activity of T cells against tumor cells. Meanwhile, Zebularine (Zeb), a DNA methyltransferase inhibitor, was intratumorally injected for promoting protein expression to epigenetically regulate the tumor immune microenvironment. In tumor models, both enhanced TAA and reduced PD-L1 expression were achieved in the tumor microenvironment, which together enhanced T-cell response against tumor cells. The whole study is well organized, the results are promising, and the associated mechanisms are explored and discussed. The content of the manuscript is attractive, but there are still some major issues need to be addressed before potential acceptance.
1. For the endosomal escape experiment, the authors have not described how to label the endosome/lysosome. It is strange that no endosomes/lysosomes were observed at 7 h after incubation. Even though the DNA complex may escape from the endosome/lysosome, not all of the endosome/lysosome ruptured. So, please check the methods of labeling the endosome or lysosome at 7 hours post-treatment of DNA particles.

2. The distribution of DNA complex and the expressed protein within tumors need to be characterized
3. Line 28, page 2: the authors claimed that gene therapy-mediated blockage of immune checkpoint could reduce the side effects that was associated with antibody therapy. In order to validate this point, the authors need to provide evidence, or at least cite literatures to support this claim.
4. Would the shPL-D1 be expressed in other type of cells other than tumor cells in the tumor microenvironment, for example, the DC cells or fibroblast cells in the tumors?

Reviewer #3:
Remarks to the Author:
The current manuscript focuses on optimizing a gene delivery system, termed as PPD, that can be used to efficiently deplete PD-L1 in cancer cells. The authors have further proposed a DNMT inhibitor-based combinatorial strategy that potentially synergizes with the PPD approach to improve anticancer immune response. Overall, this is a well-structured manuscript; an incredible amount of experimental evidence has reinforced the importance of this work, both in terms of technological development and therapeutic application. Below are a few questions that need to be clarified:

1. Experiments were carried out on four cancer cell lines originating from mouse and human species. What is the rationale for using them in this study?

2. Page 8 (Fig. 2f and 2g) – In comparison with Fig. 2h, which time point was applied to Fig. 2f and 2g as well as Supplementary Fig. 42-47?

3. Page 9 (Fig. 2h and Supplementary Fig. 49/51/53) – "Images were taken at different times (1, 4, and 7 h)". What does each time point mean? Also, the experimental procedure for studying endosomal escape shows no information about Lysotracker Green staining.

4. Page 11 (Supplementary Fig. 57) – There is no information showing the half-life of circulatory DNA under individual conditions.

5. Page 11 (Supplementary Fig. 58 and 59) – There is no information in the main text, describing the tumor model(s) used in the biodistribution study of carries/DNA complexes.

6. Page 12 (Fig. 3) – No strong evidence shows that zebularine can "increase MHC I expression". RT-qPCR should be performed to analyze HLA-A/B/C and PD-L1 mRNA expression in human cancer cells (as seen in Supplementary Fig. 87).

7. Page 12 (Fig. 3 and illustrated in Fig. 1) – No direct evidence suggests that MHC I plays a key role in zebularine cooperation with PPD treatment. Does depletion of certain MHC I molecule(s) protect tumor growth against the zebularine/PPD treatment?

8. Given that several DNMTi are under clinical studies, what is the rationale for using zebularine in the current research? Does any other DNMTi exhibit the similar effects in combination with PPD?

9. Considering the zebularine/PPD combinatorial treatment as "a novel therapeutic scheme for cancer immunotherapy", the authors should offer some insights into the mechanism of drug action – for example, performing RNA-seq to uncover major target genes, pathways and/or biological processes in response to zebularine and PPD alone or in combination. This information may strengthen the central concept of the current research (Fig. 1) and support the future investigations aimed at inhibiting DNMT activity in immunotherapy.

10. Page 19 (Fig. 5n-q) – Evidence for "personalized immunotherapy" is relatively weak. The similar experiments would ideally need to be repeated on a different pair of cell lines, such as 4T1 vs MCF7.

11. The therapeutic effect observed in this research relies on tissue culture and cell line-based xenograft studies, which could significantly differ from the original tumors. Hence, the clinical relevance of the PPD-/+zebularine (DNMTi) approach is uncertain. To avoid overstatements, the authors should consider either repeating some key experiments (for example, Fig. 3b-d) on patient-derived xenograft and/or transgenic mouse cancer models, or expanding the Discussion section to fully describe the major limitations of the current research and provide possible strategies/directions to overcome them in the future studies.

12. The readability of the manuscript does not reach publication standards. Thorough proofreading and editing are required to improve the quality of writing prior to publication.

Response to Reviewers

REVIEWER COMMENTS

Reviewer #1, expert in immune cell characterization/mouse models and therapy (Remarks to the Author):

The paper by Fang et al with the title 'Epigenetic regulation with gene immunotherapy to treat tumor and inhibit its relapse and metastasis reports on the development of a novel formulation for gene delivery. This plasmid carrier was then i.v. injected in mice to show efficacy in shRNA-silencing and in combination with zebularine (DNMT inhibitor) demonstrated anti-tumor immune effects. The authors describe in this extremely lengthy paper 55 supplementary figures, two main figures and 7 pages text how they developed the polycationic gene carrier using in vitro cell transfection systems. The in vivo anti-tumor application is explained in the remainder of the paper (in total 92 suppl figures and 7 main figures). Peritumoral injection of Zeb induces antigen processing and presentation capacity and PD-L1 expression of tumor cells, maturation markers on APC and more T cell infiltration. Addition of PD-L1 silencing plasmids in the novel vehicle mediated strong tumor outgrowth delays, protective immunity to distant tumors in two tumor models. The vehicle was delivered systemically via i.v. injections and reached the tumor site. This latter is possible for mRNA-based gene delivery systems, but not yet for plasmid DNA. The total amount of data is enormous and the in vivo data appear convincing, but are rather descriptive. Extensive analysis has been performed without really showing mode-of-action.

Main concerns:

1. In the legend at page 15 it is stated that B16F10 tumors were transplanted into BALB/c mice, however B16F10 is derived from C57BL/6 animals. I presumed this is a typo, since tumorimmunology experiments should be performed in syngeneic systems in order to study tumor-specific immune responses and not allo-MHC responses. However, on page 19 they stated that B16F10 tumor-bearing mice were re-implanted with 4T1 tumors, which are derived from BALB/c animals, so I seriously doubt if these studies were performed in syngeneic mice.

Response:

Thanks very much for your kind suggestion. As you suggested, B16F10 tumor was transplanted into C57 BL/6 mice at page 15, it was a typo in the original manuscript. We have checked the manuscript and corrected it carefully in the revised manuscript.

Moreover, to correct the tumor re-challenge experiments in syngeneic mice, B16F10 tumor-bearing mice (C57BL/6) after PPD plus Zeb treatment were re-implanted with B16F10, MC38 (murine colon carcinoma) and LLC (Lewis lung cancer) tumors, respectively. The supplementary data were shown in **Fig. R1**.

The detailed descriptions were supplemented as follows: as shown in **Fig. R1b-d**, PPD plus Zeb group could efficiently inhibit B16F10 tumor relapse compared with control group (i.e., age- and body-weight-matched healthy mice). According to the immune cell analysis in the spleen of B16F10 tumor-bearing mice after treatment, the percentage of TEM cells and TEMs/TCMs ratio in the spleen of B16F10 tumor-bearing mice in PPD plus Zeb group were much higher than that in the control group (**Fig. R1e-j)**. These results demonstrated be that PPD plus Zeb combination therapy could evoke tumor-specific immune memory effect, which considerably inhibited B16F10 tumor relapse. Nevertheless, when B16F10 bearing mice after PPD plus Zeb treatment were re-implanted with MC38 or LLC tumor, the growth curves and tumor weight of distal tumors showed negligible differences between PPD plus Zeb group and control group (**Fig. R1k-p**). On the basis of the above results, PPD plus Zeb initiated tumor specific immune memory response and suggested its potential for personalized immunotherapy.

The relevant descriptions were presented in the Revised Manuscript (Page 20, Paragraph 2, Line 18-28, red words; Page 21, Paragraph 1, Line 9-16, red words).

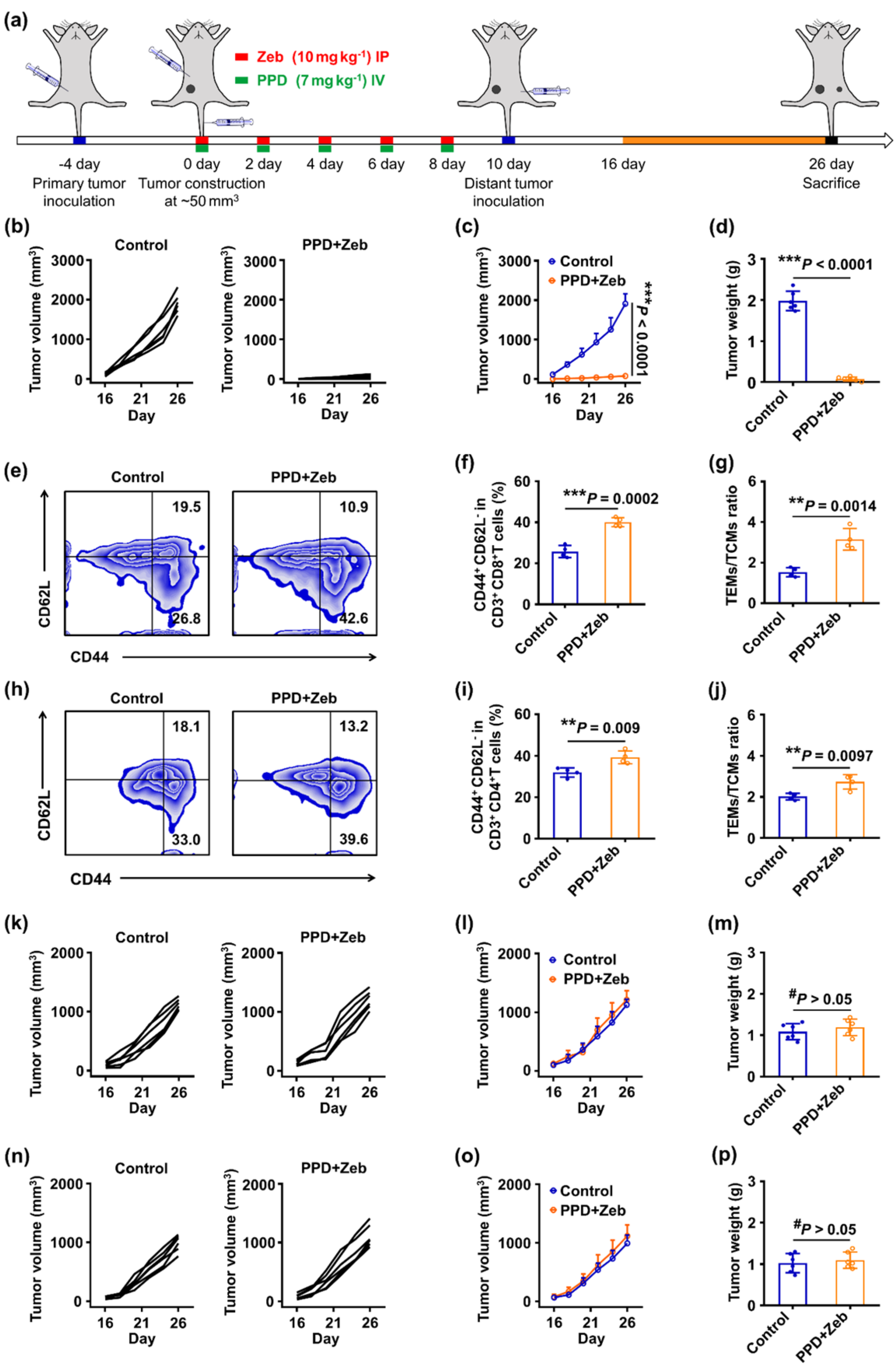

Fig. R1 (i.e., Fig. 5 in the Revised Manuscript) PPD combined with Zeb inhibited tumor

relapse and initiated tumor specific immune memory effect. (a) Therapeutic schedule for PPD plus Zeb mediated inhibition of tumor relapse. **(b)** Individual and **(c)** average tumor growth kinetics in distant B16F10 tumor-bearing mice receiving PPD plus Zeb treatment. Data are presented as mean ± SD (n=6), ***P<0.001. Age- and body-weight-matched healthy mice were used as control. **(d)** Average tumor weight of distant B16F10 tumor-bearing mice receiving PPD plus Zeb treatment. Data are presented as mean ± SD (n=6), ***P<0.001. **(e)** Representative flow cytometric analysis of TEM ($CD44^+CD62L^-$) and TCM ($CD44^+ CD62L^+$) cells gating on $CD8^+$ T cells in spleen of B16F10 tumor-bearing mice at the end of treatment. Relative quantification of **(f)** TEM ($CD44^+CD62L^-$) and **(g)** ratio of TEMs/TCMs gating on $CD8^+$T cells in the spleen of B16F10 tumor-bearing mice at the end of treatment. Data are presented as mean ± SD (n=4), **P<0.01. **(h)** Representative flow cytometric analysis of TEM ($CD44^+CD62L^-$) and TCM ($CD44^+CD62L^+$) cells gating on $CD4^+$T cells in spleen of B16F10 tumor-bearing mice at the end of treatment. Relative quantification of **(i)** TEM and **(j)** ratio of TEMs/TCMs gating on $CD4^+$T cells in the spleen of B16F10 tumor-bearing mice at the end of treatment. Data are presented as mean ± SD (n=4), **P<0.01. **(k)** Individual and **(l)** average tumor growth kinetics in distant MC38 tumor-bearing mice receiving PPD plus Zeb treatment (n=6). Age- and body-weight-matched healthy mice were used as control. **(m)** Average tumor weight of distant MC38 tumor-bearing mice receiving PPD plus Zeb treatment. Data are presented as mean ± SD (n=6), $^{\#}P$>0.05. **(n)** Individual and **(o)** average tumor growth kinetics in distant LLC tumor-bearing mice receiving PPD plus Zeb treatment (n=6). Age- and body-weight-matched healthy mice were used as control. **(p)** Average tumor weight of distant LLC tumor-bearing mice receiving PPD plus Zeb treatment. Data are presented as mean ± SD (n=6), $^{\#}P$>0.05.

2. Conclusions are based on description of the findings and based on association, without really showing intervention or proper controls. Importantly, no control shRNA plasmid was included in the in vivo studies. So, we even can not conclude that the induced tumor immunity was actually improved by down modulation of PD-L1 or just by the DNA/vehicle itself.

Response：

Thanks very much for your suggestion. To sufficiently verify the down-regulation of PD-L1 can effectively inhibit tumor growth, B16F10 tumor-bearing mice (or 4T1 tumor-bearing mice) were divided into three groups: PBS, mPEG-b-PLG/PEI-RT3/control shRNA plasmid (abbreviated as PPD-Control), and PPD. The supplementary data were shown in **Fig. R2** and **Fig. R3**.

The detailed descriptions were supplemented as follows: as shown in **Fig. R2b-c**, similar to PBS group, PPD-Control group could hardly inhibit the tumor growth of B16F10 tumor-bearing mice. However, compared with PPD-Control group, PPD could effectively inhibit the tumor growth. And the tumor weight data showed the consistent results (**Fig. R2d).** Based on the immune cell analysis, RT-qPCR assay, and ELISA assay results, PPD group could effectively down regulate PD-L1 level in tumor tissue, which was conducive to tumor eradication (**Fig. R2f-h**). The similar results were obtained in 4T1 tumor-bearing mice (**Fig. R3**).

The relevant description was supplemented in the Revised Manuscript (Page 13, Paragraph 2, Line 24-26, red words; Page 23, Paragraph 2, Line 30-31, red words).

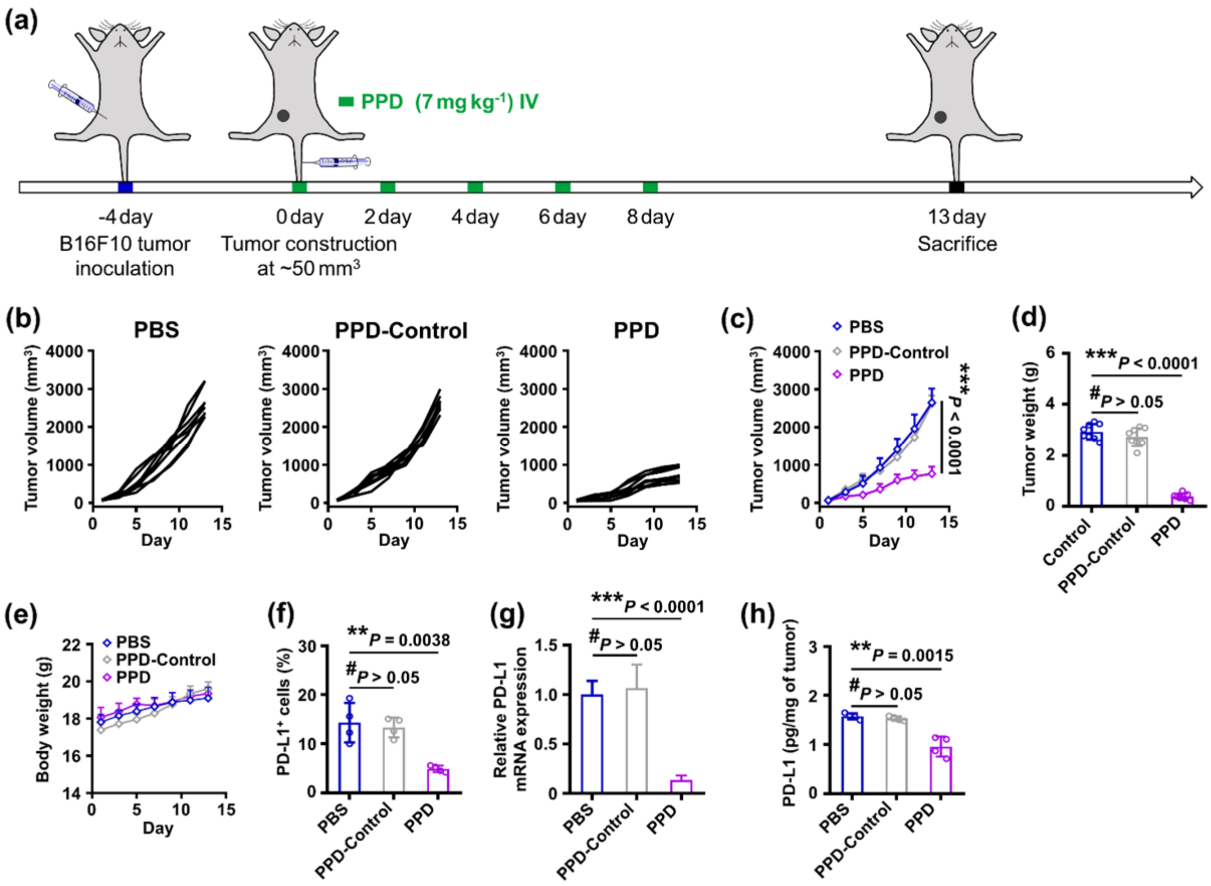

Fig. R2 (i.e., Supplementary Fig. 70 in the Revised Supplementary Information) (a) Therapeutic schedule for PPD mediated inhibition of B16F10 tumor growth. **(b)** Individual and **(c)** average tumor growth kinetics in B16F10 tumor-bearing mice receiving different treatments. Data are presented as mean ± SD (n=8), ****P*<0.001. **(d)** Average tumor weight of B16F10 tumor-bearing mice at the end of treatment, PBS and PPD-Control were served as control groups. Data are presented as mean ± SD (n=8), #P>0.05, ***P<0.001. **(e)** Average body weight changes of

B16F10 bearing mice receiving different treatments. **(f)** Percentages of PD-L1$^+$ cells in tumor tissues of B16F10 tumor-bearing mice at the end of treatment. Data are presented as mean ± SD (n=4), $^{\#}$P>0.05, ***P*<0.01. **(g)** Relative PD-L1 mRNA expression in tumor tissue of B16F10 tumor-bearing mice at the end of the treatment by RT-qPCR assay. **(h)** Levels of PD-L1 protein expression in tumor tissues of B16F10 tumor-bearing mice at the end of the treatment. Data are presented as mean ± SD (n=4), $^{\#}$*P*>0.05, ***P*<0.01.

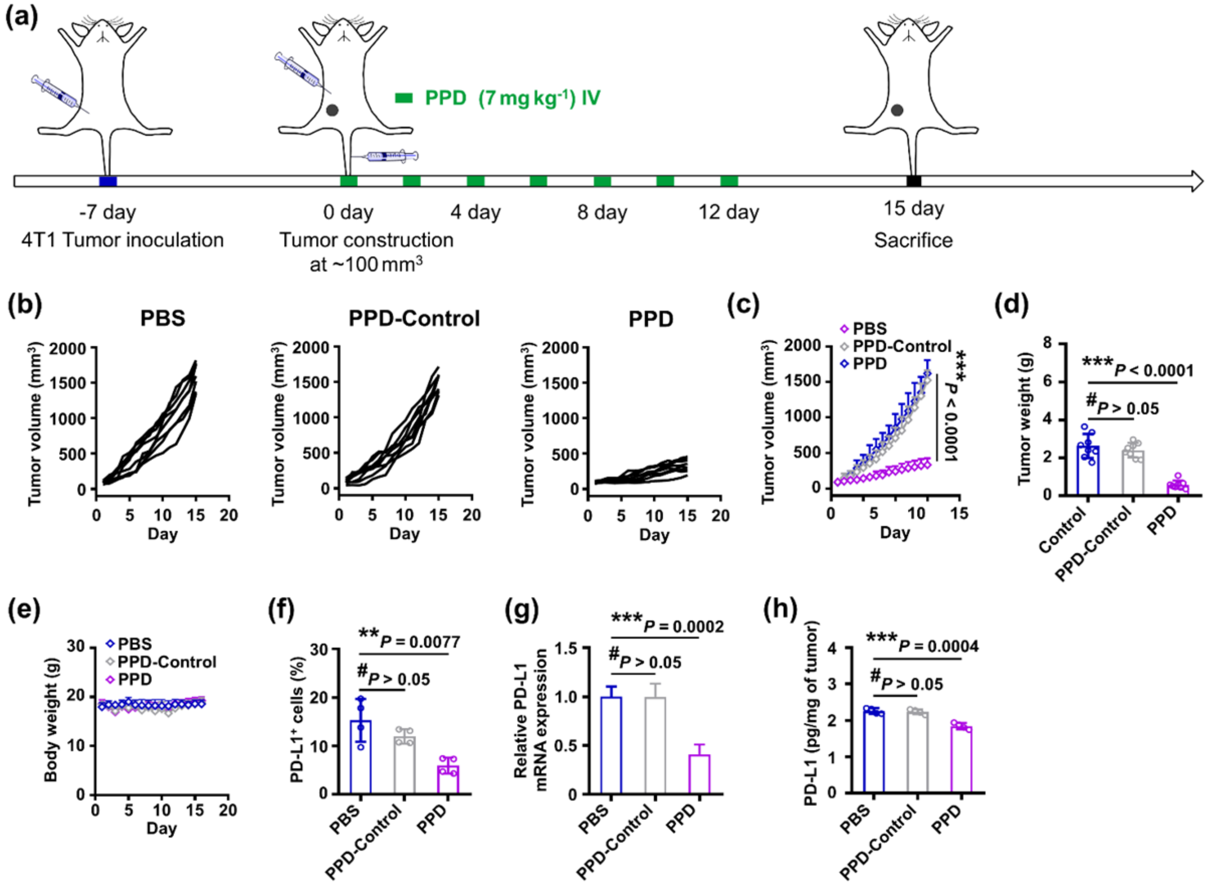

Fig. R3 (i.e., Supplementary Fig. 92 in the Revised Supplementary Information) (a) Therapeutic schedule for PPD mediated inhibition of 4T1 tumor growth. **(b)** Individual and **(c)** average tumor growth kinetics in 4T1 tumor-bearing mice receiving different treatments. Data are presented as mean ± SD (n=8), ****P*<0.001. **(d)** Average tumor weight of 4T1 tumor-bearing mice at the end of treatment, PBS and PPD-Control were served as control groups. Data are presented as mean ± SD (n=8), $^{\#}$P>0.05, ****P*<0.001. **(e)** Average body weight changes of 4T1 bearing mice receiving different treatments. **(f)** Percentages of PD-L1$^+$ cells in tumor tissues of 4T1 tumor-bearing mice at the end of treatment. Data are presented as mean ± SD (n=4), $^{\#}$*P*>0.05, ***P*<0.01. **(g)** Relative PD-L1 mRNA expression in tumor tissue of 4T1 tumor-bearing mice at the

end of the treatment by RT-qPCR assay. **(h)** Levels of PD-L1 protein expression in tumor tissues of 4T1 tumor-bearing mice at the end of the treatment. Data are presented as mean ± SD (n=4), $^{\#}P$>0.05, **P<0.01.

3. Argumentation is really poor throughout the paper, e.g. DNMT inhibitors upregulate expression of tumor antigens… via cancer testis antigens signalling,…. (p2). At multiple times explanations of the results are given, based on associations.

Response:

Thanks very much for your suggestion. The supplementary data were shown in **Fig. R4-R7**.

The detailed descriptions were supplemented as follows: recently, it was reported that DNMT inhibitors could up-regulate the expression of tumor antigens and improve the level of MHC I molecule through cancer testis antigen (CTA) signaling (Nat. Rev. Cancer 2019, 19, 151-161; Cell 2017, 171, 1284-1300; Nat. Genet. 2017, 49, 1052-1060). To verify this argumentation, tumor antigens (MAGE-E1, CD146, and TRP1), MHC I molecules (H2-K^b and H2-D^b) and CTA genes were determined at the end of B16F10 tumor treatment by ELISA or RT-PCR assay. As shown in **Fig. R4a-e**, Zeb group had much higher levels of tumor antigens (MAGE-E1, CD146, and TRP1) and MHC I molecules (H2-K^b and H2-D^b) than PBS group in B16F10 tumor-bearing mice. Moreover, according to RT-qPCR assay, Zeb could also significantly upregulate the levels of CTA genes including SSX-9, MAGE-A3, SSX-b2, Taf7l, Ctcfl, and Lipa compared with PBS group (**Fig. R5**). Furthermore, in human cancer cell lines (HepG2 and A549 cells), Zeb could also increase MHC I molecules and CTA genes expression (**Fig. R6** and **Fig. R7**). These results indicated that Zeb, as a kind of DNMT inhibitor could activate cancer testis antigen (CTA) signaling, which upregulated tumor antigen expression and improved the levels of MHC I molecules.

The relevant descriptions were supplemented in the Revised Manuscript (Page 13, Paragraph 2, Line 9-12, red words).

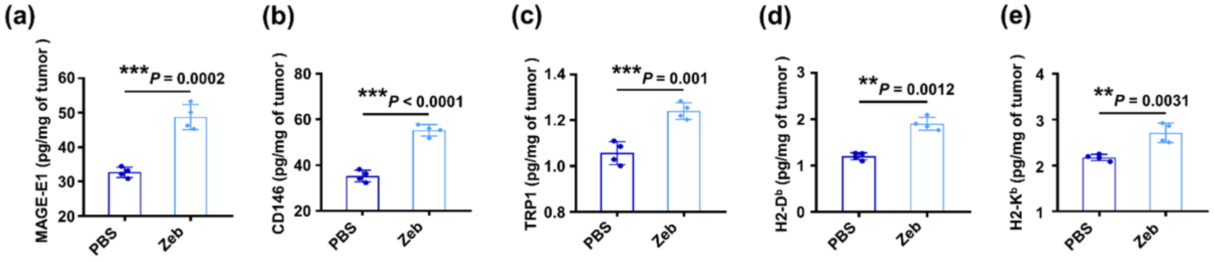

Fig. R4 (i.e., Fig.3h-l in the Revised Manuscript) Levels of tumor associated antigen including **(a)** MAGE-E1, **(b)** CD146, and **(c)** TRP1 in tumor tissues of B16F10 tumor-bearing mice receiving Zeb treatment. Data are presented as mean ± SD (n=4), ****P*<0.001. Levels of MHC I molecules including **(d)** H2-D^b and **(e)** H2-K^b in tumor tissues of B16F10-tumor-bearing BABL/c mice receiving different treatments. Data are presented as mean ± SD (n=4), ***P*<0.01, ****P*<0.001.

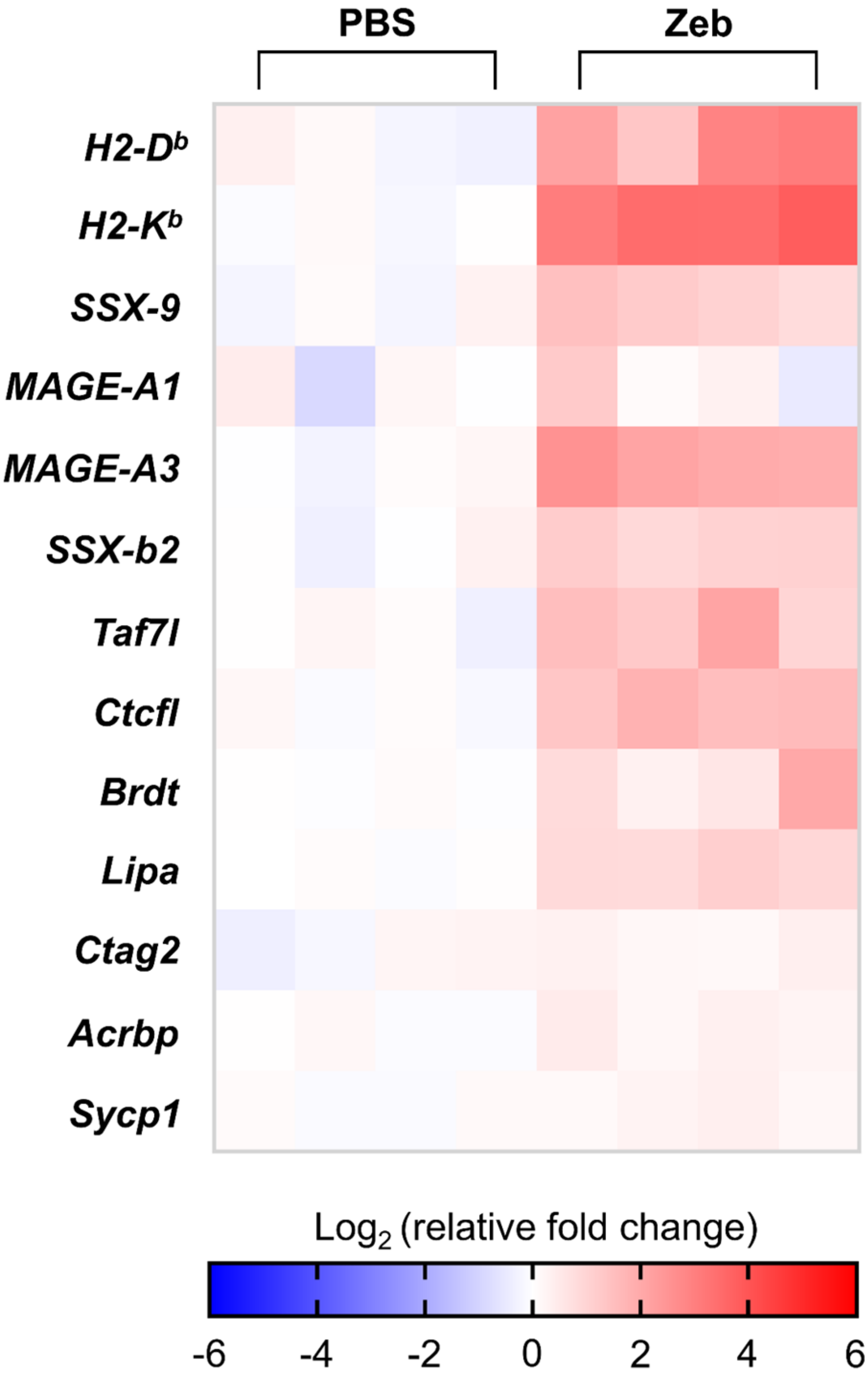

Fig. R5 (i.e., Supplementary Fig. 85 in the Revised Supplementary Information) Heatmap of relative mRNA expression of MHC I (H2-D^b and H2-K^b) and CTAs (SSX-9, SSX-b2, MAGE-A1, MAGE-A3, Taf7l, Ctcfl, Brdt, Lipa, Ctag2, Acrbp, and Sycp1) in tumor tissues of B16F10 tumor-bearing mice at the end of Zeb treatment.

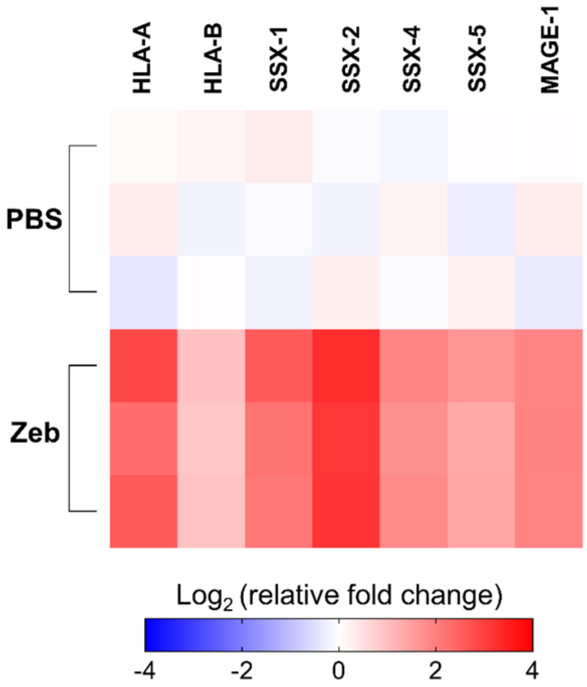

Fig. R6 (i.e., Supplementary Fig. 64 in the Revised Supplementary Information) Heatmap of relative mRNA expression of MHC I (HLA-A and HLA-B）and CTA genes (SSX-1, SSX-2, SSX-4, SSX-5, MAGE-1) in A549 cells treated with 0.5 mg/mL Zeb.

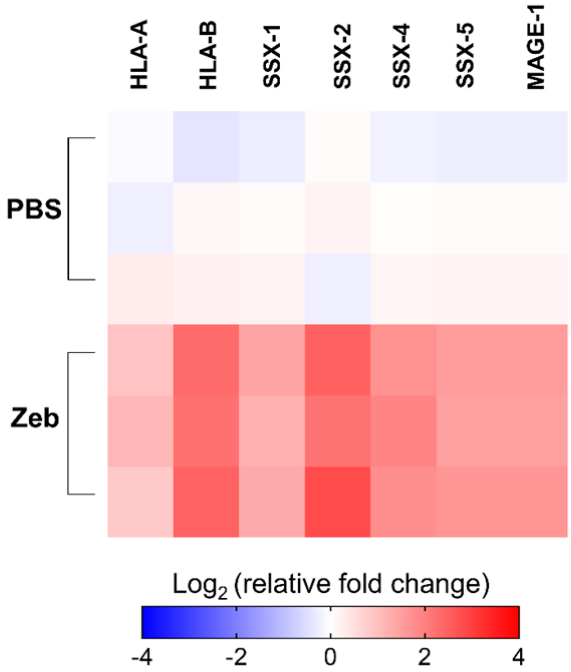

Fig. R7 (i.e., Supplementary Fig. 65 in the Revised Supplementary Information) Heatmap of relative mRNA expression of MHC I (HLA-A and HLA-B）and CTA genes (SSX-1, SSX-2, SSX-4, SSX-5, MAGE-1) in HepG2 cells treated with 0.5 mg/mL Zeb.

Reviewer #2, expert in nanoparticles/immunotherapy (Remarks to the Author):

In this manuscript, Tian and coworkers have developed a combination immunotherapy strategy to treat tumors. They synthesized and prepared a complex formulation, mPEG-b-PLG/PEI-RT/DNA, to deliver plasmid encoding shPD-L1 to tumor cells, aiming to suppress the expression of PD-L1 on tumor cells and therefore enhance the activity of T cells against tumor cells. Meanwhile, Zebularine (Zeb), a DNA methyltransferase inhibitor, was intratumorally injected for promoting protein expression to epigenetically regulate the tumor immune microenvironment. In tumor models, both enhanced TAA and reduced PD-L1 expression were achieved in the tumor microenvironment, which together enhanced T-cell response against tumor cells. The whole study is well organized, the results are promising, and the associated mechanisms are explored and discussed. The content of the manuscript is attractive, but there are still some major issues need to be addressed before potential acceptance.

1. For the endosomal escape experiment, the authors have not described how to label the endosome/lysosome. It is strange that no endosomes/lysosomes were observed at 7 h after incubation. Even though the DNA complex may escape from the endo/lysosome, not all of the

endosome/lysosome ruptured. So, please check the methods of labeling the endosome or lysosome at 7 hours post-treatment of DNA particles.

Response:

Thanks very much for your kind suggestion. The procedure of labeling endo/lysosome was supplemented as follows: B16F10, 4T1, HeLa, or MCF-7 cells were seeded in 6-well plates containing coverslips at a density of 1×10^5 cells per well and incubated for 24 h. Then the culture medium was replaced with fresh culture medium and carrier/Cy5-DNA complexes (1 mg Cy5-DNA per well) were added and incubated for 1, 4, and 7 h. After that, the cells were washed with PBS and fixed with 4% paraformaldehyde for 10 min. Nucleus were stained with 1 mg mL^{-1} of DAPI (1.5 μL per well) for 10 min, and endo/lysosome were stained with 75 nM of Lysotracker Green for 1 h. Finally, the coverslips were taken out and placed on the glass slides, enclosed with glycerol. The samples were observed by confocal laser scanning microscopy (CLSM) (ZEISS LSM780, Germany) and the correlation coefficient R values were obtained from CLSM images (ZEN software). The supplementary data were shown in **Fig. R8-R15.**

Based on your kind suggestion, the endo/lysosomal escape experiment was repeated. The detailed descriptions were supplemented as follows: Nucleus was stained with 4', 6-diamidino-2-phenylindole (DAPI). Endo/lysosome and DNA were labelled with Lysotracker Green (green) and Cy5 (red), respectively. Yellow fluorescent dots indicate the overlap of endo/lysosome (green) and carrier/DNA complexes (red) and reflect the colocation of endo/lysosome and complexes. Images were taken at different time points (1, 4, and 7 h). The yellow fluorescent dots were obviously observed at 1 h for PD and PPD group in B16F10 cells, implying that PD or PPD entered the endo/lysosome (**Fig. R8**). Additionally, more yellow fluorescent dots were observed at 4 h, indicating that more PD or PPD had entered the endo/lysosome. Remarkably, a sharp decline of the yellow fluorescent dots for PD and PPD group was observed from 4 to 7 h. The reason was that the fluorescence intensity of Lysotracker Green (green) channel for PD and PPD was significantly decreased at 7 h, which resulted from the rupture of endo/lysosome. Thus, the significant reduction of yellow fluorescent dots from 4 to 7 h confirmed that PD or PPD could escape from endo/lysosome. By comparison, only a few yellow fluorescent dots were observed for ED group throughout the process. Therefore, the endo/lysosomal escape ability of ED, PD, and PPD could not be directly compared through the confocal fluorescent images. To evaluate the endo/lysosomal escape ability, correlation coefficient R value from confocal

fluorescent images was defined as colocalization degree of carrier/DNA and endo/lysosome. In other words, the greater R value was, the more prominent colocalization degree was. Therefore, the endo/lysosomal escape ability of ED, PD, and PPD could be compared through the change extent of R value. The R values of ED, PD and PPD group increased first and then decreased in B16F10 cells (**Fig. R9**). The reduced R value from 4 to 7 h indicated ED, PD and PPD could escape from endo/lysosomal. In particular, more significant R value reduction suggested that PD and PPD possessed more excellent endo/lysosomal escape ability than ED. Moreover, similar results were obtained in 4T1, HeLa, and MCF-7 cells (**Fig. R10-R15**).

The relevant descriptions were supplemented in the Revised Manuscript (Page 9, Paragraph 2, Line 27-31, red words; Page 10, Paragraph 1, Line 1-21, red words; Page 38, Paragraph 2, Line 4-13, red words).

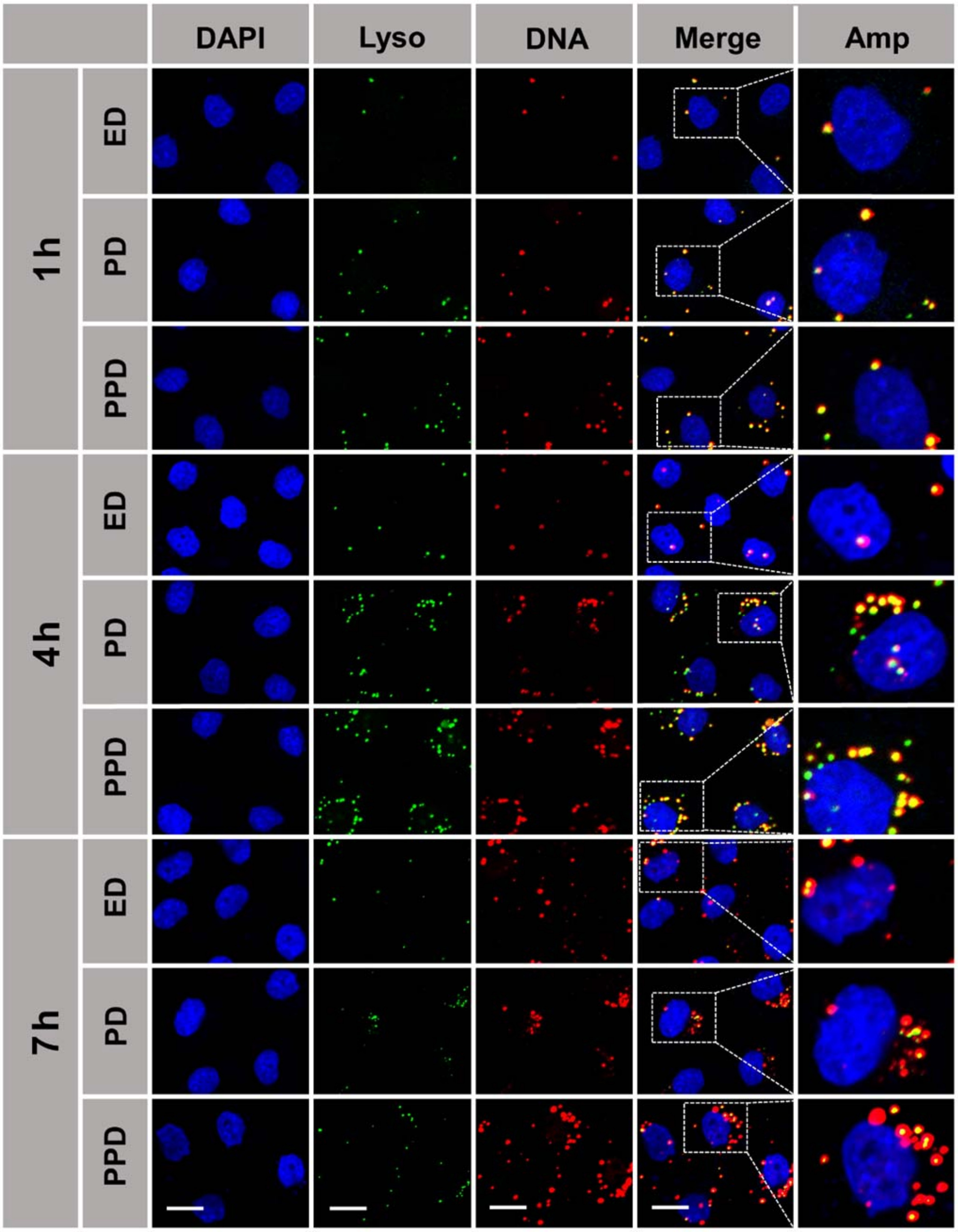

Fig. R8 (i.e., Fig. 2h in the Revised Manuscript) Representative endo/lysosomal escape images of PPD, PD, and ED at various time pointes (1, 4, and 7 h) in B16F10 cells, scale bar: 20 μm.

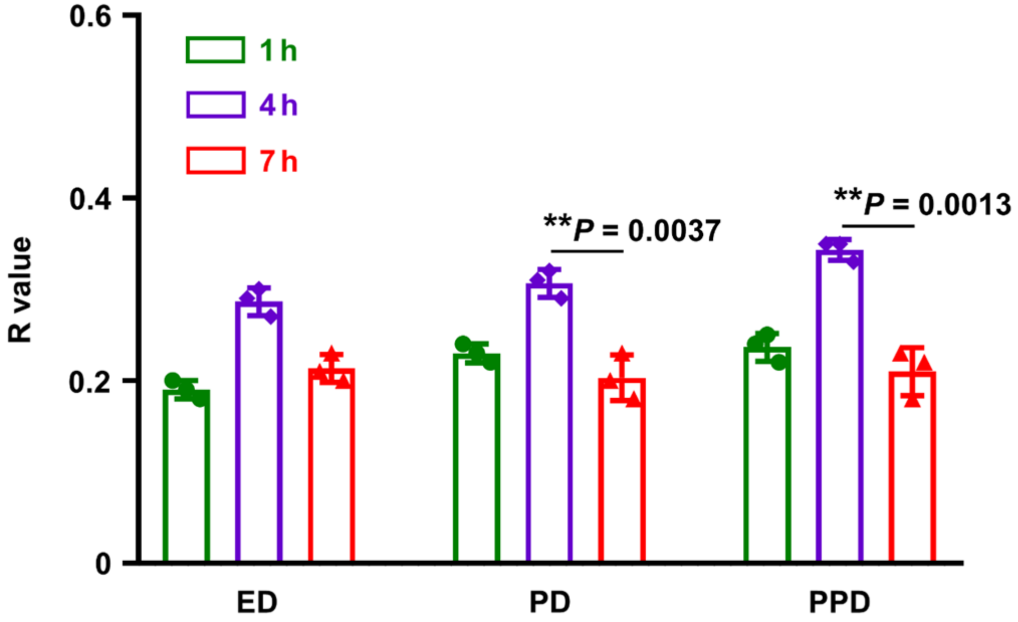

Fig. R9 (i.e., Supplementary Fig. 48 in the Revised Supplementary Information) Correlation coefficient R value obtained from the CLSM images for endo/lysosomal escape ability evaluation in B16F10 cells. Data are presented as mean ± SD (n=3), ***P*<0.01.

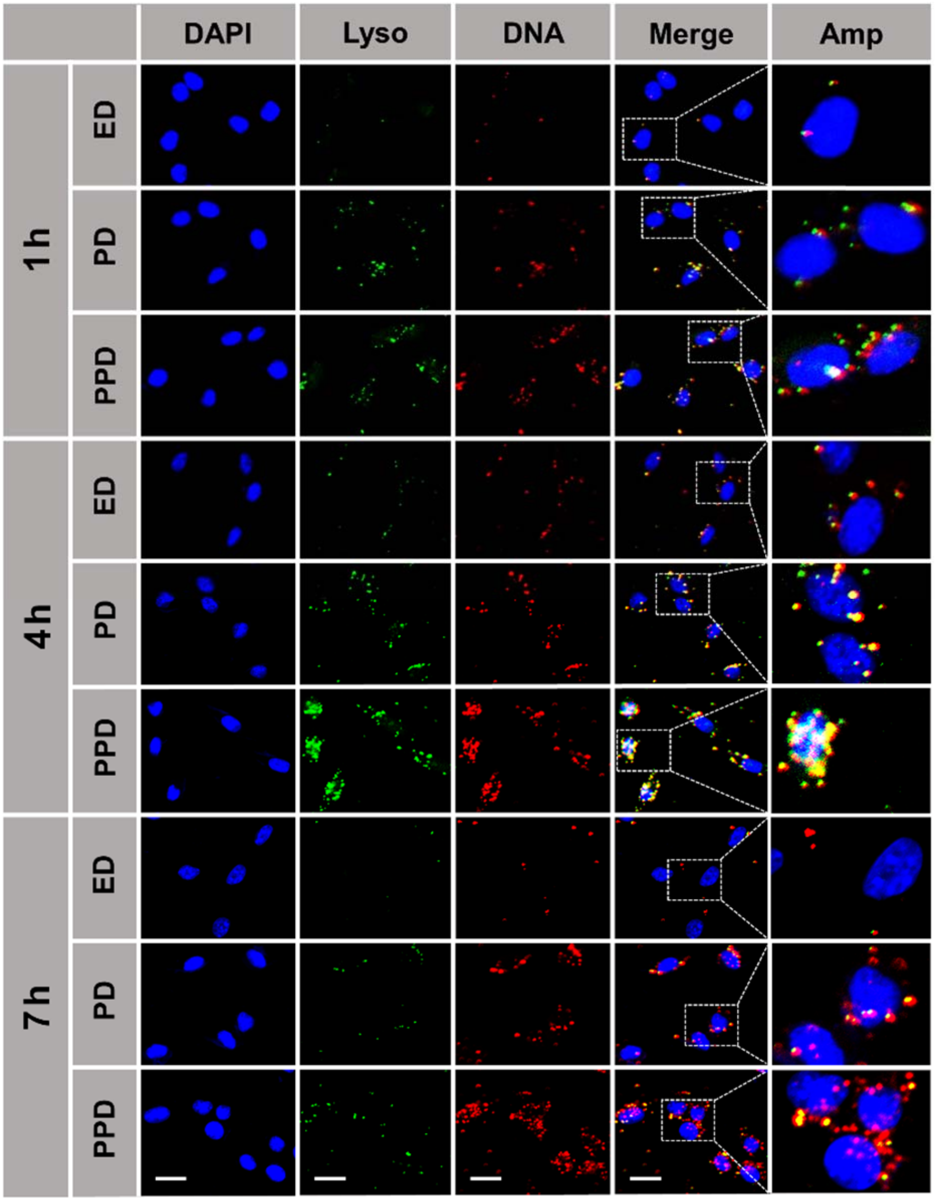

Fig. R10 (i.e., Supplementary Fig. 49 in the Revised Supplementary Information) Representative endo/lysosomal escape images of PPD, PD, and ED at various time pointes (1, 4, and 7 h) in 4T1 cells, scale bar: 20 μm.

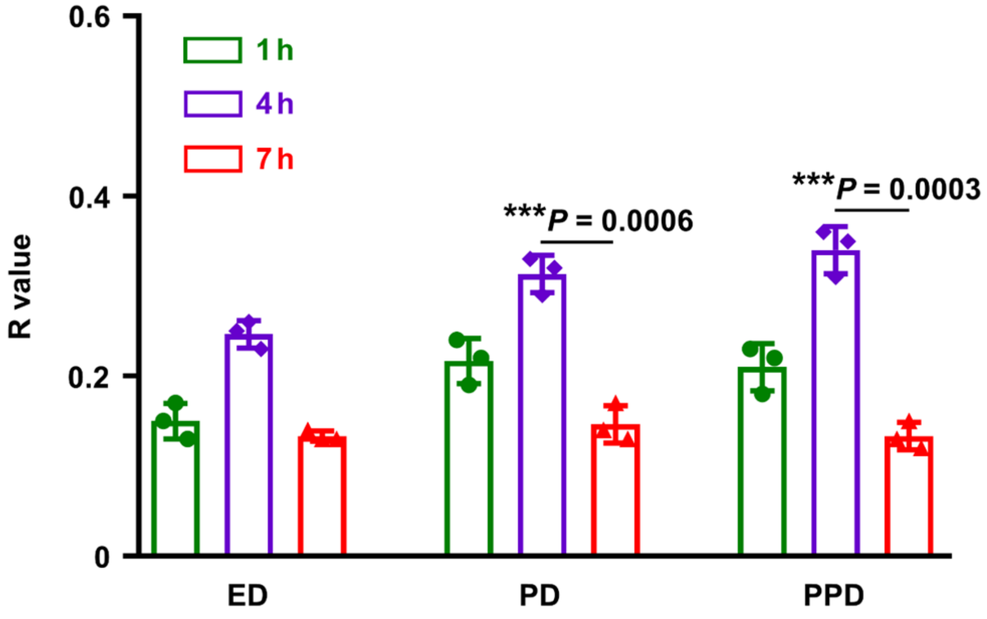

Fig. R11 (i.e., Supplementary Fig. 50 in the Revised Supplementary Information) Correlation coefficient R value obtained from the CLSM images for endo/lysosomal escape ability evaluation in 4T1 cells. Data are presented as mean ± SD (n=3), ****P*<0.001.

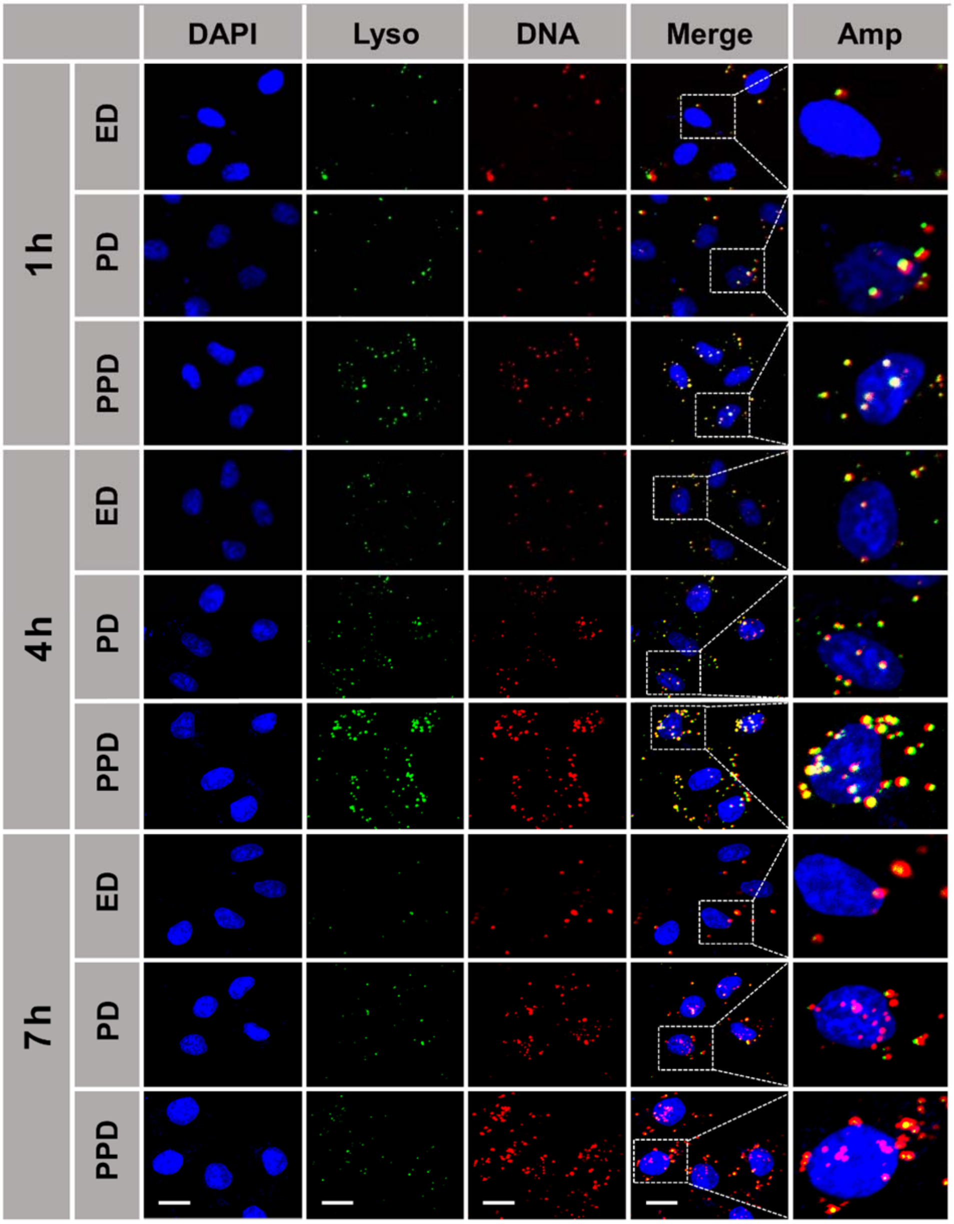

Fig. R12 (i.e., Supplementary Fig. 51 in the Revised Supplementary Information) Representative endo/lysosomal escape images of PPD, PD, and ED at various time pointes (1, 4, and 7 h) in HeLa cells, scale bar: 20 μm.

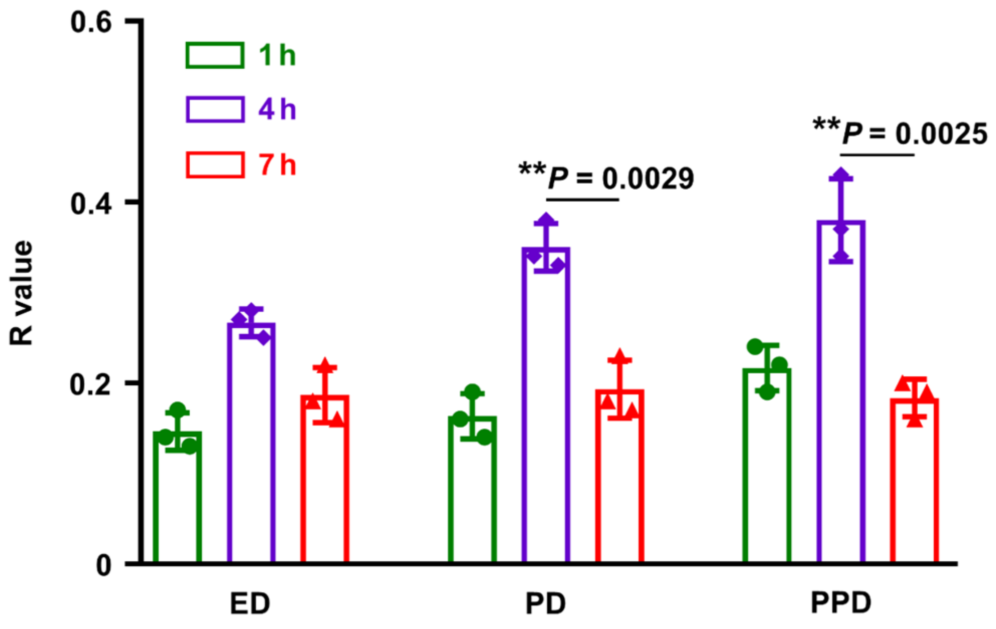

Fig. R13 (i.e., Supplementary Fig. 52 in the Revised Supplementary Information) Correlation coefficient R value obtained from the CLSM images for endo/lysosomal escape ability evaluation in HeLa cells. Data are presented as mean ± SD (n=3), ***P*<0.01.

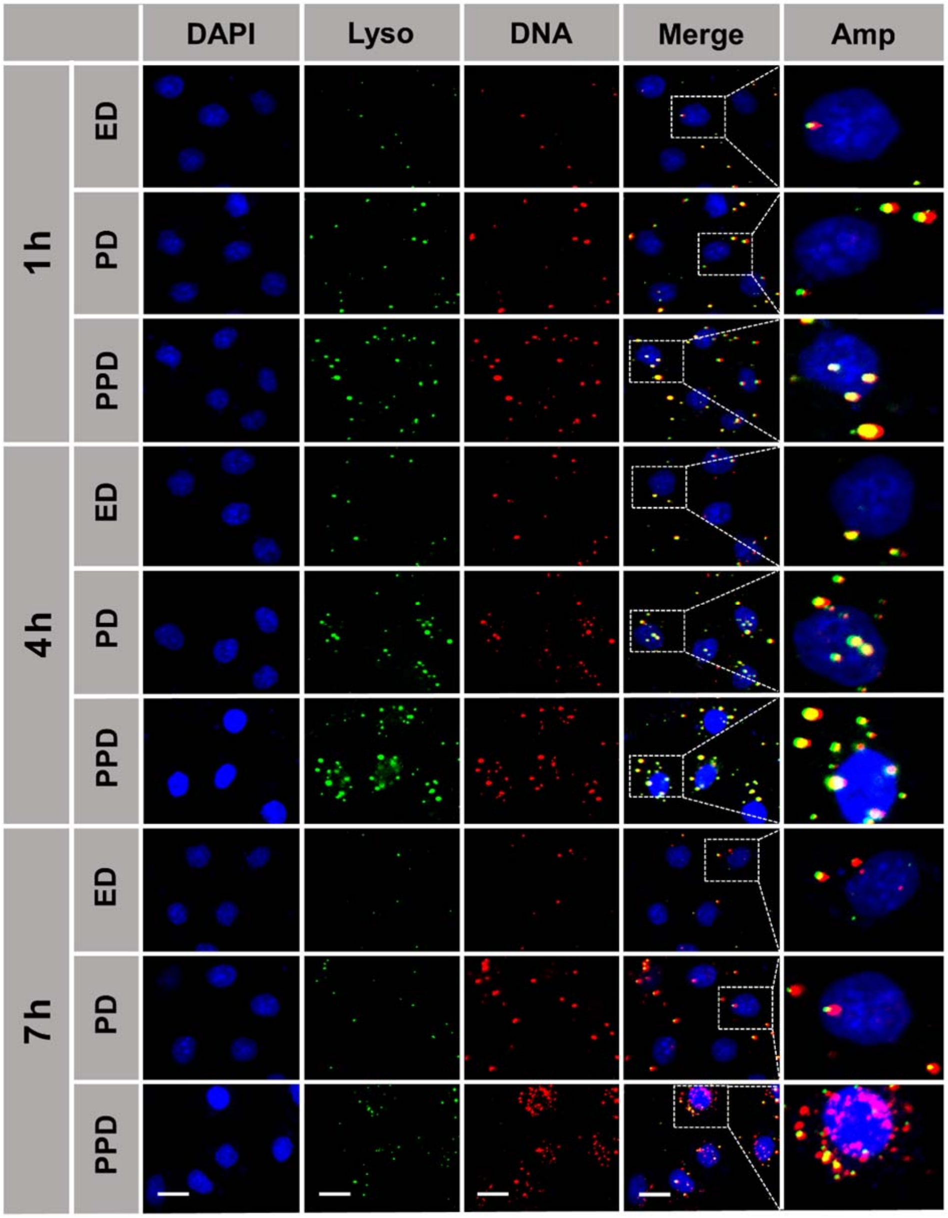

Fig. R14 (i.e., Supplementary Fig. 53 in the Revised Supplementary Information) Representative endo/lysosomal escape images of PPD, PD, and ED at various time pointes (1, 4, and 7 h) in MCF-7 cells, scale bar: 20 μm.

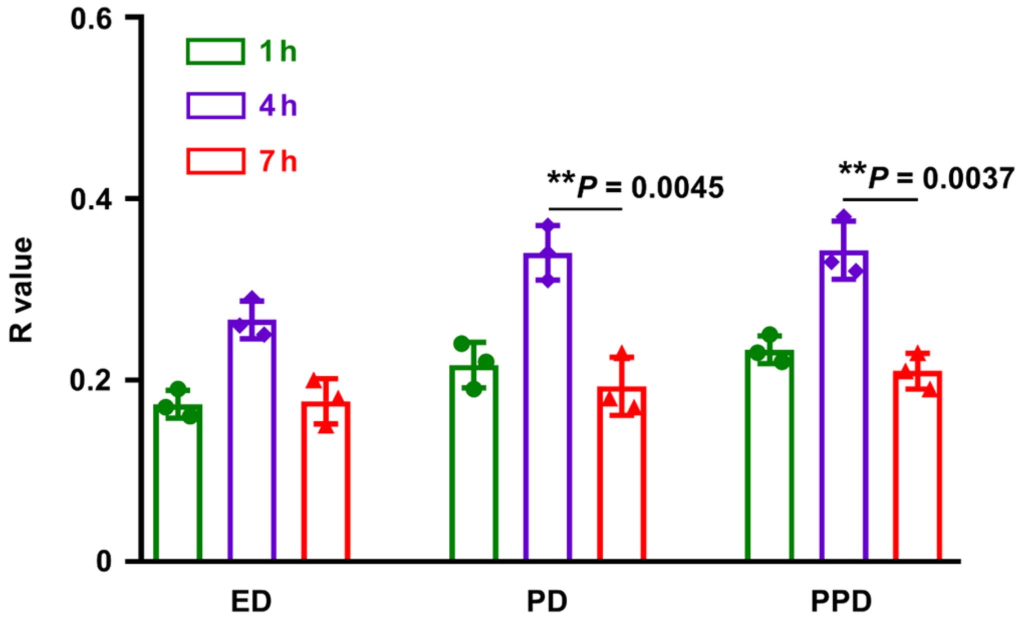

Fig. R15 (i.e., Supplementary Fig. 54 in the Revised Supplementary Information) Correlation coefficient R value obtained from the CLSM images for endo/lysosomal escape ability evaluation in MCF-7 cells. Data are presented as mean ± SD (n=3), $^{**}P<0.01$.

2. The distribution of DNA complex and the expressed protein within tumors need to be characterized.

Response:

Thanks very much for your kind suggestion. The supplementary data were shown in **Fig. R16&R17**.

The detailed descriptions were supplemented as follows: B16F10 or 4T1 tumor-bearing mice were intravenously administered with mPEG-b-PLG/PEI-RT/Cy5-DNA (PPD) complexes. After 24 h, mice were sacrificed and tumor tissue was collected. The frozen sections of tumor were stained with DAPI and observed under fluorescence microscope for the distribution analysis of DNA complexes. As shown in **Fig. R16a&b**, PPD complexes could effectively accumulate in tumor tissue and penetrate within tumor. Additionally, to characterize the distribution of the expressed protein within tumors, the plasmid encoding red fluorescent protein was used as reporter gene, and B16F10 or 4T1 tumor were intravenously administered with mPEG-b-PLG/PEI-RT/*p*DNA complexes. After 48 h, mice were sacrificed and tumor tissue was collected. The frozen sections of tumor were stained with DAPI and observed under fluorescence

microscope for the distribution analysis of expressed protein. As shown in **Fig. R17a&b**, the red fluorescent protein was efficiently expressed within tumor tissue.

The relevant experimental procedure and descriptions were supplemented in the Revised Manuscript (Page 12, Paragraph 2, Line 21-23 and Line 30-31, red words; Page 39, The last paragraph, Line 27-30, red words).

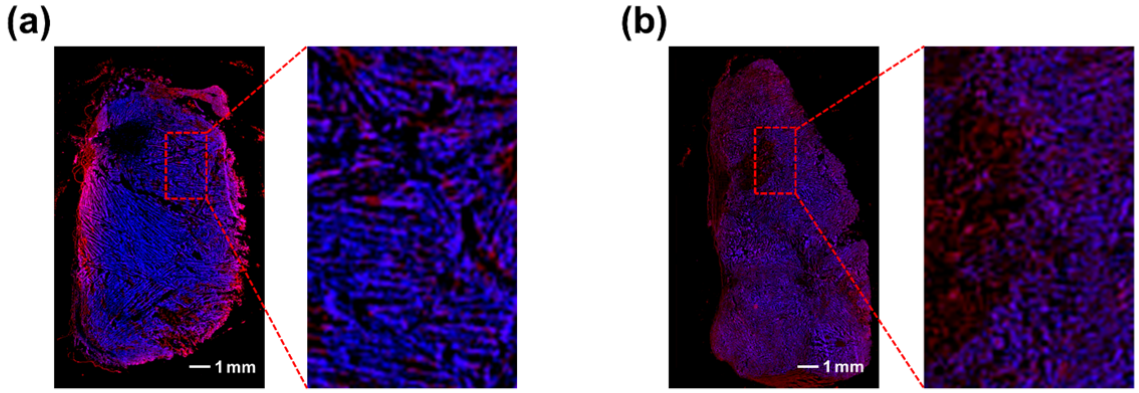

Fig. R16 (i.e., Supplementary Fig. 60 in the Revised Supplementary Information) The distribution of PPD complexes within **(a)** B16F10 and **(b)** 4T1 tumor after intravenously administration.

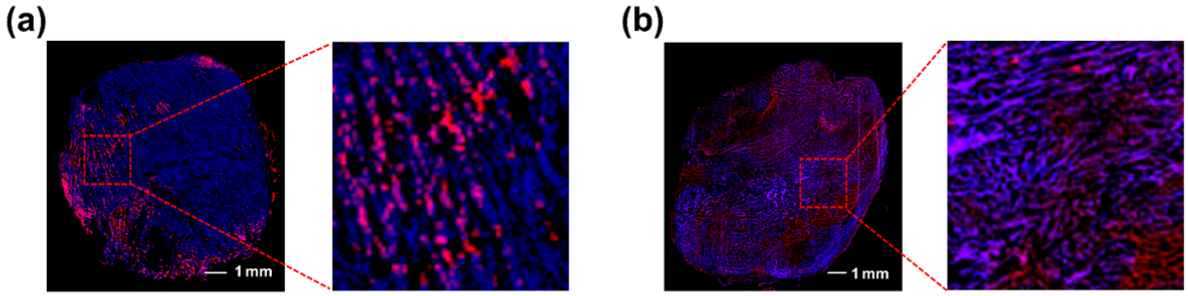

Fig. R17 (i.e., Supplementary Fig. 63 in the Revised Supplementary Information) The distribution of expressed red fluorescent protein within **(a)** B16F10 and **(b)** 4T1 tumor after intravenously administration.

3. Line 28, page 2: the authors claimed that gene therapy-mediated blockage of immune checkpoint could reduce the side effects that was associated with antibody therapy. In order to validate this point, the authors need to provide evidence, or at least cite literatures to support this claim.

Response:

Thanks very much for your kind suggestion. the supplementary data were shown in **Fig. R18&R19**.

The detailed descriptions were supplemented as follows: recently, Recently, it has been reported that PD-1 or PD-L1 targeted antibody therapies may cause severe side effects such as immune related adverse events (irAEs) (Nat. Rev. Rheumatol. 2018, 14, 569-579; N. Engl. J. Med. 2018, 378, 158-168). Among them, Th17 cells were upregulated in inflammatory organ tissues of autoimmune diseases, suggesting the percentage of Th17 cells could serve as a parameter to evaluate the irAEs of immune checkpoint inhibitor mediated immunotherapy (Clin. Sci. 2012, 122, 487-511; Nat. Commun. 2018, 9, 2237-2247). Herein, the anti-tumor efficacy and irAEs of PPD plus Zeb versus aPD-L1 plus Zeb were evaluated. Compared to PPD group with an excellent antitumor efficacy (**Fig. R18**), aPD-L1 could hardly inhibit B16F10 tumor growth. Moreover, PPD plus Zeb group exhibited more efficient antitumor effect than aPD-L1 plus Zeb group (**Fig. R18b&c**). Furthermore, the percentage of Th17 ($CD4^{+}$ $IL17^{+}$) cells in the spleen of tumor-bearing mice was detected at the end of treatment. It was found that aPD-L1 and aPD-L1 plus Zeb group had considerably higher percentage of Th17 cells than that of PBS group, which suggested the possibility of irAEs when the systematical administration of aPD-L1 monoclonal antibody in cancer therapy (**Fig. R18e**). However, the percentages of Th17 cells of PPD and PPD plus Zeb group were similar to PBS group. These results indicated that gene therapy-mediated immune checkpoint blockage could effectively reduce the side effects that was associated with antibody therapy.

The detailed experimental procedure and relevant descriptions were supplemented in the Revised Manuscript (Page 14, Paragraph 4, Line 23-31, red words; Page 15, Paragraph 1, Line 1-7, red words; Page 40, Paragraph 3, Line 21-25, red words).

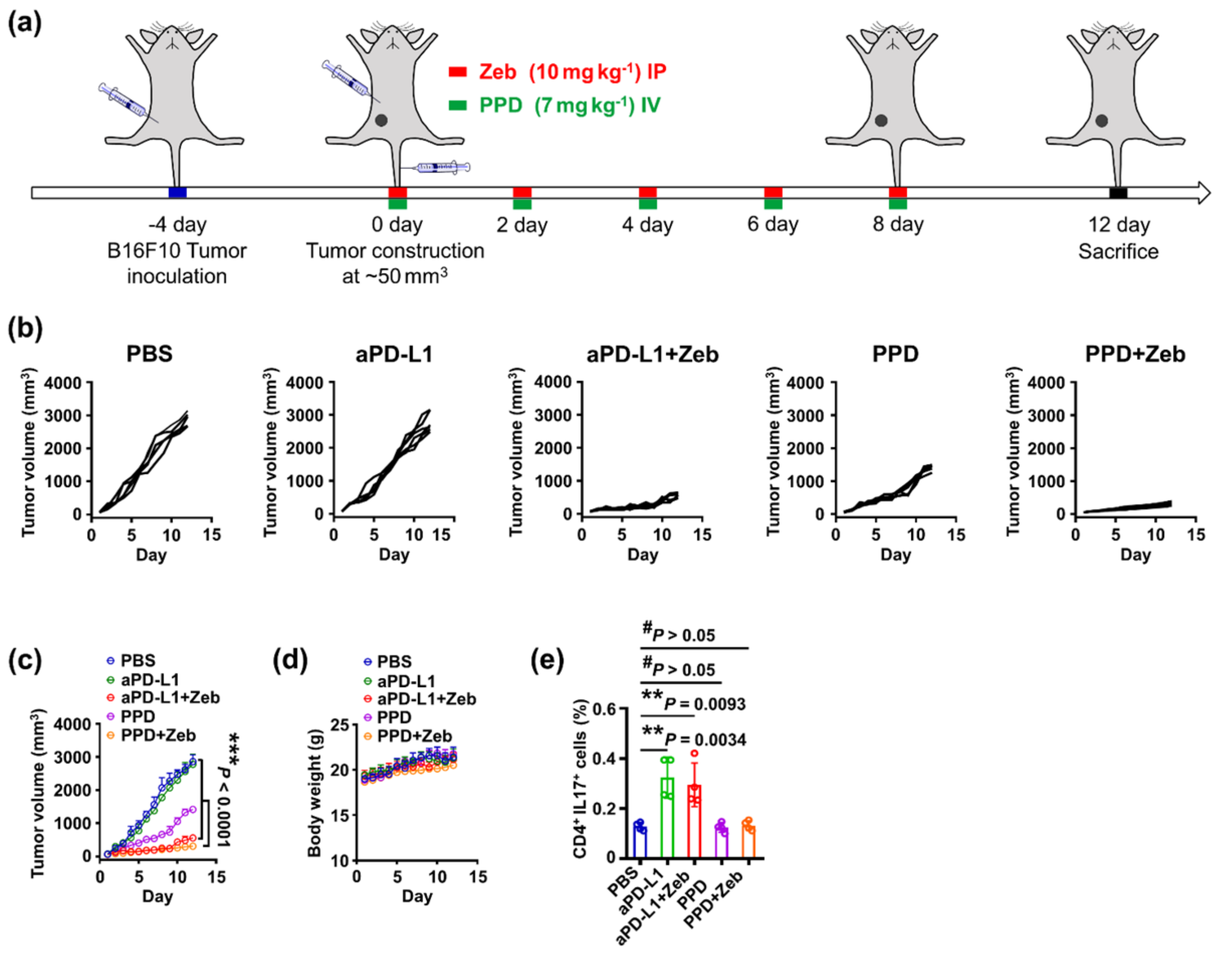

Fig. R18 (i.e., Supplementary Fig. 77 in the Revised Supplementary Information) (a) Therapeutic schedule for PPD plus Zeb or aPD-L1 plus Zeb mediated inhibition of B16F10 tumor growth. **(b)** Individual and **(c)** average tumor growth kinetics in B16F10 tumor-bearing mice receiving various treatments. Data are presented as mean ± SD (n=6), ****P*<0.001. **(d)** Average body weight changes of B16F10 tumor-bearing mice receiving different treatments. Data are presented as mean ± SD (n=6). **(e)** Percentages of Th17 cells in the splenocytes of B16F10 tumor-bearing mice receiving various treatments on day 12. Data are presented as mean ± SD (n=4), #*P*>0.05, ***P*<0.01.

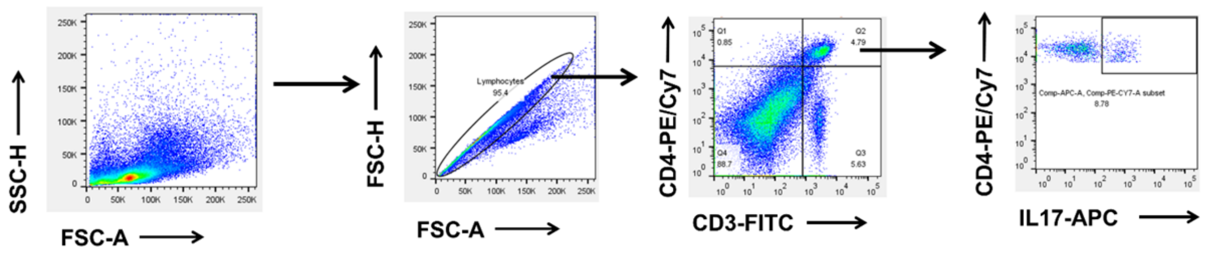

Fig. R19 Flow cytometry gating strategy for the analysis of Th17 cells in the splenocytes.

4. Would the shPD-L1 be expressed in other type of cells other than tumor cells in the tumor microenvironment, for example, the DC cells or fibroblast cells in the tumors?

Response:

Thanks very much for your kind suggestion. the supplementary data were shown in **Fig. R20&R21**.

The detailed descriptions were supplemented as follows: To characterize gene transfection of carrier/shPD-L1 in different cell types in the tumor microenvironment, the levels of PD-L1 expressed on various cell lines in tumors were studied. GFP-B16F10 cells were selected to construct the tumor model. mPEG-b-PLG/PEI-RT/*p*shPD-L1 (**PPD**) ternary complexes were administered intravenously to the tumor-bearing mice (**Fig. R20a**). Then the mice were sacrificed and tumors were collected, the levels of PD-L1 in DCs, macrophages and tumor cells in tumor tissue were analyzed by flow cytometry. As shown in **Fig. R20b&c**, PD-L1 was also expressed on DCs (CD11c+ MHC II^+) and macrophages ($CD11b^+$ $F4/80^+$) in tumor tissue. Compared with PBS group, the percentages of PD-L1 on DCs and macrophages in PPD group did not change significantly. However, the level of PD-L1 on tumor cells (GFP^+) in PPD group was much lower than that in PBS group (**Fig. R20d**). These results suggested that PPD could effectively downregulate the level of PD-L1 on tumor cells instead of DCs and macrophages in the tumor microenvironment.

The detailed experimental procedure and relevant descriptions were supplemented in the Revised Manuscript and Revised Supplementary Information (Page 40, Paragraph 2, Line 3-9, red word; Page S45, Paragraph 1, Line 1-12, red words).

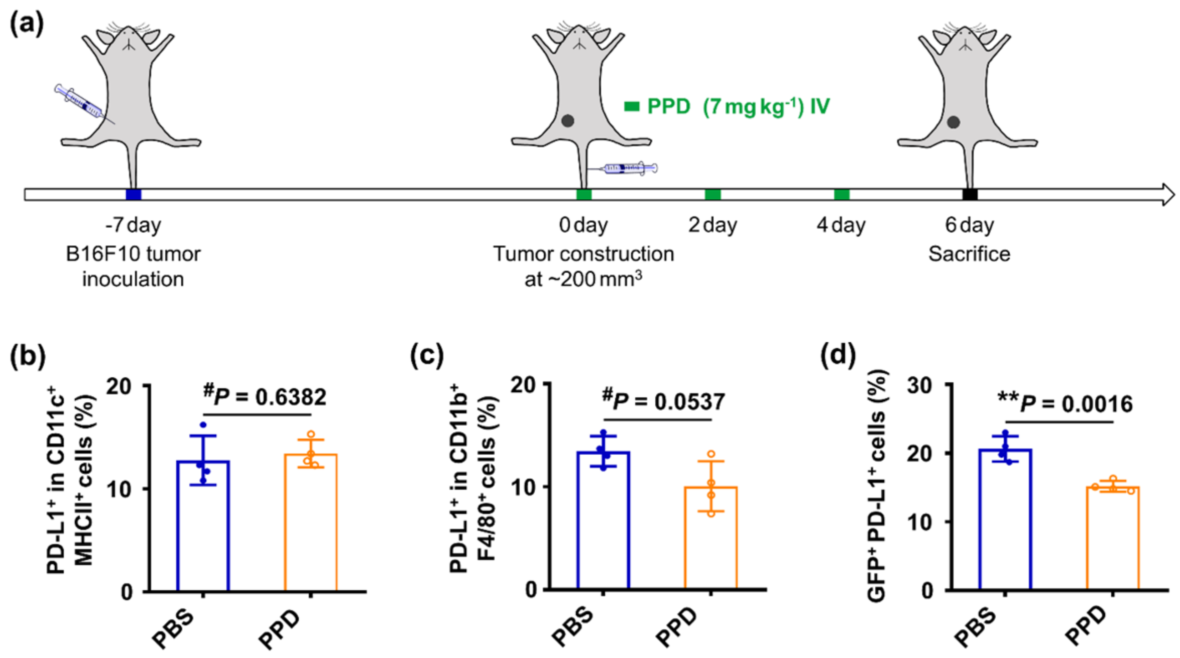

Fig. R20 (i.e., Supplementary Fig. 71 in the Revised Supplementary Information) The levels of PD-L1$^+$ cells in various cells in tumor tissue of B16F10 tumor-bearing mice receiving PPD treatment. **(a)** Therapeutic schedule for PPD mediated PD-L1 down regulation in B16F10 tumor-bearing mice. **(b)** Percentages of PD-L1$^+$ cells in DC (CD11c$^+$MHC II$^+$) cells in tumor tissue. Data are presented as mean ± SD (n=4), $^{\#}P$>0.05. **(c)** Percentages of PD-L1$^+$ cells in Macrophage (CD11b$^+$F4/80$^+$) cells in tumor tissue. Data are presented as mean ± SD (n=4), $^{\#}P$>0.05. **(d)** Percentages of GFP$^+$PD-L1$^+$ cells in tumor tissue. Data are presented as mean ± SD (n=4), $^{**}P$<0.01.

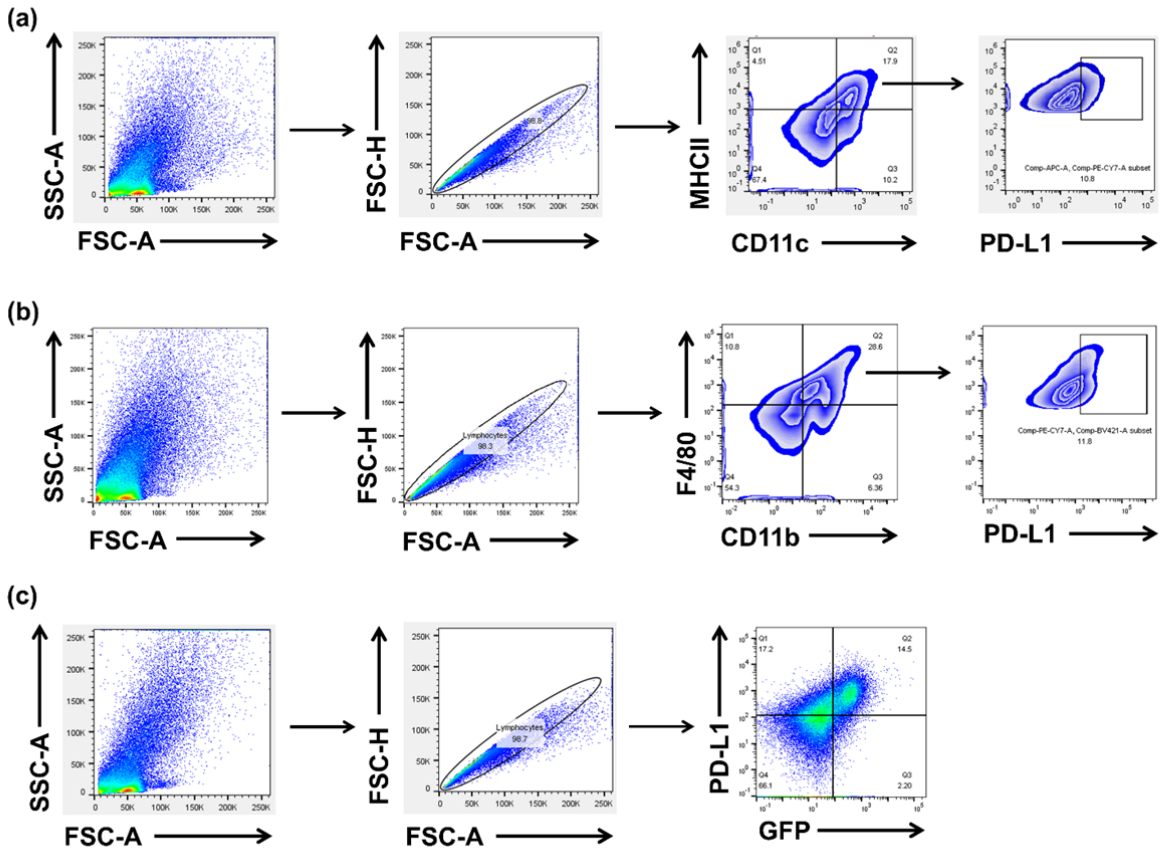

Fig. R21 **(a)** Flow cytometry gating strategy for the analysis of PD-L1^{+} cells in DC (CD11c^{+}MHC II^{+}) cells, **(b)** macrophages (CD11b^{+}F4/80^{+}), and **(c)** tumor cells (GFP^{+}) in tumor tissue of B16F10 tumor-bearing mice receiving PPD treatment.

Reviewer #3, expert in DNMT and HDAC inhibitors and epigenetic regulation (Remarks to the Author):

The current manuscript focuses on optimizing a gene delivery system, termed as PPD, that can be used to efficiently deplete PD-L1 in cancer cells. The authors have further proposed a DNMT inhibitor-based combinatorial strategy that potentially synergizes with the PPD approach to improve anticancer immune response. Overall, this is a well-structured manuscript; an incredible amount of experimental evidence has reinforced the importance of this work, both in terms of technological development and therapeutic application. Below are a few questions that need to be clarified:

1. Experiments were carried out on four cancer cell lines originating from mouse and human species. What is the rationale for using them in this study?

Response: Thanks very much for your suggestion. We hope that PPD plus Zeb treatment strategy can be used to treat cancer patients in the end, but it is well known that a reliable treatment

scheme needs to go through a series of stages before treating cancer patients. In this work, we carried out a preliminary exploration for that. Firstly, two mouse derived cell lines and two human derived cell lines were selected for verifying the effectiveness of the PPD plus Zeb treatment. Finally, PPD plus Zeb mediated tumor immunotherapy in humanized mice was further verified and supplemented in the Revised Manuscript (Page 29, Paragraph 2, Line 12-31, red words; Page 30, Paragraph 1, Line 1-2, red words).

2. Page 8 (Fig. 2f and 2g) – In comparison with Fig. 2h, which time point was applied to Fig. 2f and 2g as well as Supplementary Fig. 42-47?

Response:

Thanks very much for your kind suggestion. In Fig. 2h, the time point was 1, 4 and 7 h, respectively. The time point of Fig. 2f and 2g was 3 h. Although the time points of Fig. 2h are different from those of Fig. 2g and 2f, PPD shows the same endocytosis trend, namely, PPD had more significant uptake efficiency than PD and ED in various cell lines at the same point. The relevant time points were supplemented in the notes of Fig 2 and Supplementary Fig 49, 51, and 53 in the Revised Manuscript and Revised Supplementary Information.

3. Page 9 (Fig. 2h and Supplementary Fig. 49/51/53) – "Images were taken at different times (1, 4, and 7 h)". What does each time point mean? Also, the experimental procedure for studying endosomal escape shows no information about Lysotracker Green staining.

Response:

Thanks very much for your suggestion. The procedure of labeling endo/lysosome was supplemented as follows: B16F10, 4T1, HeLa, or MCF-7 cells were seeded in 6-well plates containing coverslips at a density of 1×10^5 cells per well and incubated for 24 h. Then the culture medium was replaced with fresh culture medium and carrier/Cy5-DNA complexes were added and incubated for 1, 4, and 7 h. After that, the cells were washed with PBS and fixed with 4% paraformaldehyde for 10 min. Nucleus were stained with 1 mg mL^{-1} of DAPI (1.5 μL per well) for 10 min, and endo/lysosome were stained with 75 nM of Lysotracker Green for 1 h. Finally, the coverslips were taken out and placed on the glass slides, enclosed with glycerol. The samples were observed by confocal laser scanning microscopy (CLSM) (ZEISS LSM780, Germany) and the correlation coefficient R values were obtained from CLSM images (ZEN software). The relevant experimental procedure was supplemented in the Revised Manuscript (Page 38, Paragraph 2, Line 4-13, red words).

4. Page 11 (Supplementary Fig. 57) – There is no information showing the half-life of circulatory DNA under individual conditions.

Response:

Thanks very much for your kind suggestion. the supplementary data were shown in **Table R1**.

the detailed descriptions were supplemented as follows: The half-life of circulatory DNA under individual conditions was supplemented. As shown in **Table R1**, the half-life of circulatory DNA in PPD group was 0.71 h, which was remarkedly longer than that of other groups. Moreover, the area under the curve ($AUC_{0\text{-}t}$) of PPD was 8.97 μg/mL h, which was also much higher than that of other groups.

The relevant descriptions were supplemented in the Revised Manuscript (Page 12, Paragraph 1, Line 9-12, red words).

Table R1 (i.e., Supplementary Table 3 in the Revised Supplementary Information) Pharmacokinetic parameters of free DNA, ED, PD, and PD, PPD.

Entry	$t_{1/2}$ [a] (h)	**A**$UC_{0\text{-}t}$ [b] (μg/mL h)
DNA	0.13 ± 0.02	1.45 ± 0.02
ED	0.16 ± 0.02	1.59 ± 0.02
PD	0.16 ± 0.03	1.77 ± 0.06
PPD	0.71 ± 0.13	8.97 ± 0.61

[a] $t_{1/2}$: half-life.

[b] $AUC_{0\text{–}t}$: area under the DNA concentration-time curve from 0 to 24 h in plasma.

5. Page 11 (Supplementary Fig. 58 and 59) – There is no information in the main text, describing the tumor model(s) used in the biodistribution study of carries/DNA complexes.

Response: Thanks very much for your kind suggestion. The tumor model mentioned in original manuscript was 4T1 tumor. In addition, the biodistribution and *in vivo* transfection of carries/DNA complexes in B16F10 tumor model were supplemented in the Revised Manuscript (**Fig. R22&23**).

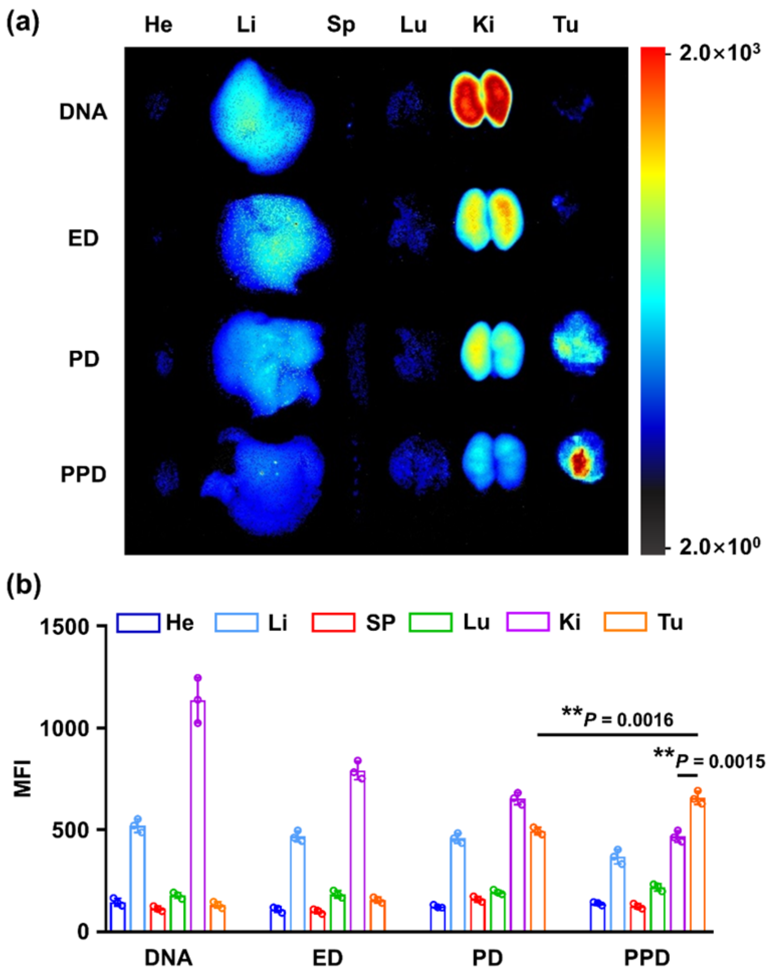

Fig. R22 (i.e., Supplementary Fig. 58 in the Revised Supplementary Information) (a) Representative images of biodistribution in heart, liver, spleen, lung, kidney, and tumor after B16F10 tumor-bearing mice intravenously administered with DNA, ED, PD, and PPD, respectively. **(b)** Mean fluorescence intensity (MFI) of biodistribution in heart, liver, spleen, lung, kidney, and tumor after B16F10 tumor-bearing mice intravenously administered with DNA, ED, PD, and PPD, respectively. Data are presented as mean ± SD (n=3), $^{**}P<0.001$.

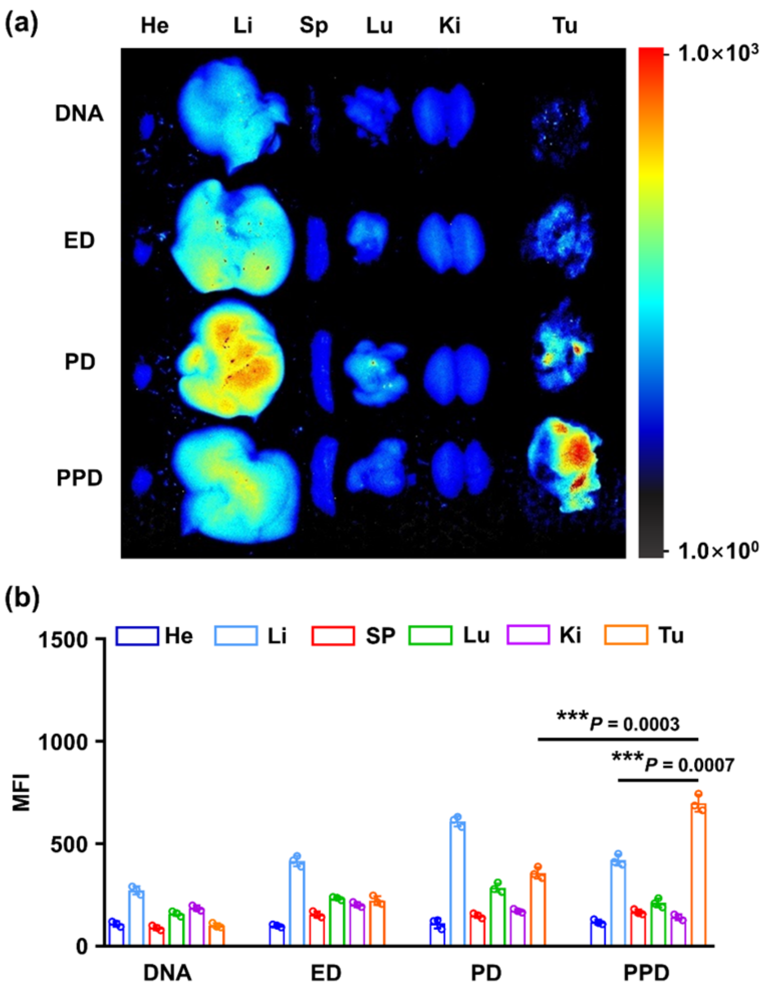

Fig. R23 (i.e., Supplementary Fig. 61 in the Revised Supplementary Information) (a) Representative images of red fluorescence protein expression in heart, liver, spleen, lung, kidney, and tumor after B16F10 tumor-bearing mice intravenously administered with DNA, ED, PD, and PPD, respectively. **(b)** Mean fluorescence intensity (MFI) of the in vivo transfection in heart, liver, spleen, lung, kidney, and tumor after B16F10 tumor-bearing mice intravenously administered with DNA, ED, PD, and PPD, respectively. Data are presented as mean ± SD (n=3), ***P<0.001.

6. Page 12 (Fig. 3) – No strong evidence shows that Zebularine can "increase MHC I expression". RT-qPCR should be performed to analyze HLA-A/B/C and PD-L1 mRNA expression in human cancer cells (as seen in Supplementary Fig. 87).

Response:

Thanks very much for your kind suggestion. Based on your suggestion, the RT-qPCR assay to detect HLA-A/B and PD-L1 mRNA expression was supplemented in human cancer cells (HeLa, HepG2, and A549 cells). As shown in **Fig. R24a&b**, Zeb could significantly upregulate the levels of HLA-A and HLA-B in HeLa cells. Additionally, Zeb could also remarkably increase PD-L1

expression on HeLa cells (**Fig. R24c**). The similar results were obtained in HepG2 and A549 cells (**Fig. R25&26**). The relevant descriptions were supplemented in the Revised Manuscript (Page 13, Paragraph 2, Line 9-12, red words).

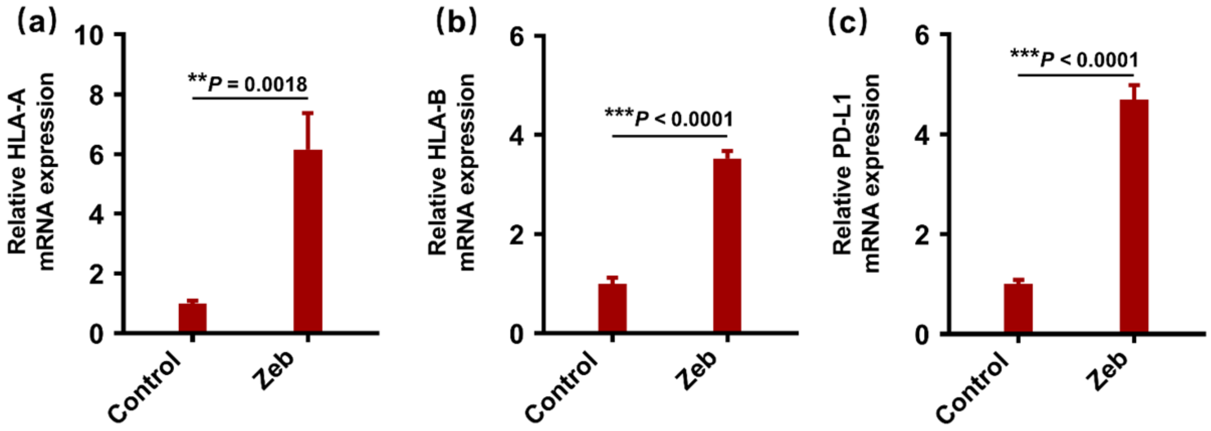

Fig. R24 (i.e., Supplementary Fig. 66 in the Revised Supplementary Information) (a) Relative HLA-A mRNA, **(b)** HLA-B mRNA, and **(c)** PD-L1 mRNA expression in HeLa cells treated with 0.5 mg/mL of Zeb by RT-qPCR assay. Data are presented as mean ± SD (n=3), **P*<0.05, ***P*<0.01, ****P*<0.001.

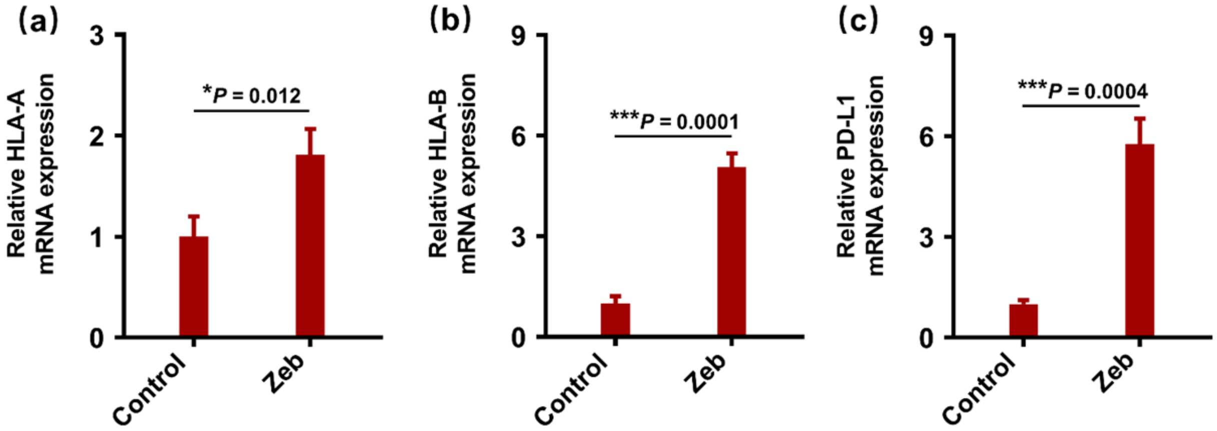

Fig. R25 (i.e., Supplementary Fig. 67 in the Revised Supplementary Information) (a) Relative HLA-A mRNA, **(b)** HLA-B mRNA, and **(c)** PD-L1 mRNA expression in HepG2 cells treated with 0.5 mg/mL of Zeb by RT-qPCR assay. Data are presented as mean ± SD (n=3), *P<0.05, **P<0.01, ***P<0.001.

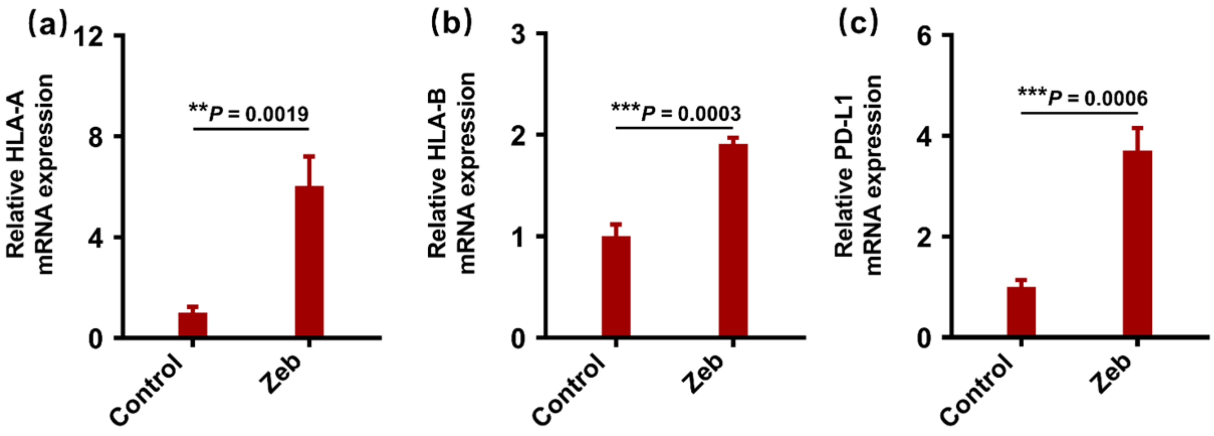

Fig. R26 (i.e., Supplementary Fig. 68 in the Revised Supplementary Information) **(a)** Relative HLA-A mRNA, **(b)** HLA-B mRNA, and **(c)** PD-L1 mRNA expression in A549 cells treated with 0.5 mg/mL of Zeb by RT-qPCR assay. Data are presented as mean ± SD (n=3), $^{**}P<0.01$, $^{***}P<0.001$.

7. Page 12 (Fig. 3 and illustrated in Fig. 1) – No direct evidence suggests that MHC I plays a key role in zebularine cooperation with PPD treatment. Does depletion of certain MHC I molecule(s) protect tumor growth against the zebularine/PPD treatment?

Response:

Thanks very much for your kind suggestion. The supplementary data were shown in **Fig R27&28**.

The detailed descriptions were supplemented as follows: to demonstrate the role of MHC I molecules in Zeb plus PPD treatment, the siRNA silencing MHC I molecules (abbreviated as siH2-K^b) was designed. As shown in **Fig. R27**, compared with the control (untreated) group, the relative H2-K^b mRNA expression in mPEG-b-PLG/PEI-RT3/siH2-K^b (denoted as PP/siH2-K^b) group was significantly downregulated in B16F10 cells. Moreover, the antitumor effect of PPD plus Zeb accompany with MHC I depletion was studied in B16F10 tumor-bearing mice. PP/siH2-K^b complexes were injected intratumorally along with PPD plus Zeb treatment. As shown in **Fig. R28b&e**, the antitumor efficacy of PPD plus Zeb was weakened after MHC I depletion in B16F10 tumor model. These results suggested that depletion of certain MHC I molecule(s) protected tumor growth against the zebularine/PPD treatment.

The relevant description was supplemented in the Revised Manuscript (Page 14, Paragraph 2, Line 3-12, red words).

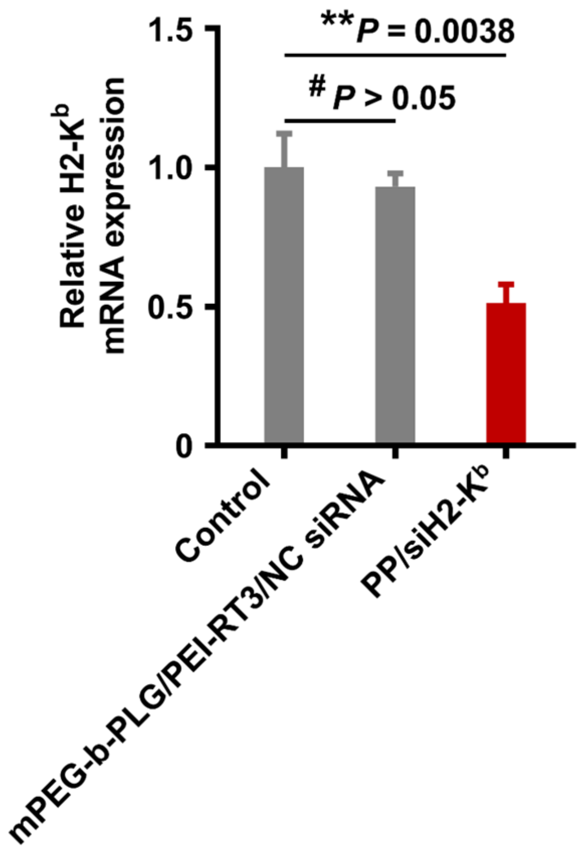

Fig. R27 ((i.e., Supplementary Fig. 72 in the Revised Supplementary Information)) Relative H2-K^b mRNA expression in B16F10 cells treated with PP/siH2-K^b complexes by RT-qPCR assay. Data are presented as mean ± SD (n=3), $^{\#}P>0.05$, $^{*}P<0.05$, $^{**}P<0.01$.

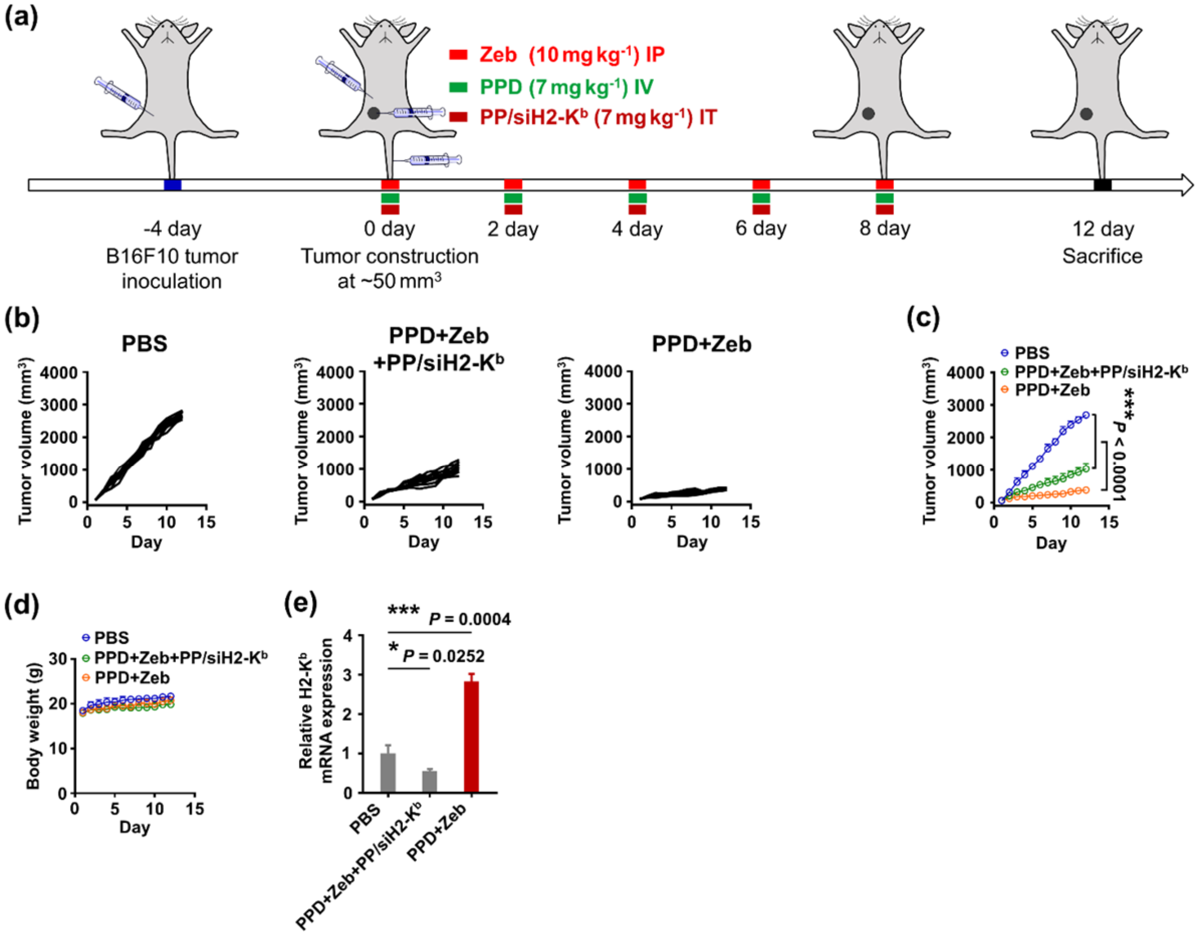

Fig. R28 (**i.e., Supplementary Fig. 73 in the Revised Supplementary Information**) **(a)** Therapeutic schedule for PPD plus Zeb accompanying with MHC I depletion mediated B16F10 tumor-bearing mice. **(b)** Individual and **(c)** average tumor growth kinetics in B16F10 tumor-bearing mice receiving various treatments. Data are presented as mean ± SD (n=8), ***P<0.001. **(d)** Average body weight changes of B16F10 tumor-bearing mice receiving different treatments (n=8). **(e)** Relative H2-K^b mRNA in B16F10 tumor-bearing mice after treatment by RT-qPCR assay. Data are presented as mean ± SD (n=3), *P<0.05, **P<0.01, ***P<0.001.

8. Given that several DNMTi are under clinical studies, what is the rationale for using zebularine in the current research? Does any other DNMTi exhibit the similar effects in combination with PPD?

Response:

Thanks very much your kind suggestion. The supplementary data were shown in **Fig R29&R30**.

The relevant descriptions were supplemented as follows: as shown in **Fig. R29**, compared with other DNMTi such as azacytidine (AC) and deoxyazacytidine (DAC), Zeb showed a better

biocompatibility in B16F10 and 4T1 cells. Moreover, the antitumor effect of PPD plus AC or DAC were evaluated in B16F10 tumor model. As shown in **Fig. R30b&c**, PPD plus AC or DAC showed an excellent antitumor efficacy which was comparable to PPD plus Zeb. Moreover, in the case of equivalent antitumor efficacy, the body weights in PPD plus AC or PPD plus DAC group were significantly reduced during treatment while PPD plus Zeb group exhibited the negligible changes, which indicated Zeb had better *in vivo* biological safety than AC and DAC (**Fig. R30d**).

The relevant descriptions were supplemented in the Revised Manuscript (Page 15, Paragraph 2, Line 8-15, red words).

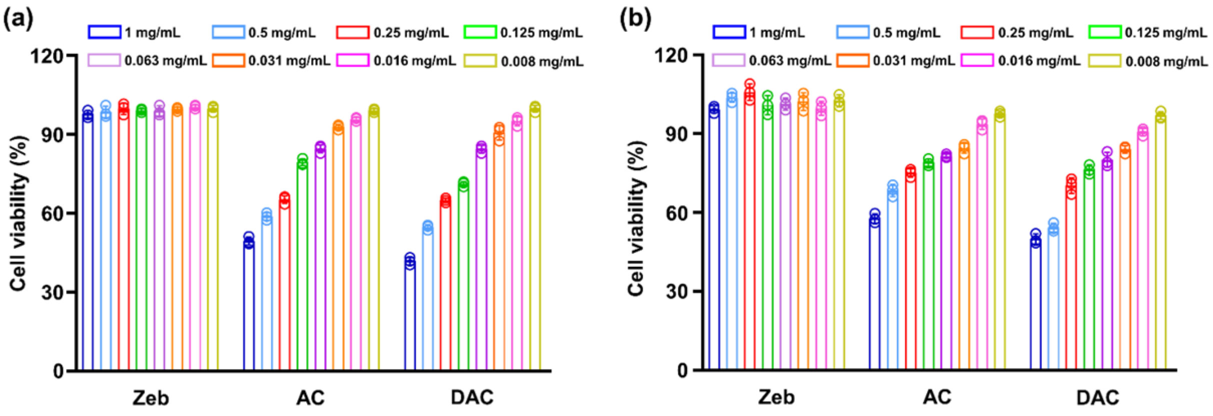

Fig. R29 (i.e., Supplementary Fig. 78 in the Revised Supplementary Information) The cell viability of Zeb, AC, and DAC at various concentrations in (a) B16F10 and (b) 4T1 cells. Data are presented as mean ± SD (n=3).

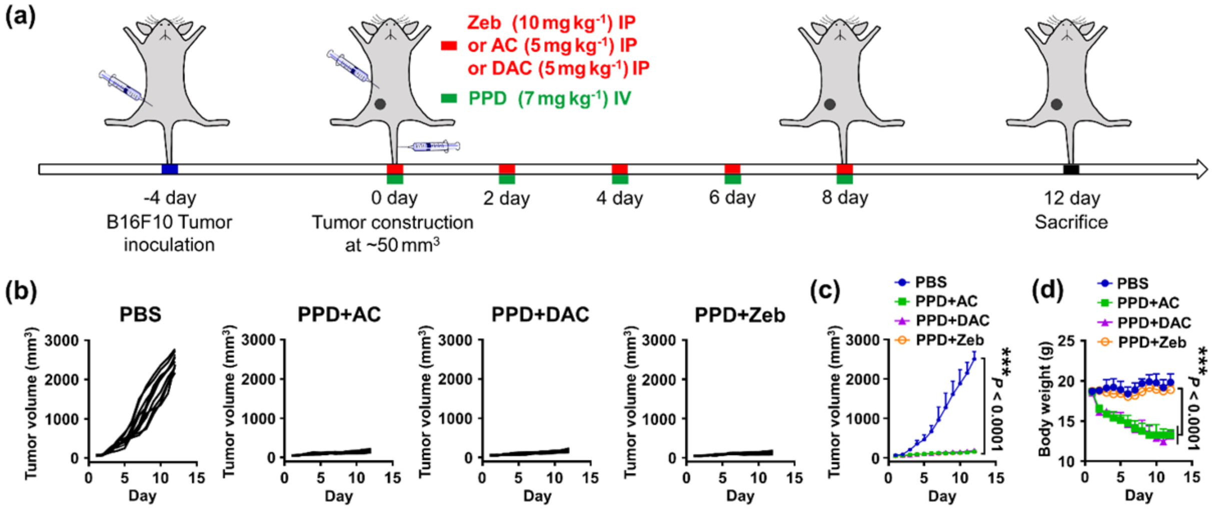

Fig. R30 (i.e., Supplementary Fig. 79 in the Revised Supplementary Information)) (a) Therapeutic schedule for PPD plus DNMTi mediated B16F10 tumor-bearing mice. **(b)** Individual and **(c)** average tumor growth kinetics in B16F10 tumor-bearing mice receiving various treatments.

Data are presented as mean ± SD (n=10), ***P<0.001. **(d)** Average body weight changes of B16F10 tumor-bearing mice receiving different treatments. Data are presented as mean ± SD (n=10).

9. Considering the zebularine/PPD combinatorial treatment as "a novel therapeutic scheme for cancer immunotherapy", the authors should offer some insights into the mechanism of drug action – for example, performing RNA-seq to uncover major target genes, pathways and/or biological processes in response to zebularine and PPD alone or in combination. This information may strengthen the central concept of the current research (Fig. 1) and support the future investigations aimed at inhibiting DNMT activity in immunotherapy.

Response:

Thanks very much for your kind suggestion. The supplementary data were shown in **Fig R31**.

The detailed descriptions were supplemented as follows: to uncover major target genes, RT-qPCR assay of B16F10 tumor model was carried out after PPD plus Zeb treatment and the heatmap of mRNA expression of relevant genes was provided. As shown in **Fig R31**, these cancer testis antigen (CTA) genes including SSX-9, MAGE-A3, SSX-b2, Taf7l, Ctcfl, and Lipa were significantly upregulated in PPD plus Zeb treatment compared with PBS group. Additionally, PPD plus Zeb treatment also increased the levels of MHC I molecules (H2-K^b) and immunoactivated cytokines including IFN-γ, TNF-α, GmzyB, IL-6, and IL-1β. Moreover, PD-L1 and immunosuppressive cytokines including IL-10 and TGF-β were significantly downregulated after PPD plus Zeb treatment. These results suggested that PPD plus Zeb combination therapy could upregulate the levels of some CTA genes (SSX-9, MAGE-A3, SSX-b2, Taf7l, Ctcfl, and Lipa) accompanying with enhancement of MHC I (H2-D^b and H2-K^b) and downregulate the PD-L1 expression in tumor tissue, resulting in the increase of immunoactivated cytokines and decrease of immunosuppressive cytokines, all of which were beneficial to inhibit tumor growth.

The relevant description was supplemented in the Revised Manuscript (Page 18, Paragraph 2, Line 21-31, red words).

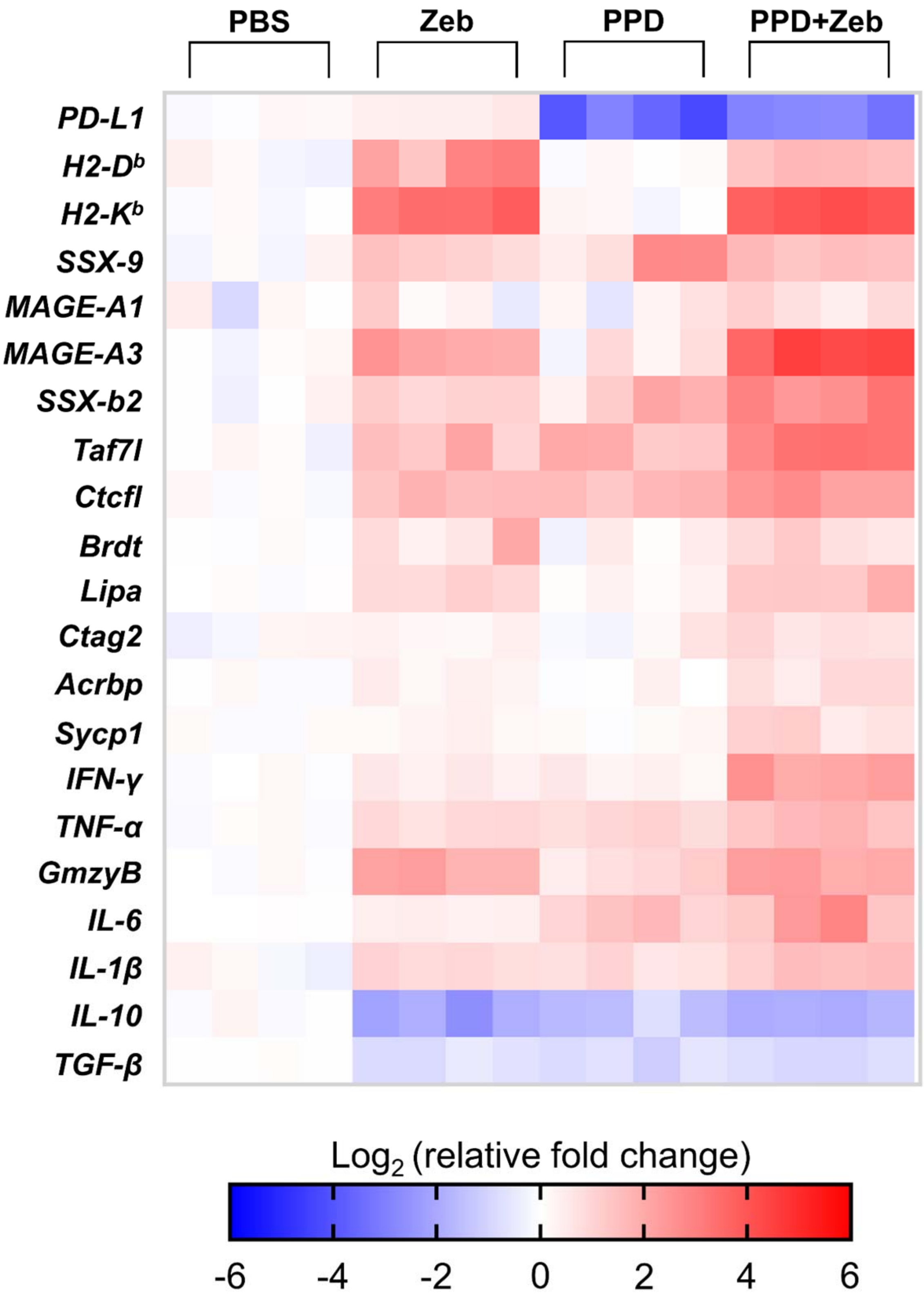

Fig. R31 (i.e., Supplementary Fig. 85 in the Revised Supplementary Information) Heatmap of relative mRNA expression of PD-L1, MHC I (H2-D^b and H2-K^b), CTAs (SSX-9, SSX-b2, MAGE-A1, MAGE-A3, Tf7al, Ctcfl, Brdt, Lipa, Ctag2, Acrbp, and Sycp1), immunoactivated

cytokines (IFN-γ, TNF-α, GmzyB, IL-6, and IL-1β), and immunosuppressive cytokines (IL-10 and TGF-β) in tumor tissues of B16F10 tumor-bearing mice at the end of treatment.

10. Page 19 (Fig. 5n-q) – Evidence for "personalized immunotherapy" is relatively weak. The similar experiments would ideally need to be repeated on a different pair of cell lines, such as 4T1 vs MCF7.

Response:

Thanks very much for your kind suggestion. As 4T1 cells are mouse-derived (BABL/c mice) and MCF-7 cells are human derived, 4T1 tumor-bearing mice could not be re-implanted with MCF-7 cells. Moreover, the similar experiments were supplemented on other pairs of mouse derived cell lines, namely, B16F10 vs MC38 (murine colon carcinoma), B16F10 vs LLC (Lewis lung cancer), and 4T1 vs CT26 cells. The supplementary data were shown in **Fig R1&R32**.

The detailed descriptions were supplemented as follows: As shown in **Fig. R1**, PPD plus Zeb group could efficiently inhibit B16F10 tumor relapse compared with control group (i.e., age- and body-weight-matched healthy mice). According to the immune cell analysis in the spleen of B16F10 tumor-bearing mice after treatment, the percentage of TEM cells and TEMs/TCMs ratio in the spleen of B16F10 tumor-bearing mice in PPD plus Zeb group were much higher than that in the control group (**Fig. R1e-j)**. These results demonstrated that PPD plus Zeb combination therapy could evoke tumor-specific immune memory effect, which considerably inhibited B16F10 tumor relapse. Nevertheless, when B16F10 tumor-bearing mice after PPD plus Zeb treatment were re-implanted with MC38 or LLC tumor, the tumor growth curves and tumor weight of distant tumors showed negligible differences between PPD plus Zeb group and control group (**Fig. R1k-p**). Moreover, PPD plus Zeb group could efficiently inhibit 4T1 tumor relapse compared with control group (**Fig. R32b-d**). Additionally, the percentage of TEM cells and TEMs/TCMs ratio in the spleen of 4T1 tumor-bearing mice in PPD plus Zeb group were much higher than that in the control group (**Fig. R32e-j**). However, when 4T1 tumor-bearing mice after PPD plus Zeb treatment were re-implanted with CT26 tumor, the tumor growth curves and tumor weight of distant tumors showed negligible differences between PPD plus Zeb group and control group (**Fig. R32k-m**). On the basis of the above results, PPD plus Zeb combination therapy initiated tumor specific immune memory response and suggested its potential for personalized immunotherapy.

The relevant descriptions were supplemented in the Revised Manuscript (Page 21, Paragraph 1, Line 9-16, red words; Page 21, Paragraph 2, Line 17-25, red words).

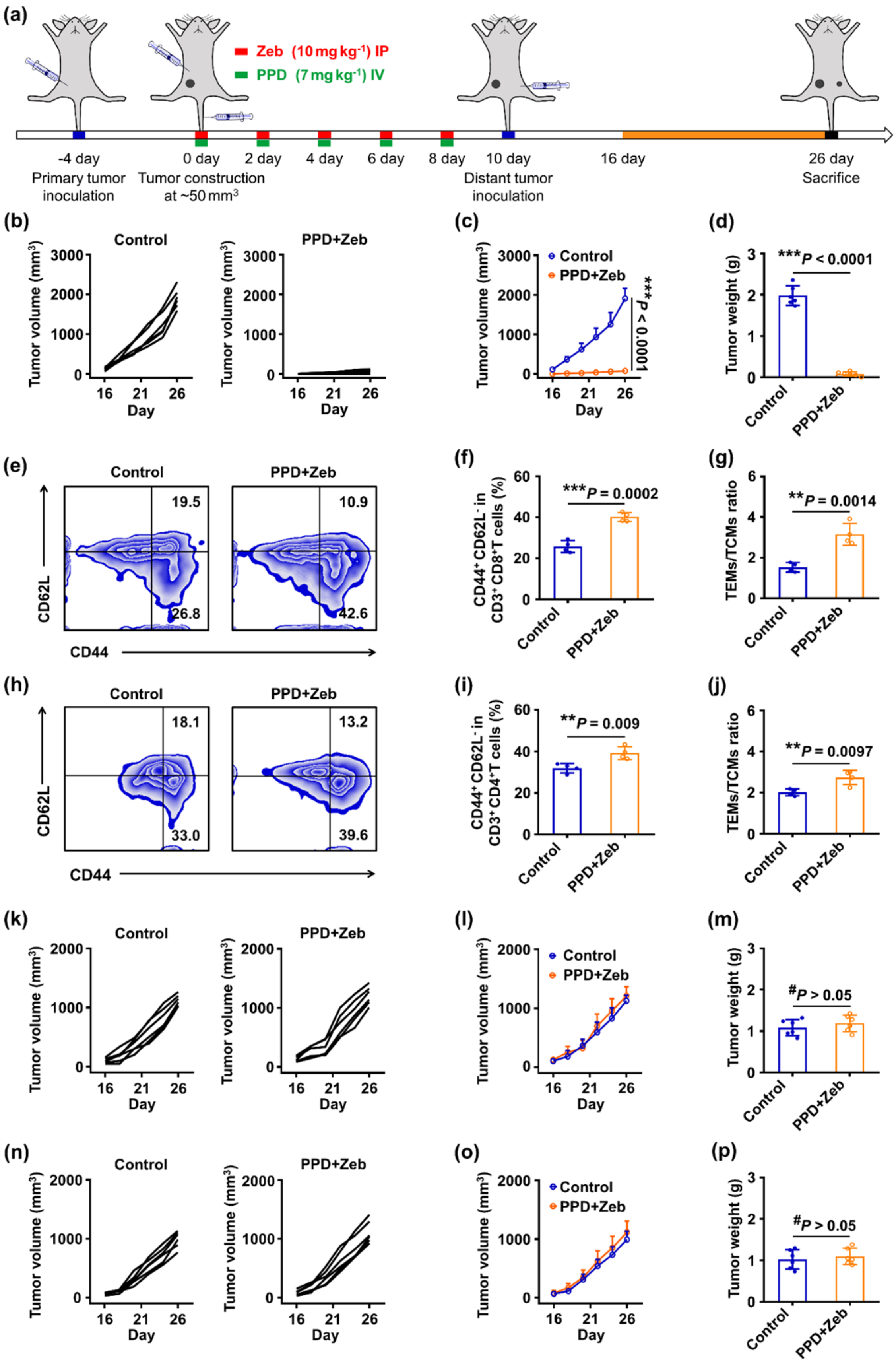

(a)
Zeb (10 mg kg-1) IP
PPD (7 mg kg-1) IV
-4 day
Primary tumor inoculation
0 day
Tumor construction at ~50 mm3
2 day
4 day
6 day
8 day
10 day
Distant tumor inoculation
16 day
26 day
Sacrifice
(b)
Control
PPD+Zeb
Tumor volume (mm3)
Day
(c)
Control
PPD+Zeb
***P < 0.0001
(d)
***P < 0.0001
Tumor weight (g)
(e)
CD62L
CD44
19.5
26.8
10.9
42.6
(f)
***P = 0.0002
CD44+CD62L- in CD3+CD8+T cells (%)
(g)
**P = 0.0014
TEMs/TCMs ratio
(h)
18.1
33.0
13.2
39.6
(i)
**P = 0.009
CD44+CD62L- in CD3+CD4+T cells (%)
(j)
**P = 0.0097
(k)
(l)
(m)
#P > 0.05
(n)
(o)
(p)
#P > 0.05

Fig. R1 (i.e. Fig. 5 in the Revised Manuscript) PPD combined with Zeb inhibited B16F10 tumor relapse and initiated tumor specific immune memory effect. (a) Therapeutic schedule for PPD plus Zeb mediated inhibition of tumor relapse. **(b)** Individual and **(c)** average tumor growth kinetics in distant B16F10 tumor-bearing mice receiving PPD plus Zeb treatment. Data are presented as mean ± SD (n=6), $^{***}P<0.001$. Age- and body-weight-matched healthy mice were used as control. **(d)** Average tumor weight of distant B16F10 tumor-bearing mice receiving PPD plus Zeb treatment. Data are presented as mean ± SD (n=6), $^{***}P<0.001$. **(e)** Representative flow cytometric analysis of TEM ($CD44^+ CD62L^-$) and TCM ($CD44^+ CD62L^{+)}$ cells gating on $CD8^+$ T cells in spleen of B16F10 tumor-bearing mice at the end of treatment. Relative quantification of **(f)** TEM cells gating on $CD8^+$T cells and **(g)** ratio of TEMs/TCMs gating on $CD8^+$T cells in the spleen of B16F10 tumor-bearing mice at the end of treatment. Data are presented as mean ± SD (n=4), $^{**}P<0.01$, $^{***}P<0.001$. **(h)** Representative flow cytometric analysis of TEM ($CD44^+ CD62L^-$) and TCM ($CD44^+ CD62L^{+)}$ cells gating on $CD4^+$ T cells in spleen of B16F10 tumor-bearing mice at the end of treatment. Relative quantification of **(i)** TEM cells gating on $CD8^+$T cells and **(j)** ratio of TEMs/TCMs gating on $CD8^+$T cells in the spleen of B16F10 tumor-bearing mice at the end of treatment. Data are presented as mean ± SD (n=4), $^{**}P<0.01$, $^{***}P<0.001$. **(k)** Individual and **(l)** average tumor growth kinetics in distant MC38 (murine colon carcinoma) tumor-bearing mice receiving PPD plus Zeb treatment. Data are presented as mean ± SD (n=6), $^{\#}P>0.05$. Age- and body-weight-matched healthy mice were used as control. **(m)** Average tumor weight of distant MC38 tumor-bearing mice receiving PPD plus Zeb treatment. Data are presented as mean ± SD (n=6), $^{\#}P>0.05$. **(n)** Individual and **(o)** average tumor growth kinetics in distant LLC (Lewis lung cancer) tumor-bearing mice receiving PPD plus Zeb treatment. Data are presented as mean ± SD (n=6), $^{\#}P>0.05$. Age- and body-weight-matched healthy mice were used as control. **(m)** Average tumor weight of distant LLC tumor-bearing mice receiving PPD plus Zeb treatment. Data are presented as mean ± SD (n=6), $^{\#}P>0.05$.

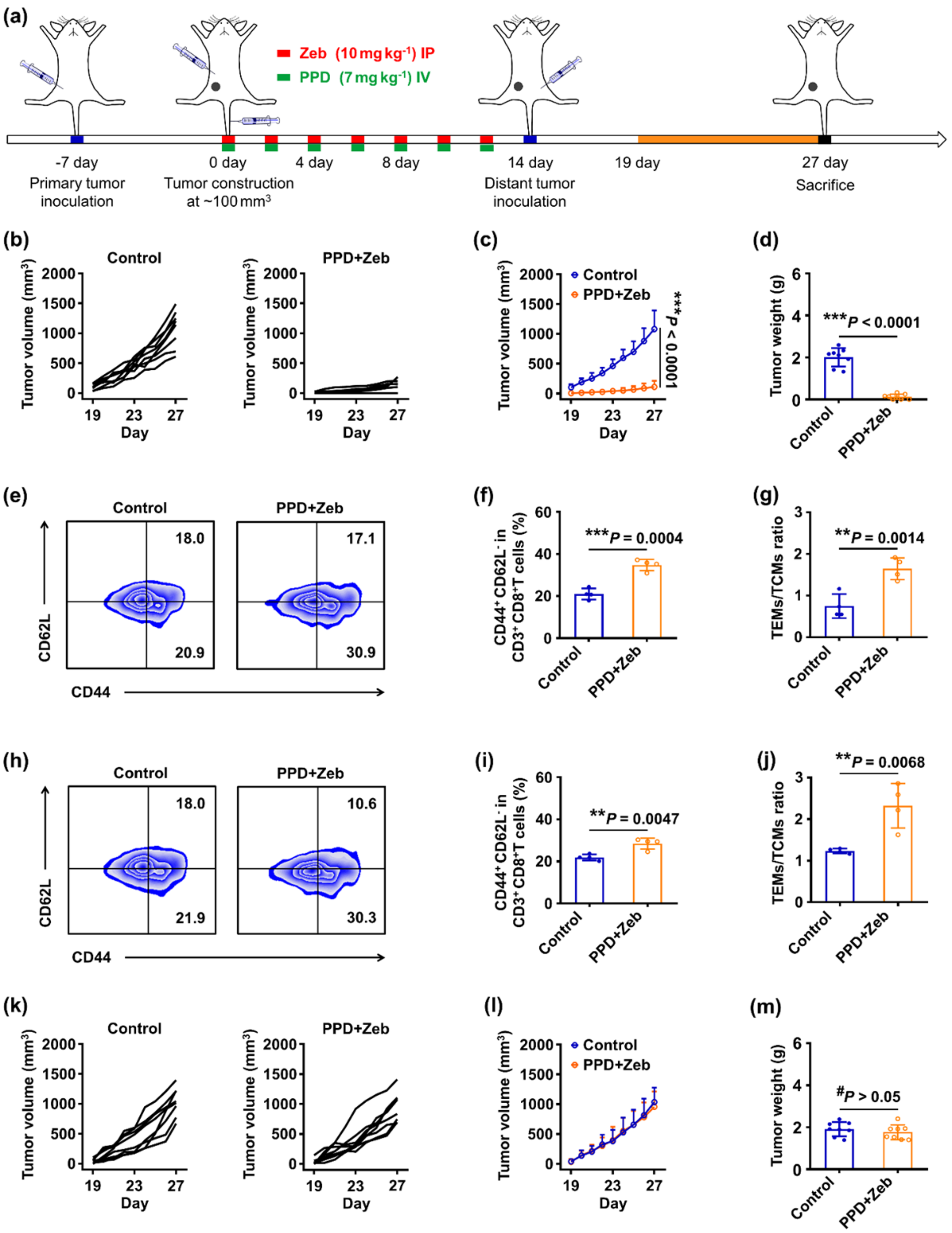

Fig. R32 (i.e. Supplementary Fig. 89 in the Revised Supplementary Information) PPD combined with Zeb inhibited 4T1 tumor relapse and initiated tumor specific immune memory effect. (a) Therapeutic schedule for PPD plus Zeb mediated inhibition of tumor relapse. **(b)** Individual and **(c)** average tumor growth kinetics in distant 4T1 tumor-bearing mice receiving PPD

plus Zeb treatment. Data are presented as mean ± SD (n=8), ***P<0.001. Age- and body-weight-matched healthy mice were used as control. **(d)** Average tumor weight of distant 4T1 tumor-bearing mice receiving PPD plus Zeb treatment. Data are presented as mean ± SD (n=8), ***P<0.001. **(e)** Representative flow cytometric analysis of TEM ($CD44^{+}$ $CD62L^{-}$) and TCM ($CD44^{+}$ $CD62L^{+}$) cells gating on $CD8^{+}$ T cells in spleen of 4T1 tumor-bearing mice at the end of treatment. Relative quantification of **(f)** TEM cells gating on $CD8^{+}$T cells and **(g)** ratio of TEMs/TCMs gating on $CD8^{+}$T cells in the spleen of 4T1 tumor-bearing mice at the end of treatment. Data are presented as mean ± SD (n=4), **P<0.01, ***P<0.001. **(h)** Representative flow cytometric analysis of TEM ($CD44^{+}$ $CD62L^{-}$) and TCM ($CD44^{+}$ $CD62L^{+}$) cells gating on $CD4^{+}$ T cells in spleen of 4T1 tumor-bearing mice at the end of treatment. Relative quantification of **(i)** TEM cells gating on $CD4^{+}$T cells and **(j)** ratio of TEMs/TCMs gating on $CD4^{+}$T cells in the spleen of 4T1 tumor-bearing mice at the end of treatment. Data are presented as mean ± SD (n=4), **P<0.01, ***P<0.001. **(k)** Individual and **(l)** average tumor growth kinetics in distant CT26 (murine colon carcinoma) tumor-bearing mice receiving PPD plus Zeb treatment. Data are presented as mean ± SD (n=8). Age- and body-weight-matched healthy mice were used as control. **(m)** Average tumor weight of distant CT26 tumor-bearing mice receiving PPD plus Zeb treatment. Data are presented as mean ± SD (n=8), $^{\#}P$>0.05.

11. The therapeutic effect observed in this research relies on tissue culture and cell line-based xenograft studies, which could significantly differ from the original tumors. Hence, the clinical relevance of the PPD-/+zebularine (DNMTi) approach is uncertain. To avoid overstatements, the authors should consider either repeating some key experiments (for example, Fig. 3b-d) on patient-derived xenograft and/or transgenic mouse cancer models, or expanding the Discussion section to fully describe the major limitations of the current research and provide possible strategies/directions to overcome them in the future studies.

Response:

Thanks very much for your suggestion. The supplementary data were shown in **Fig R33&R34** and **Table R2**.

Hu-HSC-NPG (**Hu**manized (**Hu**)-**H**ematopoietic **S**tem **C**ells transplanted (**HSC**)-**NPG** (**N**on-obese diabetes, **P**rotein kinase DNA-activated catalytic with severe combined immune deficiency, null interleukin-2 receptor **G**amma chain)) model was obtained from Beijing Vitalstar

Biotechnology Co.,Ltd, and the construction process of this model was as follows: $CD34^+$ cells were isolated and purified from human umbilical cord blood. NPG female mice aged 4 weeks were irradiated (1.6 Gy) for 4 hours, then $CD34^+$ cells were intravenously administered at dose of 1×10^5 cells per mice. After 20 weeks, the contents of human and mouse derived $CD45^+$ cells in mouse peripheral blood were detected by flow cytometry to test the qualified rate of Hu-HSC-NPG reconstruction (namely, the percentage of human $CD45^+$ cells exceeded 25%). The relevant data were presented in **Table R2**. Then Hu-HSC-NPG mice were inoculated subcutaneously with MDA-MB-231 cells at dose of 2×10^6 cells per mice, and Hu-HSC-NPG mice were randomly divided into two groups (three mice per group): PBS, PPD plus Zeb. When the average tumor volumes reached 50 mm^3, Hu-HSC-NPG mice were administered with PPD plus Zeb or PBS every two days for total five times.

The detailed descriptions were supplemented as follows: to verify PPD plus Zeb mediated antitumor effect in humanize mice model, humanized plasmid encoding shPD-L1 (abbreviated as hu-shPD-L1) was designed. According to RT-qPCR assay, m**P**EG-b-PLG/**P**EI-RT3/hu-shPD-L1 (denoted as **PPD**) significantly downregulated PD-L1 expression on MDA-MB-231 cells while mPEG-b-PLG/PEI-RT3/humanized control shRNA (denoted as PPD-Control) could hardly lower the level of PD-L1 on MDA-MB-231 cells (**Fig. R33**). Moreover, MDA-MB-231 tumor-bearing Hu-HSC-NPG model was constructed for further evaluating the antitumor effect of PPD plus Zeb in humanized mice. As shown in **Fig R34b&c**, compared with PBS group, PPD plus Zeb combination therapy could significantly inhibit the tumor growth. Moreover, the tumor weight data showed a consistent therapeutic effect (**Fig R34d**). The body weight of PPD plus Zeb group did not show significant changes throughout the process (**Fig R34e**). Furthermore, the relevant genes in tumor tissues were detected by RT-qPCR assay. Compared with PBS group, the level of PD-L1 in PPD plus Zeb group was significantly downregulated (**Fig R34f**). Moreover, MHC I molecules (HLA-A and HLA-B) and CTAs genes (SSX-1, SSX-2, SSX-4, SSX-5, and MAGE-1) were remarkably upregulated after PPD plus Zeb treatment. These results suggested PPD plus Zeb combination therapy not only downregulated PD-L1 expression on tumor cells to relieve the immunosuppression of T cells, but also induced the enhancement of MHC I molecules on tumor cells accompanying upregulation of antigen presentation machinery via CTAs signaling, thereby ultimately mounting the visibility of tumor cells to the adaptive immune system in humanized mice model. According to the antitumor effect in humanized mice, PPD plus Zeb combination therapy had a broad prospect

for clinical application.

The relevant descriptions were supplemented in the Revised Manuscript (Page 29, Paragraph 2, Line 12-31, red words; Page 30, Paragraph 1, Line 1-2, red words).

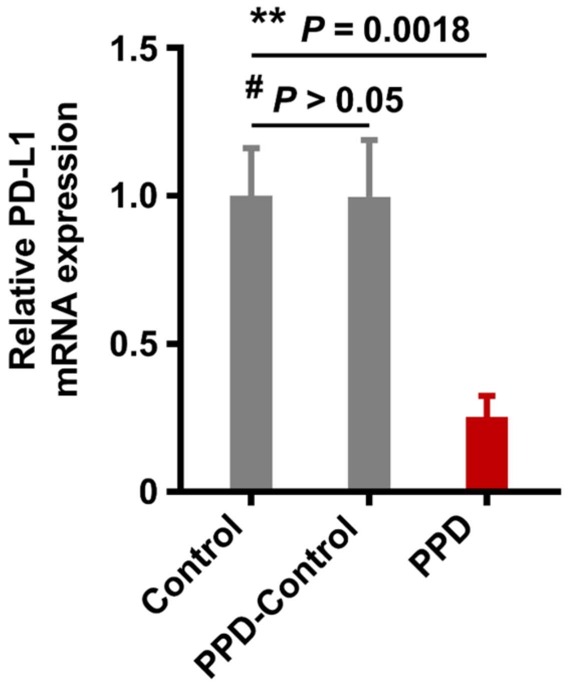

Fig R 33 (i.e. Supplementary Fig. 109 in the Revised Supplementary Information) Relative PD-L1 mRNA expression in MDA-MB-231 cells treated with PPD by RT-qPCR assay. Data are presented as mean ± SD (n=3), $^{\#}P>0.05$, $^{*}P<0.05$, $^{**}P<0.01$.

Table R2 (**i.e. Supplementary Table 5 in the Revised Supplementary Information**) Relevant parameters of Hu-HSC-NPG model mice.

Entry	$mCD45^{+}$ %MNCs [a]	$hCD45^{+}$ %MNCs [b]	$hCD3^{+}$ %$hCD45^{+}$ [c]
1	46.61%	53.39%	53.03%
2	32.66%	67.34%	39.80%
3	62.07%	37.93%	37.10%
4	56.63%	43.37%	62.23%
5	32.10%	67.90%	51.10%
6	47.59%	52.41%	60.07%

[a] The percentages of mouse CD45$^+$ cells in peripheral blood mononuclear cells (MNCs).

[b] The percentages of human CD45$^+$ cells in peripheral blood mononuclear cells.

[c] The percentages of human CD3$^+$ cells in human CD45$^+$ cells.

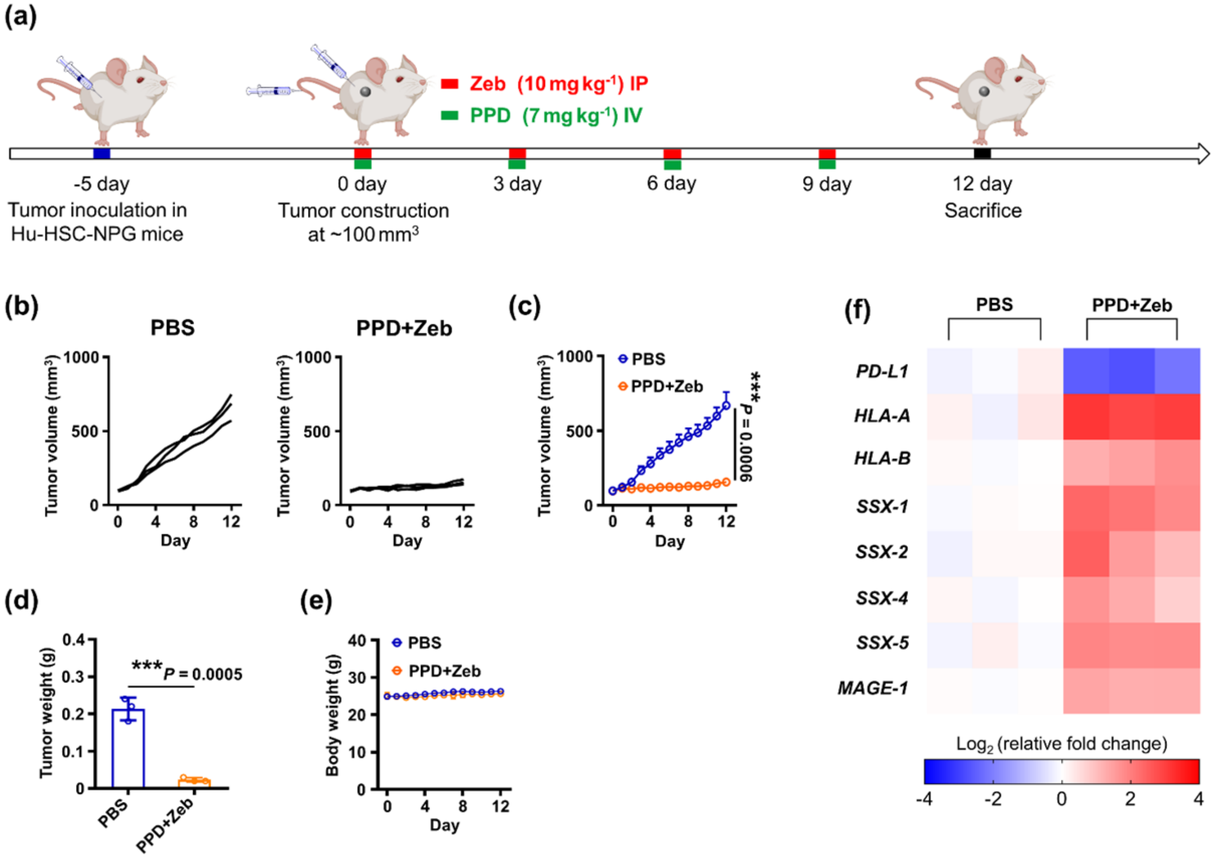

Fig R 34 (i.e. Fig. 8 in the Revised Manuscript) PPD combined with Zeb inhibited tumor growth in humanized mice model. (a) Therapeutic schedule for PPD plus Zeb mediated inhibition of tumor growth. **(b)** Individual and **(c)** average tumor growth kinetics in MDA-MB-231 tumor-bearing mice receiving PPD plus Zeb treatment. Data are presented as mean ± SD (n=3), ****P*<0.001. PBS was used as control. **(d)** Average tumor weight of MDA-MB-231 tumor-bearing mice receiving PPD plus Zeb treatment. Data are presented as mean ± SD (n=3), ****P*<0.001. **(e)** Average body weight changes of MDA-MB-231 tumor-bearing mice receiving PPD plus Zeb treatment. Data are presented as mean ± SD (n=3). **(f)** Heatmap of relative mRNA expression of PD-L1, MHC I (HLA-A and HLA-B), and CTAs (SSX-1, SSX-2, SSX-4, SSX-5, and MAGE-1) in tumor tissues of MDA-MB-231 tumor-bearing mice at the end of treatment.

12. The readability of the manuscript does not reach publication standards. Thorough proofreading and editing are required to improve the quality of writing prior to publication.

Response: Thanks very much for your kind suggestion. To improve the quality of writing, we have checked this manuscript carefully and improved it with the help of a native English speaker.

Reviewers' Comments:

Reviewer #1:
Remarks to the Author:
the authors extensively replied to the raised issues and performed a large set of additional experiments. these results strongly substantiated their findings and claims.

one small textual comments remains, related to my point 3: at row 50/51 the text still reads '... via cancer testis antigen (CTA), signaling.....', however CTA do not signal as in signal transduction. Cancer antigens are proteins presented by MHC-I molecules and that presentation is enhanced by DNMT inhibitors. please correct.

Reviewer #2:
Remarks to the Author:
The authors have addressed my comments.

Reviewer #3:
Remarks to the Author:
The authors have addressed all of my previous concerns. I would like to recommend this manuscript for publication, with a minor change:

It would be an overstatement to say that “depletion of certain MHC I molecules protected tumor growth against the zebularine/PPD treatment” (Page 14, Paragraph 2, Line 11-12), since siH2-Kb only provided the significant yet limited protection against PPD plus Zeb (Supplementary Fig. 73b-c). The authors should clarify this point in the final version of the manuscript.

Response to Reviewers

REVIEWER COMMENTS

Reviewer #1, expert in immune cell characterization/mouse models and therapy (Remarks to the Author):

REVIEWERS' COMMENTS

Reviewer #1 (Remarks to the Author): the authors extensively replied to the raised issues and performed a large set of additional experiments. these results strongly substantiated their findings and claims. One small textual comments remains, related to my point 3: at row 50/51 the text still reads '... via cancer testis antigen (CTA), signaling.....', however CTA do not signal as in signal transduction. Cancer antigens are proteins presented by MHC-I molecules and that presentation is enhanced by DNMT inhibitors. please correct.

Response:

Thanks very much for your kind suggestion. As you suggested, the relevant descriptions were modified as follows: DNA methyltransferase inhibitors (DNMTi) could not only upregulate the expression of tumor-associated antigens, but also induce the enhancement of major histocompatibility complex (MHC) class I molecules on tumor cells via cancer testis antigen (CTA), thereby ultimately mounting the visibility of tumor cells to the adaptive immune system.

The relevant descriptions were supplemented in in the Revised Manuscript (Page 2, Paragraph 2, Line 16-19, red words; Page 12, Paragraph 2, Line 19-22, red words).

Reviewer #2 (Remarks to the Author):

The authors have addressed my comments.

Response: Thanks very much for your kind suggestion again.

Reviewer #3 (Remarks to the Author):

The authors have addressed all of my previous concerns. I would like to recommend this manuscript for publication, with a minor change:

It would be an overstatement to say that “depletion of certain MHC I molecules protected tumor growth against the zebularine/PPD treatment” (Page 14, Paragraph 2, Line 11-12), since siH2-Kb only provided the significant yet limited protection against PPD plus Zeb (Supplementary Fig. 73b-c). The authors should clarify this point in the final version of the manuscript.

Response:

Thanks very much for your kind suggestion. The relevant descriptions were supplemented as follows: These results suggested that depletion of certain MHC I molecules provided the significant yet limited protection of tumor growth against Zeb plus PPD treatment.

The relevant descriptions were supplemented in in the Revised Manuscript (Page 13, Paragraph 2, Line 19-21, red words).
